# Evaluating and Validating Large Language Models for Health Education on Developmental Dysplasia of the Hip: 2-Phase Study With Expert Ratings and a Pilot Randomized Controlled Trial

**DOI:** 10.2196/73326

**Published:** 2026-01-19

**Authors:** Hui Ouyang, Gan Lin, Yiyuan Li, Zhixin Yao, Yating Li, Han Yan, Fang Qin, Jinghui Yao, Yun Chen

**Affiliations:** 1 Third Affiliated Hospital of Southern Medical University Guangzhou China; 2 School of Nursing Southern Medical University Guangzhou China; 3 Department of Pediatric Orthopedics Center for Orthopaedic Surgery Third Affiliated Hospital of Southern Medical University Guangzhou China

**Keywords:** large language models, developmental dysplasia of the hip, health education, content generation, mobile phone

## Abstract

**Background:**

Developmental dysplasia of the hip (DDH) is a common pediatric orthopedic disease, and health education is vital to disease management and rehabilitation. The emergence of large language models (LLMs) has provided new opportunities for health education. However, the effectiveness and applicability of LLMs in education with DDH have not been systematically evaluated.

**Objective:**

This study conducted an integrated 2-phase evaluation to assess the quality and educational effectiveness of LLM-generated educational materials.

**Methods:**

This study comprised 2 phases. Based on Bloom’s taxonomy, a 16-item DDH question bank was created through literature analysis and collaboration. Four LLMs (ChatGPT-4 [OpenAI], DeepSeek-V3, Gemini 2.0 Flash [Google], and Copilot [Microsoft Corp]) were questioned using standardized prompts. All responses were independently evaluated by 5 pediatric orthopedic experts using 5-point Likert measures of accuracy, fluency, and richness, the scales of Patient Education Materials Assessment Tool for Printable Materials, and DISCERN. The readability was measured by a formula. The data were examined using Kruskal-Wallis tests, ANOVA, and post hoc comparisons. In phase 2, an assessor-blinded, 2-arm pilot randomized controlled trial was conducted. A total of 127 caregivers were randomized into an LLM-assisted education group or a web search control group. The intervention included structured LLM training, supervised practice, and 2 weeks of reinforcement training. Measured at baseline, postintervention, and 2 weeks following, the outcomes were eHealth literacy (primary), DDH knowledge, health risk perception, perceived usefulness, information self-efficacy, and health information-seeking behavior. Cohen *d* effect sizes and linear mixed-effects models were used in an intention-to-treat manner.

**Results:**

There were significant differences between the 4 LLMs concerning accuracy, richness, fluency, Patient Education Materials Assessment Tool for Printable Materials Understandability, and DISCERN (*P*<.05). ChatGPT-4 (median 63.67, IQR 63.67-64.67) and DeepSeek-V3 (median 63.67, IQR 63.33-64.67) generate more accurate text than Copilot (median 59.00, IQR 58.67-59.67). DeepSeek-V3 (median 64.00, IQR 64.00-64.00) was language richer than Copilot (median 52.33, IQR 51.33-52.67). Gemini 2.0 Flash (median 72.67, IQR 72.33-73.00) was more fluent than Copilot (median 65.67, IQR 63.33-65.67). In phase 2, the intervention group showed higher eHealth literacy at T1 (33.62, 95% CI 32.76-34.49; *d*=0.20, 95% CI 0.13-0.56) and T2 (33.27, 95% CI 32.38-34.17; *d*=0.36, 95% CI 0.01-0.80), greater DDH knowledge at T1 (7.87, 95% CI 7.48-8.25, *d*=0.71, 95% CI 0.33-1.11) and T2 (7.12, 95% CI 6.72-7.51; *d*=0.54, 95% CI 0.17-0.96), and slight improvements in health risk prediction and perceived usefulness.

**Conclusions:**

Mainstream LLMs demonstrate varying capacities in generating educational content for DDH. They generated DDH caregiver education materials that were associated with modest improvements in eHealth literacy and knowledge. Although LLMs can address general informational needs, they cannot completely substitute clinical evaluation. Future research should focus on optimizing plain language, refining dialogue design, and enhancing audience personalization to improve the quality of LLMs’ materials.

**Trial Registration:**

Chinese Clinical Trial Registry ChiCTR2500108410; https://www.chictr.org.cn/showproj.html?proj=271987

## Introduction

### Background

Developmental dysplasia of the hip (DDH) is a common pediatric orthopedic condition affecting 1%-3% of infants, with a higher prevalence in girls and more frequent involvement of the left hip [1]. If undiagnosed or untreated early, DDH can lead to gait abnormalities, chronic pain, and early osteoarthritis, substantially affecting the quality of life [2]. Early diagnosis and health education are critical for improving prognosis. Delayed diagnosis and treatment often require complex surgery, which not only increases the difficulty of treatment but may also result in further functional deterioration [3,4]. Traditional educational methods are limited by time and resources, making it difficult to meet patients’ diverse informational needs. The emergence of artificial intelligence (AI) has provided new opportunities for health education.

In the broader field of digital health communication, AI-based conversational systems are increasingly being explored as tools to provide convenient and efficient services to meet people’s diverse needs. Currently, large language models (LLMs), such as ChatGPT, Google Gemini, Microsoft Copilot, and DeepSeek, are applied in health communication, including disease diagnosis [5], treatment recommendation [6], health education [7], and clinical decision-making [8]. For example, ChatGPT enables interactive discussions that tailor standardized medical information to individual patient needs, helping bridge communication gaps between clinicians and patients [9,10]. Although AI has demonstrated great potential in medical education, its use in patient-facing communication raises concerns. LLMs may provide erroneous medical advice [11], propagate outdated medical views [12], or fabricate nonexistent medical cases to generate “hallucinations” [13]. At the ethical and regulatory levels, challenges arise from the model’s “black box” decision-making, including unclear accountability, difficulties in defining legal responsibility, privacy breaches, and lagging regulatory frameworks. These issues directly jeopardize users’ safety, potentially leading to misdiagnosis, delayed treatment, and other forms of direct harm. Furthermore, most generated content maintains a university reading level, which may pose comprehension challenges for users without higher education [14]. These risks underscore the need for systematic evaluation before integrating such tools into health education.

While prior studies have primarily examined the accuracy or readability of LLM-generated content [15], few have connected content quality with its actual educational impact on end users. The extent to which LLM-generated materials can effectively support caregivers’ understanding and health literacy in specific conditions, such as DDH, remains unexplored. In DDH, caregivers have to not only comprehend specialized medical concepts but also actively recognize abnormal signs in children and make timely decisions [16]. Due to the professional complexity of orthopedic knowledge and the unique nature of pediatric disorders, basic health literacy abilities are necessary for caregivers. Therefore, the different levels of digital literacy among caregivers may make it more difficult for them to properly understand information produced by LLMs. To address this, the present study systematically evaluated multiple mainstream LLMs through expert assessment and a pilot randomized controlled trial (RCT) among caregivers. By integrating expert evaluation with caregiver validation, this study extends the current AI in health communication research from theoretical assessment to empirical verification.

### Objective

Therefore, this study aimed to provide a comprehensive evaluation and verification of LLM-generated education materials for DDH. The first phase assessed the educational quality of the outputs generated by 4 mainstream LLMs (ChatGPT-4, DeepSeek-V3, Gemini 2.0 Flash, and Copilot) through expert ratings of accuracy, understandability, actionability, and readability. The second phase involved a pilot RCT among caregivers to evaluate the actual educational impact of these materials, including digital literacy, DDH knowledge acquisition, health risk perception, information self-efficacy, perceived usefulness, and health information-seeking behaviors. This study bridged the gap by integrating the quality assessment of LLMs with RCT to validate their content reliability and educational impact. It offered evidence for the safe and effective use of LLMs in clinical education.

## Methods

### Theoretical Framework

The taxonomy by Bloom et al [17] served as the guiding pedagogical framework for designing the educational content. The taxonomy organizes cognitive processes into 6 hierarchical levels: remember, understand, apply, analyze, evaluate, and create, which is widely used to structure learning objectives and instructional materials. As users often need to acquire not only basic factual knowledge but also practical decision-making skills, this hierarchical model provided a structured approach for determining which levels of cognition should be targeted in education [18].

Guided by this framework, we developed a 16-item question bank that intentionally spanned different cognitive levels, ranging from foundational knowledge such as definitions and symptoms to more complex tasks such as interpreting clinical scenarios or making care decisions (Table 1). This ensured that the LLM-generated responses covered the breadth of learning needs relevant to caregivers. Bloom’s taxonomy, therefore, supported the construction of a comprehensive and pedagogically meaningful learning set, helping align the generated content with education requirements.

**Table 1:** Question bank framework based on Bloom’s taxonomy.

Bloom’s taxonomy and part			Content
**Remembering**			
	Basics	“What is [disease name]? Please explain its main features and potential effects in simple language.”	
	Anatomy and effects	“Who is most likely to develop [disease name]? How common is it?”	
**Understanding**			
	Etiology and risk factors	“Will [disease name] have a long-term impact on my quality of life? What aspects should I pay special attention to?”	
	Symptoms and early recognition	“What parts of the body does [disease name] mainly affect? What do these parts do in a healthy state?”	
**Applying**			
	Physician examination and diagnosis	“What symptoms or consequences may arise if these parts are damaged? Can you provide specific examples?”	
	Emergencies	“What are the main causes of [disease name]? What risk factors may increase the chances of developing it?”	
**Analyzing**			
	Hospital treatment and rehabilitation	“What lifestyle habits or environmental factors increase the risk of developing [disease name]? How can I prevent it?”	
	Medication management	“If I have a family history, is my risk of developing [disease name] higher? What preventive measures should I take?”	
	Pain management	“What are the main symptoms of [disease name]? What signs indicate the disease is worsening? Should I seek immediate medical attention?”	
**Evaluating**			
	Postoperative management	“What methods do doctors usually use to confirm the diagnosis of [disease name]? What is the purpose of each test?”	
**Creating**			
	Daily living and health management	“What indicators are most important in test results? How do these results reflect the severity of the disease?”	
	Mental health	“After confirming the diagnosis of [disease name], do I need to schedule regular follow-ups? How often should I have them and what should be checked?”	
	Digital tools and management	“What symptoms indicate that I need to seek immediate medical attention?”	
**Expanding^a^**			
	Education and support resources	“How should I handle and self-monitor during an emergency situation?”	
	Social and economic impact	“What treatments will I receive during hospitalization? What is the purpose of each treatment?”	
	Future planning and visioning	“How can I prevent infections or other complications during hospitalization? What can family members do to assist with recovery?”	

^a^Not part of Bloom’s taxonomy; it is an extension of this study.

### Study Design

The evaluation study had 2 phases. Phase 1 was a cross-sectional study in which physicians evaluated the answers provided by the LLMs. Phase 2 was a 2-arm pilot RCT comparing health education using LLMs with web-based searches.

### Phase 1: Expert Evaluation Study

#### Model Testing

Based on Bloom’s taxonomy, we collected and categorized common questions regarding DDH. We also reviewed clinical guidelines [19-23] to identify the key areas of knowledge. Using this information, an initial question bank was developed, which was subsequently refined and finalized through expert review. Each question was guided by a harmonized prompt paragraph: “Using developmental dysplasia of the hip (DDH) in children as an example, answer the following questions in detail, ensuring the content is easily understandable for non-medical professionals. Life-like examples and situations can be incorporated to help readers better grasp the information. Please reduce the number of syllables to make the sentence simpler.” All the generated texts and the complete question bank are provided in Multimedia Appendix 1. Each model generated educational materials based on 16 question banks, and the experiment was repeated 3 times for a total of 192 generations (4 models × 16 question banks × 3 times). Data were collected from January to February 2025. ChatGPT-4, DeepSeek-V3, Gemini 2.0 Flash, and Copilot were evaluated; no experimental, beta, or preview releases were included. The experiments were performed under the default settings of the web interfaces without modifying the generation parameters. To ensure reproducibility and independence of the outputs, each prompt was regenerated 3 times by establishing a new session for each run with the same original prompt. All outputs, including identical or similar responses, were retained to reflect the intrinsic variability of the models.

#### Assessment of Quality and Readability

Quality assessment tools as primary outcomes included (1) a Likert scale for assessing three items of accuracy, fluency, and richness of the material, scoring 16 questions on a scale from 1 to 5, with higher scores indicating better performance; (2) the DISCERN tool [24], which assessed the overall quality of the educational material, with a total of 16 entries, scoring on a scale from 1 to 5, with higher scores indicating better quality; and (3) the Patient Education Materials Assessment Tool for Printable Materials (PEMAT-P) [25], which contains 17 items measuring understandability and 7 items assessing actionability. These were reduced to 10 and 4 to accommodate the textual output with a 70% passing line based on the guidelines. During the evaluation process, each material was independently scored by 5 evaluators. The scores from evaluators were retained for subsequent data analysis.

Readability assessment tools as secondary outcomes included (1) the Flesch-Kincaid Reading Ease (FKRE), (2) the Flesch-Kincaid Grade Level (FKGL), and (3) the Simple Measure of Gobbledygook (SMOG) index, chosen for their widespread use and reliability in assessing text readability. All 3 score calculations involved the total number of words, sentences, and syllables. The FKRE measured the simplicity of the text, with scores ranging from 0 to 100, with higher scores indicating better readability. The FKGL represented reading level grade, with lower FKGL and SMOG indicating better comprehension and higher scores indicating more complex language. Scores above 60 or below sixth grade were the recommended reading levels for the general public. Readability scores were calculated using a web-based readability calculator (Readable; Added Bytes). The detailed formulas are provided in Multimedia Appendix 2.

#### Expert Evaluation

The material generated by the LLMs was independently assessed for quality. The material generated by the LLMs was independently assessed by 5 pediatric orthopedic physicians with expertise in DDH, selected through rigorous predefined criteria: (1) ≥10 years of clinical experience in DDH diagnosis or treatment; and (2) evaluators completed standardized training on the assessment rubric before this study, using the DDH guidelines as the gold standard [19-23]. To ensure blinding, the LLM outputs were made anonymous by an independent researcher who replaced the model names with random codes. The evaluators confirmed that they could not infer the identities of the LLMs or determine if repeated outputs came from the same model. Interrater reliability was assessed for each outcome dimension using the intraclass correlation coefficient (ICC). ICC values were interpreted as follows: <0.5=poor, 0.5-0.75=moderate, 0.75-0.9=good, and >0.9=excellent agreement.

### Phase 2: Pilot RCT

#### Participants

Participants were recruited through digital media advertisements and physician referrals. Eligibility criteria included (1) being aged ≥18 years, (2) being caregivers of children aged 0-14 years, (3) having the ability to read and understand words, and (4) having internet access. Exclusion criteria included (1) having severe hearing or visual impairment; (2) having severe schizophrenia, major depression, bipolar disorder, and other mental illnesses; or (3) participation in other related studies.

#### Sample Size

Power analysis was performed using G*Power 3.1.9.7 based on similar educational intervention studies [26]. A medium effect size (Cohen *d*=0.65) was anticipated for the primary outcome of performance, with 2-tailed α=.05, power of 0.8, and at least 38 participants per group being required. Accounting for an expected 20% attrition rate, the target sample was 49 participants per group (total n=98). There were 127 participants in the final sample (62 in the control group and 65 in the intervention group).

#### Randomization and Blinding

Recruitment took place in the Third Affiliated Hospital of Southern Medical University and community support groups. The researchers generated a computer-generated list and sealed it in an opaque envelope. Before the start of the intervention, research assistants who were not involved in hospital assessments or interventions opened the envelopes and assigned participants at random to the intervention or control group. Following informed consent, eligible participants meeting the inclusion and exclusion criteria were randomly assigned in a 1:1 ratio to either the trial or control group. The blinding of participants was not feasible due to the nature of the intervention, but the research team remained unaware of group assignments until this study concluded. Data analysts who conducted the final analyses were masked to participant identities throughout. Due to the nature of the intervention, participant blinding was not possible. However, group allocations were not disclosed to the research team until the trial was finished. Throughout, participant identities were concealed from the data analysts.

#### Control Group

Participants in the control group received standard web-based educational materials prepared by clinical experts. These materials were retrieved from official sources (eg, [27]). Participants were asked to read independently, simulating a typical web-based health information-seeking behavior.

#### Intervention Group

All researchers received standardized training to ensure consistent delivery of DDH-related information and LLM education. The intervention was delivered to participants by face-to-face communication. First, the participants were introduced to the foundational concepts of LLMs, including basic mechanisms, application categories, and core interaction capabilities. Second, a standardized consultation framework was introduced, covering device access, platform login, dialogue initiation, and structured prompt formulation. The required background information included demographic and clinical characteristics, symptom description, disease duration, medical history, lifestyle, and psychosocial factors. Participants were also provided with 16 DDH-related inquiry categories, including foundations, risk factors, early recognition, diagnosis, treatment, postoperative care, medication management, psychological support, etc. They are able to optionally output custom instructions, such as length, style, level of technical terminology, and formatting preferences. Third, strategies to improve information quality are introduced, including clear language prompts, staged questions, example guidance, support for the reasoning process, evidence sources for web retrieval, and document import. Verification approaches were emphasized, such as cross-model comparison, guideline checking, and professional consultation. Finally, risk awareness and ethical considerations were reinforced, including potential hallucinations, outdated content, privacy risks, copyright issues, and inappropriate clinical dependence. A practical demonstration was conducted using an actual DDH case. For example, a female infant, 1 year old, with asymmetric thigh folds and a family history, but no medical history. Participants inquired and learned relevant knowledge based on the background of this example. During the 2 weeks, the participants received remote support through web-based group consultations or offline feedback sessions. Researchers responded to questions related to practical application, corrected misuse behaviors, and supplemented individualized guidance.

#### Data Collection

Data were collected through questionnaire surveys. The basic information questionnaire gathered the demographic characteristics of this study’s participants. Validated scales were used to measure eHealth literacy, DDH knowledge, health risk perception, information self-efficacy, perceived usefulness, and health information-seeking behavior. There were three assessment time points: (1) baseline (T0), (2) immediately after the completion of the intervention or control group (T1), and (3) two weeks after the end of the intervention or control group (T2).

#### Primary Outcomes

The eHealth Literacy Scale (eHEALS), originally developed by Norman and Skinner [28], was adopted to measure participants’ eHealth literacy. It comprises 8 items that assess one’s ability to locate and use web-based health resources, appraise the credibility of digital health information, and apply acquired information to make informed health decisions. Each item is scored on a 5-point Likert scale, producing a total score between 8 and 40, with higher scores representing stronger eHealth literacy.

#### Secondary Outcomes

The developmental dysplasia of the hip knowledge test (DDH-KT) was developed by the research team to assess participants’ basic DDH knowledge. The items were constructed according to current clinical guidelines and health education materials and reviewed by pediatric orthopedic surgeons. Each correct answer is scored as 1 point (range 0-10), with higher scores indicating greater DDH knowledge. The full knowledge test is provided in Multimedia Appendix 3.

The Health Risk Perception Scale (HRPS) was measured based on the framework by Ajzen [29]. The scale was adapted from established health risk perception measures by Brewer et al [30], and covered 2 dimensions: perceived susceptibility and perceived severity. The items assessed participants’ subjective perception of the likelihood and potential consequences of related health problems, rated on a 5-point Likert scale. Higher scores reflected a greater level of perceived risk (Cronbach α=0.847).

The Information Self-Efficacy Scale (ISES), adapted from Pavlou and Fygenson [31], was used to evaluate participants’ confidence in obtaining and effectively using web-based health information. The scale contained 3 items rated on a 5-point Likert scale. Total scores were calculated by summing all item responses, with higher scores indicating stronger information self-efficacy (Cronbach α=0.806).

The Perceived Usefulness Scale (PUS), adapted from Cheung et al [32], assessed the extent to which participants viewed web-based health information as helpful, relevant, and beneficial for health knowledge and decision-making. Items were scored on a 5-point Likert scale, with higher scores indicating greater perceived usefulness (Cronbach α=0.852).

The Health Information-Seeking Behavior Scale (HISBS), adapted from Kankanhalli et al [33], measured the frequency and willingness to actively seek web-based health information. Responses were recorded using a 5-point Likert scale, and higher scores indicated more proactive seeking behavior (Cronbach α=0.873).

### Statistical Analysis

In phase 1, descriptive statistics were reported as mean (SD) and median (IQR). Because the final analytic values were obtained by averaging 3 generations, the normality assumptions for repeated-measures ANOVA were not met. Group differences among the 4 LLMs were analyzed using the Kruskal-Wallis H test, followed by Dunn-Bonferroni post hoc comparisons when significant. One-way ANOVA and Tukey post hoc tests were used for readability indices because the normality assumptions were satisfied. False discovery rate correction was applied across the 9 outcomes to control for multiple testing. Interrater reliability was assessed using ICC(2,k) based on a 2-way random-effects model [34]. Effect sizes were reported as epsilon-squared for nonparametric tests and eta-squared for ANOVA. Analyses were conducted in R (version 4.5.1; R Foundation) with ggplot2 (version 3.5.1; Posit, PBC) for visualization.

In phase 2, all analyses followed the intention-to-treat principle and included all randomized participants. Continuous baseline variables are presented as mean (SD), and categorical variables as counts and percentages. Differences between groups at baseline were assessed using 2-sided independent sample *t* tests for continuous variables and chi-square tests for categorical variables. Outcomes were analyzed using linear mixed-effects models with time (T1 and T2) and group (intervention vs control) as fixed effects, time × group interaction, baseline (T0) as a covariate, and participant ID as a random intercept. No imputation was performed because linear mixed-effects models estimated with restricted maximum likelihood provided unbiased estimates under the missing at random assumption [35]. Between-group effect sizes (Cohen *d*, 95% CI) and estimated marginal means (95% CI) were reported. eHEALS was defined as the primary outcome. All other outcomes, including DDH-KT, HRPS, ISES, PUS, and HISBS, were considered secondary. Given the pilot and exploratory nature of this trial, no adjustment for multiple comparisons was applied. Therefore, analyses of the outcomes were intended to be hypothesis-generating rather than confirmatory. Analyses were conducted in R (version 4.5.1) using lme4, lmerTest, and emmeans; 2-sided *P*<.05 was considered statistically significant.

### Ethical Considerations

This study was approved by the Ethics Committee of the Third Affiliated Hospital of Southern Medical University (2024-ER-113), and the first participant was enrolled in June 2025. The trial registration was completed on August 29, 2025, at the Chinese Clinical Trial Registry (ChiCTR2500108410). All research participants signed written informed consent forms. Researchers disclosed study information to participants; participants retained the right to withdraw from the study or withdraw their research data at any time without conditions, and withdrawal would not result in any adverse consequences. Participants were informed that part of the educational content was generated by AI, and the limitations of AI-generated information were explained. The use of AI-assisted materials was supervised throughout this study by qualified health care professionals. During the intervention period, participants were encouraged to report any concerns or adverse experiences related to the educational materials, and ultimately, no related adverse events were reported. All personal information and data collected during the study were kept strictly confidential. Participants who completed the entire study process received educational materials, including a parenting knowledge handbook valued at CN ¥50 RMB (approximately US $7.15), as compensation.

## Results

### Phase 1

#### Overview

**Figure 1:**
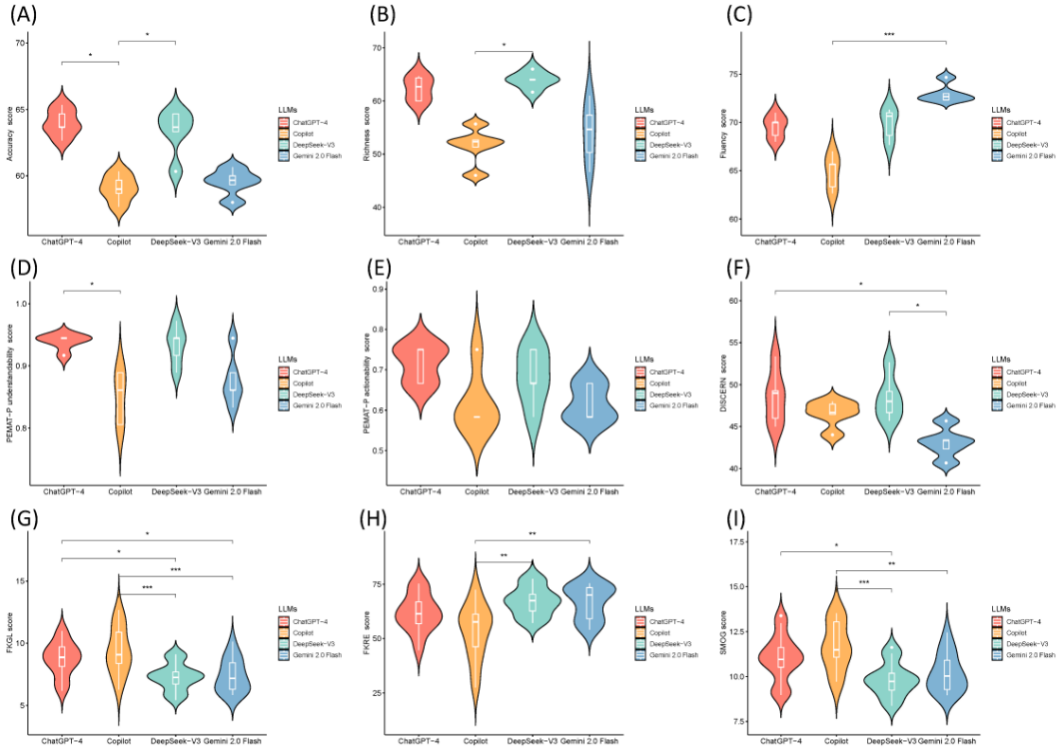
Comparison of responses across 4 LLMs. (A) Accuracy, (B) richness, (C) fluency, (D) PEMAT-P understandability, (E) PEMAT-P actionability, (F) DISCERN, (G) FKGL, (H) FKRE, and (I) SMOG index. FKGL: Flesch-Kincaid Grade Level; FKRE: Flesch-Kincaid Reading Ease; LLM: large language model; PEMAT-P: Patient Education Materials Assessment Tool for Printable Materials; SMOG: Simple Measure of Gobbledygook.

Overall, ChatGPT-4 and DeepSeek-V3 demonstrated the strongest performance in content accuracy, richness, understandability, and information quality, making them suitable for generating pediatric health communication materials. Gemini 2.0 Flash and Copilot performed well in fluency and readability metrics, while they were relatively weaker in content richness and accuracy. Table 2 provides a visual summary of the scores and the overall performance comparison. Figure 1 illustrates the comparison of the responses across the LLMs. The scoring data are presented in Multimedia Appendix 4.

**Table 2:** Comparison of model performance across different indicators.

Model		Mean (SD)	Median (IQR)		H/F^a^	*P* value	FDR-adjusted^b^ *P* value	ε²^c^/η²^d^	Significance (*P* value)
**Accuracy**					13.873	.003	.005	0.73	
	ChatGPT-4	64 (1.03)		63.67 (63.67-64.67)					*^e,f^ (.02)
	Copilot	59.07 (1.01)		59.00 (58.67-59.67)					*^g^ (.048)
	DeepSeek-V3	63.33 (1.78)		63.67 (63.33-64.67)					—^h^
	Gemini 2.0 Flash	59.53 (0.99)		59.67 (59.33-60.00)					—
**Richness**					13.68	.003	.005	0.72	
	ChatGPT-4	62.33 (2.26)		62.67 (60.00-64.33)					—
	Copilot	51.6 (3.52)		52.33 (51.33-52.67)					*^g^ (.02)
	DeepSeek-V3	63.93 (1.53)		64.00 (64.00-64.00)					—
	Gemini 2.0 Flash	54 (5.65)		54.67 (50.33-57.33)					—
**Fluency**					16.204	.001	.003	0.853	
	ChatGPT-4	69.53 (1.19)		70.00 (68.67-70.00)					—
	Copilot	64.87 (1.8)		65.67 (63.33-65.67)					***^i^ (<.001)
	DeepSeek-V3	69.87 (2.19)		70.67 (70.33-71.00)					—
	Gemini 2.0 Flash	73 (0.97)		72.67 (72.33-73.00)					—
**PEMAT-P^j^ understandability (%)**					11.421	.01	.012	0.601	
	ChatGPT-4	93.89 (1.24)		94.44 (94.44-94.44)					*^f^ (.03)
	Copilot	85 (4.21)		86.11 (80.56-88.89)					—
	DeepSeek-V3	93.33 (3.17)		94.44 (91.67-94.44)					—
	Gemini 2.0 Flash	87.78 (4.21)		86.11 (86.11-88.89)					—
**PEMAT-P actionability (%)**					7.587	.06	.06	0.399	
	ChatGPT-4	68.33 (3.73)		66.67 (66.67-66.67)					—
	Copilot	60 (6.97)		58.33 (58.33-66.67)					—
	DeepSeek-V3	68.33 (3.73)		66.67 (66.67-66.67)					—
	Gemini 2.0 Flash	66.67 (5.89)		66.67 (66.67-66.67)					—
**DISCERN**					10.243	.02	.02	0.539	
	ChatGPT-4	48.52 (3.27)		49.00 (46.00-49.27)					*^i^ (.035)
	Copilot	46.56 (1.57)		46.67 (46.47-47.67)					—
	DeepSeek-V3	48.44 (2.71)		48.00 (46.67-49.20)					*^i^ (.03)
	Gemini 2.0 Flash	43.08 (1.82)		43.33 (42.33-43.40)					—
**FKGL^k^**					8.395	<.001	.003	0.296	
	ChatGPT-4	8.74 (1.37)		8.86 (8.14-9.70)					*^g^ (.03); *^i^ (.04)
	Copilot	9.41 (1.86)		9.07 (8.38-10.88)					***^g^ (<.001); ***^i^ (<.001)
	DeepSeek-V3	7.30 (1.08)		7.26 (6.72-7.70)					—
	Gemini 2.0 Flash	7.37 (1.33)		7.19 (6.30-8.44)					—
**FKRE^l^**					14.198	.003	.005	0.225	
	ChatGPT-4	61.86 (8.58)		61.44 (56.80-66.97)					—
	Copilot	53.45 (12.62)		57.70 (46.25-61.33)					**^g^ (.006); **^i^ (.009)
	DeepSeek-V3	67.19 (6.62)		67.45 (62.73-70.43)					—
	Gemini 2.0 Flash	66.85 (7.72)		70.10 (59.19-73.48)					—
**SMOG^m^**					8.297	<.001	.003	0.293	
	ChatGPT-4	11.02 (1.26)		10.96 (10.52-11.61)					*^g^ (.02)
	Copilot	11.67 (1.33)		11.48 (11.09-13.04)					***^g^ (<.001); **^i^ (.003)
	DeepSeek-V3	9.83 (0.93)		9.74 (9.25-10.19)					—
	Gemini 2.0 Flash	10.19 (1.06)		10.03 (9.28-10.91)					—

^a^H/F: values are reported as test statistics. H statistics for the Kruskal-Wallis test and *F* statistics for 1-way ANOVA.

^b^FDR: false discovery rate.

^c^η²: eta-squared.

^d^ε²: epsilon-squared.

^e^*P <.05. **P <.01. ***P <.001. Normality was assessed for all variables. FKGL and SMOG were analyzed using 1-way ANOVA with Tukey honestly significant difference for pairwise comparisons; others were analyzed using the Kruskal-Wallis test with Dunn test (Bonferroni-corrected *P* values).

^f^vs CoPilot.

^g^vs DeePseek-V3.

^h^Not applicable.

^i^vs Gemini 2.0 Flash.

^j^PEMAT-P: Patient Education Materials Assessment Tool for Printable Materials.

^k^FKGL: Flesch-Kincaid Grade Level.

^l^FKRE: Flesch-Kincaid Reading Ease.

^m^SMOG: Simple Measure of Gobbledygook.

#### Quality Assessment

There were significant differences between the 4 LLMs in terms of accuracy, richness, fluency, PEMAT-P understandability, and DISCERN (*P*<.05). ChatGPT-4 and DeepSeek-V3 outperformed the other models in the majority of evaluation dimensions. ChatGPT-4 (median 63.67, IQR 63.67-64.67) and DeepSeek-V3 (median 63.67, IQR 63.33-64.67) generated more accurate text than Copilot (median 59.00, IQR 58.67-59.67). DeepSeek-V3 (median 64.00, IQR 64.00-64.00) was language richer than Copilot (median 52.33, IQR 51.33-52.67). Gemini 2.0 Flash (median 72.67, IQR 72.33-73.00) was more fluent than Copilot (median 65.67, IQR 63.33-65.67). Based on the PEMAT-P understandability scores, the content of ChatGPT-4 (median 94.44%, IQR 94.44%-94.44%) was more comprehensible than that of Copilot (median 86.11%, IQR 80.56%-88.89%). The PEMAT-P actionability scores were similar across the models. ChatGPT-4 (median 49.00, IQR 46.00-49.27) and DeepSeek-V3 (median 48.00, IQR 46.67-49.20) had a higher DISCERN scale score than Gemini 2.0 Flash (median 43.33, IQR 42.33-43.40).

#### Readability Assessment

Readability metrics highlighted the differences among the models. Gemini 2.0 Flash (median 66.85, IQR 59.19-73.48) and DeepSeek-V3 (median 67.19, IQR 62.73-70.43) generated sentences with higher FKRE scores, indicating easier readability compared to Copilot (median 53.45, IQR 46.25-61.33). DeepSeek-V3 (mean 7.30, SD 1.08) and Gemini 2.0 Flash (mean 7.37, SD 1.33) produced sentences with superior FKGL scores compared to ChatGPT-4 (mean 8.74, SD 1.37) and Copilot (mean 9.41, SD 1.86). DeepSeek-V3 (mean 9.83, SD 0.93) and Gemini 2.0 Flash (mean 10.19, SD 1.06) produced texts with better SMOG scores compared to ChatGPT-4 (mean 11.02, SD 1.26) and Copilot (mean 11.67, SD 1.33).

#### Visualization and Analysis

**Figure 2:**
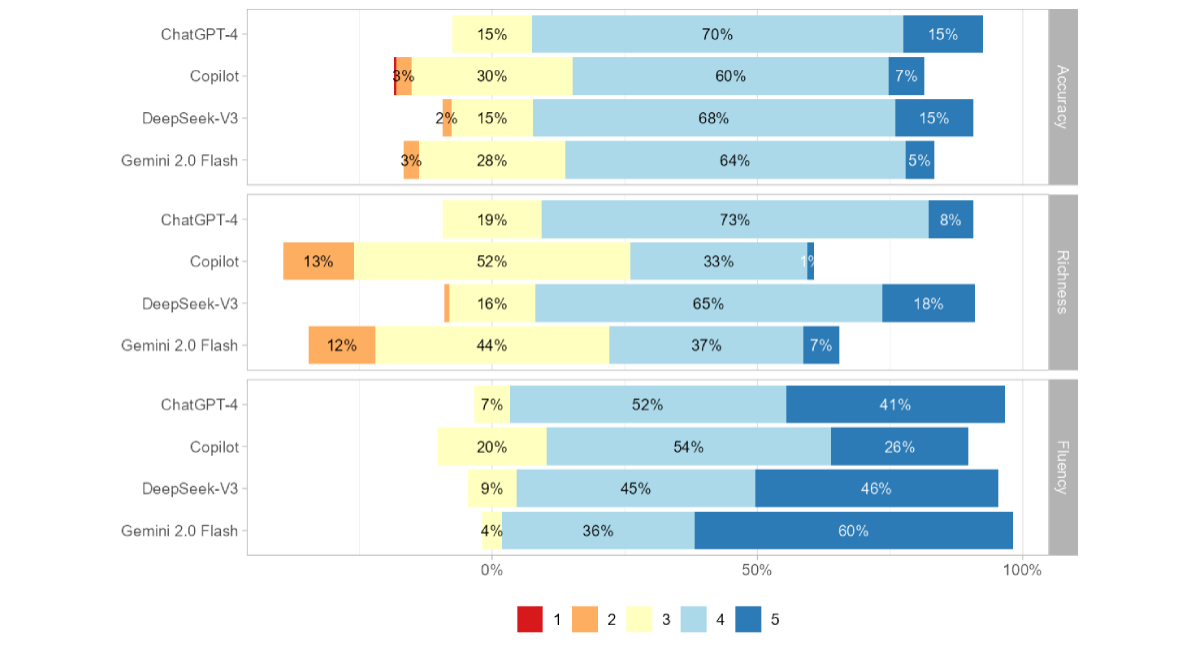
Expert Likert-scale ratings of content quality across 4 LLMs. LLM: large language model.

**Figure 3:**
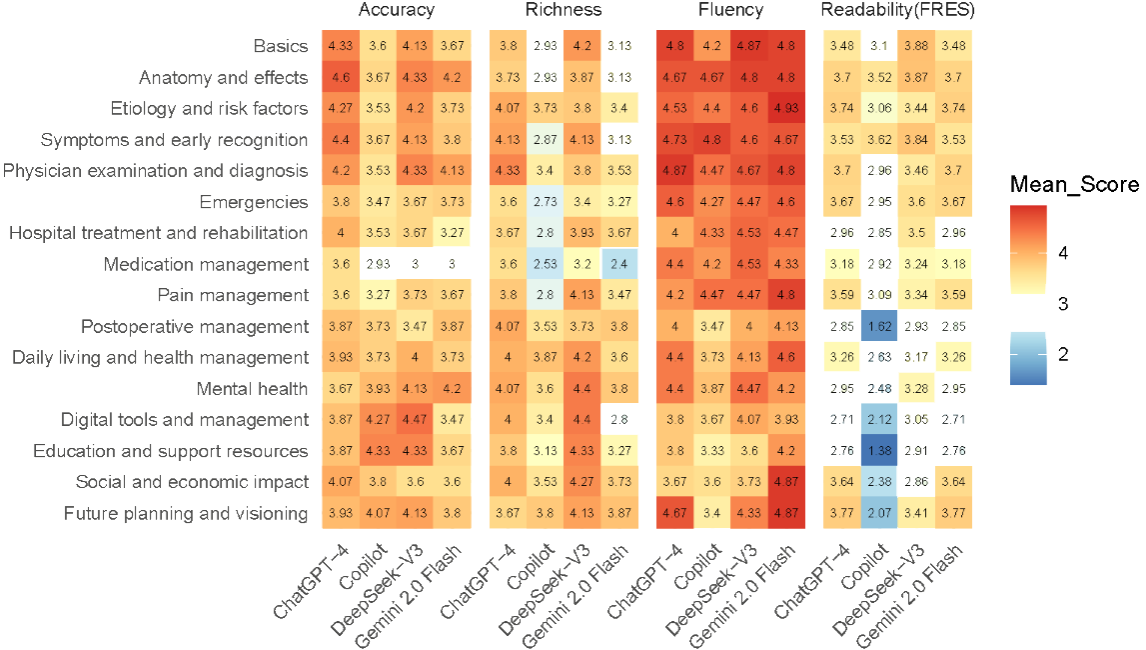
Multidimensional performance evaluation heatmap for LLMs. Heatmap showing mean scores of 4 LLMs across accuracy, richness, fluency, and readability dimensions. Higher scores are represented by warmer colors. FRES: Flesch-Kincaid Reading Ease; LLM: large language model.

The comparative evaluation of 4 LLMs demonstrated clear performance variability across accuracy, richness, and fluency, as illustrated in Figures 2 and 3. Overall, ChatGPT-4 and DeepSeek-V3 outperformed Copilot and Gemini Flash, particularly in accuracy and fluency. In terms of accuracy, the proportion of “good” and “excellent” responses reached 85% for ChatGPT-4 and 83% for DeepSeek-V3, while Gemini 2.0 Flash (70%) and Copilot (66%) displayed a lower proportion. Regarding richness, DeepSeek-V3 (83%) and ChatGPT-4 (81%) again ranked highest, reflecting strong supplementary and explanatory capability, whereas the other 2 models showed as more concise. Across fluency, all 4 models delivered strong information elaboration, with Gemini 2.0 Flash achieving the highest proportion of 96%, indicating strong coherence, readability, and natural language expression.

As shown in the heatmap (Figure 3), ChatGPT-4 and DeepSeek-V3 yielded higher mean scores across most knowledge domains, particularly in basic, effects, and symptoms. In contrast, Copilot and Gemini 2.0 Flash performed worse, especially in specialized domains such as medication management and postoperative care. These results suggested that current LLMs perform well in general health education content but remain limited in clinically nuanced and actionable information.

Across the 4 models and 6 evaluation dimensions, the interrater reliability among the 5 evaluators ranged from moderate to excellent (ICC=0.628-0.918). Table 3 shows the interrater reliability results across the 4 LLMs and evaluation dimensions based on ICC.

**Table 3:** Results of expert consistency analysis.

Dimension		ICC^a,b^	95% CI	*F* test (*df*)	*P* value	Interpretation	
**Accuracy**							
	ChatGPT-4	0.851	0.695-0.941	6.734 (15, 60)	<.001	Good	
	Copilot	0.654	0.277-0.864	2.810 (15, 60)	.002	Moderate	
	DeepSeek-V3	0.904	0.803-0.962	11.510 (15, 60)	<.001	Excellent	
	Gemini 2.0 Flash	0.727	0.433-0.892	3.565 (15, 60)	<.001	Moderate	
**Richness**							
	ChatGPT-4	0.669	0.352-0.864	3.499 (15, 60)	<.001	Moderate	
	Copilot	0.834	0.652-0.934	7.750 (15, 60)	<.001	Good	
	DeepSeek-V3	0.747	0.481-0.899	3.944 (15, 60)	.001	Moderate	
	Gemini 2.0 Flash	0.628	0.285-0.845	3.515 (15, 60)	<.001	Moderate	
**Fluency**							
	ChatGPT-4	0.914	0.825-0.966	11.931 (15, 60)	<.001	Excellent	
	Copilot	0.918	0.833-0.967	13.244 (15, 60)	<.001	Excellent	
	DeepSeek-V3	0.835	0.664-0.934	6.223 (15, 60)	<.001	Good	
	Gemini 2.0 Flash	0.876	0.746-0.951	8.038 (15, 60)	<.001	Good	
PEMAT-P^c^ understandability		0.874	0.482-0.991	7.833 (3, 12)	.004	Good	
PEMAT-P actionability		0.718	0.050-0.980	3.858 (3, 12)	.038	Moderate	
DISCERN		0.819	0.358-0.986	12.473 (3, 12)	.001	Good	

^a^ICC: intraclass correlation coefficient.

^b^Type A intraclass correlation coefficient using an absolute agreement definition.

^c^PEMAT-P: Patient Education Materials Assessment Tool for Printable Materials.

### Phase 2

#### Participant Characteristics

**Figure 4:**
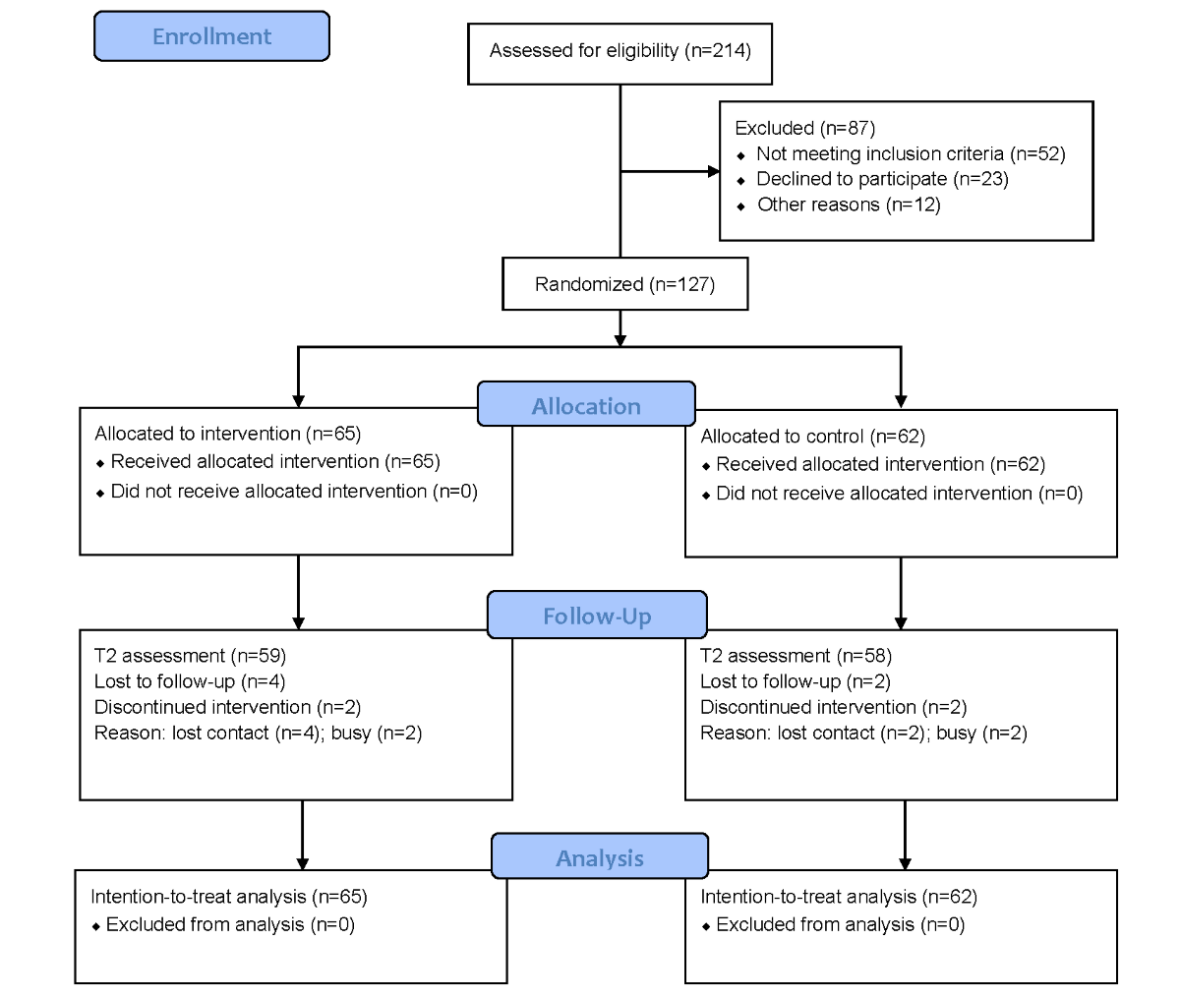
CONSORT diagram of study flow. CONSORT: Consolidated Standards of Reporting Trials.

Participants were recruited from June 2025 to September 2025. A total of 127 participants were enrolled in this study, including 65 in the intervention group and 62 in the control group. Figure 4 shows the CONSORT (Consolidated Standards of Reporting Trials) flowchart, and the CONSORT-EHEALTH (Consolidated Standards of Reporting Trials of Electronic and Mobile Health Applications and Online Telehealth) checklist is presented in Multimedia Appendix 5. Most participants completed the intervention, and the main reason for withdrawal was lack of time. Participants had a mean age of 36.57 (SD 6.22) years, and most were female (89/127, 70.07%) and highly educated (55/127, 43.31%). The mean age of participants’ children was 5.90 (SD 3.12) years. No significant differences were observed between the intervention and control groups in the baseline characteristics (*P*>.05). During this study, no privacy breaches, technical failures, or other unintended events were observed. Table 4 summarizes the demographic characteristics of the participants. The data of participants can be found in Multimedia Appendix 6.

**Table 4:** Baseline characteristics.

Background characteristic			Overall		Intervention group (n=65)		Control group (n=62)		*P* value	
Age (years), mean (SD)			36.57 (6.22)		37.06 (5.68)		36.05 (6.74)		.14	
**Sex, n (%)**										.83
	Female	89 (70.08)		45 (69.23)		44 (70.97)				
	Male	38 (29.92)		20 (30.77)		18 (29.03)				
**Education, n (%)**										.92
	Low education	18 (14.17)		11 (16.92)		7 (11.29)				
	Medium education	54 (42.52)		27 (41.54)		27 (43.55)				
	High education	55 (43.31)		27 (41.54)		28 (45.16)				
**Monthly income (CN ¥), n (%)**										.85
	≤3000 (US $429.12)	9 (7.09)		30 (61.2)		35 (71.4)				
	3001-6000 (US $429.26-US $858.23)	26 (20.47)		19 (38.8)		14 (28.6)				
	6001-10,000 (US $858.38-US $1430.39)	42 (33.07)		21 (33.87)		21 (32.31)				
	10,001-20,000 (US $1430.53-US $2860.78)	36 (28.35)		19 (30.65)		17 (26.15)				
	≥20,001 (US $2860.92)	14 (11.02)		7 (11.29)		7 (10.77)				
**Child’s gender, n (%)**										.23
	Male	71 (55.91)		32 (49.23)		24 (38.71)				
	Female	56 (44.09)		33 (50.77)		38 (61.29)				
Child’s age (years), mean (SD)			5.90 (3.12)		6.39 (3.36)		5.39 (2.78)		.06	
**Daily caregiving time for the child (hours/day), n (%)**										.37
	≤2	17 (13.39)		11 (16.92)		6 (9.68)				
	3-6	55 (43.31)		27 (41.54)		28 (45.16)				
	6-9	16 (12.6)		7 (10.77)		9 (14.52)				
	9-12	15 (11.81)		6 (9.23)		9 (14.52)				
	≥12	24 (18.9)		14 (21.54)		10 (16.13)				
**Smartphone proficiency, n (%)**										.53
	Very proficient	66 (51.97)		33 (50.77)		33 (53.23)				
	Basic proficient	22 (17.33)		14 (21.54)		8 (12.9)				
	Fairly proficient	33 (25.98)		15 (23.08)		18 (29.03)				
	Not proficient	6 (4.72)		3 (4.62)		3 (4.84)				

#### Primary Outcome

**Figure 5:**
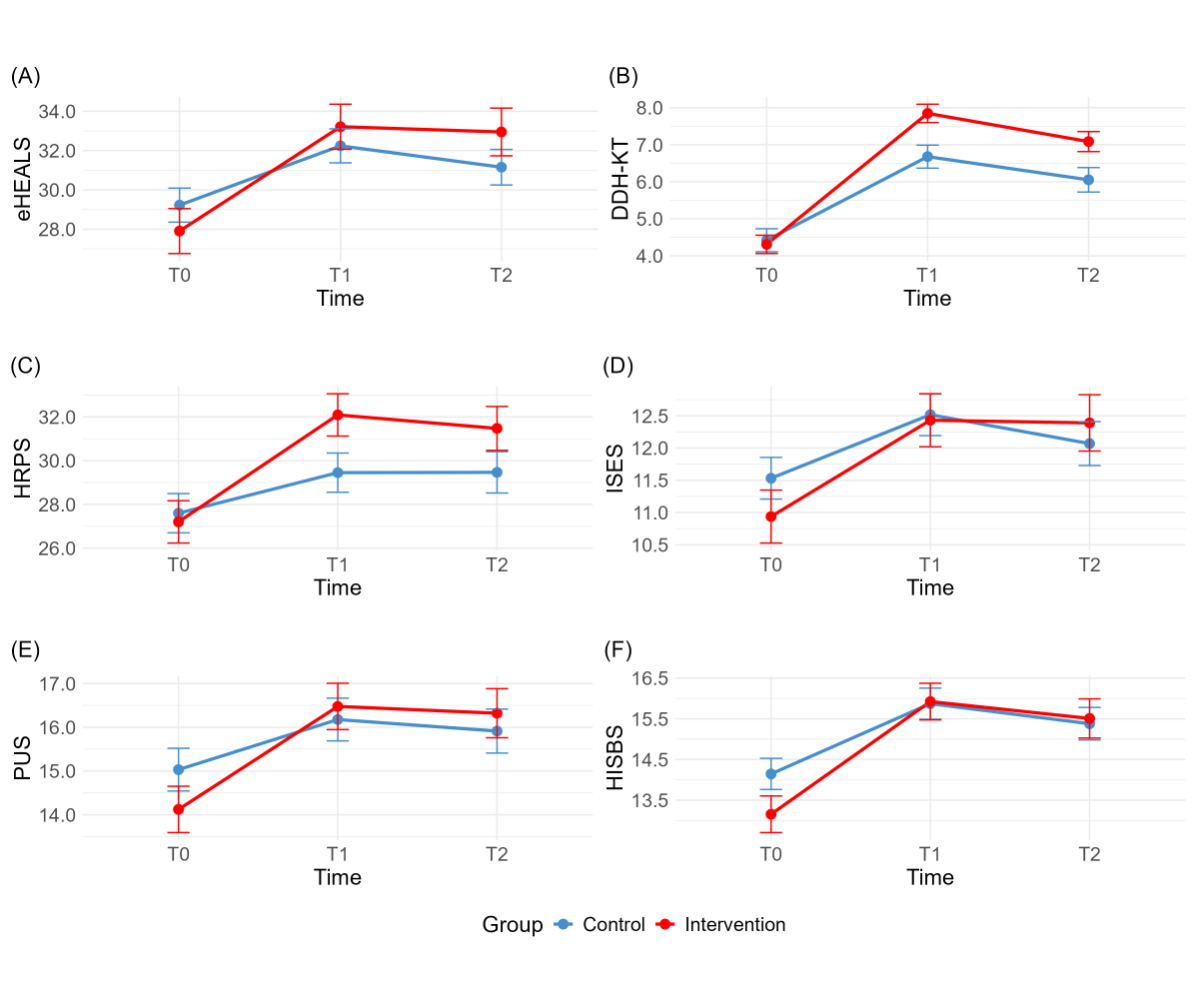
Changes in outcomes over time by groups. (A) eHEALS (primary outcome), (B) DDH-KT, (C) HRPS, (D) ISES, (E) PUS, and (F) HISBS (secondary outcomes). DDH-KT: developmental dysplasia of the hip knowledge test; eHEALS: eHealth Literacy Scale; HISBS: Health Information-Seeking Behavior Scale; HRPS: Health Risk Perception Scale; ISES: Information Self-Efficacy Scale; PUS: Perceived Usefulness Scale.

The group × time interaction in eHEALS was not significant (*P*=.26). The intervention group showed higher scores than the control group at T1 (33.62, 95% CI 32.76-34.49; *d*=0.20, 95% CI 0.13-0.56) and T2 (33.27, 95% CI 32.38-34.17; *d*=0.36, 95% CI 0.01-0.80), indicating sustained improvements following the LLM-generated learning intervention. Table 5 reports the means estimated from the model and the contrasts between groups across the specified time points; Figure 5 graphically illustrates the outcomes overtime by condition.

**Table 5:** Change in outcomes.

Time		Control EMM^a^ (95% CI)	Intervention EMM (95% CI)	Group difference			Cohen *d* (95% CI)		Group × time interaction, *P* value
				β (95% CI)	SE				
**eHEALS^b^**								.26	
	T0	29.23 (28.05 to 30.40)	27.91 (26.24 to 29.57)	—^c^	—	—		—	
	T1	31.89 (31.01 to 32.77)	33.62 (32.76 to 34.49)	1.73 (0.49 to 2.97)	0.63	0.20 (0.13 to 0.56)		—	
	T2	30.87 (29.97 to 31.78)	33.27 (32.38 to 34.17)	2.40 (1.13 to 3.67)	0.65	0.36 (0.01 to 0.80)		—	
**DDH-KT^d^**								.66	
	T0	4.42 (3.92 to 4.92)	4.31 (3.87 to 4.74)	—	—	—		—	
	T1	6.65 (6.26 to 7.05)	7.87 (7.48 to 8.25)	1.22 (0.67 to 1.77)	0.28	0.71 (0.33 to 1.11)		—	
	T2	6.02 (5.62 to 6.42)	7.12 (6.72 to 7.51)	1.10 (0.53 to 1.66)	0.29	0.54 (0.17 to 0.96)		—	
**HRPS^e^**								.25	
	T0	27.60 (26.22 to 28.97)	27.20 (25.71 to 28.69)	—	—	—		—	
	T1	29.39 (28.33 to 30.45)	32.23 (31.19 to 33.26)	2.84 (1.36 to 4.31)	0.75	0.50 (0.12 to 0.86)		—	
	T2	29.48 (28.40 to 30.56)	31.55 (30.49 to 32.61)	2.07 (0.56 to 3.59)	0.77	0.41 (0.05 to 0.79)		—	
**ISES^f^**								.25	
	T0	11.53 (10.99 to 12.08)	10.94 (10.30 to 11.58)	—	—	—		—	
	T1	12.38 (12.00 to 12.77)	12.59 (12.22 to 12.97)	0.21 (–0.33 to 0.75)	0.27	0.04 (0.30 to 0.39)		—	
	T2	11.96 (11.56 to 12.35)	12.51 (12.12 to 12.90)	0.55 (–0.00 to 1.11)	0.28	0.17 (0.19 to 0.55)		—	
**PUS^g^**								.48	
	T0	15.03 (14.27 to 15.80)	14.12 (13.29 to 14.96)	—	—	—		—	
	T1	15.93 (15.40 to 16.46)	16.70 (16.18 to 17.21)	0.77 (0.02 to 1.51)	0.38	0.11 (0.22 to 0.49)		—	
	T2	15.61 (15.06 to 16.15)	16.66 (16.12 to 17.20)	1.05 (0.28 to 1.82)	0.39	0.15 (0.19 to 0.51)		—	
**HISBS^h^**								.96	
	T0	14.15 (13.50 to 14.79)	13.15 (12.34 to 13.97)	—	—	—		—	
	T1	15.68 (15.10 to 16.27)	16.15 (15.57 to 16.72)	0.46 (–0.36 to 1.28)	0.42	0.02 (0.32 to 0.39)		—	
	T2	15.23 (14.63 to 15.83)	15.67 (15.08 to 16.26)	0.44 (–0.40 to 1.28)	0.43	0.05 (0.33 to 0.41)		—	

^a^EMM: estimated marginal mean.

^b^eHEALS: eHealth Literacy Scale.

^c^Not applicable.

^d^DDH-KT: developmental dysplasia of the hip knowledge test.

^e^HRPS: Health Risk Perception Scale.

^f^ISES: Information Self-Efficacy Scale.

^g^PUS: Perceived Usefulness Scale.

^h^HISBS: Health Information-Seeking Behavior Scale.

#### Secondary Outcomes

All secondary outcomes reported nonsignificant group × time interactions (*P*>.2), while the intervention group benefited from small to moderate impact sizes. DDH-KT scores were higher in the intervention group at T1 (7.87, 95% CI 7.48-8.25; *d*=0.71, 95% CI 0.33-1.11) and T2 (7.12, 95% CI 6.72-7.51; *d*=0.54, 95% CI 0.17-0.96). HRPS scores showed a similar pattern at T1 (32.23, 95% CI 31.19-33.26; *d*=0.50, 95% CI 0.12-0.86) and T2 (31.55, 95% CI 30.49-32.61; *d*=0.41, 95% CI 0.05-0.79). Additionally, PUS demonstrated consistent and statistically meaningful between-group differences favoring the intervention group at both T1 (16.70, 95% CI 16.18-17.21; *d*=0.11, 95% CI 0.22-0.49) and T2 (16.66, 95% CI 16.12-17.20; *d*=0.15, 95% CI 0.19-0.51). ISES and HISBS scores showed comparable positive trends; however, there were little differences across the groups.

## Discussion

### Principal Findings

This study evaluated the performance of 4 mainstream LLMs in education content and validated the effectiveness of LLM-generated caregiver education interventions. All 4 models demonstrated robust capabilities in generating content. ChatGPT-4 and DeepSeek-V3 outperformed Copilot and Gemini Flash in accuracy and fluency. The pilot trial suggests that LLM-assisted education may be associated with modest improvements in eHealth literacy (the primary outcome) and DDH knowledge compared with web-based searches; however, these findings should be interpreted as exploratory rather than confirmatory. These findings suggested that LLM-generated content was a feasible supplementary approach for health education. Its effectiveness appears to be enhanced when structured instruction and guided use are provided.

### LLMs Performance

Overall, ChatGPT-4 performed well across several dimensions. It excelled in producing content that was logically clear and linguistically fluent. ChatGPT-4 was widely suitable for tasks with moderate complexity. DeepSeek-V3 was ideal for generating complex health education content, especially for requiring depth and professionalism. Gemini 2.0 Flash excelled in fluency and readability but had minor deficits in richness and accuracy. Its concise content is suitable for quick-reference scenarios. Gemini 2.0 Flash was useful for quickly accessing information. However, it was limited in tasks requiring depth. Its design focuses on simplicity and efficiency, suitable for everyday consultations or simple questioning and answering, and other low-complexity tasks. Copilot performed weakly in several dimensions, with omissions in its generated content and slightly obscure language expressions. It was suitable for tasks that require lower content quality.

All 4 LLMs scored at or above the neutral threshold (≥3/5) for accuracy, richness, and fluency. PEMAT-P understandability ≥70% indicated that basic comprehension standards were met. However, their PEMAT-P actionability was limited. This limitation may reduce the utility of LLM-generated handouts for guiding caregiver decisions. Only Copilot provides source citations, which raises concerns about the traceability and reliability of the information. Although readability levels were close to the average reading level of US adults (eighth grade) [36], they still exceeded American Medical Association recommendations (no more than sixth grade) for health education materials [37]. Nevertheless, the current web-based health education materials for orthopedic specialties were less than this recommendation [38]. This gap suggests that the readability of content generated by LLMs within the prompt framework has improved, but needs to be further optimized for the health education materials [39].

Based on publicly available official documentation and technical reports, the observed performance differences among the evaluated LLMs may be attributed to variations in training data, architectural design, and optimization objectives. ChatGPT-4 is described as a transformer-based multimodal model trained on a mixture of public and licensed data and aligned through supervised fine-tuning and reinforcement learning from human feedback. DeepSeek-V3 uses a mixture-of-experts architecture and large-scale pretraining, which may favor long-form generation and information coverage, helping explain its more comprehensive outputs. Gemini 2.0 Flash emphasizes efficiency and interaction speed, suggesting an optimization trade-off that supports fluency and readability but may constrain depth under limited prompting. Copilot functions as a product-level system rather than a fixed foundation model, with outputs influenced by orchestration layers and underlying model routing that can vary over time. Overall, these findings indicate that suitability for caregiver-oriented health education depends on how training data, architecture, and optimization priorities align with specific educational goals, rather than on overall model capability alone.

### Evaluation Indicators

In practice, AI-assisted learning was associated with modest improvements in caregivers’ eHealth literacy and DDH knowledge compared with unguided web-based searches. This encouraged the educational value of using LLM-generated content. Short-term exposure did not significantly increase self-efficacy or active information-seeking behavior. This observation was consistent with behavioral science evidence. It emphasized that knowledge improvement was insufficient to drive behavioral change without supportive motivation, confidence, and environmental reinforcement. Lasting behavioral changes may require longer reinforcement, repeated exposure, environmental support, or clinician guidance. Although content generated by advanced models was more accurate and detailed, caregivers generally preferred concise, readable materials over lengthy or overly technical texts. This indicated that optimal education required balancing accuracy, conciseness, and clarity, rather than solely pursuing information richness.

### Comparison With Prior Work

Prior studies had mostly evaluated a single LLM using a limited set of metrics. For instance, ChatGPT-3.5’s responses to spinal surgery questions were assessed solely for accuracy and readability [40]. This study extended previous research by systematically comparing 4 mainstream LLMs under identical conditions. We included expert ratings (accuracy, richness, and fluency), standardized assessment instruments (Patient Education Materials Assessment Tool and DISCERN), readability metrics, and learning outcomes. By connecting content quality to user learning outcomes, our study provided a more comprehensive and clinically relevant assessment of LLMs for health education. Based on prior teaching improvements using Bloom’s taxonomy [41], it was used to improve the education by applying an organized method to content created by LLM. Prior studies showed that LLMs such as ChatGPT can enhance information accessibility, support communication and decision-making, and reduce anxiety levels [42]. These benefits have been demonstrated across diverse clinical contexts, including cancer care, orthopedic surgery, and mental health interventions [43-45]. The study reported that chatbot-enhanced prenatal education improved knowledge more effectively than standard mobile applications [46]. Our findings supported these findings by showing significant improvements in caregivers’ eHealth literacy and knowledge of DDH. We focused more on enhancing eHealth literacy than on specific disease knowledge. This competency was essential not only for acquiring medical knowledge but also for enabling users to properly browse and use AI solutions across varied health information demands. Given that AI systems offer more flexible, interactive, and context-adaptive support than internet search, higher levels of eHealth literacy are necessary to ensure their safe and optimal use.

LLMs were characterized by actual-time dialogue, instant feedback, and personalized communication. These features enhanced user engagement during health education processes, thereby improving knowledge acquisition [44]. Participants in the intervention group demonstrated significantly higher health-risk perception than those in the web-based group, showing that personalized AI-generated information increases perceived relevance and strengthens risk understanding. Additionally, the immediate responses and conversational interactivity of LLMs maintained user attention more effectively than static web-based information [47]. It resulted in increased satisfaction and maintained engagement.

Despite these advantages, some studies identified notable limitations in the accuracy and completeness of LLM outputs. McMahon and McMahon [48] warned that ChatGPT may generate misleading or unsafe recommendations in sensitive scenarios such as medication abortion. Ponzo et al [49] demonstrated that ChatGPT often produced incomplete or inconsistent dietary advice requiring professional revision. This pattern aligned with our heat-map analysis: LLMs performed the best in descriptive but worst in requiring clinical reasoning, procedural detail, or latest guideline recommendations, such as medication management, postoperative instructions, and emergency decision-making. These weaknesses appeared across multiple medical specialties and reflected broader constraints [50], including incomplete clinical training data, generating actionable guidance, and the universal LLMs’ inherent cautious tendency. Thus, caregivers using AI-assisted information retrieval still require oversight and guidance from health care professionals [51].

### Study Limitations

There are still some limitations to this study. First, although expert evaluation is an essential component of content quality assessment, it may carry the risk of subjective bias. Second, the evaluation was based on responses to a limited set of common DDH-related prompts. The variety and complexity of actual caregiver inquiries might not be adequately captured by such a limited selection of prompts. Third, each question was only created 3 times because of limitations on model use and study feasibility. Estimates of model variability would be more stable with more repetitions. Fourth, each LLM’s web-based interface characteristics were standardized. It may cause slight differences when compared to the normal interaction situations of actual users. Finally, because LLMs undergo frequent updates and iterative changes, the findings of this study reflect model performance during the specific access period and may not fully generalize to future versions.

### Practical Implications and Future Recommendations

The 2-stage results suggest that LLMs have potential as accessible, cost-effective, and personalized educational tools for caregivers, particularly in settings where traditional health education resources are limited. AI may supplement traditional clinician education by automating repetitive informational tasks, thereby alleviating health care professionals’ workload and allowing them to prioritize complex clinical cases. Enhancing knowledge and timely medical consultation are especially important for the early recognition of DDH. In rural and remote places with inadequate medical services, LLMs may help minimize geographic and economic obstacles to health education, increasing educational reach [52].

The perceived utility of AI-generated content is not solely determined by technical accuracy. Although ChatGPT-4 and DeepSeek-V3 generated high-quality content, users do not always prefer longer or more detailed responses. Caregivers, especially older adults, often prefer concise and clear information [53]. It suggests that instructional design should balance content quality with readability. Accordingly, when incorporating LLMs into clinical education, health educators may consider structured prompting and staged content generation. Instructional design might begin with simple explanations. As users express interest, gradually provide more specialized information with a guided summary.

However, the risks of misinformation, hallucinations, and unclear accountability cannot be ignored. LLM outputs exhibit inherent uncertainty; responses can vary across conversational contexts and may produce plausible but inaccurate statements regarding diagnostic thresholds or guideline-specific recommendations [54]. Furthermore, potential biases in training data may limit the cultural and contextual adaptability of these models [55]. As they may inadvertently reflect high-resource health care assumptions while overlooking local beliefs, language nuances, or service availability. Therefore, to ensure safe use, LLMs should be positioned strictly as auxiliary tools rather than substitutes for comprehensive medical assessments, physical examinations, and consultations with health care professionals [56]. In clinical practice, data confidentiality must be treated as a primary prerequisite. Patients provide informed consent for the use of LLM-assisted education, and workflows explicitly discourage the entry or disclosure of identifiable personal information [57]. Professional monitoring is crucial because LLM-generated content can be ambiguous, erroneous, or prejudiced. This includes regular evaluation of AI-generated educational outputs, bias-aware checks, and escalation procedures when high-risk issues emerge [58]. Future implementation strategies include retrieval-augmented generation, expert review mechanisms, and standardized safety and regulatory frameworks. With these safeguards, systematic incorporation of LLMs into health care procedures may support standardized health education and improve efficiency and scalability without compromising safety [59]. Future work should also identify the support resources required for safe adoption, including staff training, governance and auditing procedures, and technical infrastructure. Therefore, LLMs hold potential to support future health education and clinical communication.

### Implications for Practice

The implications for practice are that we (1) prefer models that cite reliable sources, (2) use prompts that request guideline-based advice, (3) always include disclaimers clarifying that LLMs cannot replace professional consultation, (4) target ≤6th-grade readability and simplify outputs with follow-up prompts, and (5) review and adapt content before sharing with patients.

### Conclusions

This study demonstrates that LLMs hold substantial potential for supporting education in DDH. ChatGPT-4 achieved 85% accuracy and 93% fluency, while DeepSeek-V3 led in 83% richness, generally outperforming the Copilot and Gemini 2.0 Flash. AI-assisted education was associated with small to moderate effect sizes for caregivers’ eHealth literacy, DDH knowledge, health risk perception, and perceived usefulness compared with web-based searches in this pilot trial. In addition, this study applied Bloom’s Taxonomy as a guiding pedagogical framework to structure the LLM-generated DDH educational content. This approach allowed the content to support the spectrum of caregiver learning needs, extending from foundational knowledge acquisition to decision-oriented guidance. Study limitations include potential expert subjectivity, a narrow prompt set with few generations, and controlled interface settings. LLMs are auxiliary tools and cannot replace the need for professionals. Future research should focus on optimizing plain language, refining dialogue design, and enhancing audience personalization to improve the quality of materials generated by LLMs.

## Appendix

Multimedia Appendix 1 Question bank and generated data.

Multimedia Appendix 2 Formulas for evaluating readability.

Multimedia Appendix 3 DDH knowledge test. DDH: developmental dysplasia of the hip.

Multimedia Appendix 4 Phase 1 scoring data.

Multimedia Appendix 5 CONSORT-eHEALTH checklist (V 1.6.1).

Multimedia Appendix 6 Phase 2 participants' scoring data.

## Abbreviations

AI: artificial intelligence
CONSORT: Consolidated Standards of Reporting Trials
CONSORT-EHEALTH: Consolidated Standards of Reporting Trials of Electronic and Mobile Health Applications and Online Telehealth
DDH: developmental dysplasia of the hip
DDH-KT: developmental dysplasia of the hip knowledge test
eHEALS: eHealth Literacy Scale
FKGL: Flesch-Kincaid Grade Level
FKRE: Flesch-Kincaid Reading Ease
HISBS: Health Information-Seeking Behavior Scale
HRPS: Health Risk Perception Scale
ICC: intraclass correlation coefficient
ISES: Information Self-Efficacy Scale
LLM: large language model
PEMAT-P: Patient Education Materials Assessment Tool for Printable Materials
PUS: Perceived Usefulness Scale
RCT: randomized controlled trial
SMOG: Simple Measure of Gobbledygook

## Data Availability

The full data supporting this study, including scoring data from phase 1 and outcomes from phase 2, can be accessed from the Multimedia Appendices.
