# Supplementary material for: Evaluating and Validating Large Language Models for Health Education on Developmental Dysplasia of the Hip: 2-Phase Study With Expert Ratings and a Pilot Randomized Controlled Trial
Source: J Med Internet Res. 2026 Jan 19;28:e73326. doi: 10.2196/73326 (PMC12865344; doi:10.2196/73326)
Supplement: Multimedia Appendix 1 [file jmir_v28i1e73326_app1.docx]

**Prompts**

Using developmental dysplasia of the hip (DDH) in children as an example, answer the following Using developmental dysplasia of the hip (DDH) in children as an example, answer the following questions in detail, ensuring the content is easily understandable for non-medical professionals. Life-like examples and situations can be incorporated to help readers better grasp the information. Please reduce the number of syllables to make the sentence simpler.

**Question bank**

| **Bloom's Taxonomy** | **Part** | **Content** |
| --- | --- | --- |
| **Remembering** | **Basics** | “What is [disease name]? Please explain its main features and potential effects in simple language.”  “Who is most likely to develop [disease name]? How common is it?”  “Will [disease name] have a long-term impact on my quality of life? What aspects should I pay special attention to?” |
|  | **Anatomy and Effects** | “What parts of the body does [disease name] mainly affect? What do these parts do in a healthy state?”  “What symptoms or consequences may arise if these parts are damaged? Can you provide specific examples?” |
| **Understanding** | **Etiology and Risk Factors** | “What are the main causes of [disease name]? What risk factors may increase the chances of developing it?”  “What lifestyle habits or environmental factors increase the risk of developing [disease name]? How can I prevent it?”  “If I have a family history, is my risk of developing [disease name] higher? What preventive measures should I take?” |
|  | **Symptoms and Early Recognition** | “What are the main symptoms of [disease name]? What signs indicate the disease is worsening? Should I seek immediate medical attention?” |
| **Applying** | **Physician Examination and Diagnosis** | “What methods do doctors usually use to confirm the diagnosis of [disease name]? What is the purpose of each test?”  “What indicators are most important in test results? How do these results reflect the severity of the disease?”  “After confirming the diagnosis of [disease name], do I need to schedule regular follow-ups? How often should I have them and what should be checked?” |
|  | **Emergencies** | “What symptoms indicate that I need to seek immediate medical attention?  “How should I handle and self-monitor during an emergency situation?” |
| **Analyzing** | **Hospital Treatment and Rehabilitation** | “What treatments will I receive during hospitalization? What is the purpose of each treatment?”  “How can I prevent infections or other complications during hospitalization? What can family members do to assist with recovery?”  “How can I reinforce the effects of treatment after discharge? What rehabilitation programs are recommended?” |
|  | **Medication Management** | “What are the common medications used to treat [disease name]? How should I take them correctly?”  “What should I do if I forget to take my medication or take it at the wrong time? Will it affect the treatment?”  “What foods should I avoid while taking medication for [disease name]? Are there common side effects?” |
|  | **Pain Management** | “What types of pain may be caused by [disease name] or its treatment? Where is the pain usually felt?”  “What methods can help relieve pain? What are the recommended pharmacological and non-pharmacological treatments?”  “What steps should I take if the pain is more than expected or worsens? Do I need to contact my doctor to adjust my treatment plan?” |
| **Evaluating** | **Post-operative Management** | “What post-surgery care precautions should I take?”  “How long after surgery can I return to normal daily activities?”  “How soon after surgery can I begin resuming activities?”  “What activities are recommended during the recovery period?”  “How do I schedule my exercise during the postoperative recovery period?”  “What are the stages during postoperative recovery? What precautions should I take in each stage?”  “Do I need a specialized rehabilitation plan? How does it help with recovery?”  “How can I prevent recurrence after surgery?”  “What complications can occur after surgery? What steps should I take?” |
| **Creating** | **Daily Living and Health Management** | “What foods are helpful in recovery from [disease name] or during recovery from surgery? What foods may aggravate the condition?”  “How should I adjust my diet to prevent constipation or other common problems?”  “What exercises can I do during the recovery phase? How often should I do them?”  “What precautions should I take when exercising, especially during postoperative recovery?”  “Does [disease name] affect the quality of my sleep? What are some ways to improve sleep and help the body recover?” |
|  | **Mental Health** | “What psychological problems may be associated with [disease name] or postoperative recovery, such as anxiety or depression? How can I deal with them positively?”  “What are the signs that I need psychological support? How can I find professional help or support resources?” |
|  | **Digital Tools and Management** | “Are there any recommended health apps or devices that can help manage [disease name] or postoperative recovery?”  “How can I use health tracking tools to monitor my condition or recovery progress?” |
| **Expanding*** | **Education and Support Resources** | “What are some ways to access reliable information about [disease name] or post-operative care?”  “Are there support groups or community resources that can help me or my family cope better?” |
|  | **Social and Economic Impact** | “What is the usual cost of treatment for [disease name]? Are there insurance or subsidy policies to reduce the financial burden?”  “What social assistance can I apply for if the disease or surgery causes financial hardship?” |
|  | **Future Planning and Visioning** | “How can I balance mental health, family responsibilities, and work in disease management? What are some practical suggestions?”  “How might [disease name] affect future quality of life? How can I plan ahead to improve my health?” |

**Reply from ChatGPT-4 (First time)**

Sure! Let’s break this down using **Developmental Dysplasia of the Hip (DDH)** in children as an example.

**1. What is Developmental Dysplasia of the Hip (DDH)?**

DDH is a condition where a baby’s hip joint doesn’t develop properly. In a normal hip, the ball of the thigh bone fits snugly into the hip socket. But in DDH, the ball might be loose, or the socket might be too shallow, which can lead to problems later on. This makes it harder for the hip joint to move smoothly.

Think of it like a ball that doesn’t fit well in a cup. If it's not fixed, it can cause pain and problems when the child grows older.

**2. Who is most likely to develop DDH? How common is it?**

DDH is more common in:

- **Girls**: They are about 4 times more likely to have DDH than boys.
- **First-born babies**: If a child is the first born, the chances are higher.
- **Babies born breech** (feet first instead of head first): This position can make it harder for the hip to form correctly.
- **Family history**: If a parent or sibling had DDH, the baby might be at higher risk.

It’s not super common, but it happens in about **1 in 1,000 babies**. However, mild cases are often harder to spot.

**3. Will DDH have a long-term impact on my child’s life? What should I pay attention to?**

If DDH is not treated, it can cause problems as the child grows, like:

- **Pain** in the hip or groin, especially when walking or running.
- **Difficulty walking** or limping.
- **Arthritis** in the hip later in life, because the joint may wear out more quickly.

But the good news is, if DDH is found early, it can usually be treated with **a simple harness** or sometimes surgery. In these cases, the child can grow up without any long-term problems.

**What to pay attention to:**

- **Early check-ups**: Pediatricians often check for DDH right after birth and during regular check-ups.
- **Signs of hip problems**: If you notice your baby has one leg shorter than the other, or if they don't seem to move their legs equally, mention it to the doctor.

If caught early, most kids grow up with no lasting effects!

Sure! Let’s talk about **developmental dysplasia of the hip (DDH)** in children.

**What parts of the body does DDH mainly affect? What do these parts do in a healthy state?**

DDH affects the **hip joint**, which is where the **thigh bone (femur)** meets the **hip socket** in the pelvis.

- **Hip joint**: This is like a ball-and-socket joint. The **ball** is the top of the thigh bone, and the **socket** is a part of the pelvis. In a healthy hip, the ball fits perfectly into the socket, allowing the leg to move smoothly in many directions, like walking, running, and jumping.
- The joint is also supported by **ligaments**, which are strong bands of tissue that hold the bones in place and help control movement.

**What symptoms or consequences may arise if these parts are damaged? Can you provide specific examples?**

If a child has DDH, the hip joint doesn’t form properly. The ball might not fit into the socket the way it should, which can cause problems like:

1. **Pain**: If the ball and socket don’t fit right, the joint can be uncomfortable or even painful, especially when walking or moving.
   - For example, a child might complain of pain in the hip, groin, or even the knee.
2. **Limited movement**: The hip might not move smoothly. This can make it hard for a child to run or jump like other kids.
   - Imagine trying to move your arm, but your shoulder joint doesn’t work right—this is similar to how the hip might feel in DDH.
3. **Limping or walking differently**: If the joint doesn’t fit properly, a child might start limping or walking in a way that puts extra stress on other parts of the body.
   - This can happen because the body is trying to adjust to the pain or discomfort in the hip.
4. **Long-term problems**: If DDH isn't treated, it can lead to arthritis or other issues later in life, because the hip joint is not working properly over time.
   - As the child grows, their hip might wear out faster than it should, which could cause chronic pain or difficulty moving when they get older.

In short, DDH can cause pain, movement issues, and long-term problems with the hip joint if it's not treated early.

Sure! Let's break it down step by step using **developmental dysplasia of the hip (DDH)** in children as an example.

**1. What are the main causes of DDH? What risk factors may increase the chances of developing it?**

**DDH** happens when a baby’s hip joint doesn’t form properly. In a healthy hip, the ball (the top of the thigh bone) fits into the socket (the part of the hip bone that holds it). In DDH, this joint is too loose or doesn’t fit together correctly.

**Main causes:**

- **Genetics**: If a parent or sibling has DDH, the baby is more likely to have it too. This is because it can run in families.
- **Position in the womb**: Babies who are in a breech position (bottom or feet first) during pregnancy are at a higher risk.
- **Gender**: Girls are more likely to have DDH than boys. This may be because the female hormone relaxin, which loosens ligaments during pregnancy, can affect the joint.

**Risk factors:**

- Being a firstborn (there’s less space in the womb).
- Tight swaddling (wrapping babies too tightly) that restricts their leg movement.
- Having a family history of DDH.

**2. What lifestyle habits or environmental factors increase the risk of developing DDH? How can I prevent it?**

- **Lifestyle/Environmental factors:**
  - **Tight swaddling**: If a baby’s legs are wrapped too tightly and can’t move, it might affect hip development.
  - **Position during birth**: Breech births or a baby being born in an abnormal position increases the risk.
- **Prevention**:
  - **Gentle swaddling**: If you choose to swaddle your baby, make sure their legs can move freely. Their legs should be able to bend up and out at the hips.
  - **Early screening**: If DDH is caught early (usually with a physical exam after birth), it can be treated right away, preventing more serious problems later.
  - **Proper positioning**: Encourage your baby to have their legs in a relaxed position, not overly straight or tightly bound.

**3. If I have a family history, is my risk of developing DDH higher? What preventive measures should I take?**

Yes, **family history** matters! If someone in your family (like a parent or sibling) has had DDH, your baby might be at a higher risk. It doesn’t guarantee your baby will have it, but it does increase the chances.

**What can you do?**

- **Talk to your doctor**: If there’s a family history of DDH, your doctor might monitor your baby more closely after birth. This could mean extra check-ups or tests.
- **Screening**: In many countries, babies are screened for DDH as part of routine newborn care. Early detection is key for quick treatment.

**Preventive measures**:

- Ensure proper positioning during pregnancy, and after birth, let your baby’s legs move freely as much as possible.
- If your baby is found to have DDH, treatments like special harnesses or braces can help straighten the hip joint.

In short:

- **DDH** is when a baby’s hip joint doesn’t develop properly, often due to genetics, position in the womb, or certain environmental factors.
- To reduce the risk, avoid tight swaddling, encourage leg movement, and keep up with regular check-ups for early detection. If there’s a family history, monitoring is important to catch it early.

Sure! Let's use **Developmental Dysplasia of the Hip (DDH)** as an example.

**What are the main symptoms of DDH in children?**

DDH is when a baby's hip joint doesn't develop properly. The hip joint is where the thigh bone (femur) fits into the hip socket (acetabulum). If the socket is too shallow or the bone isn't in place, it can cause problems.

Some common signs of DDH in babies and young children are:

1. **Uneven leg length**: One leg might appear shorter than the other.
2. **Uneven skin folds**: If you look at the folds of skin on the thighs, they might not line up evenly.
3. **Limited leg movement**: The baby might not be able to move their legs as much as usual, or one leg might seem less flexible than the other.
4. **Clicking or popping sound**: When you move the baby’s legs, you might hear a clicking sound in the hip joint.

**What signs indicate the disease is worsening?**

If DDH isn’t treated, it can get worse and cause more serious problems later on. Some signs that the condition is getting worse might include:

1. **Walking difficulties**: As the child gets older, they might have trouble walking, or their walk might seem abnormal (like limping).
2. **Pain in the hip or groin**: The child might complain about pain or discomfort in their hip area.
3. **Leg length difference**: The difference in leg lengths might become more noticeable, especially when the child is walking or standing.

**Should I seek immediate medical attention?**

If you notice any of the symptoms above, it’s a good idea to talk to your doctor. DDH is often caught during routine check-ups with a pediatrician, especially in newborns. But if you notice:

- Your baby seems to have trouble moving their legs.
- You hear a clicking sound or see uneven skin folds.
- As your child grows, they start limping or complaining of hip pain.

You should definitely schedule a visit with the doctor. Early treatment can help prevent serious problems later on.

**Example to help you understand:**

Imagine a jigsaw puzzle where the pieces don’t quite fit together. At first, the puzzle might still work, but over time, if the pieces don’t come together right, the whole picture could get messed up. That’s like the hip joint in DDH. If it isn’t fixed early, it could cause more problems later.

Does that make sense? Would you like to know more about how DDH is treated?

Sure! Let's talk about **Developmental Dysplasia of the Hip (DDH)** in children.

**1. How do doctors confirm the diagnosis of DDH? What is the purpose of each test?**

To find out if a child has DDH, doctors usually do the following:

- **Physical exam**: The doctor will feel the baby’s hips and move their legs to see if they make a clicking sound or feel loose. This helps doctors check if the hip is in the right place or if it’s dislocated.
- **Ultrasound**: This test uses sound waves to take pictures of the baby's hip joints. It’s often done for babies under 6 months old to see how the hip joint is developing.
- **X-ray**: This is used for older children (around 6 months and older). It shows if the hip joint is in the right position or if it has shifted.

**2. What indicators are most important in test results? How do these results reflect the severity of the disease?**

- **In physical exams**: The doctor might look for signs like a “clicking” or “clunking” sound when moving the legs. If the hip joint feels unstable, this can show that the hip isn’t where it should be.
- **In ultrasound and X-rays**: They will check if the ball of the hip (the femoral head) is properly placed in the socket. A big gap between the ball and socket means that the hip is dislocated or not fully formed. If the ball doesn’t fit into the socket well, it means the child has DDH, and the more misaligned it is, the more severe the condition is.

**3. After confirming the diagnosis, do I need regular follow-ups? How often should I have them and what should be checked?**

Yes, regular check-ups are important to make sure the hip is developing correctly after the diagnosis. The frequency of follow-ups depends on the child’s age and the severity of the condition:

- **For babies (under 6 months)**: The doctor might check every few weeks or months to see how the hip is developing with treatment (like a special harness).
- **For older children**: Follow-ups may happen every few months to make sure the hip is staying in the right place, especially if surgery was needed.

At each check-up, the doctor will look at how the hip joint is doing and may use tests like ultrasounds or X-rays to make sure the treatment is working.

In short:

- **Physical exams** check for clicking or looseness.
- **Ultrasounds and X-rays** show how the hip joint is positioned.
- Regular follow-ups help ensure the hip develops correctly over time.

If you have a child with DDH, regular visits to the doctor help catch any problems early and guide the right treatment.

**Symptoms that Indicate Immediate Medical Attention:**

Developmental dysplasia of the hip (DDH) in children can sometimes cause symptoms that need quick medical attention. Look for these signs:

1. **Leg or Hip Pain**: If your child has hip pain or seems to be limping and it’s not improving, you should take them to a doctor right away. Pain is a sign that something might be wrong.
2. **Limited Movement in the Hip**: If your child has trouble moving their leg or if their leg looks like it's positioned awkwardly (for example, turned in or out), this could be a sign of DDH.
3. **Uneven Legs**: If one leg seems shorter than the other, or you notice the knees are at different heights when your child is lying down, this could also be a symptom.
4. **Swelling or Redness**: If there is any swelling or redness around the hip area, it might indicate infection or another issue that needs attention.
5. **Difficulty Walking or Crawling**: If a baby or toddler who should be crawling or walking isn’t moving properly or seems to avoid putting weight on one leg, it could be DDH.

**How to Handle and Self-Monitor in an Emergency:**

If you think your child might have DDH and you notice one of these signs, follow these steps:

1. **Stay Calm**: It’s important to remain calm, as panicking won’t help. Trust your instincts, and if you feel something is wrong, it’s always better to get it checked out.
2. **Avoid Moving the Leg Too Much**: If your child is in pain or has trouble moving the leg, don’t force any movement. Keep them comfortable until you can get medical help.
3. **Keep the Area Elevated**: If there’s swelling, try to elevate the leg gently, but don’t worry too much about the position if they are in too much discomfort.
4. **Contact Your Pediatrician**: Call your child’s doctor or visit an urgent care center for advice. If the pain is severe or the leg is visibly injured, don’t wait. Go to the ER.
5. **Monitor for Changes**: While waiting for medical help, keep an eye on how your child is feeling. Are they more fussy than usual? Do they seem to get worse, or are they calmer? This can help doctors understand what’s going on.

In summary, if you see signs like pain, difficulty moving, or swelling in your child’s hip area, seek medical attention as soon as possible. Don’t try to fix it yourself, but keep your child comfortable and calm until professionals can take over.

Sure! Let's break it down:

**1. What treatments will I receive during hospitalization? What is the purpose of each treatment?**

When a child is in the hospital for **Developmental Dysplasia of the Hip (DDH)**, doctors will focus on helping the hip joint develop properly. Here’s what might happen:

- **Pavlik harness**: This is a special soft device that holds the baby's hips in a position that helps the hip joint stay in the socket. It's like wearing a baby “seatbelt” that keeps their hips in place while they grow.
- **Surgery**: If the hip doesn’t stay in place with the harness, the doctor might need to do surgery to reposition the hip and put it back in place. This usually happens when the baby is older.
- **Physical therapy**: After the hip is in the correct position, therapy might help strengthen the muscles around the hip and improve the child’s movement.

**2. How can I prevent infections or other complications during hospitalization? What can family members do to assist with recovery?**

To prevent infections or other problems, doctors will be very careful, but family members can also help:

- **Keep the area clean**: If your child has surgery, it’s important to keep the surgery area clean and dry. Family members can help by making sure the bandages stay clean and following the nurse’s instructions.
- **Watch for signs of infection**: Redness, swelling, or fever can be signs of an infection. If you notice these, tell the doctor right away.
- **Encourage comfort**: Help your child feel comfortable by bringing things from home that they love, like a favorite toy or blanket. It can make the hospital feel a little less scary.
- **Follow the doctor’s instructions**: Whether it’s about medications or how to use the harness, it’s important to follow all instructions carefully. This helps speed up recovery and prevent issues.

**3. How can I reinforce the effects of treatment after discharge? What rehabilitation programs are recommended?**

After your child leaves the hospital, it’s important to continue supporting their recovery:

- **Continue using the harness**: If your child was wearing a harness, you might need to keep using it at home for a while. Follow the doctor’s guidance on how long to use it.
- **Follow-up appointments**: Your child will need regular check-ups to make sure the hip is developing properly. These visits will help catch any problems early.
- **Physical therapy**: Even after going home, your child might need physical therapy. This helps strengthen the muscles around the hip, so the joint stays in place and the child can move well.
- **Gradual activities**: Slowly introducing activities like crawling, walking, or playing helps your child’s muscles and bones get stronger over time. Ask your doctor about which activities are safe as your child grows.

By staying on top of these treatments and follow-ups, your child will have the best chance at a full recovery!

**Developmental Dysplasia of the Hip (DDH) in Children**

DDH is a condition where the hip joint doesn't form correctly in babies or young children. This can lead to problems like the hip dislocating or not being able to move properly. It is usually treated with a mix of methods, including medications, devices, and sometimes surgery. Let’s break down your questions based on this condition.

**1. What are the common medications used to treat DDH? How should I take them correctly?**

In DDH, medication isn't always the main treatment. However, if a child is in pain or has inflammation, doctors may recommend **pain relievers** like:

- **Acetaminophen (Tylenol)**: For pain relief.
- **Ibuprofen (Advil, Motrin)**: For both pain and inflammation.

**How to take them correctly:**

- Always follow the doctor’s advice about **how much** to give and **how often** to give it.
- For example, if your doctor says to give acetaminophen every 6 hours, don’t give it more often.
- These medications can be taken with food or water to avoid stomach upset.

**2. What should I do if I forget to take my medication or take it at the wrong time? Will it affect the treatment?**

If you forget to give your child a dose of pain medication or if it's taken too late, don't worry too much, but here's what to do:

- **If you remember soon** after missing a dose, give it as soon as possible.
- **If it's almost time for the next dose**, just skip the missed one and take the next dose as scheduled. Don’t double the dose.

Missing a dose of medication occasionally isn’t usually a big problem, but make sure to keep a regular schedule to help your child feel better.

**3. What foods should I avoid while taking medication for DDH? Are there common side effects?**

When using common pain medications like acetaminophen or ibuprofen, you don’t need to avoid specific foods. However, here are a few things to keep in mind:

- **For ibuprofen**: It can upset the stomach, so it's best to give it with food to protect the stomach lining.
- **For acetaminophen**: Be careful with foods or drinks that have alcohol, as combining alcohol with this medication can be harmful to the liver.

**Common side effects** of these medications include:

- Upset stomach.
- Drowsiness or feeling tired (especially with higher doses).
- Rarely, allergic reactions like rash or swelling.

If you notice anything unusual, like your child feeling worse or having a rash, contact the doctor.

In summary:

- Medications for DDH are often used to manage pain or inflammation.
- If a dose is missed, take it when you remember, but don’t double doses.
- No need to avoid specific foods, but always follow doctor guidelines, especially when it comes to the timing and amount of medication.

Let me know if you need more details!

**1. What types of pain may be caused by Developmental Dysplasia of the Hip (DDH) or its treatment? Where is the pain usually felt?**

DDH happens when a child's hip joint doesn’t form properly. This can cause pain because the hip joint doesn’t move smoothly, leading to discomfort in the hip area. The pain can also affect the thigh or groin area. Some children might feel pain when they try to move their leg or when they are in certain positions, like sitting or standing.

If a child is being treated for DDH, especially if they have a cast, brace, or surgery, they may also feel some pain or discomfort. After treatment, they might experience soreness from the adjustments or from the position their hip is kept in for healing.

**2. What methods can help relieve pain? What are the recommended pharmacological and non-pharmacological treatments?**

There are several ways to manage pain caused by DDH:

- **Pharmacological treatments (medications)**:
  - **Pain relievers** like acetaminophen (Tylenol) or ibuprofen (Advil) can help with mild to moderate pain.
  - If the pain is more severe, a doctor may prescribe stronger medication or even a muscle relaxant.
- **Non-pharmacological treatments (without medication)**:
  - **Rest and gentle movement**: Sometimes, simply resting the hip and avoiding too much movement helps reduce pain.
  - **Physical therapy**: Exercises can help strengthen the muscles around the hip, making it easier to move and less painful.
  - **Warm or cold packs**: A warm compress or cold pack can ease soreness or swelling.
  - **Bracing**: In some cases, a special brace or harness is used to help the hip joint develop properly. This may cause some discomfort, but over time it can improve the condition and reduce pain.

**3. What steps should I take if the pain is more than expected or worsens? Do I need to contact my doctor to adjust my treatment plan?**

If the pain becomes worse than expected, or if your child seems to be in a lot of pain even after taking medicine or using other pain-relief methods, it's important to take action. Here’s what you can do:

- **Contact your doctor**: Let them know how much pain your child is in and if it doesn’t get better or gets worse over time.
- **Look for signs of something more serious**: If your child’s hip seems swollen, hot, or there’s a sudden change in how they move their leg, these could be signs of an infection or a problem with the treatment.
- **Adjust the treatment plan**: Your doctor might change the medications or suggest other treatments, like a different brace or more physical therapy, to help reduce pain and make the healing process go more smoothly.

It’s always better to check with the doctor if you’re worried, as they can provide the best guidance for your child’s specific situation.

Sure! Let’s break it down using developmental dysplasia of the hip (DDH) in children as an example. DDH is when a child’s hip joint doesn’t develop normally, which can cause the hip to be unstable or dislocated. Sometimes surgery is needed to fix it.

**1. Post-surgery care precautions:**

- **Keep the hip stable:** After surgery, you may need to use a special brace or cast to keep the hip in the right position. It’s important to follow your doctor’s instructions on how to wear it.
- **Limit movement:** Avoid bending or twisting the hip too much in the first few weeks. For example, don’t let the child sit cross-legged or squat.
- **Watch for signs of infection:** Keep the surgery site clean, and look out for redness, swelling, or pus.

**2. How long after surgery can I return to normal daily activities?**

- It usually takes **several weeks to months** for full recovery. At first, limit activities like running or jumping. Your doctor will tell you when it’s safe to return to things like school, playing, or sports.

**3. How soon can I begin resuming activities?**

- **Gentle activities** like walking or light stretching can begin after a few weeks, depending on how the surgery site heals. The child will likely need to avoid more active play until they’ve had time to heal properly.

**4. What activities are recommended during recovery?**

- **Gentle exercises** such as walking or swimming (once the doctor says it’s okay) can help strengthen the hip without putting too much strain on it. **Physical therapy** will help with moving the hip in a safe way.
- **Avoid high-impact activities** like running or jumping in the first few months.

**5. How do I schedule my exercise during the recovery period?**

- Follow your doctor’s and physical therapist’s advice. Usually, physical therapy starts a few weeks after surgery. Start with light exercises and gradually increase intensity. Be sure to take breaks and avoid overdoing it.

**6. What are the stages during postoperative recovery? What precautions should I take in each stage?**

- **Stage 1: Immediately after surgery (1-2 weeks)**
  - Rest and keep the hip protected with a brace or cast.
  - Avoid putting weight on the hip.
  - Be cautious of infection by keeping the area clean.
- **Stage 2: Early recovery (2-6 weeks)**
  - Begin gentle movements, like walking with support.
  - Keep activities low-impact.
  - Avoid high-stress movements like running or squatting.
- **Stage 3: Mid-recovery (6-12 weeks)**
  - Gradually return to more normal activities.
  - Continue physical therapy for strength and flexibility.
- **Stage 4: Full recovery (3-6 months)**
  - Return to sports or more active play as guided by your doctor.
  - Keep monitoring the hip for signs of discomfort.

**7. Do I need a specialized rehabilitation plan? How does it help with recovery?**

- Yes, a **rehabilitation plan** (usually physical therapy) is important. It helps the child regain strength, flexibility, and movement in the hip. The therapist will teach exercises that are safe and specific to the recovery stage.

**8. How can I prevent recurrence after surgery?**

- **Follow-up appointments:** Make sure the child has regular check-ups with the doctor to catch any problems early.
- **Maintain strength:** Continue with physical therapy and exercises to keep the muscles around the hip strong and supportive.
- **Be careful with activities:** Avoid risky activities like rough play or heavy lifting, which could stress the hip.

**9. What complications can occur after surgery? What steps should I take?**

- **Infection:** Keep the wound clean, and watch for signs like redness, swelling, or pus. Contact your doctor if you see these.
- **Blood clots:** Movement is important to prevent blood clots. Keep up with any prescribed exercises and walking.
- **Hip dislocation:** If the hip shifts out of place, you may notice pain or difficulty moving. If this happens, call your doctor right away.

Overall, the key to recovery is following your doctor’s and physical therapist’s advice, taking things slow, and being patient. Healing takes time, but with care and the right approach, the child will be able to return to normal activities safely.

Sure! Let's talk about **Developmental Dysplasia of the Hip (DDH)** in children and recovery after surgery, with simple answers to your questions.

**1. What foods are helpful in recovery from DDH or during surgery? What foods may aggravate the condition?**

When a child is recovering from surgery for DDH, it's important to eat foods that support healing and overall health.

**Helpful foods:**

- **Protein-rich foods** (like chicken, fish, beans, eggs) help the body heal tissues and muscles.
- **Calcium-rich foods** (like milk, cheese, yogurt) are good for bones, which is important when recovering from surgery on the hip.
- **Vitamin C** (found in fruits like oranges, strawberries, or vegetables like broccoli) helps with tissue repair.
- **Whole grains** (like oats, brown rice, and whole wheat) help keep energy up during recovery.

**Foods to avoid:**

- **Junk food** (like chips or sweets) can slow down recovery and might not give the body the nutrients it needs to heal.
- **Too much salt** might cause swelling, which is something to avoid after surgery.

**2. How should I adjust my diet to prevent constipation or other common problems?**

After surgery, constipation can be a common issue. To avoid it, here’s what you can do:

- **Eat high-fiber foods** (like fruits, vegetables, whole grains). Fiber helps the digestive system work better and prevents constipation.
- **Drink plenty of water**—this keeps the body hydrated and helps the intestines stay healthy.
- **Prune juice or high-fiber snacks** (like apples, pears, or beans) can also help if constipation is a problem.

**3. What exercises can I do during the recovery phase? How often should I do them?**

During recovery from DDH surgery, gentle exercises are important to help improve strength and mobility.

- **Gentle leg movements**: Moving the legs slowly to increase range of motion can help. For example, moving the legs up and down while lying on the back.
- **Physical therapy exercises**: These may include simple stretching or strengthening exercises suggested by a physical therapist. They help rebuild strength and flexibility in the hip.
- **Walking**: Depending on how your child feels, walking (with assistance or support) is a good low-impact exercise to help regain strength.

These exercises should start slowly and increase as the child heals. Typically, a doctor or physical therapist will guide how often to do them, but starting with 2-3 times a day, for short sessions, is a good start.

**4. What precautions should I take when exercising, especially during postoperative recovery?**

Here are some tips to stay safe when exercising during recovery:

- **Avoid high-impact activities** like running or jumping until the doctor says it’s okay. These can strain the hip and slow healing.
- **Don't push through pain**. If a child feels pain while doing an exercise, it’s important to stop and rest.
- **Use supportive gear** (like crutches or a brace) if needed, as advised by a doctor.
- **Follow a physical therapist's advice**—they’ll create a plan to guide you step-by-step.

**5. Does DDH affect the quality of my sleep? What are some ways to improve sleep and help the body recover?**

Yes, DDH can sometimes cause pain, especially when a child is trying to get comfortable or sleep after surgery. Here's how you can improve sleep during recovery:

- **Comfortable position**: Make sure the child’s hips are supported in a position that doesn't cause pain. Pillows can be used to prop up legs or support the body.
- **Pain management**: If there's pain, make sure the child takes any prescribed pain medicine (like acetaminophen or ibuprofen) before bed, as recommended by the doctor.
- **Keep a calm environment**: Quiet, dark rooms and a regular bedtime routine can help the child relax and sleep better.
- **Gentle stretching**: Light stretches before bed, as recommended by a doctor or therapist, can help relax the muscles around the hip.

By following these guidelines, it can make a big difference in helping a child recover and feel better after surgery for DDH.

**Psychological Problems in Children with DDH (Developmental Dysplasia of the Hip) or Post-Op Recovery:**

When a child has DDH, it means their hip joint hasn’t developed properly, which can cause pain or make it hard for them to walk. If the child needs surgery or a long recovery, they may experience **anxiety** or **depression** because of physical discomfort, being in a cast, or missing out on activities like playing with friends.

- **Anxiety**: The child may feel worried or scared about doctors, hospitals, or even the recovery process. They might have trouble sleeping or seem more easily upset.
- **Depression**: Feeling sad or withdrawn is also possible, especially if the child is stuck at home and can’t do the things they usually enjoy, like sports or playing outside.

**How to Deal Positively:**

1. **Stay Calm and Reassuring**: Children look to their parents for comfort. Showing that you are calm and confident helps them feel safe and less anxious.
2. **Create a Routine**: Even with limited mobility, try to keep things familiar. Having a daily routine can bring comfort and control.
3. **Encourage Open Communication**: Let your child express their feelings. It’s okay if they feel scared or sad. Talking about those feelings helps them cope.
4. **Involve Them in Fun Activities**: While they might not be able to run or jump, you can find activities they enjoy—like reading together, drawing, or doing puzzles.
5. **Stay Positive**: Remind your child that the surgery or treatment is helping them get better, and soon they’ll be able to play again.

**Signs You Might Need Psychological Support:**

If you're noticing your child or even yourself feeling overwhelmed, it might be time to look for extra help. Signs that someone might need psychological support include:

1. **Extreme Worrying or Fear**: If the child is constantly anxious, crying, or seems scared about everything related to their hip or recovery, this could be a sign that they need professional support.
2. **Withdrawal**: If they’re avoiding social situations, isolating themselves, or losing interest in things they used to enjoy, it might be a sign of depression.
3. **Changes in Behavior**: If the child is acting out more than usual, or showing signs of frustration or anger that can’t be explained, this might be a sign of stress.
4. **Trouble Sleeping**: If they’re having trouble falling asleep or waking up often because of pain or fear, it’s a red flag.

**How to Find Help:**

1. **Talk to Your Doctor**: If you’re concerned, start with a pediatrician or the surgeon. They can guide you to child-friendly therapists or counselors who specialize in helping kids with health challenges.
2. **Look for a Child Psychologist**: These professionals know how to work with children to help them manage their feelings during tough times like recovery.
3. **Support Groups**: There are online or in-person groups for parents dealing with DDH or similar conditions. These can be great for sharing advice and emotional support.
4. **Use Mental Health Hotlines**: In many places, there are free helplines you can call to talk to someone about what you're going through.

The key is to recognize that it’s okay to seek help when emotions are tough. Getting the right support can make a huge difference for both the child and their family.

**Developmental Dysplasia of the Hip (DDH) in children** is a condition where a baby’s hip joint doesn’t form properly, which can make it unstable. This means the ball of the hip joint doesn’t fit properly into the socket, which can lead to problems as the child grows.

**Health Apps or Devices for Managing DDH or Postoperative Recovery:**

For DDH or after surgery, there are a few tools and apps that can help both parents and doctors keep track of the child’s recovery.

1. **Baby and Child Health Apps**: There are apps designed to track your child's growth, milestones, and health progress. Some of these apps can help you track things like your baby’s physical therapy sessions, any changes in their movements, or their symptoms after treatment.
   - Example: **Baby Connect** allows you to log daily activities like feedings, diaper changes, and any signs you might notice related to their hip development or post-surgery recovery. This can be helpful for parents to track how well their child is recovering and share this information with doctors.
2. **Post-Surgery Monitoring Apps**: If your child had surgery to treat DDH, some apps can help you track things like pain levels, mobility, and any issues with the hip as it heals.
   - Example: **My Surgery** helps parents track recovery steps after surgery. It may remind you when it's time for follow-up doctor visits, checkups, or physical therapy exercises.
3. **Wearable Devices**: There are devices that monitor movement and help track the baby’s hip development after surgery.
   - Example: Some **wearable devices** can be used to monitor movement patterns and alert you if the child’s hip is not healing properly. These devices are often used by doctors to keep an eye on your child's progress.

**Using Health Tracking Tools for DDH or Recovery Progress:**

1. **Track Symptoms and Changes**: If your child is in a brace or cast after surgery for DDH, you can use health apps to track things like how often you need to change the brace, any discomfort your child might be having, or if the brace is helping to keep the hip in place.
2. **Set Reminders for Exercises or Checkups**: After surgery, the child might need physical therapy exercises. Some health apps allow you to set reminders for when it's time to do certain stretches or exercises that will help the hip joint heal properly.
3. **Log Pain and Mobility**: Some apps allow you to log pain levels (on a scale of 1 to 10) and mobility changes, like if the child is having trouble moving their legs or walking. This can help doctors know how well the recovery is going.
4. **Track Growth and Development**: Since DDH happens in babies and young children, it's important to monitor their growth. Some apps help track things like how the child’s walking and movement are developing over time. This information can be helpful for doctors to spot any problems early.

In short, health tracking apps and devices can help you manage the recovery of a child with DDH by providing reminders, logging important information, and keeping track of progress. These tools make it easier to see if your child is healing well and when to take action if something is wrong.

**Developmental Dysplasia of the Hip (DDH) in children** is a condition where a baby’s hip joint doesn’t form properly, which can make it unstable. This means the ball of the hip joint doesn’t fit properly into the socket, which can lead to problems as the child grows.

**Health Apps or Devices for Managing DDH or Postoperative Recovery:**

For DDH or after surgery, there are a few tools and apps that can help both parents and doctors keep track of the child’s recovery.

1. **Baby and Child Health Apps**: There are apps designed to track your child's growth, milestones, and health progress. Some of these apps can help you track things like your baby’s physical therapy sessions, any changes in their movements, or their symptoms after treatment.
   - Example: **Baby Connect** allows you to log daily activities like feedings, diaper changes, and any signs you might notice related to their hip development or post-surgery recovery. This can be helpful for parents to track how well their child is recovering and share this information with doctors.
2. **Post-Surgery Monitoring Apps**: If your child had surgery to treat DDH, some apps can help you track things like pain levels, mobility, and any issues with the hip as it heals.
   - Example: **My Surgery** helps parents track recovery steps after surgery. It may remind you when it's time for follow-up doctor visits, checkups, or physical therapy exercises.
3. **Wearable Devices**: There are devices that monitor movement and help track the baby’s hip development after surgery.
   - Example: Some **wearable devices** can be used to monitor movement patterns and alert you if the child’s hip is not healing properly. These devices are often used by doctors to keep an eye on your child's progress.

**Using Health Tracking Tools for DDH or Recovery Progress:**

1. **Track Symptoms and Changes**: If your child is in a brace or cast after surgery for DDH, you can use health apps to track things like how often you need to change the brace, any discomfort your child might be having, or if the brace is helping to keep the hip in place.
2. **Set Reminders for Exercises or Checkups**: After surgery, the child might need physical therapy exercises. Some health apps allow you to set reminders for when it's time to do certain stretches or exercises that will help the hip joint heal properly.
3. **Log Pain and Mobility**: Some apps allow you to log pain levels (on a scale of 1 to 10) and mobility changes, like if the child is having trouble moving their legs or walking. This can help doctors know how well the recovery is going.
4. **Track Growth and Development**: Since DDH happens in babies and young children, it's important to monitor their growth. Some apps help track things like how the child’s walking and movement are developing over time. This information can be helpful for doctors to spot any problems early.

In short, health tracking apps and devices can help you manage the recovery of a child with DDH by providing reminders, logging important information, and keeping track of progress. These tools make it easier to see if your child is healing well and when to take action if something is wrong.

**Ways to Access Reliable Information about Developmental Dysplasia of the Hip (DDH) or Post-Operative Care**

1. **Doctor or Specialist**: A doctor, like a pediatrician or an orthopedic specialist, is the best place to start for reliable information. They can explain DDH and the steps for treatment or recovery. They also provide advice for post-surgery care.
2. **Hospitals and Clinics**: Hospitals often have pamphlets or websites with helpful information about DDH and recovery. You can trust these sources because they’re written by medical professionals.
3. **Medical Websites**: Websites like the American Academy of Orthopaedic Surgeons (AAOS) or the Mayo Clinic offer clear and accurate information. Just make sure the website ends in “.org” or “.gov,” as these are often trustworthy.
4. **Books**: There are books written by experts on child health and DDH. Ask your doctor for recommendations on reliable books.
5. **Peer-Reviewed Journals**: If you're looking for more detailed information, medical journals (like those available through Google Scholar) provide in-depth studies. However, these might be harder to understand without a medical background.

**Support Groups or Community Resources for Families**

1. **Online Support Groups**: Websites like Facebook have groups where families of children with DDH can connect. You can ask questions, share stories, and get advice from others who are going through similar experiences.
2. **Non-Profit Organizations**: Groups like the International Hip Dysplasia Institute provide resources, support, and information. They may also have local meetups or online communities.
3. **Parent Networks**: Some hospitals or clinics have networks where parents of children with DDH can talk to each other. These networks provide emotional support and share tips for managing life with DDH.
4. **Local Support Groups**: Some areas have local groups for parents of kids with DDH. You can ask your doctor or search online for one near you.
5. **Counseling or Therapy**: If the stress of dealing with DDH feels overwhelming, counseling might help. Talking to a professional can provide ways to cope and manage your feelings.

These resources can help you and your family better understand DDH and find support during the recovery process.

**Cost of Treatment for Developmental Dysplasia of the Hip (DDH) in Children**

The treatment for DDH in children can range from non-surgical methods like wearing a special brace or harness to surgery if needed. The cost depends on how severe the case is and what treatments are required.

- **Non-surgical treatment**: This could be around $1,000–$3,000 for a harness or brace, depending on your location and healthcare system. Follow-up appointments and X-rays might add more to the cost.
- **Surgery**: If surgery is required, the cost can be much higher. For example, hip surgery could cost $10,000–$50,000 or more, depending on the type of surgery, hospital, and country.

**Insurance and Subsidy Policies**

Many countries have insurance policies that can help reduce the cost.

- **Private Insurance**: If you have private health insurance, it may cover part or all of the treatment, depending on your plan. You should check with your insurance provider to see what is covered under DDH treatment.
- **Public Insurance or Subsidies**: In countries with public healthcare, like the UK or Canada, the cost of treating DDH might be covered by the government. However, waiting times may be longer, and you may need to check specific eligibility requirements.

Some hospitals or clinics also offer sliding scale fees, meaning the cost can be adjusted based on your income.

**Social Assistance for Financial Hardship**

If DDH treatment causes financial strain, there are different types of assistance you can apply for, depending on where you live.

- **Health Assistance Programs**: In many countries, there are government programs that help with medical costs, especially for children. These might cover part of the treatment or provide grants to families with low income.
- **Charity Organizations**: Some non-profits and charity organizations provide financial help or assistance for families dealing with medical conditions like DDH. You can reach out to groups like the **March of Dimes** or local support organizations.
- **Disability Benefits**: If DDH leads to long-term problems or disabilities, you may be able to apply for disability benefits for your child. This can help with the cost of care and special needs.

**Real-life Example**

Imagine a family with a young child diagnosed with DDH. The child needs to wear a special brace for a few months. The family’s private insurance might cover part of the cost, but there’s still a bit left to pay. If they’re struggling to cover this, they could apply for help through their country’s health assistance program or a charity that helps families with medical costs. These programs could make a huge difference, covering some or all of the remaining expenses.

By checking with your doctor, insurance, or local community services, you can find out which options are best for you to manage the financial burden of DDH treatment.

**How can I balance mental health, family responsibilities, and work in disease management (using DDH as an example)?**

Managing **Developmental Dysplasia of the Hip (DDH)** in a child can feel overwhelming, especially when you have to balance mental health, family duties, and work. Here are some simple suggestions:

1. **Ask for help**: It’s important to remember that you don’t have to do everything alone. Ask family members, friends, or coworkers to help with tasks. For example, if your child with DDH needs frequent doctor visits, you can ask a relative to help with childcare or grocery shopping.
2. **Set small, realistic goals**: Break down big tasks into small ones. For instance, instead of stressing over all the medical appointments, focus on one at a time. Don’t worry about everything at once.
3. **Create a routine**: DDH treatment can involve a lot of appointments and care. Try to set a schedule that works for your family and job. This helps to keep things predictable and reduces stress.
4. **Take care of your own mental health**: Take time for yourself, even if it’s just for a few minutes each day. Doing something you enjoy—like reading or taking a walk—can help recharge your mental health. When you feel better, you’ll be able to manage things better.
5. **Talk to your employer**: Many jobs offer flexibility. Let your employer know about your situation. You may be able to adjust your hours or work from home on certain days to help balance care responsibilities.

**How might DDH affect future quality of life, and how can I plan ahead to improve my health?**

DDH in a child, if untreated, could lead to problems in the future, like:

- **Chronic pain**: Over time, untreated DDH may cause pain in the hip joints.
- **Mobility issues**: The child might find it harder to walk or play as they grow older.
- **Arthritis**: In some cases, it can lead to arthritis in the hips when the child gets older.

To improve the future quality of life and health:

1. **Early diagnosis and treatment**: The sooner DDH is found, the better. Treatments, like wearing a special harness or even surgery, can help prevent problems later on. Always follow the doctor’s advice.
2. **Physical therapy**: After treatment, physical therapy can help strengthen muscles around the hip, improving mobility and reducing pain.
3. **Regular check-ups**: Keep up with medical appointments, even if the child seems fine. This ensures the condition doesn’t worsen over time.
4. **Healthy lifestyle**: Encourage your child to stay active in a way that supports healthy joints. This could mean swimming or low-impact activities.

Planning ahead means taking these steps now to avoid complications later. By staying on top of the condition and supporting your child’s health, you can help them live an active and pain-free life in the future.

**Reply from ChatGPT-4 (Second time)**

Sure! Let's break this down using **Developmental Dysplasia of the Hip (DDH)** in children.

**What is DDH?**

DDH is a condition where a child's hip joint doesn’t form properly. Normally, the ball (top of the thigh bone) fits tightly into the socket in the hip bone. But in DDH, the ball and socket don't line up as they should. This can cause problems with movement and pain.

**Main Features of DDH:**

- **Hip instability**: The ball can slip out of the socket easily.
- **Limited movement**: The hip may not move smoothly.
- **Pain**: Over time, if untreated, it can cause hip pain.
- **Uneven leg length**: One leg might appear shorter than the other.

**Who is most likely to develop DDH? How common is it?**

DDH is more common in:

- **Newborns and babies**: It happens early in life, sometimes even before birth.
- **Girls**: It’s more common in girls than boys.
- **First-born children**: Babies born first in the family may have a higher chance.
- **Family history**: If someone in the family had DDH, there’s a higher chance of it happening.

About 1 in every 1,000 to 2,000 babies will have DDH that needs treatment, but it’s much more common in mild cases that may not need treatment.

**Will DDH have a long-term impact on my quality of life? What aspects should I pay special attention to?**

If DDH is treated early, most children lead normal, active lives. However, if it’s not treated, it can cause long-term problems.

**Long-term impact:**

- **Pain**: As a child grows, untreated DDH can lead to hip pain, especially as they get older.
- **Arthritis**: Over time, the hip joint might wear out, leading to arthritis (joint pain and stiffness).
- **Difficulty walking**: In severe cases, it can affect walking or running.

**What to pay attention to:**

- **Regular check-ups**: If your baby has DDH, regular doctor visits can catch it early.
- **Treatment**: Early treatment (like braces or surgery) can fix the problem and avoid long-term pain.
- **Development milestones**: Watch for signs like trouble walking or favoring one leg.

With early diagnosis and treatment, children with DDH can usually live a normal life without major problems!

**Developmental Dysplasia of the Hip (DDH) in Children**

**What parts of the body does DDH mainly affect?**

DDH mainly affects the **hip joint**. The hip is where the top of the thigh bone (called the femur) fits into a cup-shaped part of the pelvis called the **acetabulum**. This joint is like a ball and socket, allowing your legs to move in many directions.

In a healthy hip:

- The ball (top of the thigh bone) fits perfectly into the socket (the acetabulum), and they move smoothly together.
- This allows us to walk, run, jump, and do all kinds of movements easily.

**What symptoms or consequences may arise if these parts are damaged?**

If DDH happens, it means that the hip joint hasn’t developed properly. The ball might be loose or not fit into the socket as it should.

Symptoms and consequences of this can include:

1. **Hip pain:**
   - The joint may not work smoothly, causing pain, especially when walking or running.
   - In young children, they might have trouble walking or appear to limp.
2. **Limited movement:**
   - If the ball doesn't fit into the socket properly, the child might find it hard to move their leg in certain directions.
   - For example, they might struggle to sit cross-legged or may not be able to move their leg out to the side as easily.
3. **Uneven leg length:**
   - One leg might be shorter than the other because the hip joint isn't growing the way it should. This can lead to difficulty walking or running and may cause a noticeable limp.
4. **Arthritis in the future:**
   - If the hip joint isn’t properly treated, it can wear down over time, leading to **arthritis** (pain and stiffness) when the child grows up.

**Real-life example:** Imagine a child who loves to play soccer. If they have DDH, they may start feeling pain in their hip after running or kicking the ball. They might even notice that their leg feels stiff or harder to move in certain directions. Over time, they might not be able to run or play the way they used to.

In severe cases, if the joint is left untreated, it can cause long-term problems, like **arthritis** as the child grows into an adult, making it harder for them to stay active.

Does that make sense?

**What are the main causes of Developmental Dysplasia of the Hip (DDH)? What risk factors may increase the chances of developing it?**

DDH happens when a baby’s hip joint doesn’t form properly. The ball (femoral head) and socket (acetabulum) in the hip joint don’t fit together the way they should, which can cause the joint to become loose or dislocated.

**Main causes**:

- **Genetics**: Sometimes, DDH runs in families, so it could be passed down from parents to children.
- **Position in the womb**: If a baby is in a breech position (feet or bottom first instead of head first) during pregnancy, they may have a higher chance of getting DDH.
- **First-born babies**: It’s more common in first-born children because the mother's womb may be more tight or less flexible.

**Risk factors**:

- **Family history**: If your family has a history of DDH, your baby is more likely to have it.
- **Gender**: Girls are more likely to have DDH than boys.
- **Breech birth**: Babies who are born feet-first or bottom-first have a higher risk.
- **Tight swaddling**: Wrapping a baby too tightly around the hips can increase the chance of DDH.

**What lifestyle habits or environmental factors increase the risk of developing DDH? How can I prevent it?**

Certain factors can make DDH more likely, and some lifestyle habits or environmental choices might play a role.

**Lifestyle and environmental factors**:

- **Swaddling too tightly**: Wrapping a baby in a way that limits movement of their legs can increase the risk of hip problems.
- **Not moving enough**: Babies who don’t have enough room to move their legs may have a higher chance of developing DDH. For example, keeping babies in restrictive positions for long periods might be harmful.

**Prevention**:

- **Allow your baby to move freely**: Let your baby stretch and move their legs as much as possible. This helps their hip joints develop properly.
- **Gentle swaddling**: If you do swaddle, make sure the baby’s hips can move freely and aren’t too tightly wrapped.
- **Early detection**: If DDH is found early, it’s easier to treat. Regular checkups with a pediatrician can help catch it early.

**If I have a family history, is my risk of developing DDH higher? What preventive measures should I take?**

Yes, if someone in your family has had DDH, your baby may be at a higher risk of getting it too.

**Higher risk**:

- Family history is one of the most significant risk factors. If a parent or sibling had DDH, your baby is more likely to have it.
- **Other factors**: If your baby is a girl or born in a breech position, the risk might go up even more.

**Preventive measures**:

- **Talk to your doctor**: If DDH runs in your family, let your pediatrician know. They can monitor your baby’s hips closely during checkups.
- **Follow guidelines for swaddling**: Make sure the baby’s hips can move freely when wrapped. Also, use slings or carriers that allow the legs to spread out instead of being restricted.
- **Get regular checkups**: Early detection of DDH can help treat it quickly and prevent problems later in life.

I hope that makes things clearer! Feel free to ask if you want any more details.

Sure! Let's break it down:

**What are the main symptoms of Developmental Dysplasia of the Hip (DDH) in children?**

DDH is a condition where a baby’s hip joint doesn’t develop properly. In simple terms, the ball part of the hip joint doesn’t fit well into the socket. The main symptoms are:

- **Uneven leg length:** One leg may seem shorter than the other.
- **Hip click or pop:** When the baby’s hip is moved, you might hear a clicking sound.
- **Asymmetry in skin folds:** If you look at the baby’s thighs, the skin folds might not line up symmetrically.
- **Limping or waddling:** As the child gets older and starts walking, they may limp or walk in a way that seems strange.

**What signs indicate the disease is worsening?**

If DDH isn’t treated, it can get worse. Signs of worsening DDH might include:

- **Pain in the hip or groin:** The child may start complaining about pain while walking or even while sitting.
- **Limited movement:** The child might have trouble moving the hip or leg in certain directions.
- **Limping gets worse:** As they get older, the limping may become more noticeable.
- **Stiffness in the hip:** The hip joint may feel stiff, making walking more difficult.

**Should I seek immediate medical attention?**

Yes, if you notice any of these signs, it’s important to see a doctor. Early treatment is key to helping the child’s hip joint develop properly. If your baby or child shows:

- A **clicking** sound in the hip that doesn’t go away,
- Uneven skin folds or legs,
- Difficulty moving their leg or seems to be in pain,

You should definitely talk to a doctor. The sooner DDH is treated, the better the outcome for the child.

Think of it like this: imagine a ball and socket (like a ball in a cup). If the ball doesn’t fit well in the cup, it might get stuck, hurt, or not move right. Treating DDH early helps make sure the ball and socket fit properly as the child grows!

Let’s break down these questions using **developmental dysplasia of the hip (DDH)** in children as an example. DDH is a condition where a child’s hip joint doesn’t develop properly. The ball part of the joint (the femoral head) doesn’t fit well into the socket (the acetabulum), which can lead to problems like dislocation or instability.

**1. How do doctors confirm the diagnosis of DDH? What is the purpose of each test?**

To confirm if a child has DDH, doctors usually use a combination of these tests:

- **Physical Exam**: The doctor checks the baby’s hips by gently moving the legs and looking for any signs of instability. They might feel or hear a "click" when moving the hip, which could indicate that the joint is not stable. This is often done soon after birth.
  - **Purpose**: This helps doctors see if there’s any obvious hip problem right away.
- **Ultrasound**: For babies under 6 months, doctors often use an ultrasound to look at the hip joint. It’s a safe test that uses sound waves to create images of the joint and check how the ball fits into the socket.
  - **Purpose**: It helps doctors see how well the hip joint is developing and if there’s any dislocation or instability. This is especially useful because babies’ bones are soft and not visible on X-rays.
- **X-ray**: After the baby is about 6 months old, doctors may take an X-ray. By then, the bones are hard enough to be seen clearly.
  - **Purpose**: X-rays can show how much the hip joint is misaligned or if it’s dislocated, giving doctors a clearer picture of how serious the problem is.

**2. What indicators are most important in test results? How do these results reflect the severity of DDH?**

The test results help doctors understand how severe the DDH is. Here are some key things they look for:

- **Hip stability**: Whether the hip joint moves out of its proper position (dislocates or subluxates). If the joint is very loose, this might be a sign of more severe DDH.
- **Development of the socket (acetabulum)**: How well the hip socket has formed around the ball of the joint. If the socket is shallow or not forming properly, it can indicate that DDH is more severe.
- **Femoral head position**: The position of the ball part of the hip joint (femoral head). If it’s out of place or doesn’t fit into the socket, the condition might be more severe.
- **Degree of dislocation**: How far the ball is out of the socket. If the ball is completely out (a dislocation), this is a more serious form of DDH.

**3. Do I need regular follow-ups after DDH is diagnosed? How often should I have them and what should be checked?**

Yes, after DDH is diagnosed, regular follow-ups are usually needed to make sure the hip is developing correctly and that the treatment is working. The frequency and what doctors check depend on the child’s age and how severe the DDH is.

- **Newborn to 6 months**: Follow-up visits might be scheduled every few weeks or months. The doctor will check if the hip is stable and if the joint is developing well.
- **6 months to 1 year**: If the hip joint is still developing, the doctor might check every few months to see if the treatment (like a harness or brace) is helping. At this stage, X-rays might be taken to see how the bones are growing.
- **After 1 year**: If treatment is needed, follow-ups might be spaced out to every 6 months or so. The doctor will check if the hip is stable and properly aligned.

The main goal during these visits is to make sure the joint is forming properly and the child’s hips are stable, helping prevent future problems like arthritis or walking difficulties.

**In summary:**

- Doctors confirm DDH with physical exams, ultrasounds (for babies under 6 months), and X-rays (for older children).
- Key indicators are hip stability, how well the socket is formed, and the position of the femoral head.
- Regular follow-ups are important to make sure treatment is working, especially during the first few years, and they happen at different intervals depending on the child’s age and condition.

I hope that makes things clearer! Let me know if you need more details on any part.

**Symptoms that need immediate medical attention:**

If a child has Developmental Dysplasia of the Hip (DDH), you might notice things like:

1. **Legs appear uneven** – One leg may seem shorter or look turned out differently than the other.
2. **Limited movement** – If the baby or child is not able to move their legs as easily or seems to have trouble bending them.
3. **Pain or discomfort** – Your child might cry or seem in pain when you move their legs, especially during diaper changes or when you try to position them for sleep.
4. **Clicking sound** – A clicking noise or feeling when you move the baby’s leg might be a sign.
5. **Limping** – If your child is old enough to walk, you might notice them limping or favoring one leg over the other.

**What to do in an emergency situation:**

If you notice any of these symptoms, it’s important to act quickly.

1. **Call your doctor** – If you see signs like pain, limited movement, or any of the above, call your child’s doctor or go to the emergency room to get help. DDH is easier to treat when caught early.
2. **Avoid moving the leg too much** – Don’t try to force or move the child’s leg in ways that seem uncomfortable or painful. It's better to wait for medical professionals to assess the situation.
3. **Stay calm and comfort your child** – In the moment, try to stay calm. Hold your baby or child and comfort them while you wait for medical help.

Remember, DDH can often be treated successfully if caught early, so it’s important to get medical attention as soon as you notice any worrying signs.

Let's break down the questions one by one using *developmental dysplasia of the hip (DDH)* in children as an example.

**1. What treatments will I receive during hospitalization? What is the purpose of each treatment?**

When a child is hospitalized for DDH, the treatment usually focuses on correcting the hip joint. Here’s what might happen:

- **Pavlik harness**: This is a special type of brace worn to hold the baby’s hips in a position that helps the hip joint develop correctly. It keeps the hips flexed and the knees apart, which helps the hip joint move into the socket.
- **Surgery**: If the harness doesn't work, surgery may be needed to reposition the hip joint. This is typically done if the hip joint is very dislocated.
- **Plaster cast**: After surgery, a cast may be applied to keep the hip in the right position while it heals.
- **Pain management**: Medication will be given to help the child feel comfortable during the treatment. The purpose is to control pain and help the child rest while they recover.

**2. How can I prevent infections or other complications during hospitalization? What can family members do to assist with recovery?**

Preventing infections and complications is key to a smooth recovery. Here’s how:

- **Proper hygiene**: Keeping the skin around the harness or cast clean and dry helps prevent skin infections. Family members can help by making sure the harness or cast doesn’t get too wet or dirty.
- **Monitoring the hip joint**: It’s important to check the fit of the harness or cast regularly. If it’s too tight, it can affect blood flow or cause discomfort. Family members can help by making sure it’s in the right position and that the baby is comfortable.
- **Encouraging movement (if possible)**: If the child is old enough to move around, gentle exercises or positioning can help keep the muscles and joints from getting stiff. Family members can assist by gently moving the child’s legs, as instructed by the doctor or physical therapist.
- **Medications and follow-up**: Family members should help make sure that medications are given on time and that the child attends any follow-up appointments to check on progress and avoid complications.

**3. How can I reinforce the effects of treatment after discharge? What rehabilitation programs are recommended?**

Once the child is discharged, it’s important to continue supporting the treatment at home.

- **Continued use of the Pavlik harness**: If the harness was used, it may need to be worn for a few months even after leaving the hospital. Parents should follow the doctor’s advice on when to use it and for how long.
- **Physical therapy**: A physical therapist may suggest exercises to strengthen the muscles around the hip and improve movement. These exercises help the child develop better control of the joint and improve long-term mobility.
- **Regular check-ups**: Follow-up appointments with the doctor are important to monitor how the hip is developing. The doctor will check if the joint is in the correct position and if treatment needs adjustment.

In summary, the treatments during hospitalization aim to fix the hip joint, prevent complications like infections, and manage pain. After discharge, family members can continue supporting the recovery process by using the harness, doing physical therapy, and going to check-ups. This all helps the child’s hip joint develop normally and avoid future problems.

Let's use developmental dysplasia of the hip (DDH) in children as an example to answer these questions:

**1. What are the common medications used to treat DDH? How should I take them correctly?**

For DDH in children, most treatments focus on helping the hip joint develop properly. Medications are not always the main treatment, but some might be used to help with pain or inflammation if needed. Here's what may be used:

- **Pain relievers**: Sometimes doctors give medications like acetaminophen (Tylenol) or ibuprofen (Advil) to help reduce pain or swelling if a child is uncomfortable.
- **Antibiotics**: In rare cases, if the child has an infection related to surgery, doctors may prescribe antibiotics.

To take these medications correctly:

- Follow the doctor’s instructions about how much to give and how often.
- Make sure to measure the medicine using the right tool, like a measuring spoon or syringe, if it's liquid.
- Don't skip doses, and don't give more than the prescribed amount.

**2. What should I do if I forget to take my medication or take it at the wrong time? Will it affect the treatment?**

If you forget to give the medication to your child or give it at the wrong time:

- **If it's close to the next dose**: Just wait and give the next dose as planned.
- **If it's still far from the next dose**: Give the missed dose as soon as you remember, but don’t double the dose to make up for it.

Missing a dose once or twice may not cause serious harm, but it's important not to make a habit of it. It might slow down the healing or make the pain last longer, so try to stick to the schedule as closely as possible.

**3. What foods should I avoid while taking medication for DDH? Are there common side effects?**

For DDH treatment, there aren’t specific foods that you need to avoid, especially if you’re using over-the-counter pain relievers. However, if your child is on any antibiotics:

- **Antibiotics**: Some antibiotics might interact with certain foods, like dairy or calcium-rich foods, which could make the medicine less effective. Your doctor will tell you if this is the case.

**Common side effects** of medications:

- **Pain relievers** (like ibuprofen or acetaminophen) can sometimes cause stomach upset or make the child feel drowsy.
- **Antibiotics** can cause upset stomachs or diarrhea. If your child has a side effect, talk to the doctor. They can adjust the treatment if needed.

In short, it’s about giving the right medicine at the right time and watching for any signs of side effects. Always ask your doctor if you’re unsure about any details!

**Pain caused by Developmental Dysplasia of the Hip (DDH) in children:**

DDH is a condition where a child’s hip joint is not formed properly. This can cause pain, but it doesn’t always happen right away. In younger babies, it might not hurt, but as they grow and start walking, the pain can begin.

**Where is the pain usually felt?**

- **Hips and groin**: The most common place for pain is in the hip area or the groin (the area where the thigh meets the body).
- **Thigh or knee**: Sometimes, pain can also be felt in the thigh or knee because the hip joint affects how the entire leg moves.

**Pain caused by treatment:**

- If a baby or child is treated with a special harness or cast (to keep the hip in place while it heals), there might be some discomfort from wearing the device. This is usually not very painful, but it could cause some skin irritation or mild soreness.

**Ways to relieve pain:**

**Pharmacological treatments (medications):**

- **Pain relief medicines** like acetaminophen (Tylenol) or ibuprofen (Advil) can help reduce pain and inflammation. These are usually recommended by doctors for children with DDH, especially if the child feels discomfort while moving or after treatment like a cast.
- In more severe cases, a doctor might give stronger pain relief medicines, but this is not very common for DDH.

**Non-pharmacological treatments (things you can do without medicine):**

- **Warm or cold packs**: A warm compress or ice pack (covered in a cloth) on the hip can help relieve discomfort. It’s important to make sure that the ice or heat isn’t too hot or cold to avoid burning or damaging the skin.
- **Gentle stretching**: If allowed by the doctor, gentle stretches or physical therapy can help make the hip more comfortable. This helps strengthen muscles around the hip and improves mobility.

**What to do if the pain gets worse or is unexpected:**

If your child’s pain becomes worse than expected, or if the pain is new and stronger, it’s important to reach out to your doctor. Here’s why:

- **Pain during treatment**: If the pain doesn’t go away or gets worse, it could mean that the treatment isn’t working as well as it should, or the hip may have moved out of place again.
- **Signs of infection**: Sometimes, a cast or harness can cause skin problems or infections, which may make the area painful.

**Steps to take:**

1. **Contact your doctor**: Don’t wait too long. A doctor can check if the hip is healing properly and adjust the treatment if needed.
2. **Follow-up appointments**: Make sure to keep any scheduled follow-up visits. Your doctor will check how the hip is doing and might recommend extra steps like changing the harness or cast.

In short, if the pain gets worse or doesn't improve with the usual treatments, it’s always a good idea to contact your doctor. They may need to make some changes to the treatment plan.

Sure! Let’s break everything down in simple terms using developmental dysplasia of the hip (DDH) in children as an example:

**1. What post-surgery care precautions should I take?**

After surgery for DDH, you need to be careful about not putting too much pressure on the hip. You should:

- Keep the child’s hip in the position the doctor recommends (usually with a brace or cast).
- Avoid any activities that involve too much movement, like running or jumping.
- Follow the doctor’s instructions about how to move the child’s legs to avoid strain on the healing hip.

**2. How long after surgery can I return to normal daily activities?**

It varies by child, but in general:

- You might need to wait 6-12 weeks before the child can start normal activities.
- Some children might need more time, depending on how well they heal and how their hip responds to the surgery.

**3. How soon after surgery can I begin resuming activities?**

Children usually start with gentle activities after a few weeks (around 4-6 weeks):

- Simple movements like sitting up, playing with toys, or crawling.
- The doctor will guide you on when to start more active play.

**4. What activities are recommended during the recovery period?**

- **Gentle play:** Keep the child active, but avoid high-impact activities. Things like playing with blocks or toys are great.
- **Physical therapy:** The doctor might recommend exercises to keep the muscles around the hip strong.
- **Walking with assistance:** Gradually, the child might start walking with a special brace or crutches to protect the hip.

**5. How do I schedule my exercise during the postoperative recovery period?**

- Start with small, easy exercises like gentle stretching or walking (with help) after a few weeks.
- Work up to more physical activities slowly, based on the doctor’s advice.
- Stick to a schedule recommended by the doctor or physical therapist.

**6. What are the stages during postoperative recovery? What precautions should I take in each stage?**

Recovery usually has 3 stages:

- **Early stage (1-4 weeks):** Focus on resting and protecting the hip. Avoid bending the leg too much or putting weight on it.
- **Middle stage (4-8 weeks):** Gradually introduce light movement and some standing, but avoid high-impact activities.
- **Late stage (8+ weeks):** More active play and walking, but still no running or jumping until cleared by the doctor.

**7. Do I need a specialized rehabilitation plan? How does it help with recovery?**

Yes, a rehabilitation (rehab) plan helps a lot. It usually involves:

- **Physical therapy:** Strengthening muscles around the hip and improving flexibility.
- **Gradual progress:** It helps avoid overdoing it, so the hip doesn’t get injured again.
- **Customized exercises:** The plan will be tailored to your child’s needs, helping them recover at the right pace.

**8. How can I prevent recurrence after surgery?**

To prevent the hip problem from coming back:

- Follow the doctor’s advice about movement limits and exercises.
- Avoid activities that put too much stress on the hip (like rough sports) until the doctor says it’s okay.
- Keep up with physical therapy to maintain strength and flexibility in the hip.

**9. What complications can occur after surgery? What steps should I take?**

Possible complications can include:

- **Infection:** Make sure to keep the wound clean. Watch for signs like redness or swelling.
- **Hip dislocation:** If the hip moves out of place, you’ll need to get medical help quickly.
- **Blood clots or swelling:** Keep the child’s legs elevated and watch for swelling. If it’s severe, contact the doctor.
- If you notice anything unusual, like a lot of pain, swelling, or redness, always call the doctor.

Hope that helps! If you need more details on any of these, just let me know.

Sure! Let's break down each question using **developmental dysplasia of the hip (DDH)** in children as an example. DDH is a condition where a child’s hip joint doesn’t form properly, which can lead to instability or dislocation. Here’s how the questions apply:

**1. What foods are helpful in recovery from DDH or during recovery from surgery? What foods may aggravate the condition?**

- **Helpful foods:**
  - **Calcium-rich foods**: Like milk, cheese, yogurt, leafy greens (like spinach), and fortified plant-based milks. Calcium helps build strong bones, which is important for hip recovery.
  - **Vitamin D**: Found in fatty fish (like salmon), egg yolks, and fortified foods. Vitamin D helps the body absorb calcium.
  - **Protein**: Foods like chicken, beans, eggs, and nuts. Protein helps repair muscles and tissues after surgery.
- **Foods to avoid or limit:**
  - **Processed foods**: Like chips, fast food, or sugary snacks. These can make inflammation worse and slow down recovery.
  - **High salt foods**: These can lead to fluid retention and swelling, which isn’t good during recovery.

**2. How should I adjust my diet to prevent constipation or other common problems?**

- **Fiber-rich foods**: Eating foods like whole grains (brown rice, oatmeal), fruits (apples, berries), and vegetables (broccoli, carrots) helps keep things moving in the digestive system.
- **Drink plenty of water**: Staying hydrated is key to preventing constipation.
- **Limit foods that may cause bloating**: Foods like beans or carbonated drinks may cause discomfort, so keep an eye on how your child’s body reacts to them.

**3. What exercises can I do during the recovery phase? How often should I do them?**

- **Gentle range-of-motion exercises**: These help move the hip joint gently and maintain flexibility. For example, simple leg lifts or bending the knees while lying down can help.
- **Physical therapy**: After surgery, the doctor may recommend specific exercises or stretches to strengthen muscles around the hip joint and improve movement.
- **Frequency**: Start slowly and follow the doctor’s instructions. Exercises might be done a few times a day, with rest periods in between.

**4. What precautions should I take when exercising, especially during postoperative recovery?**

- **Follow medical advice**: Always listen to your doctor or physical therapist. They will give the best guidance on when and how to start exercises.
- **Avoid high-impact activities**: During recovery, exercises like jumping, running, or heavy lifting could put too much pressure on the hip joint.
- **Take it slow**: Don’t rush recovery. Start with gentle exercises and slowly increase intensity as the hip gets stronger.
- **Watch for pain**: If the child feels pain, stop the exercise and contact the doctor.

**5. Does DDH affect the quality of my sleep? What are some ways to improve sleep and help the body recover?**

- **Effect on sleep**: In some cases, DDH might cause discomfort, which could affect sleep. If the hip is unstable or painful, it can make it harder to sleep at night.
- **Ways to improve sleep:**
  - **Comfortable sleeping position**: Use pillows to support the hips and make the child feel more comfortable while sleeping.
  - **Pain relief**: Follow the doctor’s advice on pain management to help the child sleep better.
  - **Routine**: Keeping a regular sleep schedule helps the body recover and rest properly.
  - **Calming environment**: A quiet, dark room can help the child sleep more soundly.

In all cases, it’s important to stay in touch with the doctor to make sure your child is healing well and to get personalized advice.

Sure! Let's talk about **developmental dysplasia of the hip (DDH)** in children. This is a condition where the hip joint doesn’t form properly, which can lead to hip problems as the child grows. It’s often treated with braces, casts, or sometimes surgery.

**Psychological Problems with DDH or After Surgery:**

1. **Anxiety**: A child with DDH might feel anxious, especially if they need to wear a special brace or have surgery. The thought of being different from other kids, or the fear of the unknown (like surgery), can be overwhelming. Parents might also feel anxious about the treatment and the child’s future.

*Example*: Imagine a child who has to wear a large hip brace for months. They might worry about being made fun of at school or miss out on playing with friends.

1. **Depression**: In some cases, the child might feel sad or upset. If they can’t participate in activities like other children because of the condition or recovery, they may feel left out. Parents can also feel stressed or sad watching their child go through the treatment.

*Example*: After surgery, a child may feel frustrated about not being able to run or play sports like before. Over time, this can lead to feelings of sadness.

**How to Deal with These Feelings Positively:**

1. **Communication**: Talk to your child openly about how they feel. Reassure them that it’s okay to feel upset and that they’re not alone in this. Listen to their concerns.
2. **Provide Comfort and Support**: Give extra love and care. Keep their routine as normal as possible and encourage small activities they enjoy, even if they can’t do everything right away.
3. **Stay Positive and Encourage Small Wins**: Celebrate the small achievements during recovery. If they take a few steps without pain or get through a day wearing their brace, give them praise. This can boost their mood.
4. **Seek Professional Help**: If you notice that the anxiety or sadness is lasting longer, a therapist or counselor who specializes in working with kids can help. Sometimes, therapy can help children express their feelings and learn how to cope.

**Signs You Might Need Psychological Support:**

- **Frequent Sadness or Mood Swings**: If your child seems unusually sad or has big mood changes that last for weeks.
- **Avoiding Activities**: If they start avoiding things they once enjoyed, like playing with friends or going outside, because of their condition or recovery.
- **Trouble Sleeping**: If they have trouble sleeping or seem worried a lot, it could be a sign they are anxious.
- **Anger or Frustration**: If they get angry or frustrated easily, or have trouble calming down.

**How to Find Help:**

1. **Talk to a Doctor**: Your child’s doctor might be able to recommend a counselor or psychologist who works with children, especially those with medical conditions like DDH.
2. **Look for Child Therapy Services**: Many hospitals or clinics have therapists who specialize in working with kids. You can ask about this during your regular appointments.
3. **Support Groups**: Look for groups where parents of children with DDH connect. They can share their experiences and offer advice on handling emotional challenges.
4. **Online Resources**: Websites like the American Academy of Pediatrics (AAP) and the International Hip Dysplasia Institute offer resources and advice for families dealing with DDH.

It’s important to know that feeling anxious or sad during treatment or recovery is common, and help is available. Talking about it and getting support can make a big difference for both the child and their family.

Sure! Let’s break it down using **developmental dysplasia of the hip (DDH)** in children.

**1. Health Apps or Devices to Manage DDH or Postoperative Recovery:**

There are a few apps and devices that could be helpful for managing DDH, especially after surgery. These tools can help both parents and doctors track the child's recovery:

- **Pain Management Apps**: Apps like *Medisafe* or *PainScale* help parents track pain levels. They allow you to record when your child feels pain and how severe it is. This can help doctors know if they need to adjust medications or treatments.
- **Physical Therapy Apps**: After surgery, children often need physical therapy to help their hip move properly again. Apps like *PhysiApp* or *PT Timer* show exercises and remind you to do them. They can guide you through exercises that improve strength and movement in the hip.
- **Wearable Devices**: Some devices can track physical activity and recovery. For example, a simple pedometer or fitness tracker like *Fitbit* can monitor how much your child is moving. It helps you know if they’re walking more after surgery or if they need more rest.
- **Post-Surgery Care Apps**: Some apps help track appointments, medications, and progress. *CareClinic* is an example. Parents can use it to log recovery milestones and any complications, so doctors can see the full picture when they check in.

**2. Using Health Tracking Tools to Monitor DDH or Recovery:**

After surgery for DDH, monitoring your child's progress is key. Health tracking tools can help in several ways:

- **Tracking Pain**: You can use apps to rate your child’s pain each day. If pain goes down, that’s a good sign of healing. If it stays the same or gets worse, you might need to check in with the doctor.
- **Monitoring Physical Activity**: A fitness tracker can measure how active your child is. If the tracker shows little movement, it might mean the child is not walking enough. On the other hand, if they’re over-exerting themselves, it might be a sign to rest more.
- **Medication Reminders**: Post-surgery, your child may need medication. Apps like *Medisafe* can remind you when to give them medication and keep track of doses to avoid mistakes.
- **Recording Recovery Milestones**: Apps like *CareClinic* or even a simple note-taking app can help you document your child's progress. You can note if your child is walking, playing, or doing physical activities they couldn’t do before surgery. This gives a picture of how they are recovering over time.

In short, health apps and devices help you track important things like pain, activity, medication, and milestones, so you can make sure your child is recovering well from DDH or surgery. They also help keep everything organized, making it easier to share progress with doctors.

**1. How to access reliable information about DDH (Developmental Dysplasia of the Hip) or post-operative care:**

When you're looking for trusted information about DDH or what to do after surgery, you want to rely on sources that doctors, hospitals, or other medical experts trust. Here are some good options:

- **Doctor or healthcare provider**: Always ask your child’s doctor or specialist. They can give you information specific to your child’s case and explain things clearly.
- **Reputable websites**: Websites like the **American Academy of Orthopaedic Surgeons (AAOS)** or the **National Institutes of Health (NIH)** provide reliable information.
- **Hospitals and clinics**: Many hospitals have detailed guides for parents. Check their websites for helpful resources or ask them for brochures or pamphlets.
- **Books**: There are books written by medical professionals that cover DDH and how to take care of it after surgery.

**2. Are there support groups or community resources that can help me or my family cope better?**

Yes, there are many ways to find support for families dealing with DDH:

- **Support groups**: Many hospitals or local organizations run groups for parents of children with DDH. These groups let you connect with other families going through similar experiences. You can share stories, advice, and emotional support.
- **Online communities**: Websites like Facebook and Reddit have groups specifically for DDH. You can ask questions, find support, and learn from others who’ve been through it.
- **Nonprofits and foundations**: Some organizations focus on hip health and DDH. They may provide information, resources, or even financial help if your child needs treatment. Examples include the **International Hip Dysplasia Institute (IHDI)** and other related organizations.
- **Therapists or counselors**: Sometimes talking to a professional can help your family cope with the stress of DDH. A therapist can give advice on how to handle emotional challenges that might come with caring for a child with DDH.

Remember, you're not alone in this, and there are people and groups ready to support you!

**What is the usual cost of treatment for developmental dysplasia of the hip (DDH)?**

The cost of treating DDH in children can vary depending on the severity of the condition, the type of treatment needed, and where you live. For example:

1. **Non-surgical treatments (like braces or hip harnesses)**: This can cost around $500 to $2,000, depending on the type of device and the time it's needed.
2. **Surgical treatments**: If surgery is needed (like hip surgery or a procedure to move the hip into place), it can cost between $5,000 to $20,000 or more. This includes hospital fees, surgeon's fees, and post-surgery care.
3. **Physical therapy and follow-up visits**: After treatment, your child may need physical therapy, which can add to the cost.

However, many of these treatments can be covered by **insurance**, depending on your plan.

**Are there insurance or subsidy policies to reduce the financial burden?**

Yes, there are insurance and subsidy options available to help reduce the cost:

1. **Health insurance**: Most health insurance plans will cover treatments for DDH, but you should check your specific plan to see what’s included. Some insurance plans cover surgery, physical therapy, and medical devices (like braces).
2. **Government health programs**: In many countries, there are government programs or subsidies that help with the costs of medical treatments, especially for children. This could include public health insurance or financial assistance programs.
3. **Charity or Non-profits**: Some non-profit organizations may help cover the cost of surgery or devices, especially for children. It's worth looking into local community resources or medical charities.

**What social assistance can I apply for if the disease or surgery causes financial hardship?**

If DDH treatment causes financial hardship, there are a few types of social assistance that may be available:

1. **Financial aid programs**: Many hospitals or clinics offer financial assistance programs for families who are struggling to pay medical bills. They may reduce the cost or set up a payment plan.
2. **Government financial support**: If you're in financial need, you can apply for government support programs that help with medical expenses. This could be a special grant, social welfare, or low-income healthcare coverage.
3. **Disability benefits**: If DDH leads to long-term disability or complications, your child might qualify for disability benefits. This can help cover medical costs, therapy, or equipment needed for daily life.
4. **Charity programs**: Some charities offer financial support or fundraisers to help families with expensive medical treatments.

It’s a good idea to talk to your doctor or a hospital social worker to find out what help is available in your area. They can point you to programs that might cover part of the cost.

Sure! Let’s break this down into simple ideas and talk about developmental dysplasia of the hip (DDH) in children.

**How can I balance mental health, family responsibilities, and work in disease management?**

Managing a child’s condition like DDH can be stressful. It may require frequent doctor visits, treatments, or even surgery. Here's how you can balance everything:

1. **Mental Health:**
   - **Take care of yourself:** Make sure to rest, eat well, and relax. If you’re feeling overwhelmed, it's okay to ask for help or talk to a counselor.
   - **Stay positive:** Focus on the small wins. Celebrate when your child’s condition improves or when you manage a difficult situation well.
2. **Family Responsibilities:**
   - **Share tasks:** If you have a partner or other family members, ask them to help with things like taking your child to appointments or managing household chores.
   - **Communicate:** Make sure everyone in your family knows what needs to be done and how they can support you.
3. **Work:**
   - **Flexible Schedule:** If possible, talk to your employer about flexible hours or remote work, especially during busy times like doctor appointments or recovery.
   - **Prioritize tasks:** Focus on the most important work and let go of less urgent tasks if you’re feeling stressed. Your health and family come first.

**How might DDH affect future quality of life? How can I plan ahead to improve my health?**

DDH can affect how your child moves and their ability to do things like walk, run, or play sports. If untreated, it may cause pain or arthritis as they grow older. However, with early treatment, many children lead normal, active lives.

1. **Future Quality of Life:**
   - **If treated early:** The sooner DDH is diagnosed and treated, the better the long-term outcome. Surgery or a brace might be needed, but it can prevent problems later on.
   - **Without treatment:** If it’s not treated, the hip joint may wear out quicker, causing pain or even needing more surgeries later in life.
2. **Planning Ahead:**
   - **Follow-up care:** Regular doctor visits to check on how your child’s hips are doing will help catch any problems early.
   - **Physical therapy:** Doing exercises or stretches might help your child move better and reduce pain.
   - **Healthy lifestyle:** Encourage activities like swimming or biking to keep joints strong and flexible. Stay active to maintain overall health.

In short, by staying on top of treatment and caring for both your child's health and your own mental well-being, you can improve the long-term outlook for DDH and ensure a better quality of life.

**Reply from ChatGPT-4 (Third time)**

**What is Developmental Dysplasia of the Hip (DDH)?**

Developmental Dysplasia of the Hip (DDH) is a condition where a baby’s hip joint doesn't form properly. Instead of the ball of the hip fitting snugly into the socket, it can be loose or even dislocated. Think of it like a puzzle piece that doesn’t quite fit. If left untreated, this can cause pain and problems with walking as the child grows.

**Who is most likely to develop DDH? How common is it?**

DDH is more common in:

- **Girls** (they are about four times more likely than boys).
- **First-born babies** (because their hips may be more tightly positioned in the womb).
- Babies born **in a breech position** (feet or bottom first, instead of head first).
- It can also run in families, so if a sibling had DDH, there’s a higher chance of it happening again.

DDH is **fairly common**—about 1 in 100 to 1 in 1,000 babies are affected, but some mild cases may not show up right away.

**Will DDH have a long-term impact on my quality of life? What aspects should I pay special attention to?**

If treated early, children with DDH can grow up with little to no long-term problems. However, if it’s not treated, it can lead to:

- **Hip pain** as the child gets older.
- **Arthritis** in the hip joint because the joint doesn’t work smoothly.
- Difficulty walking or running properly.

It’s important to pay attention to how your child walks. If they have trouble walking, their legs seem different lengths, or they have a limp, you should talk to a doctor. Early treatment usually means better outcomes.

**What parts of the body does developmental dysplasia of the hip (DDH) mainly affect? What do these parts do in a healthy state?**

DDH mainly affects the **hips** in children. The **hip joint** is where the **thigh bone** (femur) fits into the **pelvis** (the part of the body that holds your organs like your stomach).

In a healthy hip:

- The thigh bone should fit tightly and smoothly into the pelvis.
- This allows you to **walk**, **run**, and **move your legs** without pain or stiffness.

**What symptoms or consequences may arise if these parts are damaged? Can you provide specific examples?**

If the hip joint doesn’t form properly (as in DDH), the thigh bone may not fit tightly into the pelvis. This can cause several issues:

1. **Pain and discomfort**: The child might feel pain in the hip, especially when walking or running. For example, a child might limp or not want to walk as much.
2. **Stiffness or limited movement**: If the joint doesn’t move properly, the child may have trouble bending their legs. For example, they might not be able to sit cross-legged or squat comfortably.
3. **Limping or walking awkwardly**: A child may start walking with a limp or dragging one leg, because the hip joint is not stable.
4. **Arthritis in the future**: If left untreated, the hip joint can wear down over time, which can lead to arthritis (pain and swelling in the joints) as the child grows older.

In severe cases, DDH can make it hard for a child to walk at all, and they may need surgery to fix the hip joint. Early treatment can help prevent most of these issues.

Sure! Let’s break down *Developmental Dysplasia of the Hip* (DDH) in children and answer your questions clearly.

**What are the main causes of DDH?**

DDH happens when a baby’s hip joint doesn't develop properly. Normally, the hip joint has a ball-and-socket shape, where the ball (the top of the thigh bone) fits snugly into the socket of the hip bone. In DDH, the socket may be too shallow or the ball may not fit properly.

The exact cause isn’t always clear, but it can happen because of:

1. **Genetics (Family History):** If other family members had DDH, the baby might be more likely to have it.
2. **Position in the Womb:** If the baby is cramped in the womb (like being in a breech position, where the baby’s feet are down instead of the head), it can affect the hip's development.
3. **Other Factors:** Sometimes, babies born prematurely or with other medical conditions may be more likely to have DDH.

**What risk factors may increase the chances of developing DDH?**

1. **Breech Birth:** Babies who are born feet-first (breech) are more likely to have DDH.
2. **Firstborn Babies:** The risk is higher in first-born children because the womb has less space for movement.
3. **Family History:** If a parent or sibling had DDH, the risk is higher.
4. **Gender:** Girls are more likely to develop DDH than boys.

**What lifestyle habits or environmental factors increase the risk of developing DDH? How can I prevent it?**

While DDH is mostly about genetics and the baby’s position in the womb, certain things can help lower the risk:

- **Proper Positioning:** After birth, avoiding swaddling the baby’s legs too tightly and allowing them to move their hips freely can help. Avoiding positions that restrict the baby’s hips, like keeping the legs straight and together, can be helpful.
- **Early Detection:** DDH is often found during a baby’s first check-up. If it's caught early, it can be treated quickly. So, it’s important to go to all scheduled pediatric appointments.

**If I have a family history, is my risk of developing DDH higher? What preventive measures should I take?**

Yes, if there is a family history of DDH, your baby might have a higher risk of developing it.

The best preventive measure is early screening. Babies are often checked for DDH in their first few weeks of life. If the doctor catches it early, there are treatments like braces or harnesses that can help the hip develop properly. In rare cases, surgery may be needed later if it isn’t detected early.

**To summarize:**

- **Causes of DDH:** It can be caused by genetics, the baby’s position in the womb, or other health factors.
- **Risk Factors:** Breech birth, being the firstborn, and having a family history can increase the risk.
- **Prevention:** Ensuring the baby’s hips have room to move and getting early check-ups can help. If there’s a family history, regular screenings can help catch DDH early.

Let me know if you need further clarification!

**Main Symptoms of Developmental Dysplasia of the Hip (DDH)**:

DDH is a condition that affects babies and young children, where the hip joint doesn’t develop properly. This can make the ball part of the hip joint not fit well into the socket. Here are the common symptoms:

- **For babies**: You might notice one leg appears shorter than the other, or that one leg seems to turn outward. Sometimes, the baby might have trouble moving their hips or legs normally.
- **For toddlers and older kids**: They may have a limp, walk on their tiptoes, or have trouble walking. They might also complain of hip pain or have trouble moving their hip joint easily.

**Signs the Condition Might Be Worsening**:

If DDH isn't treated, it can get worse. You might notice things like:

- **Increased pain**: The child may start complaining of hip or groin pain, especially while walking or running.
- **Limited movement**: They might find it harder to move their leg or hip or complain that it feels stiff.
- **Walking problems**: The child may start limping or walking unevenly, or they might stop walking altogether if they’re in too much pain.

**Should I Seek Immediate Medical Attention?**:

If you notice any of these signs in your child, it’s important to see a doctor. Early treatment is key to fixing DDH before it causes more problems. If you see your child having trouble moving their leg, or if they are in pain and can’t walk or move normally, it’s a good idea to seek medical attention sooner rather than later. A doctor can help figure out what’s going on and how to treat it.

**Real-life example**:

Imagine a toddler who loves running around the park but starts limping and refuses to walk after a while. This could be a sign that their hip joint isn't working properly. It's a good idea to take them to the doctor, who might run some tests, like an X-ray or ultrasound, to see if DDH is the cause.

The sooner it’s spotted, the easier it is to fix!

Sure! Let's break this down step-by-step, using **developmental dysplasia of the hip (DDH)** in children as the example.

**1. How do doctors confirm the diagnosis of DDH?**

To confirm if a baby or child has DDH, doctors usually use the following methods:

- **Physical Exam**: The doctor checks if the baby’s hips feel stable. They might gently move the baby's legs to see if there’s any clicking sound or looseness. This can be done in a simple check when the baby is only a few weeks old.
- **Ultrasound**: If the baby is very young (usually under 6 months), an ultrasound may be used. This test uses sound waves to create an image of the hip joint. It helps doctors see if the ball of the hip is in the socket correctly or if it’s too loose or out of place.
- **X-rays**: For older children (over 6 months), doctors often use X-rays to get a clear image of the bones. X-rays help doctors see if the hip joint is shaped properly and if the ball is staying in the socket like it should.

**Purpose of each test**:

- The **physical exam** helps doctors spot early signs of DDH by feeling the hips and moving the legs.
- **Ultrasound** helps doctors see soft tissues and joints in younger babies.
- **X-rays** give a clear picture of the bones in older babies and children.

**2. What indicators are most important in test results? How do these results reflect the severity of the disease?**

- **Hip joint position**: The most important thing doctors look for is how well the hip joint fits together. In DDH, the ball (femoral head) might not fit snugly in the socket (acetabulum), which can cause instability or even dislocation.
- **Angle of the hip joint**: Doctors measure the angle at which the ball fits into the socket. If the angle is too wide, it can show the hip isn’t properly aligned.
- **Movement**: During physical exams, if the baby’s leg can move easily or "click" out of place, it suggests the hip joint is loose, which is a sign of DDH.

**What do these results mean for severity?**

- If the hip is only slightly out of place, doctors might call it **mild** DDH. In these cases, the baby might just need a simple treatment like a special harness.
- If the ball of the hip is far out of the socket or the joint isn’t shaped correctly, it might be more **severe**. This could require more treatment, like surgery or casting, to help the hip joint grow properly.

**3. After confirming the diagnosis, do I need regular follow-ups?**

Yes, follow-up appointments are usually needed to make sure the hip joint is developing correctly over time.

- **How often should follow-ups be?**
  - For **mild cases**, the doctor might want to check the hip every few months, especially during the first year.
  - For more **severe cases**, follow-ups might be more frequent, such as every 1–2 months, depending on treatment (like a harness or surgery).
- **What should be checked?**
  - The doctor will check if the hip is still in the correct position.
  - They may repeat **ultrasounds or X-rays** to see how the hip is growing and developing.
  - The doctor will also check if the baby is moving their legs normally and if there are any signs of problems, like pain or difficulty moving.

These regular visits are important because they help catch any problems early and make sure the treatment is working. If the hip is not developing correctly, the doctor might change the treatment plan.

So, to sum it up:

- **Diagnosis** involves physical exams, ultrasounds, and X-rays.
- **Test results** show if the hip joint is in the right position, and this tells doctors how serious the DDH is.
- **Follow-ups** help doctors track progress and adjust treatment if needed.

Does that help? Let me know if you'd like me to go over anything in more detail!

**Symptoms That Need Immediate Medical Attention for DDH in Children:**

Developmental dysplasia of the hip (DDH) happens when a child's hip joint doesn’t form properly. This can make movement painful or difficult. Here are some symptoms that should make you seek medical help right away:

1. **Pain in the Hip or Groin Area**: If your child complains of pain in their hip or groin, especially when moving, it could be a sign that something is wrong with their hip joint.
2. **Limping or Difficulty Walking**: If your child starts limping, refuses to walk, or avoids putting weight on one leg, it’s important to get checked by a doctor.
3. **Uneven Leg Length**: If one leg appears shorter than the other or looks differently shaped, it may be a sign of DDH.
4. **Limited Range of Motion**: If your child’s leg cannot move normally, like when you try to change their diaper or help them move, it could indicate DDH.
5. **Swelling or Bruising**: Swelling, bruising, or visible deformity around the hip after a fall or injury should be treated as an emergency.

**Handling and Self-Monitoring During an Emergency:**

If you notice any of these symptoms, here’s how you can handle the situation and monitor your child:

1. **Stay Calm**: It’s important not to panic. Stay calm so you can help your child in the best way possible.
2. **Limit Movement**: If your child is in pain or limping, try to limit their movement to prevent further injury. You can help them sit down or carry them carefully.
3. **Keep Them Comfortable**: Make sure your child is comfortable while waiting for medical help. You can try to keep them still and warm, and reassure them it’ll be okay.
4. **Call for Help**: If the symptoms are severe, like pain, swelling, or difficulty moving, call a doctor or go to the emergency room right away. Time matters when dealing with joint issues.
5. **Note Any Changes**: Keep an eye on any changes in your child’s condition, such as more swelling, redness, or more difficulty moving. This information can help doctors figure out the best treatment.

By keeping an eye on these signs and acting quickly, you can help your child get the treatment they need to feel better sooner.

Sure! Let’s break it down using developmental dysplasia of the hip (DDH) in children. DDH is a condition where a child’s hip joint doesn’t form properly, which can lead to the ball of the thigh bone not fitting into the hip socket. Here’s a simple explanation of your questions:

**1. What treatments will I receive during hospitalization? What is the purpose of each treatment?**

When a child is in the hospital with DDH, there are a few treatments they might receive:

- **Pavlik Harness**: This is a special strap device used to hold the baby’s legs in a specific position to help the hip joint grow properly. It’s like a comfy brace that helps keep the hip in place while it develops.
- **Casting or Bracing**: Sometimes, if the harness doesn’t work, doctors use a full-body cast or other types of braces to keep the hip joint in the right position.
- **Surgery**: If the hip joint is very out of place, surgery might be needed to reposition the bones and help them grow correctly. This is usually done if other treatments don’t work.
- **Pain management**: If the child feels pain, the hospital will give medicine to help them feel better.

The goal of all these treatments is to make sure the hip joint develops properly and works like it should.

**2. How can I prevent infections or other complications during hospitalization? What can family members do to assist with recovery?**

To prevent infections or other problems during the hospital stay:

- **Cleanliness**: Make sure the child’s skin stays clean, especially around the cast or harness. This will help prevent infections. Family members can help by keeping the area dry and clean.
- **Monitoring for problems**: Watch for any signs of infection like redness, swelling, or unusual smells. Let the doctor know right away if these happen.
- **Pain management**: Family members can help by making sure the child gets their medicine on time to keep pain under control. This helps them recover faster and feel more comfortable.
- **Follow the doctor’s advice**: The family should help make sure the child wears the harness or cast as instructed and limits movement to avoid any damage to the hip.

**3. How can I reinforce the effects of treatment after discharge? What rehabilitation programs are recommended?**

After leaving the hospital, it’s important to continue the treatment at home:

- **Continue using the harness or braces**: The doctor will tell you how long the child needs to wear the harness or brace. Follow those instructions exactly.
- **Physical Therapy (PT)**: In some cases, the doctor may recommend physical therapy after treatment. PT helps children strengthen muscles around the hip and improve movement.
- **Regular check-ups**: The child will need follow-up visits with the doctor to make sure the hip is healing properly. These check-ups are important to ensure the treatment is working.
- **Activity restrictions**: The child might need to avoid certain activities, like running or jumping, for a while until the hip is fully healed.

Family members can support the recovery process by reminding the child to follow the doctor’s instructions and helping them with physical therapy exercises if needed.

In short, the key to treating DDH is sticking to the treatment plan, preventing infections, and following the doctor’s advice for a smooth recovery.

Sure! Let’s break it down in simple terms. We'll use *developmental dysplasia of the hip (DDH)* in children as an example.

**1. Common medications used to treat DDH in children and how to take them correctly:**

For DDH, kids may need different treatments, depending on how severe the condition is. **Medications are not always the main treatment**, but they might be used for pain relief or to help relax muscles around the hip joint.

- **Pain medications**: If a child feels pain (usually after surgery or a procedure), a doctor might recommend things like **acetaminophen (Tylenol)** or **ibuprofen (Advil)**. These help with pain and inflammation.
- **Muscle relaxants**: Sometimes, medications like **diazepam (Valium)** might be used if the muscles around the hip are too tight. This helps the muscles relax.

**How to take them correctly**:

- Follow the doctor’s advice or the instructions on the label. For example, if your doctor tells you to give a child a painkiller every 6 hours, do it exactly at those times.
- Always check the **dosage** based on the child’s age or weight.

**2. What to do if you forget to take the medication or take it at the wrong time?**

If you forget to give the medication, don’t worry too much. Here’s what you can do:

- **If it's almost time for the next dose**: Just skip the missed dose and give the next one at the regular time.
- **If it's still early**: Take the missed dose as soon as you remember, unless it’s too close to the next dose.

**Will it affect the treatment?**

- Missing one dose usually won't harm the treatment much. However, it’s important to stay on track because skipping doses regularly can slow down recovery.

**3. What foods should I avoid while taking medication for DDH? Are there common side effects?**

- **Food to avoid**: Most medications used for DDH don't need to be taken with special foods. However, for **pain meds like ibuprofen**, it’s a good idea to give them with food to prevent stomach upset.
- **Common side effects**:
  - **Pain medications** like acetaminophen or ibuprofen can sometimes cause upset stomachs or drowsiness.
  - **Muscle relaxants** like diazepam might make the child sleepy or dizzy.

Always ask your doctor if you’re unsure about certain foods or side effects related to any medications.

By following these simple steps, you’ll be able to help your child get the best care for DDH!

**1. What types of pain may be caused by DDH or its treatment? Where is the pain usually felt?**

**DDH (Developmental Dysplasia of the Hip)** happens when a child's hip joint doesn’t form properly. This can cause pain because the joint doesn't move as it should.

- **Pain from DDH:**
  - The pain can happen in the **hip**, **groin**, or sometimes the **thigh** or **knee**. It can feel sharp or dull.
  - Babies may not be able to say they’re in pain, but they might cry more when you try to move their legs or change their position.
- **Pain from treatment (e.g., a brace or cast):**
  - After treatments like wearing a **Pavlik harness** (a special brace for babies), the skin may get irritated, causing discomfort.
  - In older children, surgery or wearing a cast may lead to some soreness and stiffness in the area around the hip.

**2. What methods can help relieve pain? What are the recommended pharmacological and non-pharmacological treatments?**

**Non-pharmacological treatments:**

- **Gentle movement:** For babies and kids, carefully stretching or moving the leg (under doctor guidance) can help keep the joint flexible.
- **Massage and warmth:** Massaging the hip area gently or using a warm compress can relax muscles and reduce pain.
- **Changing positions:** Keeping the child comfortable by changing their position or using pillows for support can help avoid pressure on the painful area.

**Pharmacological treatments (medications):**

- **Pain relievers:** For children, doctors may suggest **acetaminophen** (like Tylenol) or **ibuprofen** (like Advil) to reduce pain and inflammation.
- **Topical treatments:** Sometimes, doctors might recommend creams or gels for extra pain relief.

**3. What steps should I take if the pain is more than expected or worsens? Do I need to contact my doctor to adjust my treatment plan?**

- **If the pain gets worse or feels much stronger than expected**, it's important to check in with the doctor. Sometimes, pain can mean something isn’t right, like a problem with the treatment or a sign of infection.
- **Call your doctor right away** if:
  - The child is crying more than usual, even after pain medicine.
  - You notice **swelling**, **redness**, or **fever** near the hip.
  - The child refuses to move their leg or can't put weight on it.
- **Adjusting treatment:** The doctor may want to change the treatment plan if the pain doesn't get better. This could involve switching medications, adjusting the brace or cast, or exploring other treatments like physical therapy.

If the pain doesn't go away, or if it's much worse than what the doctor expected, it’s always a good idea to ask for help.

Sure! Let’s talk about post-surgery care for a child who had surgery for developmental dysplasia of the hip (DDH), in simple words.

**1. What post-surgery care precautions should I take?**

- **Keep the hip protected:** After surgery, the hip joint needs time to heal. Avoid moving the leg in ways that could stress the joint, like crossing the legs or putting weight on it too soon.
- **Use braces or casts if needed:** Your doctor might give a brace or cast to keep the leg in the right position.
- **Follow the doctor’s instructions carefully:** Be sure to stick to the schedule for follow-up visits and take any medications prescribed to manage pain or prevent infection.

**2. How long after surgery can I return to normal daily activities?**

- **Usually a few months:** Most children can start doing their normal activities after 3 to 6 months, but it depends on how well the hip is healing. Your doctor will guide you on when it’s safe to return to full activities.

**3. How soon after surgery can I begin resuming activities?**

- **Start slowly after a few weeks:** Light activities like walking or gentle stretching can begin after a few weeks, but nothing too intense. The doctor will tell you when it’s okay to start more active movements.

**4. What activities are recommended during the recovery period?**

- **Gentle exercises:** Activities like swimming or gentle leg movements can help the muscles around the hip get stronger without putting too much pressure on the joint.
- **Walking with support:** Your child might be encouraged to walk with crutches or a walker at first.

**5. How do I schedule my exercise during the postoperative recovery period?**

- **Follow the doctor’s plan:** Your doctor or physical therapist will create a safe exercise schedule. This might start with stretching and light activities, then build up as the hip gets stronger.
- **Don’t rush:** It’s important to wait for the doctor’s approval before trying harder exercises.

**6. What are the stages during postoperative recovery? What precautions should I take in each stage?**

- **Stage 1 (Immediate recovery):** Right after surgery, the child will need rest and pain management. Keep the leg in a stable position as advised by the doctor.
- **Stage 2 (Early recovery):** After a few weeks, gentle movements and stretches can begin. Avoid putting weight on the leg until the doctor clears it.
- **Stage 3 (Later recovery):** As the hip heals, more strength-building exercises can be done. But avoid sports or heavy activities until the doctor says it's okay.

**7. Do I need a specialized rehabilitation plan? How does it help with recovery?**

- **Yes, a rehabilitation plan is very important.** A physical therapist will create a plan that helps your child move properly again. This plan includes exercises to improve strength, flexibility, and balance, which speeds up recovery.

**8. How can I prevent recurrence after surgery?**

- **Follow the doctor’s guidelines carefully:** Make sure your child does the recommended exercises, wears braces or supports if needed, and avoids risky movements that could harm the joint again.
- **Regular check-ups:** Continue to see the doctor for regular check-ups to make sure the hip is healing well.

**9. What complications can occur after surgery? What steps should I take?**

- **Infection:** Keep the wound clean, and watch for signs like redness, swelling, or fever. Contact the doctor right away if these occur.
- **Hip dislocation or stiffness:** If the child feels pain or can’t move the leg normally, see the doctor right away.
- **Blood clots:** In rare cases, blood clots can form. Be aware of swelling or pain in the leg, and contact the doctor immediately if you suspect this.

Each recovery journey can be different, so always follow the specific advice your doctor gives you!

**Developmental Dysplasia of the Hip (DDH) in Children:**

DDH is a condition where the hip joint doesn’t develop properly. In some cases, the ball of the hip doesn’t fit into the socket. It can cause problems with movement and might need treatment, such as surgery. Here’s how we can think about recovery and care:

**1. What foods are helpful during recovery from DDH or surgery?**

- **Calcium-rich foods:** Foods like milk, cheese, yogurt, and leafy greens (like spinach) help bones heal and grow stronger.
- **Vitamin D foods:** These help your body absorb calcium. Good sources include fish (like salmon), eggs, and fortified foods like some cereals and juices.
- **Protein-rich foods:** After surgery, protein helps muscles and tissues recover. Eggs, chicken, beans, and tofu are great options.

**Foods to avoid:**

- **Processed foods:** High-fat, sugary, or salty foods can slow down recovery. Avoid chips, cookies, and sugary drinks.
- **Too much caffeine:** It can affect calcium absorption, so try not to have too much coffee or soda.

**2. How should I adjust my diet to prevent constipation or other problems?**

- **Fiber-rich foods:** Foods like whole grains, fruits (like apples and berries), and vegetables (like broccoli) can help with digestion and prevent constipation.
- **Drink plenty of water:** Staying hydrated is very important to keep things moving in your digestive system.
- **Small, balanced meals:** Eating smaller meals throughout the day helps the digestive system work better.

**3. What exercises can I do during the recovery phase? How often should I do them?**

- **Gentle stretching:** Stretching helps with flexibility and keeps joints moving. Try simple stretches for the legs and hips.
- **Walking:** Once you're able, light walking can strengthen muscles around the hip. Start with short walks and gradually increase.
- **Swimming:** Swimming is a great exercise for joint recovery because the water supports the body while you move. It’s gentle on the hips.

**How often?**

- Start slow with exercises 2-3 times a week. As recovery goes on, increase the number of days you exercise, but always listen to your body.

**4. What precautions should I take when exercising, especially during postoperative recovery?**

- **Avoid high-impact activities:** Don’t do running, jumping, or activities that put too much pressure on the hip early in recovery.
- **Use supportive devices:** If your doctor recommends a brace or special shoes, wear them as directed.
- **Go slow:** Don’t push yourself too hard, especially if you're feeling pain or discomfort.

**5. Does DDH affect the quality of my sleep? What are some ways to improve sleep and help the body recover?**

- **Pain or discomfort from the hip can make sleep difficult** if the joint is still healing. If your child is experiencing pain, they might find it hard to get comfortable at night.

**To improve sleep:**

- **Positioning:** Use pillows to support the body and keep the hip in a comfortable position. For example, place a pillow between the legs when lying on the side.
- **Pain management:** Follow your doctor’s advice about pain medicine to help sleep better.
- **Routine:** Try to establish a regular bedtime routine to help the body relax and fall asleep easier.

Recovery can be tough, but with the right foods, exercises, and self-care, it’s possible to heal and feel better. Always check with a doctor or therapist before starting any new exercises or making major changes to diet or lifestyle.

Sure! Let's break this down with the example of **Developmental Dysplasia of the Hip (DDH)** in children.

**1. Psychological problems related to DDH or recovery:**

When a child has DDH, it means their hip joint hasn’t developed properly, which can cause pain or difficulty moving around. If a child needs surgery to fix it, this can be stressful for both the child and the parents. During recovery, a few emotional or mental health challenges may come up:

- **Anxiety**: The child might feel nervous or scared about the surgery or recovery process. They may worry about being in pain, staying in the hospital, or not being able to play like other kids.
- **Depression**: After surgery, the child might feel sad or frustrated because they can't do things they normally enjoy. They may also feel isolated if they can’t play with friends for a while.
- **Stress**: Parents might also feel stressed about their child’s recovery and the changes it brings to daily life, like needing help with basic tasks or worrying about the child’s health.

**How to deal with these feelings positively:**

- **Talk openly**: Let your child express their fears or concerns. If you’re the parent, it helps to stay calm and reassuring. Let them know it’s okay to feel worried, but they are not alone.
- **Set small goals**: After surgery, try setting little goals for progress. For example, “Today, we’re going to walk a little further than yesterday.” This gives a sense of accomplishment.
- **Offer comfort**: Let your child know that recovery takes time, and you’re there with them every step of the way. A favorite toy, book, or game can make them feel better.
- **Encourage fun activities**: Find activities that they can still enjoy, like drawing or watching movies, to help distract them and boost their mood during recovery.

**2. Signs you may need psychological support:**

If the feelings of anxiety, sadness, or stress are not getting better and start affecting daily life, it might be time to seek professional help. Some signs that it’s time for psychological support include:

- **Constant worry or fear**: If the child is feeling nervous about simple things like going to school or playing with friends.
- **Lack of interest**: If the child is no longer interested in things they used to enjoy.
- **Trouble sleeping or eating**: If the child is having trouble sleeping or eating, or if their sleep patterns or appetite change a lot.
- **Feeling hopeless**: If they say things like "I don't want to get better" or show a lack of hope about recovery.

**Finding professional help or support resources:**

- **Talk to your doctor**: If you notice these signs, start by speaking to your child’s doctor. They can refer you to a psychologist or counselor who specializes in helping children with medical conditions.
- **Visit a therapist**: Therapists can help children understand and express their emotions, and teach coping strategies. Some therapists may even offer family counseling to support everyone involved.
- **Support groups**: Sometimes it helps to talk to other parents who have gone through the same thing. There are online groups or local meetups for families dealing with DDH or children’s surgeries.
- **Hotlines or online resources**: Many countries have helplines for mental health support that are free and confidential.

Taking care of your child’s emotional health is just as important as their physical recovery. A combination of open communication, patience, and professional support can help both the child and parents cope positively through the process.

Sure! Let's break this down using **Developmental Dysplasia of the Hip (DDH)** as an example.

**1. Are there any recommended health apps or devices that can help manage DDH or postoperative recovery?**

For DDH, there are no specific apps just for this condition. However, there are general apps and devices that can help:

- **Physical Therapy Apps**: These apps show you exercises to help strengthen the hip and improve movement. Examples include apps like **PT Timer** or **PhysioAdvisor**.
- **Pain Management Apps**: Apps like **myPainDiary** can help track pain levels, which is useful during recovery after surgery or treatment.
- **Post-Surgery Tracker**: If someone had surgery for DDH, apps like **CareClinic** help track medication, follow-up appointments, and daily activities.
- **Wearable Devices**: Devices like smartwatches (like **Fitbit** or **Apple Watch**) can track your steps, remind you to move, and monitor your activity. This is helpful for people recovering from DDH surgery to make sure they're staying active without overdoing it.

These tools help you keep track of your health and make sure you’re following your doctor’s advice.

**2. How can I use health tracking tools to monitor my condition or recovery progress?**

Tracking your health is important for staying on top of recovery. Here's how you can use health tools:

- **Track Symptoms**: Use a symptom tracker app like **Daylio** or **Pillow** to log how your hip feels each day (pain level, stiffness, etc.). This will help you see patterns and show your doctor how you're doing.
- **Track Movement**: After surgery, you may need to improve your range of motion. Use apps or devices like a **smartwatch** to monitor how much you’re moving, or even track how long you’re walking or standing each day.
- **Monitor Exercise**: After DDH treatment or surgery, certain exercises can be key. Apps like **MyFitnessPal** or **Nike Training Club** can help you follow the exercises recommended by your doctor and make sure you're doing them regularly.
- **Medication and Appointments**: Keep track of when to take your meds or when your next doctor's appointment is. Apps like **Medisafe** can send reminders about medications, while apps like **MyChart** allow you to see appointments and health records.

By using these tools, you can stay on track with your recovery and get help faster if something doesn't seem right.

**Accessing Reliable Information About DDH (Developmental Dysplasia of the Hip):**

1. **Ask Your Doctor or Specialist**: If your child is diagnosed with DDH, your doctor is a great place to start. They can explain the condition and give you up-to-date information about treatments and post-op care. Don't hesitate to ask questions about your child’s specific case.
2. **Trusted Websites**: Some websites are known for providing accurate and helpful health information. Examples include:
   - **Mayo Clinic** ([www.mayoclinic.org](http://www.mayoclinic.org/))
   - **WebMD** ([www.webmd.com](http://www.webmd.com/))
   - **American Academy of Orthopaedic Surgeons** ([www.aaos.org](http://www.aaos.org/)) These sites are regularly updated and reviewed by medical professionals.
3. **Hospitals and Clinics**: Many hospitals have information for patients and families, both in person and online. They can give pamphlets, videos, or websites that explain DDH.
4. **Books and Articles**: Some medical books and journals are written in a way that is easy to understand for parents. Ask your doctor if they recommend any.

**Support Groups and Community Resources for Coping:**

1. **Online Support Groups**: There are many online communities where parents and caregivers share experiences. Examples include:
   - **Facebook Groups**: Search for groups like "DDH Parents Support" or "Hip Dysplasia Awareness."
   - **Forums and Websites**: Websites like "Hip Dysplasia Awareness" or "The DDH Foundation" offer forums where you can connect with others.
2. **Local Support Groups**: Many cities or hospitals have local groups that meet in person. These groups offer emotional support and practical tips from others who understand what you’re going through.
3. **Counselors or Social Workers**: If you feel overwhelmed, a counselor or social worker can help you manage emotions and guide you through the challenges of caring for a child with DDH. They can often connect you with other families or groups.
4. **Charities and Foundations**: Organizations like the **Hip Dysplasia Institute** or **DDH Foundation** offer support, information, and sometimes even financial help for families.

These resources can help you feel more connected, informed, and supported throughout your child’s treatment and recovery process.

**What is the usual cost of treatment for developmental dysplasia of the hip (DDH) in children?**

The cost of treatment for DDH can vary depending on the severity of the condition and the type of treatment needed. In mild cases, treatment might just involve wearing a special harness, which can cost a few hundred dollars. For more severe cases, surgery may be necessary, and that can cost several thousand dollars, especially if the child needs to stay in the hospital for a few days.

For example, if a child needs surgery to fix the hip joint and is hospitalized, the total cost (including surgery, hospital stay, and follow-up visits) could range from $10,000 to $50,000 or more in some countries. However, this price can change based on where you live, the hospital, and the specific care required.

**Are there insurance or subsidy policies to reduce the financial burden?**

Yes, in many places, health insurance can help cover the cost of treatment for DDH. If you have health insurance, it’s important to check whether the procedure, hospital stay, and any required follow-up care are covered. In some cases, insurance companies may cover the entire cost, while in others, they may only cover part of it, leaving you with some out-of-pocket expenses.

There are also government subsidies or programs available in some countries that help with medical costs. For example, in the U.S., Medicaid might help lower the cost if the child’s family meets certain income requirements. Some countries with universal healthcare (like the UK or Canada) might cover the costs through their national healthcare system.

**What social assistance can I apply for if the disease or surgery causes financial hardship?**

If the costs of treating DDH are too high and it’s causing financial hardship, there are several types of social assistance programs you might be able to apply for:

1. **Government Programs**: Many governments offer financial aid for medical treatments, especially for children. For instance, there may be a national health program that will help cover the cost of surgeries or medical equipment like braces.
2. **Charity Assistance**: Some non-profit organizations and charities assist families who are struggling with the costs of medical care. They might offer grants or financial help to pay for treatment or equipment related to DDH.
3. **Hospital Financial Assistance Programs**: Some hospitals offer financial assistance programs to help families who are having trouble paying for treatment. These programs might reduce your bill or allow you to make smaller, more affordable payments.
4. **Insurance Programs**: If you don’t have insurance, some countries provide public health insurance options for families with low income. These programs often cover a wide range of medical treatments, including surgeries for DDH.

The key is to reach out to the hospital, insurance company, or social services in your area to ask about the assistance programs that can help make treatment more affordable. It’s important not to be afraid to ask for help; there are often more options available than you might initially think.

Sure, let's use **developmental dysplasia of the hip (DDH)** in children as an example to answer your questions.

**1. How can I balance mental health, family responsibilities, and work in disease management? What are some practical suggestions?**

**Managing DDH** in children can be a big task. You might feel worried or stressed, but balancing it all is possible with some planning.

- **Take care of your mental health**: It’s important to talk about your feelings. If you're feeling overwhelmed, talking to a counselor or joining a support group can help. Just like we take care of our bodies, we also need to care for our minds.
- **Ask for help with family responsibilities**: You don’t have to do everything alone. Ask family members or friends for help, whether it’s with school runs or household chores. Sharing the load can relieve some stress.
- **Plan your time at work**: If you have a job, try to have flexible hours or even work from home when possible. This way, you can balance doctor’s appointments and your child's needs while still meeting work responsibilities.
- **Set priorities**: Focus on what matters most—your child's health and well-being. Don’t be too hard on yourself if you can’t get everything done. It’s okay to let some things wait.

**2. How might DDH affect future quality of life? How can I plan ahead to improve my health?**

**DDH** is a condition where a child’s hip joint doesn’t form properly. If not treated early, it could lead to problems later, like joint pain or difficulty walking.

- **Future challenges**: Without treatment, a child with DDH might experience pain in the hips as they get older, which could limit their activities. They may also be at risk for arthritis in the future. But with early treatment (like wearing a brace or having surgery), children can grow up to lead normal, active lives.
- **How to improve health**: To help your child live a healthy life despite DDH:
  - Follow the doctor’s advice carefully. If they recommend using a brace or having surgery, make sure it's done on time.
  - Encourage your child to stay active. Exercise can help keep the joints strong and reduce the chances of future problems.
  - Keep regular check-ups with the doctor to catch any issues early.

By planning ahead, you can reduce the chances of problems as your child grows and help them live a healthy, active life.

Both your **mental health** and your child’s **health** are important. Planning and asking for support can make everything more manageable, even with a condition like DDH.

**Reply from Copilot (First time)**

Sure, I'd be happy to help explain Developmental Dysplasia of the Hip (DDH) in simple terms!

**What is Developmental Dysplasia of the Hip (DDH)?**

**DDH** is a condition where a baby's hip joint hasn't formed properly. In a normal hip, the ball at the top of the thigh bone fits snugly into a socket in the pelvis1. In DDH, the ball is loose in the socket and can easily move out of place.

**Main Features and Potential Effects**

- **Main Features**: The hip joint is unstable, and the ball part of the thigh bone doesn't fit well into the socket.
- **Potential Effects**: If not treated, it can lead to problems like limping, walking difficulties, and pain later in life.

**Who is Most Likely to Develop DDH?**

- **Most Likely**: Girls, firstborn children, and babies born in the breech position (feet first).
- **Commonality**: About 1 to 2 babies per 1,000 are born with DDH.

**Long-term Impact on Quality of Life**

- **Long-term Impact**: With early diagnosis and treatment, most children can have normal hip function. However, untreated DDH can lead to long-term issues like arthritis and chronic pain.
- **Aspects to Pay Attention To**: Regular check-ups with a pediatrician, especially if there's a family history of DDH or if the baby was born in the breech position.

Does this help clarify things a bit?

Sure, I'd be happy to help explain Developmental Dysplasia of the Hip (DDH) in simple terms!

**What parts of the body does DDH mainly affect?**

DDH mainly affects the **hip joint**. In a healthy state, the hip joint is a ball-and-socket joint where the ball at the top of the thigh bone (femur) fits snugly into the socket in the pelvis1.

**What do these parts do in a healthy state?**

In a healthy hip joint, the ball and socket fit together tightly, allowing smooth and stable movement of the leg. This stability is crucial for walking, running, and other activities.

**What symptoms or consequences may arise if these parts are damaged?**

If the hip joint is damaged due to DDH, several symptoms and consequences can arise:

- **Pain and discomfort**: The hip may feel unstable or painful, especially when moving.
- **Limping or walking difficulties**: Children might walk with a limp or have trouble walking normally.
- **Uneven leg lengths**: One leg might appear shorter than the other.
- **Limited movement**: The hip joint might not move as freely as it should, making it difficult to bend or rotate the leg.
- **Skin folds**: Uneven skin folds on the thighs can be a sign of DDH.

**Real-life example**

Imagine a child trying to play a game of tag but struggling to keep up because their hip hurts and feels wobbly. They might limp or have trouble running, which can make playing with friends difficult and frustrating.

Does this help clarify things a bit?

Sure! Let's use Developmental Dysplasia of the Hip (DDH) in children as an example.

**What are the main causes of DDH?**

The exact causes of DDH aren't fully understood, but it's likely due to a mix of genetic and environmental factors. Some babies might be born with looser ligaments, making their hip joints less stable2. Other factors include the baby's position in the womb (like being in a breech position) and the mother's hormones during pregnancy.

**What risk factors may increase the chances of developing DDH?**

Several factors can increase the risk of DDH:

- **Family history**: If a close relative had DDH, the baby is more likely to have it.
- **First-born babies**: They have less room to move in the womb, which can affect hip development.
- **Female sex**: Girls are more likely to develop DDH than boys.
- **Breech position**: When the baby is positioned feet-first in the womb, it can put extra stress on the hips.
- **Other orthopedic problems**: Conditions like clubfoot can also increase the risk.

**What lifestyle habits or environmental factors increase the risk of developing DDH?**

- **Swaddling**: Wrapping babies with their legs straight can increase the risk. It's better to swaddle them with their hips bent and legs apart.
- **Limited movement**: Keeping babies in a tight space where they can't move their legs freely can also contribute to DDH.

**How can I prevent it?**

To help prevent DDH, you can:

- **Avoid tight swaddling**: Make sure to swaddle babies with their hips bent and legs apart.
- **Encourage movement**: Let babies move their legs freely to help their hips develop properly.
- **Regular check-ups**: Have your baby checked by a doctor regularly to catch any issues early.

**If I have a family history, is my risk of developing DDH higher?**

Yes, having a family history of DDH increases your baby's risk. If you know there's a history of DDH in your family, it's important to mention this to your doctor so they can monitor your baby more closely.

**What preventive measures should I take?**

- **Regular check-ups**: Ensure your baby gets regular check-ups, especially in the first few months.
- **Inform your doctor**: Let your doctor know about any family history of DDH.
- **Proper swaddling**: Swaddle your baby with their hips bent and legs apart.

I hope this helps! Do you have any more questions about DDH or anything else?

Sure! Let's break it down:

**Main Symptoms of Developmental Dysplasia of the Hip (DDH)**

- **Unequal leg lengths**: One leg might be shorter than the other.
- **Asymmetrical skin folds**: The skin folds on the thighs or buttocks might not match up.
- **Limited movement**: One hip might not move as easily as the other.
- **Limping or waddling gait**: The child might walk with a limp or a "duck-like" walk.

**Signs the Disease is Worsening**

- **Increased limping**: The limp gets worse or doesn't improve.
- **Pain**: The child starts to feel pain in the hip or leg.
- **Decreased mobility**: The child finds it harder to move the affected leg.
- **Visible deformity**: The hip joint might look out of place.

**When to Seek Immediate Medical Attention**

- **Sudden pain**: If the child suddenly feels pain in the hip or leg.
- **Severe limping**: If the limp becomes severe or the child can't walk.
- **Visible deformity**: If the hip joint looks out of place or swollen.
- **Any new symptoms**: If you notice any new symptoms or changes in the child's condition.

Does this help clarify things?

Sure! Let's break it down step by step:

**1. How do doctors confirm the diagnosis of Developmental Dysplasia of the Hip (DDH)?**

Doctors use a few methods to diagnose DDH:

- **Physical Examination**: They check the baby's hips using special tests called the **Barlow and Ortolani maneuvers**. These tests help see if the hip joint is loose or unstable1.
- **Ultrasound**: For babies younger than 6 months, doctors use an ultrasound to get a clear picture of the hip joint.
- **X-rays**: For older babies and children, X-rays help see the bones and check for any abnormalities.

**2. What indicators are important in test results?**

- **Barlow and Ortolani Tests**: If the hip "clunks" during these tests, it might indicate instability.
- **Ultrasound**: It shows how well the hip joint is formed and if the femoral head is properly seated in the acetabulum (hip socket).
- **X-rays**: They show the shape of the hip joint and any misalignment or dislocation.

**3. Do I need regular follow-ups after diagnosis?**

Yes, regular follow-ups are important to monitor the baby's hip development and ensure the treatment is working. The frequency of follow-ups depends on the severity of DDH and the treatment plan:

- **For Mild Cases**: Follow-ups might be every few months to check if the hip is developing normally.
- **For Severe Cases**: Follow-ups might be more frequent, especially if the baby is using a brace or has had surgery.

During follow-ups, doctors will check the baby's hip stability, movement, and overall development to make sure everything is progressing well.

Does this help clarify things?

Sure, I'd be happy to help explain this in simple terms!

**Symptoms Indicating Immediate Medical Attention for DDH**

If your child has **Developmental Dysplasia of the Hip (DDH)**, you should seek immediate medical attention if you notice any of these symptoms:

1. **Uneven leg lengths**: One leg appears shorter than the other.
2. **Uneven skin folds**: The skin folds on the thighs or buttocks are not symmetrical.
3. **Limited movement**: One leg doesn't move as freely or widely as the other.
4. **Limping or waddling gait**: Your child walks with a limp or a waddling motion.
5. **Pain or discomfort**: If your child seems to be in pain or is uncomfortable when moving.

**Handling and Self-Monitoring During an Emergency Situation**

If you suspect your child has DDH and needs immediate medical attention, here's what you can do:

1. **Stay calm**: Keep yourself and your child calm to avoid further stress.
2. **Avoid unnecessary movement**: Try not to move your child's hip or leg too much.
3. **Keep the hip in a safe position**: Gently support the affected hip in a comfortable position.
4. **Contact a healthcare professional**: Call your pediatrician or go to the nearest emergency room for professional medical help.
5. **Follow medical advice**: Once you reach a healthcare professional, follow their instructions carefully.

**Real-Life Example**

Imagine you're at a park and notice your 6-month-old baby has one leg that seems shorter and doesn't move as much as the other. You also see that the skin folds on their thighs are uneven. You should take your baby to the doctor right away to get it checked out.

Does this help clarify things?

Sure! Let's break it down step by step:

**Treatments During Hospitalization**

1. **Pavlik Harness**: A special brace that keeps the baby's hip in the correct position. It helps the hip joint develop properly.
2. **Surgery**: If the hip is severely dislocated, a doctor might perform surgery to put the hip back in place.

**Preventing Infections or Complications**

1. **Keep the Area Clean**: Make sure the harness or surgical area is clean to avoid infections.
2. **Follow Doctor's Instructions**: Follow all care instructions given by the healthcare team.
3. **Hand Hygiene**: Wash hands frequently to prevent the spread of germs.

**Helping with Recovery After Discharge**

1. **Physical Therapy**: Helps strengthen the muscles around the hip and improve movement.
2. **Regular Check-ups**: Keep visiting the doctor to monitor the hip's progress.
3. **Encouragement**: Support and encourage the child to move and play as recommended by the doctor.

Does this help clarify things?

Sure! Let's break it down:

**Common Medications for Developmental Dysplasia of the Hip (DDH)**

For children with DDH, the most common treatment is **bracing** rather than medication. The **Pavlik Harness** is often used for newborns and young infants to keep the hip joint in place while it develops properly. In more severe cases, surgery might be needed2.

**How to Take Medication Correctly**

Since DDH treatment mainly involves bracing, it's important to follow the doctor's instructions on how long and when to wear the brace. For example, a baby might need to wear the Pavlik Harness full-time for 6-12 weeks, and then part-time for another 4-6 weeks1.

**What to Do If You Forget to Take Your Medication or Wear the Brace**

If you forget to wear the brace or take medication, try to put it on as soon as you remember. If it's close to the next scheduled time, it's usually okay to wait. However, if you forget often, it's best to talk to your doctor for advice.

**Foods to Avoid While Taking Medication for DDH**

There aren't specific foods to avoid for DDH treatment since it's mainly about bracing and sometimes surgery. However, maintaining a healthy diet is always important for overall health and recovery.

**Common Side Effects**

For bracing, some babies might experience skin irritation or discomfort. If surgery is needed, common side effects might include pain, swelling, and infection2. Always consult your doctor if you notice any unusual symptoms.

Does this help clarify things?

Sure, I'd be happy to help explain Developmental Dysplasia of the Hip (DDH) in simple terms!

**What types of pain may be caused by DDH or its treatment? Where is the pain usually felt?**

DDH can cause pain in the hip or groin area. If untreated, it can lead to arthritis and more severe pain later in life1. Treatment, like surgery, can also cause pain around the hip.

**What methods can help relieve pain? What are the recommended pharmacological and non-pharmacological treatments?**

To relieve pain from DDH, you can use:

- **Pharmacological treatments**: Painkillers like acetaminophen or ibuprofen.
- **Non-pharmacological treatments**: Physical therapy, exercises to strengthen muscles, and using supportive devices like braces or crutches.

**What steps should I take if the pain is more than expected or worsens? Do I need to contact my doctor to adjust my treatment plan?**

If the pain is worse than expected or gets worse, you should contact your doctor. They might need to adjust your treatment plan, which could include changing medications, adding physical therapy, or considering other interventions.

Does this help clarify things?

Sure, I'd be happy to help explain everything in simple terms! Let's use Developmental Dysplasia of the Hip (DDH) in children as an example.

**Post-Surgery Care Precautions**

After surgery, it's important to follow these precautions:

- **Keep the hip area clean and dry** to prevent infection.
- **Avoid putting weight on the hip** until your doctor says it's okay.
- **Use crutches or a walker** if needed to help with walking.
- **Follow your doctor's instructions** for any medications or physical therapy.

**Returning to Normal Daily Activities**

You can usually start doing light activities within **2-3 weeks** after surgery, but it might take **6-12 months** to fully recover and return to all normal activities.

**Resuming Activities**

You can start gentle activities like walking and light stretching **2-3 weeks** after surgery. More strenuous activities should be resumed gradually, following your doctor's advice.

**Recommended Activities During Recovery**

During recovery, it's good to do:

- **Gentle walking** to improve circulation.
- **Physical therapy exercises** to strengthen muscles.
- **Swimming** (once your doctor gives the okay) to help with mobility without putting too much strain on the hip.

**Scheduling Exercise**

Start with **short sessions** of exercise, about **10-15 minutes**, 2-3 times a day. Gradually increase the duration and intensity as you feel stronger and your doctor approves.

**Stages During Postoperative Recovery**

1. **Immediate Post-Surgery (0-2 weeks)**: Focus on healing and gentle movements.
2. **Early Recovery (2-6 weeks)**: Start light activities and physical therapy.
3. **Intermediate Recovery (6-12 weeks)**: Increase activity levels gradually.
4. **Long-Term Recovery (3-12 months)**: Continue strengthening exercises and return to normal activities.

**Precautions in Each Stage**

- **Immediate Post-Surgery**: Avoid putting weight on the hip, keep it clean.
- **Early Recovery**: Follow physical therapy routines, avoid heavy lifting.
- **Intermediate Recovery**: Gradually increase activity, listen to your body.
- **Long-Term Recovery**: Continue exercises, avoid overexertion.

**Specialized Rehabilitation Plan**

Yes, a specialized rehabilitation plan is important. It helps tailor exercises to your specific needs, ensuring a safe and effective recovery.

**Preventing Recurrence**

To prevent recurrence, follow your doctor's advice, continue with prescribed exercises, and attend regular check-ups.

**Possible Complications**

Complications can include infection, blood clots, or issues with the hip joint. If you notice unusual pain, swelling, or redness, contact your doctor immediately.

I hope this helps! Do you have any specific questions or need more details on any part?

Sure! Let's break it down step by step:

**Foods Helpful in Recovery from DDH or During Post-Surgery Recovery**

- **Fruits and Vegetables**: These are packed with vitamins and minerals that help your body heal. Think of colorful fruits like berries and oranges, and leafy greens like spinach and kale.
- **Lean Proteins**: Foods like chicken, fish, beans, and tofu help repair tissues and build muscle.
- **Whole Grains**: Foods like brown rice, quinoa, and whole wheat bread provide energy and keep you full longer.
- **Healthy Fats**: Avocados, nuts, and olive oil are good for your joints and overall health.

**Foods That May Aggravate DDH**

- **Processed Foods**: These often contain unhealthy fats and sugars that can slow down healing.
- **Sugary Drinks**: Drinks like soda and sweetened juices can lead to weight gain and inflammation.
- **Excess Salt**: Too much salt can cause water retention and swelling.

**Adjusting Your Diet to Prevent Constipation**

- **Drink Plenty of Water**: Aim for at least 8 glasses a day.
- **Eat Fiber-Rich Foods**: Include fruits, vegetables, whole grains, and beans in your diet.
- **Limit Processed Foods**: These can be low in fiber and hard to digest.

**Exercises During the Recovery Phase**

- **Gentle Stretching**: Helps maintain flexibility and reduce stiffness.
- **Walking**: A low-impact exercise that keeps your joints moving.
- **Physical Therapy Exercises**: Specific exercises recommended by your therapist to strengthen muscles around the hip.

**Precautions When Exercising, Especially During Postoperative Recovery**

- **Listen to Your Body**: Stop if you feel pain.
- **Follow Your Doctor's Advice**: Stick to the exercise plan given by your healthcare provider.
- **Use Supportive Gear**: Wear appropriate footwear and use any recommended braces or supports.

**Does DDH Affect Sleep Quality?**

- **Yes**: Pain and discomfort from DDH can make it hard to sleep well.
- **Improving Sleep**:
  - **Comfortable Bedding**: Use a supportive mattress and pillows.
  - **Relaxation Techniques**: Try deep breathing, meditation, or gentle yoga before bed.
  - **Consistent Sleep Schedule**: Go to bed and wake up at the same time every day.

Sure, I'd be happy to help explain this in simple terms!

**Psychological Problems Associated with DDH or Postoperative Recovery**

Children with Developmental Dysplasia of the Hip (DDH) and their families might face **anxiety** and **depression**. This can happen because DDH often requires treatments like surgery, which can be scary and stressful1. Post-surgery, both children and parents might feel overwhelmed by the recovery process, pain, and changes in daily life.

**How to Deal with Them Positively**

1. **Stay Informed**: Knowing what to expect can reduce fear. Talk to your doctor about the treatment and recovery process.
2. **Seek Support**: Join support groups or online forums where you can share experiences and get advice from others in similar situations.
3. **Practice Self-Care**: Take time for yourself. Do activities you enjoy and make sure to rest.
4. **Stay Positive**: Focus on small improvements and celebrate progress, no matter how small.
5. **Professional Help**: Don't hesitate to seek help from a therapist or counselor if you're feeling overwhelmed.

**Signs You Need Psychological Support**

- Feeling sad or hopeless most of the time
- Losing interest in activities you used to enjoy
- Having trouble sleeping or sleeping too much
- Feeling anxious or worried a lot
- Experiencing changes in appetite or weight
- Having trouble concentrating or making decisions

**Finding Professional Help or Support Resources**

1. **Talk to Your Doctor**: They can refer you to a mental health professional.
2. **Look for Local Support Groups**: Hospitals or clinics often have support groups for families dealing with DDH.
3. **Online Resources**: Websites like the American Psychological Association (APA) can help you find therapists in your area.
4. **School Counselors**: If your child is school-aged, school counselors can provide support and resources.

Remember, it's okay to ask for help. Taking care of your mental health is just as important as taking care of your physical health. Do you have any specific concerns or questions about DDH?

Sure! Let's break it down simply:

**Recommended Health Apps for Managing DDH or Postoperative Recovery**

1. **MyFitnessPal**: Helps track nutrition and exercise, which is important for overall health and recovery.
2. **Medisafe**: Reminds you to take medications on time, ensuring you follow your treatment plan.
3. **Headspace**: Offers meditation and mindfulness exercises to help manage stress and improve mental well-being.
4. **Recovery Record**: Tracks your recovery progress, sets goals, and provides motivational messages.
5. **PT Pal**: Provides physical therapy exercises to help with mobility and strength.

**How to Use Health Tracking Tools**

1. **Track Your Progress**: Use apps like Recovery Record to log your daily activities, pain levels, and any symptoms you experience.
2. **Set Reminders**: Apps like Medisafe can remind you to take your medications and complete your exercises.
3. **Monitor Your Health**: Use wearable devices to track your heart rate, steps, and sleep patterns. This data can help you and your doctor understand how well you're recovering.
4. **Stay Connected**: Use apps like MyChart to communicate with your healthcare team, ask questions, and get advice.

By using these tools, you can stay on top of your health and recovery, making it easier to manage DDH or postoperative care. Does this help clarify things?

Sure! Let's talk about Developmental Dysplasia of the Hip (DDH) in children and how to find reliable information, support groups, and community resources.

**Reliable Information about DDH**

1. **Pediatrician**: Your child's doctor is a great first source of information. They can explain DDH, its symptoms, and treatment options.
2. **Children's Hospitals**: Websites like the Children's Hospital of Philadelphia (CHOP) provide detailed information about DDH.
3. **American Academy of Pediatrics (AAP)**: They offer guidelines and resources for parents.
4. **Medical Websites**: Trusted sites like Mayo Clinic, WebMD, and Orthobullets have reliable information on DDH.

**Post-Operative Care for DDH**

1. **Follow-Up Appointments**: Regular visits to your child's doctor to monitor recovery.
2. **Physical Therapy**: Helps improve strength and mobility.
3. **Home Care Instructions**: Follow the doctor's advice on activities, exercises, and any necessary braces or supports.

**Support Groups and Community Resources**

1. **Online Support Groups**: Websites like Facebook and Reddit have communities where parents share experiences and advice.
2. **Local Hospitals**: Some hospitals have support groups for families dealing with DDH.
3. **Non-Profit Organizations**: Organizations like the International Hip Dysplasia Institute (IHDI) offer resources and support.

Would you like more details on any of these points?

Sure, I'd be happy to help explain this in simple terms!

**Developmental Dysplasia of the Hip (DDH) in Children**

**DDH** is a condition where a baby's hip joint hasn't formed properly. Imagine the hip joint as a ball-and-socket: in DDH, the ball (femur) doesn't fit snugly into the socket (acetabulum), making the joint unstable1.

**Treatment Costs**

The cost of treating DDH can vary depending on how early it's detected and the severity of the condition. Here's a simple breakdown:

- **Early Detection**: If DDH is found early (usually within the first few months), treatment with a **Pavlik harness** (a special brace) can cost around **$601**.
- **Late Detection**: If DDH is found later, surgery might be needed, which can cost around **$4,352** per hip. In more complex cases, the cost can go up to **$7,052**.

**Insurance and Subsidies**

Many health insurance plans cover the treatment of DDH, especially if it's considered a medical necessity. It's important to check with your insurance provider to understand what's covered. Some countries or regions may also offer subsidies or financial assistance programs to help with the costs.

**Social Assistance**

If the treatment or surgery for DDH causes financial hardship, you might be eligible for social assistance programs. These can include:

- **Government Health Programs**: Some countries have government-funded health programs that provide financial support for medical treatments.
- **Non-Profit Organizations**: There are organizations that offer grants or financial aid to families dealing with medical conditions like DDH.
- **Community Support**: Local community centers or charities might offer assistance with medical expenses or provide resources for additional support.

I hope this helps! Do you have any more questions about DDH or anything else?

Sure, I'd be happy to help! Let's break it down step by step.

**Balancing Mental Health, Family Responsibilities, and Work in Disease Management**

Managing a condition like Developmental Dysplasia of the Hip (DDH) in children can be challenging. Here are some practical suggestions:

1. **Create a Routine**: Establish a daily routine that includes time for medical appointments, family activities, and work. This helps in managing time effectively and reduces stress.
2. **Seek Support**: Don't hesitate to ask for help from family, friends, or support groups. Sharing responsibilities can lighten the load.
3. **Set Realistic Goals**: Break tasks into smaller, manageable goals. This makes it easier to achieve them without feeling overwhelmed.
4. **Take Breaks**: Make sure to take short breaks throughout the day to relax and recharge. This can help in maintaining mental health.
5. **Communicate**: Keep open lines of communication with your employer about your child's condition and any necessary accommodations.
6. **Self-Care**: Prioritize your own mental and physical health. Take time for yourself to relax and unwind.

**Impact of DDH on Future Quality of Life**

DDH can affect a child's future quality of life in several ways:

1. **Mobility Issues**: Children with DDH may experience difficulties in walking or running, which can impact their physical activities and social interactions.
2. **Pain and Discomfort**: Untreated DDH can lead to chronic pain and discomfort, affecting daily activities and overall well-being.
3. **Arthritis**: There is a higher risk of developing arthritis in the affected hip, which can further limit mobility and cause pain.
4. **Surgical Interventions**: Some children may require surgery to correct the hip joint, which can have long-term implications on their health.

**Planning Ahead to Improve Health**

To improve the health and quality of life for a child with DDH, consider the following steps:

1. **Regular Check-Ups**: Schedule regular visits with a pediatric orthopedic specialist to monitor the child's hip development and address any issues early on.
2. **Physical Therapy**: Engage in physical therapy exercises to strengthen the muscles around the hip and improve mobility.
3. **Healthy Lifestyle**: Encourage a healthy diet and regular physical activity to support overall health and well-being.
4. **Education**: Educate yourself and your child about DDH, its treatment options, and ways to manage it effectively.
5. **Support Networks**: Connect with other families dealing with DDH to share experiences and gain emotional support.

By following these suggestions, you can better manage DDH, support your child's health, and maintain a balanced life. Does this help clarify things?

**Reply from Copilot (Second time)**

Sure, I'd be happy to help explain Developmental Dysplasia of the Hip (DDH) in simple terms!

**What is Developmental Dysplasia of the Hip (DDH)?**

DDH is a condition where a baby's hip joint hasn't formed properly. In a normal hip, the ball at the top of the thigh bone fits snugly into a socket in the pelvis1. In DDH, the ball is loose in the socket and can easily move out of place.

**Main Features and Potential Effects**

- **Main Features**: The hip joint is unstable and can be partially or completely dislocated1.
- **Potential Effects**: If not treated, it can lead to problems with walking, pain, and arthritis later in life1.

**Who is Most Likely to Develop DDH?**

- **Most Likely**: Girls, firstborn children, and babies born in the breech position (feet first)1.
- **Commonality**: About 1 in 1,000 babies are born with DDH.

**Long-term Impact on Quality of Life**

- **Long-term Impact**: With early treatment, most children can have normal hip function1. Without treatment, it can lead to long-term issues like limping, pain, and difficulty walking1.
- **Aspects to Pay Attention To**: Regular check-ups with a pediatrician, especially if there's a family history of DDH or if the baby was in the breech position.

Does this help clarify things a bit?

Sure, I'd be happy to help explain Developmental Dysplasia of the Hip (DDH) in simple terms!

**What parts of the body does DDH mainly affect?**

DDH mainly affects the **hip joint**. The hip joint is where the thigh bone (femur) meets the pelvis1.

**What do these parts do in a healthy state?**

In a healthy hip joint, the ball-shaped head of the femur fits snugly into a socket (acetabulum) in the pelvis. This allows for smooth movement and stability when walking, running, and other activities1.

**What symptoms or consequences may arise if these parts are damaged?**

If the hip joint is affected by DDH, the ball may not fit properly into the socket. This can lead to:

- **Hip instability**: The hip joint may feel loose and can easily move out of place.
- **Pain**: There might be discomfort or pain in the hip, especially when walking or moving.
- **Limping**: A child might walk with a limp or have an unusual gait.
- **Leg length discrepancy**: One leg might appear shorter than the other.
- **Limited movement**: The range of motion in the hip might be restricted.

**Specific examples**

- **Example 1**: A baby with DDH might have one leg that seems shorter than the other. This can be noticed when dressing the baby or looking at their legs while they're lying down.
- **Example 2**: A toddler with DDH might limp or have trouble walking normally. They might also avoid putting weight on one leg because it feels unstable or painful.

I hope this helps! Do you have any more questions about DDH or anything else?

Sure! Let's break it down:

**What are the main causes of Developmental Dysplasia of the Hip (DDH)?**

DDH happens when a baby's hip joint doesn't form properly. The exact cause isn't known, but it's likely due to a mix of genetic and environmental factors2. Some possible causes include:

- **Genetics**: If a parent or sibling had DDH, the baby is more likely to have it too.
- **Breech position**: When a baby is in a feet-first position in the womb, it can affect hip development.
- **Tight uterus**: Limited space in the womb can restrict fetal movement, impacting hip joint formation.

**What risk factors may increase the chances of developing DDH?**

Some factors that can increase the risk of DDH include:

- **Family history**: Having a close relative with DDH.
- **First-born babies**: Limited space in the uterus for the first baby.
- **Female sex**: Girls are more likely to develop DDH than boys.
- **Other orthopedic problems**: Conditions like clubfoot can be associated with DDH.

**What lifestyle habits or environmental factors increase the risk of developing DDH?**

- **Swaddling**: Wrapping babies with their legs straight can increase the risk.
- **Infant positioning**: Keeping babies in positions that restrict hip movement can contribute to DDH.

**How can I prevent it?**

To reduce the risk of DDH:

- **Avoid tight swaddling**: Keep babies' legs in a natural position.
- **Regular check-ups**: Early detection through physical exams and ultrasounds can help.
- **Encourage movement**: Allow babies to move their legs freely.

**If I have a family history, is my risk of developing DDH higher?**

Yes, having a family history of DDH increases your risk.

**What preventive measures should I take?**

- **Regular screenings**: Have your baby checked regularly by a healthcare provider.
- **Be aware of symptoms**: Look out for signs like uneven skin folds or a leg that appears shorter.
- **Consult a specialist**: If there's a family history, consult a pediatric orthopedic specialist.

I hope this helps! Do you have any more questions about DDH?

Sure! Let's talk about Developmental Dysplasia of the Hip (DDH) in children.

**Main Symptoms of DDH:**

1. **Uneven leg lengths**: One leg might look shorter than the other.
2. **Uneven skin folds**: The skin folds on the thighs might not match up.
3. **Limited movement**: The affected hip might not move as easily or as far as the other side.
4. **Limping or waddling**: The child might walk with a limp or a waddling gait.

**Signs the Disease is Worsening:**

1. **Increased limping**: If the child starts limping more or it gets worse.
2. **Pain**: Any pain in the hip or leg, especially when moving.
3. **Decreased mobility**: If the child's ability to move the hip decreases further.
4. **Visible deformity**: Any noticeable changes in the shape of the hip or leg.

**When to Seek Immediate Medical Attention:**

- **Sudden pain**: If the child suddenly feels pain in the hip or leg.
- **Severe limping**: If the limping becomes severe or the child can't walk at all.
- **Swelling or bruising**: Any swelling or bruising around the hip area.

Would you like to know more about how DDH is diagnosed or treated?

Sure, I'd be happy to help explain Developmental Dysplasia of the Hip (DDH) in simple terms!

**Diagnosis Methods**

Doctors use a few methods to confirm DDH:

1. **Physical Exam**: They check the baby's hips using two special tests called the **Ortolani** and **Barlow** maneuvers. These tests help see if the hip joint is loose or unstable1.
2. **Ultrasound**: This imaging test is used for babies younger than 6 months. It helps get a clear picture of the hip joint2.
3. **X-ray**: For older babies, an X-ray can show how the hip joint is developing.

**Important Indicators in Test Results**

- **Ortolani Test**: If the hip "clunks" back into place, it suggests instability.
- **Barlow Test**: If the hip can be pushed out of place, it indicates a problem.
- **Ultrasound/X-ray**: These show the shape and position of the hip joint.

**Follow-Up Schedule**

Yes, regular follow-ups are important! Here's what to expect:

- **First Few Months**: Frequent check-ups to monitor the hip's development.
- **Every 3-6 Months**: Regular visits to ensure the hip is developing normally.
- **As Needed**: If there are any concerns or changes, additional tests might be needed.

Does this help clarify things?

Sure, I'd be happy to help explain DDH in simple terms!

**Symptoms Indicating Immediate Medical Attention**

If your child has **Developmental Dysplasia of the Hip (DDH)**, you should seek immediate medical attention if you notice:

- **One leg shorter than the other**.
- **Uneven skin folds** on the thighs.
- **Less movement or flexibility** on one side.
- **Limping, toe walking**, or a **waddling gait**.

**Handling and Self-Monitoring During an Emergency**

If you suspect DDH and it's an emergency, here's what you can do:

1. **Keep calm**: Stay calm to think clearly and act quickly.
2. **Avoid moving the hip**: Do not try to move or adjust the hip yourself.
3. **Call for help**: Contact emergency services or take your child to the nearest hospital.
4. **Provide comfort**: Keep your child comfortable and reassured while waiting for help.

Would you like more information on DDH or any other topic?

Sure! Let's break it down step by step:

**Treatments During Hospitalization**

1. **Pavlik Harness**: This is a special brace that keeps the baby's hips in the right position to help the hip joint develop properly. It's like a soft, adjustable seatbelt for the hips.
2. **Surgery**: In more severe cases, doctors might need to perform surgery to move the hip joint into the correct position1. Think of it as fixing a puzzle piece that's not fitting right.

**Preventing Infections or Complications**

1. **Keep Everything Clean**: Make sure that anyone who touches the baby's hips or the harness has clean hands. It's like washing your hands before eating.
2. **Follow Doctor's Instructions**: Make sure to follow all the care instructions given by the doctors and nurses. It's like following a recipe to make sure the food turns out right.
3. **Watch for Signs of Infection**: Look out for redness, swelling, or unusual pain around the hip area. If you notice anything, tell the doctor right away.

**Reinforcing Treatment After Discharge**

1. **Regular Check-ups**: Keep going to the doctor for regular check-ups to make sure the hip is developing well. It's like going to the mechanic to make sure your car is running smoothly.
2. **Physical Therapy**: Some kids might need physical therapy to help strengthen their muscles and improve movement. It's like going to the gym to get stronger.
3. **Safe Handling**: Make sure to handle the baby gently and avoid putting pressure on the hips. It's like being extra careful with a delicate vase.

Does this help clarify things?

Sure, I'd be happy to help explain things simply!

**Common Medications for Developmental Dysplasia of the Hip (DDH)**

For children with DDH, the most common treatment is **bracing**, especially using a **Pavlik Harness**. This brace helps keep the hip joint in place while it develops properly1. In some cases, **medications** like pain relievers (e.g., acetaminophen or ibuprofen) might be used to manage discomfort.

**How to Take Medications Correctly**

- **Pain Relievers**: Follow the dosage instructions on the label or as prescribed by your doctor. Usually, it's taken with food to avoid stomach upset.
- **Bracing**: Ensure the harness is worn as directed by your doctor, usually full-time for several weeks, then part-time.

**What to Do If You Forget to Take Medication or Take It at the Wrong Time**

If you forget to take a dose, take it as soon as you remember unless it's almost time for the next dose. If it's almost time for the next dose, skip the missed dose and continue with your regular schedule. Do not double the dose to make up for the missed one.

**Foods to Avoid While Taking Medication**

While there are no specific foods to avoid with DDH treatments, it's always good to avoid foods that might upset the stomach, especially if taking pain relievers.

**Common Side Effects**

- **Pain Relievers**: Stomach upset, nausea, or dizziness.
- **Bracing**: Skin irritation or discomfort from wearing the harness.

Does this help clarify things?

Sure! Let's break it down:

**What types of pain may be caused by DDH or its treatment? Where is the pain usually felt?**

- **Pain from DDH**: Children with DDH might feel pain in their hip or groin area. This pain can be due to the hip joint being loose or partially dislocated.
- **Pain from treatment**: Treatments like surgery or using a brace (like the Pavlik harness) can cause discomfort or pain in the hip area.

**What methods can help relieve pain? What are the recommended treatments?**

- **Non-pharmacological treatments**: Gentle exercises, physical therapy, and using supportive devices like braces can help manage pain.
- **Pharmacological treatments**: Pain relievers like acetaminophen or ibuprofen can be used to reduce pain and inflammation.

**What steps should I take if the pain is more than expected or worsens?**

- **Contact your doctor**: If the pain is more than expected or gets worse, it's important to contact your doctor. They might need to adjust your treatment plan or check for any complications.

Does this help clarify things?

Sure, I'd be happy to help explain everything in simple terms! Let's use Developmental Dysplasia of the Hip (DDH) in children as an example.

**Post-Surgery Care Precautions**

After surgery, it's important to follow these precautions:

- **Keep the hip area clean and dry** to prevent infection.
- **Avoid putting weight on the hip** until your doctor says it's okay.
- **Use crutches or a walker** if needed to help with walking.
- **Follow your doctor's instructions** for any medications or physical therapy.

**Returning to Normal Daily Activities**

You can usually start doing light activities within **2-3 weeks** after surgery, but it might take **6-12 months** to fully recover and return to all normal activities.

**Resuming Activities**

You can start gentle activities like walking and light stretching **within a few days** after surgery. More strenuous activities should be resumed gradually, following your doctor's advice.

**Recommended Activities During Recovery**

- **Walking**: Start with short walks and gradually increase the distance.
- **Physical Therapy Exercises**: Gentle stretching and strengthening exercises.
- **Swimming**: A great low-impact exercise to improve mobility and strength.

**Scheduling Exercise During Postoperative Recovery**

- **Start with light exercises** as soon as your doctor allows.
- **Gradually increase the intensity** and duration of your exercises.
- **Listen to your body** and rest if you feel pain or discomfort.

**Stages During Postoperative Recovery**

1. **Immediate Post-Surgery**: Focus on healing and preventing infection.
2. **Early Recovery (2-3 weeks)**: Gentle activities and physical therapy.
3. **Intermediate Recovery (3-6 weeks)**: Gradual increase in activities.
4. **Late Recovery (6-12 months)**: Return to normal activities and sports.

**Precautions in Each Stage**

- **Immediate Post-Surgery**: Keep the area clean, avoid weight-bearing.
- **Early Recovery**: Follow physical therapy exercises, avoid heavy lifting.
- **Intermediate Recovery**: Gradually increase activity, avoid overexertion.
- **Late Recovery**: Continue strengthening exercises, avoid high-impact activities.

**Specialized Rehabilitation Plan**

Yes, a specialized rehabilitation plan is important. It helps tailor exercises to your specific needs, ensuring a safe and effective recovery.

**Preventing Recurrence**

To prevent recurrence, follow your doctor's advice, continue with prescribed exercises, and attend regular follow-up appointments.

**Possible Complications and Steps to Take**

Complications can include infection, blood clots, or issues with the hip joint. If you notice any unusual pain, swelling, or redness, contact your doctor immediately.

I hope this helps! Do you have any specific questions or need more details on any of these points?

Sure! Let's break it down step by step:

**Foods Helpful in Recovery from DDH or During Post-Surgery Recovery**

- **Fruits and Vegetables**: These are packed with vitamins and minerals that help your body heal. Think of colorful fruits like berries and oranges, and leafy greens like spinach and kale.
- **Lean Proteins**: Foods like chicken, fish, beans, and tofu help repair tissues and build muscle.
- **Whole Grains**: Foods like brown rice, oatmeal, and whole-wheat bread provide energy and keep you full longer.
- **Healthy Fats**: Avocados, nuts, and olive oil provide essential fatty acids that support overall health.

**Foods That May Aggravate DDH**

- **Processed Foods**: These often contain unhealthy fats and sugars that can slow down healing.
- **Sugary Drinks**: Drinks like soda and sweetened juices can lead to weight gain and inflammation.
- **Excessive Salt**: Too much salt can cause water retention and swelling.

**Adjusting Your Diet to Prevent Constipation**

- **Stay Hydrated**: Drink plenty of water throughout the day.
- **Eat Fiber-Rich Foods**: Include fruits, vegetables, whole grains, and legumes in your diet.
- **Limit Processed Foods**: These can be low in fiber and contribute to constipation.

**Exercises During the Recovery Phase**

- **Gentle Stretching**: Helps maintain flexibility and reduce stiffness.
- **Walking**: A low-impact exercise that can help improve circulation and muscle strength.
- **Physical Therapy Exercises**: Specific exercises prescribed by a therapist to strengthen the hip muscles.

**Precautions When Exercising, Especially During Postoperative Recovery**

- **Follow Doctor's Advice**: Always adhere to the exercise plan given by your healthcare provider.
- **Avoid High-Impact Activities**: Steer clear of activities that put too much stress on your hip, like running or jumping.
- **Listen to Your Body**: If you feel pain, stop and consult your doctor.

**Does DDH Affect Sleep Quality?**

- **Yes, it can**: Pain and discomfort from DDH can make it hard to sleep well.
- **Ways to Improve Sleep**:
  - **Comfortable Sleeping Position**: Use pillows to support your hip.
  - **Relaxation Techniques**: Try deep breathing or meditation before bed.
  - **Consistent Sleep Schedule**: Go to bed and wake up at the same time every day.

I hope this helps! Do you have any more questions or need further clarification on any of these points?

Sure, I'd be happy to help explain this in simple terms!

**Psychological Problems Associated with DDH or Postoperative Recovery**

Children with Developmental Dysplasia of the Hip (DDH) and their families might face **anxiety** and **depression**. This can happen because:

- **Anxiety**: Parents might worry about their child's health, treatment, and future.
- **Depression**: Both children and parents might feel sad or hopeless due to the challenges of managing DDH and the recovery process after surgery2.

**How to Deal with Them Positively**

1. **Stay Informed**: Learn about DDH and the recovery process. Knowledge can reduce fear of the unknown.
2. **Seek Support**: Talk to friends, family, or join support groups for parents of children with DDH.
3. **Professional Help**: Consider seeing a therapist or counselor who can provide strategies to manage anxiety and depression.
4. **Self-Care**: Take time for yourself. Do activities you enjoy and take care of your own health.

**Signs You Need Psychological Support**

You might need psychological support if you or your child:

- Feel very sad or hopeless for a long time.
- Have trouble sleeping or sleeping too much.
- Lose interest in activities you used to enjoy.
- Feel anxious or worried a lot.
- Have trouble concentrating or making decisions.

**Finding Professional Help or Support Resources**

1. **Talk to Your Doctor**: Your pediatrician or surgeon can refer you to a mental health professional.
2. **Online Resources**: Websites like the American Psychological Association (APA) can help you find therapists.
3. **Local Support Groups**: Look for local support groups for families dealing with DDH.
4. **School Counselors**: If your child is school-aged, school counselors can provide support and resources.

Sure! Let's break it down simply:

**Recommended Health Apps for Managing DDH or Postoperative Recovery**

1. **MyFitnessPal**: Helps track nutrition and exercise, which is important for overall health and recovery.
2. **Medisafe**: Reminds you to take medications on time, ensuring you follow your treatment plan.
3. **Headspace**: Offers meditation and mindfulness exercises to help manage stress and improve mental well-being.
4. **Recovery Record**: Allows you to track your recovery progress, set goals, and receive motivational messages.
5. **PT Pal**: Provides physical therapy exercises and routines to help with mobility and strength.

**Using Health Tracking Tools to Monitor Your Condition or Recovery Progress**

1. **Use Apps to Track Symptoms**: Apps like **PainTracker** can help you log pain levels, which is useful for your doctor to adjust your treatment.
2. **Set Reminders**: Apps like **Pill Reminder** can remind you to take your medications and follow your care plan.
3. **Monitor Physical Activity**: Use fitness trackers to monitor your activity levels and ensure you're staying active as recommended.
4. **Stay Connected with Healthcare Providers**: Apps like **MyChart** allow you to communicate with your doctors and access your medical records easily.
5. **Track Wound Healing**: Apps like **Recovery Record** can help you monitor wound healing and track any signs of infection.

By using these tools, you can stay on top of your health and recovery, making it easier to manage DDH or postoperative care. Does this help clarify things?

Sure! Let's talk about Developmental Dysplasia of the Hip (DDH) in children and how to find reliable information, support groups, and community resources.

**Reliable Information about DDH**

1. **Pediatrician**: Your child's doctor is a great first source of information. They can explain DDH, its symptoms, and treatment options.
2. **Children's Hospitals**: Websites like the Children's Hospital of Philadelphia (CHOP) provide detailed information about DDH.
3. **American Academy of Pediatrics (AAP)**: They offer guidelines and resources for parents.
4. **Medical Websites**: Trusted sites like Mayo Clinic, WebMD, and Orthobullets have reliable information on DDH.

**Post-Operative Care for DDH**

1. **Follow-Up Appointments**: Regular visits to the doctor to monitor your child's recovery.
2. **Physical Therapy**: Helps improve strength and mobility.
3. **Home Care Instructions**: Follow the doctor's advice on activities, exercises, and any necessary braces or supports.

**Support Groups and Community Resources**

1. **Online Support Groups**: Websites like Facebook and Reddit have communities where parents share experiences and advice.
2. **Local Hospitals**: Some hospitals have support groups for families dealing with DDH.
3. **Non-Profit Organizations**: Organizations like the International Hip Dysplasia Institute (IHDI) offer resources and support.

Would you like more details on any of these points?

Sure, I'd be happy to help explain this in simple terms!

**Developmental Dysplasia of the Hip (DDH) Treatment Costs**

**What is the usual cost of treatment for DDH?** The cost of treating DDH can vary depending on how early the condition is detected and the type of treatment needed. For example:

- **Early treatment**: If DDH is detected early (within the first few months), treatment with a Pavlik harness (a special brace) can cost around **$601**.
- **Late treatment**: If DDH is detected later, surgery might be needed, which can cost around **$4,352** per child. In more complex cases, the cost can go up to **$7,052**.

**Are there insurance or subsidy policies to reduce the financial burden?** Yes, many health insurance plans cover the cost of DDH treatment, including the use of a Pavlik harness and surgeries. It's important to check with your insurance provider to understand what is covered under your plan. Some countries or regions may also offer subsidies or financial assistance programs for families facing high medical costs due to DDH treatment.

**Social Assistance for Financial Hardship Due to DDH**

**What social assistance can I apply for if the disease or surgery causes financial hardship?** If the cost of DDH treatment causes financial hardship, you may be eligible for social assistance programs. These programs can help cover medical expenses, provide financial support, or offer other resources to ease the burden. Examples include:

- **Medicaid**: In the United States, Medicaid provides health coverage to low-income families, which can help cover the costs of DDH treatment.
- **Social Security Disability Insurance (SSDI)**: If the child's condition leads to long-term disability, SSDI may provide financial support.
- **State-specific programs**: Some states or regions have their own assistance programs for families dealing with high medical costs. It's a good idea to check with local social services agencies for available support.

I hope this helps! If you have any more questions or need further clarification, feel free to ask.

Sure, I'd be happy to help! Let's break it down into two parts: managing mental health, family responsibilities, and work in disease management, and understanding the impact of DDH on future quality of life and planning ahead.

**Balancing Mental Health, Family Responsibilities, and Work in Disease Management**

Managing a chronic condition like Developmental Dysplasia of the Hip (DDH) can be challenging, but it's important to balance your mental health, family responsibilities, and work. Here are some practical suggestions:

1. **Set Priorities**: Identify what's most important to you and focus on those areas. This might mean setting aside specific times for work, family, and self-care.
2. **Create a Routine**: Having a daily routine can help you manage your time better and reduce stress. Include time for medical appointments, family activities, and work tasks.
3. **Seek Support**: Don't hesitate to ask for help from family, friends, or support groups. Sharing your experiences can make you feel less isolated.
4. **Take Breaks**: Make sure to take regular breaks to relax and recharge. This can help prevent burnout and improve your overall well-being.
5. **Stay Organized**: Use tools like calendars, to-do lists, and reminders to keep track of your responsibilities and appointments.
6. **Practice Self-Care**: Take time for yourself to do things you enjoy, whether it's reading, exercising, or spending time with loved ones.
7. **Communicate**: Openly communicate with your employer about your condition and any accommodations you might need. This can help create a more supportive work environment.

**Impact of DDH on Future Quality of Life and Planning Ahead**

DDH can affect your future quality of life, but with proper management, you can improve your health and well-being. Here are some steps to plan ahead:

1. **Regular Check-Ups**: Schedule regular visits with your healthcare provider to monitor your condition and make any necessary adjustments to your treatment plan.
2. **Stay Active**: Engage in physical activities that are safe for your condition, such as swimming or gentle exercises, to maintain joint health and mobility.
3. **Healthy Diet**: Eat a balanced diet rich in nutrients to support overall health and joint function.
4. **Educate Yourself**: Learn about DDH and its management to make informed decisions about your care.
5. **Plan for the Future**: Consider long-term goals and how your condition might impact them. This might include planning for potential surgeries or lifestyle adjustments.
6. **Stay Positive**: Focus on what you can do rather than what you can't. A positive mindset can help you cope with challenges and improve your quality of life.

By balancing your mental health, family responsibilities, and work, and planning ahead for your health, you can manage DDH effectively and improve your overall well-being. Do you have any specific concerns or questions about managing DDH?

**Reply from Copilot (Third time)**

Sure, I'd be happy to help explain Developmental Dysplasia of the Hip (DDH) in simple terms!

**What is Developmental Dysplasia of the Hip (DDH)?**

DDH is a condition where a baby's hip joint hasn't formed properly. In a normal hip, the ball at the top of the thigh bone (femur) fits snugly into a socket in the pelvis1. In DDH, the ball is loose in the socket and can easily move out of place.

**Main Features and Potential Effects**

- **Main Features**: The hip joint is unstable, and the ball of the thigh bone doesn't fit well into the socket.
- **Potential Effects**: If not treated, it can lead to problems like limping, walking difficulties, and pain in the hip or leg.

**Who is Most Likely to Develop DDH?**

- **Most Likely to Develop**: Girls, firstborn children, babies born in the breech position (feet up by the shoulders), and those with a family history of DDH.
- **How Common is it?**: It's relatively rare, affecting about 1 to 2 babies per 1,000.

**Long-term Impact on Quality of Life**

- **Long-term Impact**: With early diagnosis and treatment, most children with DDH can lead normal, active lives.
- **Aspects to Pay Attention to**: Regular check-ups with a pediatrician, ensuring proper swaddling techniques, and monitoring for any signs of hip problems like uneven leg lengths or limited movement in one leg.

Does this help clarify things a bit?

Sure, I'd be happy to help explain Developmental Dysplasia of the Hip (DDH) in simple terms!

**What parts of the body does DDH mainly affect?**

DDH mainly affects the **hip joint**. In a healthy state, the hip joint is a ball-and-socket joint where the ball (the head of the thigh bone or femur) fits snugly into the socket (part of the pelvis called the acetabulum)1.

**What symptoms or consequences may arise if these parts are damaged?**

If the hip joint is damaged or doesn't develop properly (as in DDH), several symptoms and consequences can arise:

- **Pain and discomfort**: The child might experience pain in the hip, especially when moving.
- **Limping or walking difficulties**: The child might walk with a limp or have trouble walking normally.
- **Uneven leg lengths**: One leg might be shorter than the other.
- **Limited movement**: The child might have difficulty moving their hip joint fully.
- **Asymmetrical skin folds**: There might be uneven skin folds on the thighs.

**Real-life example**

Imagine a baby named Lily who has DDH. Lily's hip joint didn't form properly, so her thigh bone doesn't fit well into the hip socket1. Because of this, Lily might have trouble crawling or walking as she grows older. Her parents might notice that one of her legs seems shorter than the other, and she might walk with a limp1.

Does this help clarify things a bit?

Sure, I'd be happy to help explain Developmental Dysplasia of the Hip (DDH) in simple terms!

**What are the main causes of DDH?**

DDH happens when a baby's hip joint doesn't form properly. The exact cause isn't always clear, but it can be due to a mix of genetic and environmental factors2. For example, if a baby's family has a history of DDH, they might be more likely to have it too. Also, if a baby is in a breech position (feet-first) in the womb, it can increase the risk2.

**What risk factors may increase the chances of developing DDH?**

Some risk factors include:

- **Family history**: If parents or siblings had DDH, the baby is at higher risk.
- **First-born babies**: They have less room to move in the womb, which can affect hip development.
- **Female babies**: Girls are more likely to develop DDH than boys.
- **Breech position**: Babies in this position are at higher risk.
- **Tight uterus**: Limits the baby's movement, which can affect hip development.

**What lifestyle habits or environmental factors increase the risk of developing DDH?**

- **Swaddling**: Wrapping babies with their legs straight can increase the risk. It's better to swaddle them with their hips bent and apart.
- **Limited movement**: Keeping babies in one position for too long can also increase the risk.

**How can I prevent it?**

To help prevent DDH, you can:

- **Avoid tight swaddling**: Make sure to swaddle babies with their hips bent and apart.
- **Encourage movement**: Let babies move their legs freely.
- **Regular check-ups**: Have your baby checked by a doctor regularly to catch any issues early.

**If I have a family history, is my risk of developing DDH higher?**

Yes, having a family history of DDH does increase your risk.

**What preventive measures should I take?**

If you have a family history of DDH, it's important to:

- **Regular check-ups**: Make sure your baby gets regular check-ups with a doctor.
- **Be aware of symptoms**: Look out for signs like uneven skin folds or one leg appearing shorter.
- **Follow doctor's advice**: If your doctor recommends any special care or treatment, follow their advice closely.

I hope this helps! Do you have any more questions about DDH?

Sure! Let's break it down:

**Main Symptoms of Developmental Dysplasia of the Hip (DDH)**

- **Uneven leg lengths**: One leg might be shorter than the other.
- **Asymmetrical skin folds**: The skin folds on the thighs or buttocks might not line up evenly.
- **Limited movement**: One hip might not move as easily as the other.
- **Limping or waddling gait**: When walking, the child might limp or have a waddling walk.

**Signs the Disease is Worsening**

- **Increased limping**: The child's limp gets worse.
- **Pain**: The child starts to feel pain in the hip or leg.
- **Decreased mobility**: The child finds it harder to move the affected hip.
- **Visible deformity**: The hip joint might look out of place.

**When to Seek Immediate Medical Attention**

- **Sudden pain**: If the child suddenly feels pain in the hip or leg.
- **Severe limping**: If the child's limp becomes severe.
- **Swelling or bruising**: If there's swelling or bruising around the hip.
- **Inability to walk**: If the child can't walk or move the leg.

Does this help clarify things?

Sure, I'd be happy to help explain Developmental Dysplasia of the Hip (DDH) in simple terms!

**Diagnosis Methods**

Doctors use a few methods to confirm DDH:

1. **Physical Exam**: They check the baby's hips using two special tests called the **Barlow and Ortolani maneuvers**. These tests help see if the hip joint is loose or unstable1.
2. **Ultrasound**: This imaging test is used for babies younger than 6 months. It helps get a clear picture of the hip joint2.
3. **X-ray**: For older babies, an X-ray is used to see the bones and check for any abnormalities.

**Important Indicators in Test Results**

- **Barlow and Ortolani Tests**: If the hip "clunks" during these tests, it might indicate DDH.
- **Ultrasound**: Shows if the hip joint is properly formed.
- **X-ray**: Shows the position and shape of the hip bones.

**Severity Reflection**

- **Mild DDH**: The hip joint is slightly loose but might get better on its own.
- **Severe DDH**: The hip joint is very loose or dislocated, which might need treatment like a brace or surgery.

**Follow-ups After Diagnosis**

Yes, regular follow-ups are important! Here's what to expect:

- **Frequency**: Follow-ups might be every few weeks or months, depending on the severity.
- **Checks**: Doctors will check the hip's position and movement, and might use ultrasounds or X-rays to monitor progress.

Does this help clarify things a bit?

Sure, I'd be happy to help explain this in simple terms!

**Symptoms Indicating Immediate Medical Attention for DDH**

If your child has **Developmental Dysplasia of the Hip (DDH)**, you should seek immediate medical attention if you notice any of these symptoms:

1. **Uneven leg lengths**: One leg appears shorter than the other.
2. **Uneven skin folds**: The skin folds on the thighs or buttocks are not symmetrical.
3. **Limited movement**: One leg doesn't move as freely or widely as the other.
4. **Limping or waddling gait**: Your child walks with a limp or a waddling motion.
5. **Pain or discomfort**: Your child seems to be in pain or is uncomfortable when moving.

**Handling and Self-Monitoring During an Emergency Situation**

If you suspect your child has DDH and it's an emergency, here's what you can do:

1. **Keep calm**: Stay calm to help your child stay calm too.
2. **Avoid moving the hip**: Do not try to move or adjust the hip yourself.
3. **Support the leg**: Gently support the affected leg in a comfortable position.
4. **Seek medical help immediately**: Call emergency services or take your child to the nearest hospital.

Remember, it's always best to consult with a pediatrician if you have any concerns about your child's hip development. They can provide guidance and ensure your child gets the appropriate care.

Does this help clarify things?

Sure, I'd be happy to help explain things in simple terms! Let's use Developmental Dysplasia of the Hip (DDH) in children as an example.

**Treatments During Hospitalization**

1. **Pavlik Harness**: This is a special brace that keeps the baby's hips in the right position. It helps the hip joint develop properly.
2. **Surgery**: In more severe cases, doctors might need to perform surgery to put the hip back in place and secure it.

**Preventing Infections or Complications**

- **Keep Everything Clean**: Make sure the baby's surroundings are clean to avoid infections.
- **Follow Doctor's Instructions**: Follow all the doctor's advice on how to care for the baby, including how to use the brace or take care of the surgical area.
- **Family Support**: Family members can help by keeping the baby's environment clean and making sure the baby is comfortable and safe.

**Reinforcing Treatment After Discharge**

- **Physical Therapy**: After the baby goes home, physical therapy can help strengthen the hip muscles and improve movement.
- **Regular Check-ups**: Keep going to the doctor for regular check-ups to make sure the hip is developing well.
- **Encouragement**: Encourage the baby to move and play as much as possible to help with recovery.

Does this help clarify things?

Sure! Let's break it down:

**Common Medications for Developmental Dysplasia of the Hip (DDH)**

For children with DDH, the most common treatment is **bracing** rather than medication. The **Pavlik Harness** is often used for newborns and young infants to keep the hip joint in place while it develops properly.

**How to Take the Medication Correctly**

Since DDH treatment mainly involves using a brace, it's important to:

1. **Wear the brace as directed**: Usually, full-time wear for 6-12 weeks.
2. **Follow the doctor's instructions**: Some doctors allow removal for bathing and diaper changes.
3. **Keep the legs apart**: This helps the hips stay in the correct position.

**What to Do If You Forget to Take the Medication or Take It at the Wrong Time**

If you forget to wear the brace or take it off too early:

1. **Put it back on as soon as you remember**: Don't leave it off for too long.
2. **Contact your doctor**: If you're unsure or if it happens frequently, call your healthcare provider for advice.

**Foods to Avoid While Taking Medication for DDH**

There aren't specific foods to avoid for DDH treatment since it's mainly about wearing a brace. However, maintaining a healthy diet is always important for overall health.

**Common Side Effects**

Since DDH treatment is non-medication based, there aren't common side effects from medications. However, wearing a brace might cause some skin irritation or discomfort. If this happens, contact your doctor for advice on how to manage it.

Does this help clarify things?

Sure, I'd be happy to help explain this in simple terms!

**What types of pain may be caused by DDH or its treatment? Where is the pain usually felt?**

**DDH** can cause pain in the hip or groin area. If untreated, it can lead to arthritis and more severe pain later in life2. Treatment, like surgery, can also cause pain around the hip.

**What methods can help relieve pain? What are the recommended treatments?**

**Non-pharmacological treatments** include:

- **Physical therapy**: Exercises to strengthen muscles around the hip.
- **Bracing**: Using a Pavlik harness for babies to keep the hip in place.
- **Safe swaddling**: Keeping the baby's hips apart when swaddling.

**Pharmacological treatments** include:

- **Pain relievers**: Over-the-counter medications like acetaminophen or ibuprofen.

**What steps should I take if the pain is more than expected or worsens?**

If the pain is worse than expected or gets worse, **contact your doctor**. They might need to adjust your treatment plan or check for complications.

Does this help clarify things?

Sure, I'd be happy to help explain everything in simple terms! Let's use Developmental Dysplasia of the Hip (DDH) in children as an example.

**Post-Surgery Care Precautions**

After surgery, it's important to follow these precautions:

- **Keep the hip area clean and dry** to prevent infection.
- **Avoid putting pressure on the hip** by not sitting on hard surfaces or crossing legs.
- **Use crutches or a walker** as advised by the doctor to avoid putting weight on the hip.
- **Follow the doctor's instructions** for any medications to manage pain and prevent infection.

**Returning to Normal Daily Activities**

Most children can start doing light activities within **2-3 weeks** after surgery. Full recovery and return to normal activities might take **3-6 months**. Always follow the doctor's advice on when it's safe to resume activities.

**Resuming Activities**

Children can usually start gentle activities like walking and light play **2-3 weeks** after surgery. More strenuous activities like running and jumping should be avoided until the doctor gives the go-ahead, usually around **3-6 months**.

**Recommended Activities During Recovery**

During recovery, it's good to do:

- **Gentle walking** to keep the joint moving.
- **Physical therapy exercises** to strengthen muscles around the hip.
- **Swimming** (if allowed by the doctor) as it's gentle on the joints.

**Scheduling Exercise**

Start with **short sessions** of gentle exercises and gradually increase the duration as you feel stronger. Aim for **2-3 times a day** but listen to your body and rest if you feel pain.

**Stages During Postoperative Recovery**

1. **Immediate Post-Surgery (0-2 weeks)**: Focus on healing and gentle movements.
2. **Early Recovery (2-6 weeks)**: Gradually increase activity levels with physical therapy.
3. **Intermediate Recovery (6-12 weeks)**: Resume more normal activities but avoid heavy lifting.
4. **Long-Term Recovery (3-6 months)**: Continue strengthening exercises and slowly return to full activities.

**Specialized Rehabilitation Plan**

Yes, a specialized rehabilitation plan is important. It helps tailor exercises to your child's specific needs, ensuring proper healing and preventing complications.

**Preventing Recurrence**

To prevent recurrence, follow the doctor's advice on activity restrictions, continue with prescribed exercises, and attend regular follow-up appointments.

**Possible Complications**

Complications can include infection, blood clots, or issues with the hip joint. If you notice increased pain, swelling, or redness, contact the doctor immediately.

I hope this helps! Do you have any more questions or need further clarification?

Sure! Let's break it down step by step:

**Foods Helpful in Recovery from DDH or During Post-Surgery Recovery**

**Helpful Foods:**

- **Fruits and Vegetables:** Packed with vitamins and minerals, they help your body heal.
- **Lean Proteins:** Chicken, fish, beans, and tofu help repair tissues.
- **Whole Grains:** Foods like brown rice and oatmeal provide energy and fiber.
- **Dairy:** Milk, yogurt, and cheese provide calcium for strong bones.

**Foods to Avoid:**

- **Sugary Foods:** Can lead to inflammation and slow healing.
- **Processed Foods:** Often high in unhealthy fats and additives.
- **Excessive Salt:** Can cause water retention and swelling.

**Adjusting Your Diet to Prevent Constipation**

- **Increase Fiber:** Eat more fruits, vegetables, and whole grains.
- **Stay Hydrated:** Drink plenty of water throughout the day.
- **Limit Processed Foods:** They often lack fiber and can cause constipation.

**Exercises During Recovery**

**Recommended Exercises:**

- **Gentle Stretching:** Helps maintain flexibility.
- **Walking:** Low-impact exercise to improve circulation.
- **Physical Therapy Exercises:** Specific exercises prescribed by your therapist.

**Frequency:**

- Aim for at least 30 minutes of gentle exercise daily, but follow your doctor's advice.

**Precautions When Exercising**

- **Start Slow:** Gradually increase the intensity of your exercises.
- **Listen to Your Body:** Stop if you feel pain or discomfort.
- **Follow Instructions:** Stick to the exercise plan given by your healthcare provider.

**DDH and Sleep Quality**

**Does DDH Affect Sleep?**

- Yes, pain and discomfort from DDH can affect sleep quality.

**Improving Sleep:**

- **Comfortable Bedding:** Use supportive pillows and mattresses.
- **Relaxation Techniques:** Try deep breathing or meditation before bed.
- **Consistent Schedule:** Go to bed and wake up at the same time every day.

I hope this helps! Do you have any specific questions or need more details on any of these points?

Sure, I'd be happy to help explain this in simple terms!

**Psychological Problems Associated with DDH or Postoperative Recovery**

Children with Developmental Dysplasia of the Hip (DDH) and their families might face **anxiety** and **depression**. This can happen because:

- **Anxiety**: Parents might worry about their child's health, treatment, and future.
- **Depression**: Both children and parents might feel sad or hopeless due to the challenges of managing DDH and its treatments.

**How to Deal with Them Positively**

1. **Stay Informed**: Learn about DDH and its treatments. Knowledge can reduce fear of the unknown.
2. **Seek Support**: Talk to friends, family, or join support groups for parents of children with DDH.
3. **Professional Help**: Consider seeing a therapist or counselor who can provide strategies to manage anxiety and depression.
4. **Self-Care**: Take time for yourself to relax and recharge. This can help you stay strong for your child.

**Signs You Need Psychological Support**

You might need support if you or your child:

- Feel overwhelmed or constantly worried.
- Have trouble sleeping or experience changes in appetite.
- Feel sad or hopeless for a long time.
- Have trouble concentrating or enjoying activities you used to like.

**Finding Professional Help or Support Resources**

1. **Talk to Your Doctor**: They can refer you to a mental health professional.
2. **Online Resources**: Websites like the American Psychological Association (APA) can help you find therapists.
3. **Local Support Groups**: Look for local groups for parents of children with DDH.
4. **School Counselors**: If your child is school-aged, school counselors can provide support and resources.

I hope this helps! Do you have any other questions or need more information?

Sure! Let's talk about Developmental Dysplasia of the Hip (DDH) in children and how health apps and devices can help manage it and monitor recovery.

**Recommended Health Apps for DDH Management and Postoperative Recovery**

1. **MyFitnessPal**: This app helps track nutrition and exercise, which is important for overall health and recovery.
2. **Medisafe**: This app reminds you to take medications on time, which is crucial after surgery.
3. **Headspace**: This app offers guided meditation and mindfulness exercises to help manage stress and improve mental well-being during recovery.
4. **Recovery Record**: This app helps track progress, set goals, and receive motivational messages to support recovery.
5. **PT Pal**: This app provides physical therapy exercises and routines to promote mobility and strength.

**How to Use Health Tracking Tools for DDH Management and Recovery**

1. **Track Nutrition**: Use apps like MyFitnessPal to log what you eat and ensure you're getting the right nutrients for healing.
2. **Medication Reminders**: Apps like Medisafe can remind you when to take your medications, ensuring you don't miss any doses.
3. **Monitor Pain Levels**: Use apps like PainTracker to record your pain levels, helping your doctor adjust your treatment plan if needed.
4. **Physical Therapy**: Apps like PT Pal provide exercises and routines to follow, helping you regain strength and mobility.
5. **Mindfulness and Stress Management**: Use Headspace to practice meditation and mindfulness, which can help reduce stress and improve your overall well-being.

By using these apps and tools, you can better manage DDH and monitor your recovery progress. They provide support, reminders, and motivation to help you stay on track with your treatment plan.

Does this help clarify things?

Sure! Let's talk about Developmental Dysplasia of the Hip (DDH) in children and how to find reliable information, support groups, and community resources.

**Reliable Information about DDH**

1. **Pediatrician**: Your child's doctor is a great first source of information. They can explain DDH and answer your questions.
2. **Children's Hospitals**: Websites like the Children's Hospital of Philadelphia (CHOP) have detailed information about DDH.
3. **American Academy of Pediatrics (AAP)**: They provide guidelines and information on DDH.
4. **Medical Websites**: Trusted sites like Mayo Clinic, WebMD, and Orthobullets offer reliable information4.

**Post-Operative Care for DDH**

1. **Follow-Up Appointments**: Regular visits to your child's doctor to monitor progress.
2. **Physical Therapy**: Helps in recovery and improving mobility.
3. **Home Care Instructions**: Follow the doctor's advice on activities, exercises, and any braces or supports needed.

**Support Groups and Community Resources**

1. **Online Support Groups**: Websites like Facebook and Reddit have groups where parents share experiences and advice.
2. **Local Hospitals**: Some hospitals have support groups for families dealing with DDH.
3. **Non-Profit Organizations**: Organizations like the International Hip Dysplasia Institute (IHDI) offer resources and support.

Would you like more details on any of these points?

Sure, I'd be happy to help explain!

**Developmental Dysplasia of the Hip (DDH) in Children**

**What is the usual cost of treatment for DDH?** The cost of treating DDH can vary depending on the severity and the age at which it's diagnosed. For early detection and treatment, using a Pavlik harness (a special brace), the cost is around **$601 per child**. However, if DDH is detected late and requires surgery, the cost can go up to **$7,052 per child**. This includes different types of surgeries like closed or open reduction1.

**Are there insurance or subsidy policies to reduce the financial burden?** Yes, many health insurance plans cover the treatment of DDH, especially if it's considered a medical necessity. It's important to check with your insurance provider to understand what's covered. Some countries or regions may also offer subsidies or financial assistance programs for families facing high medical costs due to DDH treatment.

**What social assistance can I apply for if the disease or surgery causes financial hardship?** If the cost of DDH treatment causes financial hardship, you may be eligible for social assistance programs. These can include:

- **Medicaid**: Provides health coverage to low-income families.
- **Children's Health Insurance Program (CHIP)**: Offers low-cost health coverage to children in families that earn too much for Medicaid but not enough to afford private coverage.
- **State-specific programs**: Some states have additional programs to help families with medical expenses.

Would you like more information on any of these points?

Sure, I'd be happy to help! Let's break it down into two parts: managing mental health, family responsibilities, and work while dealing with DDH, and planning ahead for future health improvement.

**Balancing Mental Health, Family Responsibilities, and Work in Disease Management**

Managing a child's DDH can be overwhelming, but here are some practical suggestions:

1. **Create a Routine**: Establish a daily routine that includes time for medical appointments, family activities, and work. This helps in managing time effectively and reduces stress.
2. **Seek Support**: Don't hesitate to ask for help from family, friends, or support groups. Sharing responsibilities can lighten the load.
3. **Set Boundaries**: Clearly define work hours and personal time. This ensures you have time to relax and recharge.
4. **Stay Organized**: Keep track of medical appointments, medications, and therapies using a planner or digital calendar.
5. **Practice Self-Care**: Take time for yourself to relax and unwind. Activities like reading, walking, or meditation can help reduce stress.
6. **Communicate**: Talk openly with your employer about your situation. They may offer flexible working hours or other accommodations.

**Impact of DDH on Future Quality of Life and Planning Ahead**

DDH can affect a child's future quality of life, but planning ahead can help improve health outcomes:

1. **Early Detection and Treatment**: Regular check-ups and early treatment can prevent complications and improve long-term outcomes.
2. **Physical Therapy**: Engage in physical therapy to strengthen muscles and improve mobility.
3. **Healthy Lifestyle**: Encourage a healthy diet and regular exercise to maintain overall health.
4. **Monitor Progress**: Keep track of the child's development and seek medical advice if there are any concerns.
5. **Stay Informed**: Educate yourself about DDH and stay updated on the latest treatments and therapies.
6. **Plan for the Future**: Work with healthcare providers to create a long-term care plan that addresses potential challenges and ensures the child receives the necessary support.

By balancing responsibilities and planning ahead, you can effectively manage DDH and improve your child's quality of life. Do you have any specific concerns or questions about managing DDH?

**Reply from DeepSeek-V3 (First time)**

**What is Developmental Dysplasia of the Hip (DDH)?**

DDH is a condition where a baby’s hip joint doesn’t form properly. The hip joint is like a ball (the top of the thigh bone) sitting in a socket (part of the pelvis). In DDH, the ball may be loose in the socket, partially out, or completely out of place. If not treated, it can lead to pain, walking problems, or arthritis later in life.

Think of it like a puzzle piece that doesn’t fit right. If you don’t fix it early, it can cause bigger problems as the child grows.

**Who is most likely to develop DDH? How common is it?**

DDH is more common in:

- **Firstborn babies**: The womb is tighter, which can affect hip development.
- **Girls**: Their ligaments are looser, making the hip joint less stable.
- **Babies born breech (feet first)**: This position puts extra pressure on the hips.
- **Family history**: If a parent or sibling had DDH, the risk is higher.

About 1 in 1,000 babies has DDH severe enough to need treatment. Mild cases are more common but often fix themselves.

**Will DDH have a long-term impact on my quality of life? What should I pay attention to?**

If treated early, most children with DDH grow up without problems. Treatment might include a special harness, brace, or, in rare cases, surgery.

If not treated, DDH can cause:

- **Walking difficulties**: The child might limp or walk unevenly.
- **Pain**: The hip joint can wear out faster, leading to arthritis.
- **Uneven leg lengths**: One leg might grow shorter than the other.

**What to pay attention to:**

- **Early signs**: Look for uneven skin folds on the thighs, one leg that seems shorter, or difficulty moving one leg.
- **Regular check-ups**: Doctors check babies’ hips during routine visits. Don’t skip these!
- **Follow treatment plans**: If your child needs a harness or brace, use it as directed.

**Real-life example:**
Imagine a baby named Mia. Her parents noticed her left leg didn’t move as much as her right. The doctor diagnosed DDH and gave her a soft harness to wear. After a few months, her hip developed normally, and she grew up to run and play like any other kid. Early action made all the difference!

In short, DDH is treatable, especially when caught early. Pay attention to signs, follow the doctor’s advice, and your child can live a healthy, active life.

**What is Developmental Dysplasia of the Hip (DDH)?**

Developmental Dysplasia of the Hip (DDH) is a condition where the hip joint doesn’t form properly in babies and young children. The hip joint is where the thigh bone (femur) connects to the pelvis. In a healthy hip, the top of the thigh bone (the "ball") fits snugly into a socket in the pelvis. This allows the hip to move smoothly and support the body’s weight.

**What parts of the body does DDH mainly affect?**

DDH mainly affects the **hip joint**. The hip joint is made up of two main parts:

1. **The ball**: This is the rounded top of the thigh bone (femur).
2. **The socket**: This is the cup-shaped part of the pelvis where the ball sits.

In a healthy hip, the ball and socket fit together perfectly. This allows the hip to move freely and carry the body’s weight when standing, walking, or running.

**What symptoms or consequences may arise if these parts are damaged?**

If the hip joint doesn’t form properly (like in DDH), it can cause problems. Here are some examples:

1. **Limping or uneven walking**: If the hip is unstable, a child might walk with a limp. For example, one leg might look shorter, or they might waddle like a duck.
2. **Pain or stiffness**: As the child grows, the poorly formed hip can cause pain, especially during activities like running or climbing stairs.
3. **Limited movement**: The hip might not move as freely as it should. For example, a child might have trouble spreading their legs apart, like when sitting on a tricycle.
4. **Arthritis later in life**: If DDH isn’t treated, it can lead to early arthritis in the hip. This means the joint becomes worn out and painful, even in adulthood.

**Real-life example:**

Imagine a baby named Mia. When Mia was born, her hip socket was too shallow, so the ball of her thigh bone didn’t fit properly. At first, her parents didn’t notice anything wrong. But as Mia started walking, they saw she was limping and one leg seemed shorter. The doctor diagnosed her with DDH. Mia wore a special brace for a few months to help her hip develop correctly. Because her parents caught it early, Mia’s hip healed, and she can now run and play like other kids.

**Key takeaway:**

DDH affects the hip joint, which is important for movement and support. If not treated, it can cause pain, limping, and long-term problems like arthritis. But with early detection and treatment, most children with DDH can grow up to have healthy, strong hips.

3 / 3

**What are the main causes of DDH? What risk factors may increase the chances of developing it?**

**Developmental Dysplasia of the Hip (DDH)** is a condition where the hip joint doesn’t form properly in babies and young children. The hip joint is like a ball (the top of the thigh bone) that fits into a socket (part of the pelvis). In DDH, the ball may be loose in the socket or even slip out completely.

**Main Causes:**

- The exact cause isn’t always clear, but it’s often related to how the hip develops before birth or in early infancy.
- Sometimes, the baby’s position in the womb (like being breech, where the baby’s bottom is down instead of the head) can put pressure on the hips, leading to DDH.

**Risk Factors:**

1. **Family History:** If someone in the family had DDH, the baby is more likely to have it too.
2. **Breech Position:** Babies born bottom-first are at higher risk.
3. **Firstborn Children:** The womb is tighter with the first baby, which can increase pressure on the hips.
4. **Gender:** Girls are more likely to have DDH than boys.
5. **Swaddling:** If a baby’s legs are wrapped tightly straight down (like in some swaddling practices), it can increase the risk.

**What lifestyle habits or environmental factors increase the risk of developing DDH? How can I prevent it?**

**Lifestyle/Environmental Factors:**

- **Swaddling Incorrectly:** Wrapping a baby’s legs too tightly in a straight position can put stress on the hips. Always swaddle with the legs loose and able to bend.
- **Carrying the Baby Incorrectly:** Using baby carriers or slings that force the legs straight can also increase the risk. Make sure the baby’s legs are spread apart and supported.

**Prevention Tips:**

1. **Proper Swaddling:** When swaddling, leave room for the baby’s legs to move and bend. The hips should be able to spread naturally.
2. **Use Hip-Friendly Carriers:** Choose baby carriers that support the baby’s thighs and keep the hips in a healthy position.
3. **Regular Checkups:** Have your baby’s hips checked by a doctor during routine visits. Early detection can prevent serious problems.

**If I have a family history, is my risk of developing DDH higher? What preventive measures should I take?**

**Family History and Risk:**

- Yes, if someone in your family had DDH, your baby is more likely to have it too. This is because genes can play a role in how the hip joint forms.

**Preventive Measures:**

1. **Tell Your Doctor:** If DDH runs in your family, let your pediatrician know. They may check your baby’s hips more closely.
2. **Hip Ultrasounds:** In some cases, the doctor might recommend an ultrasound to check the hip joint, especially if there are other risk factors like breech position.
3. **Monitor Hip Movement:** Watch for signs like uneven leg folds, difficulty moving one leg, or a clicking sound in the hip. If you notice anything unusual, tell your doctor.
4. **Avoid Tight Swaddling:** As mentioned earlier, always swaddle with the hips in a natural position.

**Real-Life Example:**

Imagine a baby named Emma. Her mom had DDH as a baby, so Emma’s doctor checks her hips carefully at every visit. Emma’s mom also makes sure to swaddle her loosely, with her legs able to bend and spread naturally. When carrying Emma in a baby carrier, her mom chooses one that supports her thighs and keeps her hips in a healthy position. These small steps help reduce Emma’s risk of developing DDH.

**What are the main symptoms of Developmental Dysplasia of the Hip (DDH) in children?**

Developmental Dysplasia of the Hip (DDH) is a condition where a baby’s hip joint doesn’t form properly. This can make the hip loose or even dislocated. Here are some signs to look for:

1. **Uneven Legs or Buttocks**: One leg might look shorter than the other. The folds on the thighs or buttocks might not match.
2. **Limited Movement**: The baby might not move one leg as much as the other. For example, one leg might not spread out as far during diaper changes.
3. **Clicking or Popping Sounds**: You might hear a clicking or popping sound when moving the baby’s hip.
4. **Walking Problems**: When the child starts walking, they might limp or walk on their toes.

**What signs indicate the disease is worsening?**

If DDH isn’t treated, it can get worse. Here are some signs that the condition might be worsening:

1. **Increased Pain**: The child might start to feel pain in the hip, especially when moving it.
2. **Difficulty Walking**: The child might limp more or have trouble walking. They might avoid putting weight on the affected leg.
3. **Uneven Gait**: The child’s walk might look uneven or wobbly.
4. **Reduced Mobility**: The child might not be able to move the hip as much as before. For example, they might not be able to spread their legs wide.

**Should I seek immediate medical attention?**

Yes, if you notice any of these signs, you should see a doctor as soon as possible. Early treatment is very important for DDH. The sooner it’s treated, the better the chances of fixing the problem without surgery.

Here’s a real-life example: Imagine you’re changing your baby’s diaper and notice that one leg doesn’t move as freely as the other. Or maybe you hear a clicking sound when you move their hip. These could be signs of DDH. Don’t wait—make an appointment with your pediatrician. They can check the hip and might send you to a specialist if needed.

In summary, if you see any signs of DDH, like uneven legs, limited movement, or clicking sounds, don’t wait. Early treatment can make a big difference.

**What methods do doctors usually use to confirm the diagnosis of Developmental Dysplasia of the Hip (DDH)? What is the purpose of each test?**

1. **Physical Examination**:
   - Doctors check the baby’s hips during routine check-ups. They move the legs to see if the hip joint is loose or dislocated.
   - **Purpose**: To find signs of DDH early, like a clicking sound or limited movement in the hip.
2. **Ultrasound**:
   - This is a safe and painless test that uses sound waves to create pictures of the baby’s hip joint.
   - **Purpose**: To see if the hip socket is shallow or if the ball of the hip is not in the right place. It’s often used for babies under 6 months.
3. **X-ray**:
   - For older babies (over 6 months), doctors may use an X-ray to look at the bones of the hip.
   - **Purpose**: To check how the hip bones are growing and if they are in the correct position.

**What indicators are most important in the test results? How do these results reflect the severity of the disease?**

1. **Hip Socket Shape (on Ultrasound or X-ray)**:
   - A shallow hip socket means the hip joint is not forming properly. This is a key sign of DDH.
   - **Severity**: The shallower the socket, the more severe the DDH.
2. **Position of the Hip Ball (Femoral Head)**:
   - If the ball of the hip is not sitting in the socket, it could be partially or fully dislocated.
   - **Severity**: A fully dislocated hip is more serious than a slightly loose one.
3. **Range of Motion (Physical Exam)**:
   - If the baby’s legs don’t move as freely as they should, it could mean the hip is not stable.
   - **Severity**: Less movement often means a more severe problem.

**After confirming the diagnosis of DDH, do I need to schedule regular follow-ups? How often should I have them and what should be checked?**

Yes, regular follow-ups are very important to make sure the treatment is working and the hip is developing properly.

1. **Frequency**:
   - For mild cases: Every few months.
   - For severe cases: More often, like every 4-6 weeks, especially if the baby is wearing a brace or cast.
2. **What’s Checked**:
   - **Hip Movement**: The doctor will check if the hip is becoming more stable.
   - **Imaging Tests**: Ultrasounds or X-rays may be repeated to see if the hip socket is improving.
   - **Growth and Development**: The doctor will make sure the baby’s legs are growing evenly and the child is meeting milestones like crawling or walking.

**Real-Life Example:**

Imagine a baby named Mia. At her 2-month check-up, the doctor notices her left hip feels loose. An ultrasound shows her hip socket is shallow. Mia gets a special brace to wear. Her parents bring her back every 6 weeks for check-ups. Over time, the ultrasound shows her hip socket is getting deeper, and her hip becomes more stable. By the time Mia starts walking, her hip is normal, thanks to early treatment and regular follow-ups.

This shows how important tests, treatment, and follow-ups are for DDH!

**What symptoms indicate that I need to seek immediate medical attention?**

If you notice any of the following signs in your child, it’s important to see a doctor right away:

1. **Uneven leg lengths**: One leg looks shorter than the other when your child is lying down.
2. **Uneven skin folds**: The folds on the thighs or buttocks look different on one side compared to the other.
3. **Limited movement**: Your child has trouble moving one leg or hip as much as the other.
4. **Clicking or popping sounds**: You hear a clicking or popping sound when moving your child’s hip.
5. **Limping or walking oddly**: If your child is old enough to walk, they might limp or walk on their toes.

For example, if you’re changing your baby’s diaper and notice one leg doesn’t move as easily as the other, or if your toddler starts limping suddenly, these could be signs of DDH.

**How should I handle and self-monitor during an emergency situation?**

If you suspect your child has DDH, here’s what you can do:

1. **Stay calm**: It’s important to stay calm so you can think clearly and help your child.
2. **Avoid moving the hip too much**: Try not to move the affected hip too much, as this could cause more discomfort.
3. **Comfort your child**: Keep your child comfortable and try to distract them with toys or songs.
4. **Seek medical help**: Call your doctor or go to the emergency room if the symptoms seem severe.

For example, if your baby seems to be in pain when you move their legs during a diaper change, stop moving the legs and call your doctor. If your toddler is limping and complaining of pain, keep them off their feet and take them to the doctor as soon as possible.

Remember, early treatment is key to helping your child recover fully, so don’t wait to seek medical advice if you notice any of these symptoms.

2 / 2

1. **Pavlik Harness**:
   - **What it is**: A soft brace that keeps your baby’s hips in the right position.
   - **Purpose**: It helps the hip joint grow correctly. Think of it like a gentle guide for the hip to stay in place.
   - **Example**: Like how a cast helps a broken bone heal straight, the harness helps the hip grow properly.
2. **Casting (Spica Cast)**:
   - **What it is**: A hard cast that covers the baby’s hips and legs.
   - **Purpose**: It holds the hip in place if the harness doesn’t work.
   - **Example**: Imagine a stiff, protective shell that keeps everything stable so the hip can heal.
3. **Surgery**:
   - **What it is**: A doctor fixes the hip joint in an operation.
   - **Purpose**: For severe cases, surgery puts the hip back in the right position.
   - **Example**: Like fixing a puzzle piece that’s in the wrong spot, the surgeon moves the hip to where it should be.
4. **Physical Therapy**:
   - **What it is**: Gentle exercises to help the hip move and strengthen.
   - **Purpose**: It keeps the hip flexible and strong after treatment.
   - **Example**: Like stretching and moving a stiff door hinge to make it work smoothly again.

**How can I prevent infections or other complications during hospitalization? What can family members do to assist with recovery?**

1. **Prevent Infections**:
   - **Wash Hands**: Doctors, nurses, and family should wash hands often.
   - **Keep Wounds Clean**: If there’s surgery, keep the area clean and dry.
   - **Example**: Like cleaning a scraped knee to avoid germs.
2. **Family Help**:
   - **Follow Instructions**: Learn how to care for the harness, cast, or surgery site.
   - **Comfort**: Keep the baby calm and happy. Play with them and distract them if they’re uncomfortable.
   - **Example**: Like helping a child with a broken arm feel better by reading them a story.
3. **Watch for Problems**:
   - **Check Skin**: Look for redness or sores under the harness or cast.
   - **Monitor Fever**: Tell the doctor if the baby has a fever or seems sick.
   - **Example**: Like checking a bandage to make sure it’s not causing problems.

**How can I reinforce the effects of treatment after discharge? What rehabilitation programs are recommended?**

1. **Follow-Up Appointments**:
   - **What to do**: Go to all doctor visits to check the hip’s progress.
   - **Purpose**: The doctor makes sure the hip is healing well.
   - **Example**: Like taking your car for regular check-ups to keep it running smoothly.
2. **Physical Therapy**:
   - **What to do**: Do exercises at home or with a therapist.
   - **Purpose**: Keeps the hip strong and flexible.
   - **Example**: Like doing stretches to keep your muscles from getting stiff.
3. **Use Braces or Casts as Directed**:
   - **What to do**: Wear the harness or cast exactly as the doctor says.
   - **Purpose**: Gives the hip time to heal in the right position.
   - **Example**: Like wearing a retainer after braces to keep teeth straight.
4. **Encourage Movement**:
   - **What to do**: Let the baby move and play safely.
   - **Purpose**: Helps the hip develop naturally.
   - **Example**: Like letting a toddler walk and explore to build strong legs.
5. **Healthy Habits**:
   - **What to do**: Feed the baby a healthy diet and make sure they get enough sleep.
   - **Purpose**: Helps the body heal and grow.
   - **Example**: Like eating well and resting to recover from a cold.

By following these steps, you can help your child’s hip heal and grow strong!

**What are the common medications used to treat developmental dysplasia of the hip (DDH) in children? How should I take them correctly?**

Developmental dysplasia of the hip (DDH) is a condition where a baby’s hip joint doesn’t form properly. It’s not usually treated with medications. Instead, doctors focus on fixing the hip joint using special braces, harnesses, or sometimes surgery. For example, a Pavlik harness is a soft brace that helps hold the baby’s hips in the right position so they can develop normally.

If your child has pain after treatment, the doctor might recommend pain relievers like **acetaminophen** (Tylenol) or **ibuprofen** (Advil). Always follow the doctor’s instructions on how much to give and how often. Use the measuring tool that comes with the medicine to avoid giving too much.

**What should I do if I forget to give my child their pain medication or give it at the wrong time? Will it affect the treatment?**

If you forget to give your child their pain medication, don’t worry too much. Pain relievers like acetaminophen or ibuprofen are not the main treatment for DDH. They are just used to manage discomfort. If you miss a dose, give it as soon as you remember, but don’t double the dose to make up for it. For example, if you were supposed to give it at 10 AM and remember at 1 PM, give it then. If it’s almost time for the next dose, skip the missed one.

Missing a dose of pain medication won’t affect the hip treatment itself. The most important part of DDH treatment is using the brace or harness correctly and following up with the doctor.

**What foods should I avoid while giving my child medication for DDH? Are there common side effects?**

Since DDH is not treated with long-term medications, there aren’t specific foods to avoid. However, if your child is taking pain relievers like acetaminophen or ibuprofen, here are a few tips:

1. **Acetaminophen (Tylenol):** Avoid giving your child sugary drinks or snacks right after the medicine. Too much sugar can sometimes upset their stomach. Also, make sure they don’t take more than the recommended dose, as too much acetaminophen can harm the liver.
2. **Ibuprofen (Advil):** Give this medicine with food or milk to prevent stomach upset. Avoid giving it on an empty stomach.

**Common side effects** of these medications are usually mild and include:

- Upset stomach
- Drowsiness
- Allergic reactions (rare, but watch for rashes or swelling)

If your child shows signs of an allergic reaction or severe side effects, stop the medication and call the doctor right away.

**Real-life example:**

Imagine your baby is wearing a Pavlik harness for DDH. They might feel a little uncomfortable at first, so you give them a dose of acetaminophen as the doctor suggested. You accidentally forget the next dose, but it’s okay because the harness is doing the real work of fixing the hip. Just give the next dose on time and keep using the harness as directed. If your child seems extra fussy or has a rash, call the doctor to make sure everything is okay.

**1. What types of pain may be caused by DDH or its treatment? Where is the pain usually felt?**

Developmental Dysplasia of the Hip (DDH) is a condition where a baby’s hip joint doesn’t form properly. The hip joint is where the thigh bone (femur) connects to the pelvis. If DDH isn’t treated, it can cause pain later in life, usually in the hip or groin area. Sometimes, the pain can spread to the thigh or knee.

If a child needs treatment for DDH, like wearing a brace or having surgery, they might feel some discomfort or pain. For example:

- **Brace treatment**: A special device (like a harness) is used to hold the hip in place. This might feel tight or uncomfortable at first.
- **Surgery**: After surgery, the child may feel pain around the hip or where the incision was made.

**2. What methods can help relieve pain? What are the recommended pharmacological and non-pharmacological treatments?**

There are ways to help ease the pain caused by DDH or its treatment:

**Non-pharmacological (without medicine):**

- **Comfortable positioning**: For babies in a brace, make sure they’re positioned comfortably. Use soft padding if needed.
- **Gentle massage**: For older kids, gently massaging the area around the hip can help ease muscle tension.
- **Heat or cold packs**: A warm towel or cold pack (wrapped in a cloth) can help reduce soreness.
- **Physical therapy**: Exercises can strengthen the muscles around the hip and improve movement.

**Pharmacological (with medicine):**

- **Pain relievers**: Doctors may recommend medicines like acetaminophen (Tylenol) or ibuprofen (Advil) to help with pain. Always follow the doctor’s instructions for dosage.

**3. What steps should I take if the pain is more than expected or worsens? Do I need to contact my doctor to adjust my treatment plan?**

If the pain is worse than expected or doesn’t go away, here’s what to do:

1. **Check for signs of problems**: Look for redness, swelling, or fever. These could mean an infection or another issue.
2. **Adjust comfort**: Make sure the brace isn’t too tight or causing pressure sores. Reposition the child if needed.
3. **Call the doctor**: If the pain is severe, lasts a long time, or you’re worried, contact your doctor. They might need to adjust the treatment plan, like changing the brace or checking for complications after surgery.

For example, if your baby seems very fussy and won’t stop crying, or if an older child can’t move their leg properly, it’s time to call the doctor. They can help figure out what’s wrong and make sure the treatment is working as it should.

**Real-life example:**

Imagine your baby is wearing a hip brace. At first, they might cry a lot because it feels strange. You can try soothing them with a warm blanket or gentle rocking. If they’re still uncomfortable after a few days, or if you notice redness on their skin, call the doctor. They might adjust the brace or suggest other ways to make your baby more comfortable.

**What post-surgery care precautions should I take?**

After surgery for developmental dysplasia of the hip (DDH), it’s important to keep the hip safe and help it heal. Here are some key precautions:

- **Keep the wound clean and dry** to prevent infection. Follow your doctor’s instructions on how to care for the incision.
- **Avoid putting weight on the hip** unless your doctor says it’s okay. You might need crutches or a walker for a while.
- **Follow your doctor’s advice on movement**. Some kids may need a brace or cast to keep the hip in place.
- **Watch for signs of infection**, like redness, swelling, or fever. Tell your doctor right away if you notice these.

**How long after surgery can I return to normal daily activities?**

This depends on the child’s age, the type of surgery, and how well they heal. For most kids:

- **Light activities** like sitting or gentle movements can start within a few weeks.
- **Full recovery** and return to normal activities (like running or playing sports) can take several months. Your doctor will guide you on when it’s safe.

**How soon after surgery can I begin resuming activities?**

- **Right after surgery**, the focus is on rest and healing. Your child may need to stay in the hospital for a few days.
- **Gentle movements** (like wiggling toes or light stretching) can start soon after surgery, but only as recommended by the doctor.
- **Physical therapy** may begin within a few weeks to help strengthen the hip and improve movement.

**What activities are recommended during the recovery period?**

- **Physical therapy exercises** are the most important. These help the hip get stronger and more flexible.
- **Low-impact activities** like swimming (once the wound heals) or walking with support can be helpful.
- **Avoid high-impact activities** like running, jumping, or contact sports until the doctor says it’s safe.

**How do I schedule my exercise during the postoperative recovery period?**

- Start with **short, gentle exercises** a few times a day. For example, 5–10 minutes of stretching or light movements.
- Gradually increase the time and intensity as the hip gets stronger. Your physical therapist will create a plan tailored to your child’s needs.
- Always follow your doctor’s or therapist’s instructions to avoid overdoing it.

**What are the stages during postoperative recovery? What precautions should I take in each stage?**

1. **Early Stage (First Few Weeks)**:
   - Focus on rest and wound healing.
   - Avoid putting weight on the hip.
   - Watch for signs of infection or pain.
2. **Middle Stage (Weeks to Months)**:
   - Start physical therapy and gentle exercises.
   - Use crutches or a walker if needed.
   - Avoid activities that strain the hip.
3. **Late Stage (Months Later)**:
   - Gradually return to normal activities.
   - Continue strengthening exercises.
   - Follow up with the doctor to make sure the hip is healing well.

**Do I need a specialized rehabilitation plan? How does it help with recovery?**

Yes, a **specialized rehabilitation plan** is very important. It helps:

- **Restore movement** and strength in the hip.
- **Prevent stiffness** or muscle weakness.
- **Guide your child** safely back to normal activities.
  A physical therapist will create a plan based on your child’s needs and progress.

**How can I prevent recurrence after surgery?**

- **Follow the doctor’s instructions** carefully, especially about weight-bearing and activity limits.
- **Attend all follow-up appointments** to monitor the hip’s progress.
- **Continue exercises** to keep the hip strong and stable.
- **Avoid activities** that could put too much stress on the hip too soon.

**What complications can occur after surgery? What steps should I take?**

Some possible complications include:

1. **Infection**:
   - Keep the wound clean and dry.
   - Watch for redness, swelling, or fever.
   - Call the doctor if you notice these signs.
2. **Stiffness or limited movement**:
   - Do physical therapy exercises regularly.
   - Follow the therapist’s advice on stretching and movement.
3. **Dislocation or recurrence**:
   - Avoid putting too much stress on the hip.
   - Follow activity restrictions and use support devices (like braces) if needed.
4. **Pain or swelling**:
   - Use ice packs and pain relievers as recommended by the doctor.
   - Rest and avoid overusing the hip.

If you notice any unusual symptoms, contact your doctor right away. Early treatment can prevent bigger problems.

By following these steps and working closely with your doctor and therapist, your child can recover well and return to their normal activities safely!

**Developmental Dysplasia of the Hip (DDH) in Children: Recovery and Care Tips**

**1. What foods are helpful in recovery from DDH or during recovery from surgery? What foods may aggravate the condition?**

**Helpful Foods:**

- **Protein-rich foods**: Help repair tissues and muscles. Examples: eggs, chicken, fish, beans, and yogurt.
- **Calcium and vitamin D**: Strengthen bones. Examples: milk, cheese, leafy greens (like spinach), and fortified cereals.
- **Fruits and vegetables**: Provide vitamins and minerals for healing. Examples: oranges (vitamin C), carrots, and berries.
- **Whole grains**: Give energy and fiber. Examples: oatmeal, brown rice, and whole-grain bread.

**Foods to Avoid:**

- **Sugary snacks and drinks**: Can cause inflammation and slow healing.
- **Processed foods**: Often lack nutrients and may contain unhealthy fats.
- **Caffeine**: Can interfere with sleep, which is important for recovery.

**Real-life example**: Think of your body like a car. Good food is the high-quality fuel that helps it run smoothly and repair itself. Junk food is like cheap fuel—it might keep the car moving, but it won’t help it run well or fix any damage.

**2. How should I adjust my diet to prevent constipation or other common problems?**

- **Drink plenty of water**: Staying hydrated helps digestion.
- **Eat fiber-rich foods**: Keeps bowel movements regular. Examples: whole grains, fruits (like apples and pears), and vegetables (like broccoli).
- **Avoid too much dairy**: Can sometimes cause constipation in kids.
- **Small, frequent meals**: Easier on the stomach than large meals.

**Real-life example**: Imagine your digestive system like a river. Water and fiber keep the river flowing smoothly. Without them, the river gets clogged, and things don’t move well.

**3. What exercises can I do during the recovery phase? How often should I do them?**

- **Gentle stretches**: Help keep muscles flexible. Example: gently moving the legs in circles (if approved by the doctor).
- **Walking (if allowed)**: Builds strength without straining the hips.
- **Swimming (if approved)**: A low-impact way to exercise the whole body.
- **Physical therapy exercises**: A therapist may suggest specific moves to strengthen the hip.

**How often?** Start slow, like 5–10 minutes a day, and increase as the child gets stronger. Always follow the doctor’s advice.

**Real-life example**: Think of recovery like training for a sport. You start with easy drills and slowly work up to harder ones. Pushing too hard too fast can cause injuries.

**4. What precautions should I take when exercising, especially during postoperative recovery?**

- **Follow the doctor’s instructions**: Don’t do anything they haven’t approved.
- **Avoid high-impact activities**: Running or jumping can strain the hips.
- **Use support if needed**: Crutches or braces might be necessary for a while.
- **Stop if there’s pain**: Pain means the body isn’t ready for that activity yet.
- **Warm up and cool down**: Gentle stretches before and after exercise prevent injuries.

**Real-life example**: Think of the hip like a healing cut. If you pick at it or move it too much, it won’t heal properly. Gentle care helps it heal faster.

**5. Does DDH affect the quality of my sleep? What are some ways to improve sleep and help the body recover?**

**How DDH affects sleep:**

- Pain or discomfort from the hip can make it hard to sleep.
- Braces or casts used for treatment might make it uncomfortable to lie down.

**Ways to improve sleep:**

- **Comfortable sleeping position**: Use pillows to support the hips and legs.
- **Pain management**: Give prescribed pain medicine if needed (ask the doctor first).
- **Relaxing bedtime routine**: Reading or listening to soft music can help the child relax.
- **Keep the room cool and dark**: A calm environment helps with better sleep.

**Real-life example**: Think of sleep like a rechargeable battery. Good sleep helps the body “recharge” and heal faster. If the battery doesn’t charge fully, the body can’t work as well.

**Final Thought:**

Recovery from DDH or surgery takes time and care. Good food, gentle exercise, and rest are like tools that help the body heal. Always follow the doctor’s advice and take things one step at a time.

**What psychological problems may be associated with DDH or postoperative recovery, such as anxiety or depression? How can I deal with them positively?**

**Psychological Problems:**

1. **Anxiety:** Children with DDH might feel scared about doctor visits, tests, or surgery. Parents might also worry about their child’s health and future.
2. **Depression:** Long treatments or recovery can make children feel sad or frustrated. They might miss playing with friends or feel different from others.
3. **Low Self-Esteem:** Wearing a brace or cast can make kids feel self-conscious. They might think they look different or can’t do things other kids can.
4. **Frustration or Anger:** Limited movement or pain can make children irritable or upset. They might act out because they don’t know how to express their feelings.

**How to Deal with Them Positively:**

1. **Talk Openly:** Let your child express their feelings. Use simple words to explain what’s happening and why treatment is important.
2. **Stay Positive:** Focus on small wins, like progress in recovery. Celebrate milestones, like getting a smaller cast or walking again.
3. **Keep Routines:** Try to maintain normal activities as much as possible. This helps kids feel secure and less anxious.
4. **Encourage Play:** Even with limits, find fun activities your child can do. This keeps their spirits up and distracts them from discomfort.
5. **Seek Support:** Talk to other parents or join support groups. Sharing experiences can make you feel less alone.

**What are the signs that I need psychological support? How can I find professional help or support resources?**

**Signs You Need Psychological Support:**

1. **For Children:**
   - They seem sad, cry a lot, or lose interest in things they used to enjoy.
   - They have trouble sleeping, eating, or focusing.
   - They act out more than usual or seem angry all the time.
   - They say things like “I’m no good” or “I don’t want to try anymore.”
2. **For Parents:**
   - You feel overwhelmed, tired, or hopeless.
   - You’re constantly worried and can’t relax.
   - You’re having trouble sleeping or eating.
   - You feel irritable or snap at others often.

**How to Find Professional Help or Support Resources:**

1. **Talk to Your Doctor:** They can recommend a child psychologist or counselor who specializes in helping kids with medical conditions.
2. **School Resources:** If your child is in school, ask about counselors or support programs.
3. **Support Groups:** Look for local or online groups for parents of kids with DDH. Sharing experiences can help you feel less alone.
4. **Hotlines or Websites:** Many organizations offer free help. For example, in the U.S., you can call the National Alliance on Mental Illness (NAMI) helpline or visit their website.
5. **Books or Videos:** Look for child-friendly books or videos about dealing with medical conditions. These can help your child understand and cope better.

Remember, asking for help is a sign of strength, not weakness. Taking care of your mental health helps you take better care of your child!

**Developmental Dysplasia of the Hip (DDH) in Children**

**1. Are there any recommended health apps or devices that can help manage DDH or postoperative recovery?**

Yes, there are some health apps and devices that can help parents manage DDH or track their child’s recovery after surgery. These tools are not a replacement for doctor visits but can help you stay organized and monitor progress.

- **Health Apps**: Apps like **Baby Connect** or **Growth: Baby & Child Tracker** can help you log important details about your child’s hip health. For example, you can track how often your child wears their brace (a common treatment for DDH), note any discomfort, or record milestones like improved movement. Some apps also send reminders for follow-up appointments or physical therapy sessions.
- **Wearable Devices**: While there aren’t specific devices for DDH, general baby monitors or movement trackers (like **Owlet Smart Sock**) can help you keep an eye on your child’s activity levels. After surgery, you might want to ensure your child isn’t moving too much or too little, and these devices can help.
- **Physical Therapy Apps**: If your child needs physical therapy, apps like **PhysiApp** or **PT Timer** can guide you through exercises at home. These apps often include videos and timers to make it easier to follow the therapist’s instructions.

**2. How can I use health tracking tools to monitor my child’s condition or recovery progress?**

Health tracking tools can help you stay on top of your child’s DDH treatment or recovery. Here’s how you can use them:

- **Track Brace Wear Time**: If your child needs to wear a brace (like a Pavlik harness), use an app to log how many hours a day they wear it. This helps ensure they’re following the doctor’s recommendations.
- **Monitor Pain or Discomfort**: Use a simple notes app or a baby tracker app to record any signs of pain, like crying or fussiness. This information can help your doctor adjust the treatment plan if needed.
- **Record Milestones**: After surgery or during treatment, track your child’s progress. For example, note when they start crawling, walking, or moving their legs more freely. This helps you and the doctor see if the treatment is working.
- **Set Reminders**: Use your phone’s calendar or a health app to set reminders for brace adjustments, physical therapy sessions, or follow-up appointments. This ensures you don’t miss important steps in the recovery process.
- **Share Data with Your Doctor**: Bring your notes or app data to appointments. This gives the doctor a clear picture of your child’s progress and helps them make better decisions about their care.

**Real-Life Example**: Imagine your baby, Mia, has DDH and needs to wear a brace for 23 hours a day. You use a baby tracker app to log when the brace is on and off. You also record when she seems uncomfortable or when she starts moving her legs more. At her next doctor’s visit, you show the app data, and the doctor can see that Mia is improving and doesn’t need surgery.

In short, health apps and tracking tools can make it easier to manage DDH and recovery. They help you stay organized, monitor progress, and share important information with your child’s doctor.

**Accessing Reliable Information about DDH or Post-Operative Care**

1. **Talk to Your Doctor**: Your child’s doctor is the best source of information. They can explain DDH (developmental dysplasia of the hip) in simple terms and answer your questions. For example, they might say, “DDH means your child’s hip joint isn’t formed properly, but we can fix it with treatment.”
2. **Trusted Websites**: Look for information on websites like:
   - **Mayo Clinic** or **Cleveland Clinic**: These are well-known hospitals with easy-to-understand health information.
   - **American Academy of Pediatrics (AAP)**: They provide reliable info about children’s health.
   - **NHS (UK)**: If you’re in the UK, the NHS website is a great resource.

Example: If you search “DDH in kids” on the Mayo Clinic website, you’ll find clear explanations about symptoms, treatments, and what to expect after surgery.

1. **Books or Pamphlets**: Ask your doctor for brochures or books about DDH. These are often written in simple language for parents.
2. **Videos or Animations**: Sometimes, watching a video can help you understand better. For example, YouTube channels like **Nemours KidsHealth** have videos explaining DDH and treatments like braces or surgery.
3. **Ask for Written Instructions**: After surgery, ask the hospital for a simple guide on post-operative care. For example, “How to care for your child’s cast” or “Signs of infection to watch for.”

**Support Groups and Community Resources for DDH**

1. **Online Support Groups**: These are places where parents can share their experiences and ask questions. Examples include:
   - **Facebook Groups**: Search for “DDH Parents Support Group” to find other families going through the same thing.
   - **Inspire DDH Community**: An online forum where parents talk about their child’s journey with DDH.

Example: A parent might post, “My baby just got a Pavlik harness. Any tips to make them more comfortable?” Other parents can share their advice.

1. **Local Support Groups**: Some hospitals or clinics have in-person groups for parents of kids with DDH. Ask your doctor if there’s one near you.

Example: A group might meet once a month to talk about challenges, like how to keep a toddler entertained while they’re in a cast.

1. **Nonprofit Organizations**: Groups like **Steps Charity Worldwide** or **International Hip Dysplasia Institute (IHDI)** offer resources, tips, and even financial help for families dealing with DDH.

Example: IHDI has a website with videos, FAQs, and stories from other families. They also have a helpline you can call for advice.

1. **Physical Therapists**: After surgery, your child might need physical therapy. Therapists can teach you exercises to help your child’s hip get stronger. They can also connect you with other families.
2. **School or Daycare Resources**: If your child is older, talk to their school or daycare. They might have programs to help kids with mobility issues, like using a wheelchair or crutches.

**Real-Life Example**

Imagine your baby is diagnosed with DDH and needs a Pavlik harness (a soft brace). You might feel worried and have lots of questions. Here’s how you can cope:

- **Step 1**: Ask your doctor to explain how the harness works and how long your baby will need it.
- **Step 2**: Join a Facebook group for parents of kids with DDH. You can ask, “How do I bathe my baby in the harness?” Other parents will share tips.
- **Step 3**: Visit the IHDI website to watch videos about the harness and read success stories from other families.

By using these resources, you’ll feel more confident and supported as you help your child through treatment.

**What is Developmental Dysplasia of the Hip (DDH)?**

DDH is a condition where a baby’s hip joint doesn’t form properly. The hip socket may be too shallow, or the ball of the hip may not fit well into the socket. This can cause the hip to be loose or dislocated. If not treated early, it can lead to pain, limping, or arthritis later in life.

**What is the usual cost of treatment for DDH?**

The cost of treating DDH depends on how severe it is and the type of treatment needed.

1. **Mild Cases**: A special harness (like the Pavlik harness) may be used to hold the baby’s hips in the right position. This can cost between **100to100*to*500**.
2. **Moderate to Severe Cases**: If the harness doesn’t work, surgery may be needed. Surgery costs can range from **5,000to5,000*to*20,000** or more, depending on the hospital and the complexity of the surgery.
3. **Follow-Up Care**: Regular check-ups, X-rays, or physical therapy may add to the cost.

**Are there insurance or subsidy policies to reduce the financial burden?**

Yes, there are ways to reduce costs:

1. **Health Insurance**: Most health insurance plans cover DDH treatment, including surgery. Check with your insurance provider to see what’s included.
2. **Government Programs**: In many countries, programs like Medicaid (in the U.S.) or the National Health Service (NHS in the U.K.) can help cover costs.
3. **Hospital Financial Aid**: Some hospitals offer discounts or payment plans for families who can’t afford treatment.

**What social assistance can I apply for if the disease or surgery causes financial hardship?**

If DDH treatment causes financial stress, here are some options:

1. **Non-Profit Organizations**: Groups like *Shriners Hospitals for Children* or *Miracle Feet* provide free or low-cost care for children with hip problems.
2. **Government Assistance**: Programs like Temporary Assistance for Needy Families (TANF) or Supplemental Security Income (SSI) can help families with medical expenses.
3. **Local Charities**: Many communities have charities that help families pay for medical bills or transportation to hospitals.
4. **Crowdfunding**: Websites like GoFundMe can help raise money for treatment costs.

**Real-Life Example**

Imagine a family with a 6-month-old baby diagnosed with DDH. The doctor recommends a Pavlik harness, which costs 300.Their insurance covers 80300.*Their insurance covers* 8060. If surgery is needed later, the family can apply for financial aid through the hospital or a charity to reduce the cost.

**Key Takeaway**

DDH treatment can be expensive, but there are many resources to help. Always ask your doctor or hospital about insurance, subsidies, or charities that can make treatment more affordable. You’re not alone—help is available!

**How can I balance mental health, family responsibilities, and work in disease management? What are some practical suggestions?**

Managing a child’s developmental dysplasia of the hip (DDH) can feel overwhelming. But with some planning, you can balance your mental health, family life, and work. Here are some simple tips:

1. **Ask for help**: You don’t have to do everything alone. Ask family, friends, or neighbors to help with chores or babysitting. For example, if your child needs regular doctor visits, someone can help pick up your other kids from school.
2. **Take care of yourself**: Your mental health matters. Take short breaks to relax, even if it’s just 10 minutes to drink tea or take a walk. If you feel too stressed, talk to a counselor or join a support group for parents of kids with DDH.
3. **Plan ahead**: Use a calendar to organize doctor appointments, work meetings, and family time. This helps you stay on track and reduces last-minute stress.
4. **Talk to your employer**: If work feels too much, explain your situation to your boss. They might let you work flexible hours or from home on busy days.
5. **Involve your family**: Teach older kids or your partner about DDH and how they can help. For example, they can play with the child in ways that don’t hurt their hips.
6. **Celebrate small wins**: Managing DDH is a long process. Celebrate small successes, like your child’s progress in treatment or a day when everything goes smoothly.

**How might DDH affect future quality of life? How can I plan ahead to improve health?**

DDH is a condition where a baby’s hip joint doesn’t form properly. If not treated early, it can cause problems like pain, limping, or arthritis later in life. But with good care, most kids grow up to live healthy, active lives. Here’s how you can plan ahead:

1. **Start treatment early**: If DDH is caught early, treatments like a special brace (Pavlik harness) can fix the hip. Follow the doctor’s advice closely to avoid long-term issues.
2. **Regular check-ups**: Even after treatment, take your child for regular hip check-ups. This helps catch any problems early.
3. **Encourage safe activities**: As your child grows, encourage activities that are easy on the hips, like swimming or biking. Avoid high-impact sports like football until the doctor says it’s safe.
4. **Teach good habits**: Help your child maintain a healthy weight. Extra weight can put stress on the hips and increase the risk of arthritis.
5. **Plan for the future**: If DDH causes long-term issues, like arthritis, talk to the doctor about ways to manage it. This might include physical therapy, exercises, or surgery when they’re older.
6. **Stay positive**: Many kids with DDH grow up without any problems. Focus on giving your child a happy, active childhood, and they’ll likely have a great quality of life.

By staying proactive and taking small steps, you can help your child live a healthy, happy life despite DDH.

**Reply from DeepSeek-V3 (Second time)**

**What is Developmental Dysplasia of the Hip (DDH)?**

Developmental dysplasia of the hip (DDH) is a condition where a baby’s hip joint doesn’t form properly. The hip joint is like a ball (the top of the thigh bone) sitting in a socket (part of the pelvis). In DDH, the ball may be loose in the socket, partially out, or completely out of place. If not treated, it can lead to pain, walking problems, or arthritis later in life.

Think of it like a puzzle piece that doesn’t fit right. If you don’t fix it early, it can cause bigger problems as the child grows.

**Who is most likely to develop DDH? How common is it?**

DDH is more common in:

- **Firstborn babies**: The womb is tighter, which can affect hip development.
- **Girls**: They are 4–5 times more likely to have DDH than boys.
- **Babies born breech (feet first)**: This position puts extra pressure on the hips.
- **Family history**: If a parent or sibling had DDH, the risk is higher.

About 1–3 in 1,000 babies have DDH that needs treatment. It’s not super common, but it’s important to catch it early.

**Will DDH have a long-term impact on my quality of life? What aspects should I pay special attention to?**

If treated early, most children with DDH grow up with normal hip function and no long-term problems. However, if it’s not treated, it can lead to:

- **Pain**: The hip may hurt during activities like walking or running.
- **Limping**: The child might walk unevenly.
- **Arthritis**: This can happen earlier in life, causing stiffness and pain.

**What to pay attention to:**

1. **Early signs**: Look for uneven leg lengths, less movement in one leg, or uneven skin folds on the thighs.
2. **Regular check-ups**: Doctors check babies’ hips during routine visits. Don’t skip these!
3. **Treatment**: If DDH is found, follow the doctor’s plan. This might include a special harness, brace, or, in rare cases, surgery.

Think of it like fixing a wobbly table leg. If you tighten it early, the table works fine. But if you wait too long, it might break.

**Real-life example:**

Imagine a baby named Mia. Her parents notice one leg seems shorter, and her diaper changes are harder on one side. The doctor checks and finds DDH. Mia wears a special harness for a few months. By the time she starts walking, her hip is normal, and she runs and plays like any other kid.

Catching DDH early makes all the difference!

**What parts of the body does developmental dysplasia of the hip (DDH) mainly affect? What do these parts do in a healthy state?**

**Parts Affected:**
DDH mainly affects the hip joint. The hip joint is where the thigh bone (femur) connects to the pelvis. In a healthy hip, the top of the thigh bone (the "ball") fits snugly into a socket in the pelvis (the "cup"). This allows the leg to move smoothly in many directions, like when you walk, run, or sit.

**What These Parts Do in a Healthy State:**

- The **ball and socket** work together to support your body weight.
- They let your leg move freely without pain.
- They keep your body stable when you stand or move.

Think of the hip joint like a door hinge. If the hinge is aligned properly, the door opens and closes smoothly. But if the hinge is loose or misaligned, the door won’t work well.

**What symptoms or consequences may arise if these parts are damaged? Can you provide specific examples?**

**Symptoms of DDH:**

1. **Uneven Legs:** One leg may look shorter than the other because the hip joint isn’t in the right place.
2. **Limited Movement:** The child may not be able to move their leg as freely as usual. For example, they might have trouble spreading their legs apart during diaper changes.
3. **Limping or Waddling:** When the child starts walking, they might limp or walk with a waddling gait because the hip isn’t stable.
4. **Clicking or Popping Sounds:** Sometimes, parents might hear a clicking sound when moving the baby’s hip.

**Consequences if Untreated:**

- **Pain:** As the child grows, the misaligned hip can cause pain, especially during physical activities.
- **Arthritis:** Over time, the joint may wear out faster, leading to early arthritis (joint pain and stiffness).
- **Uneven Walking:** The child might walk awkwardly, which can affect their posture and cause back or knee problems later in life.

**Real-Life Example:**
Imagine a baby named Mia. Her parents notice that one of her legs seems shorter, and her diaper changes are harder because her legs don’t spread easily. When Mia starts walking, she limps and seems unbalanced. If her parents don’t get her checked, Mia might have trouble running or playing sports as she grows older, and her hip could hurt a lot as an adult.

But if DDH is caught early, doctors can use treatments like a special brace to help the hip grow correctly. This way, Mia can grow up to walk, run, and play without pain.

In short, DDH affects the hip joint, which is crucial for movement. If not treated, it can cause pain and mobility issues. But with early care, most children can grow up just fine!

**What are the main causes of DDH? What risk factors may increase the chances of developing it?**

**Main Causes:**
Developmental Dysplasia of the Hip (DDH) happens when the hip joint doesn’t form properly. The hip is a ball-and-socket joint. In DDH, the "ball" (top of the thigh bone) doesn’t fit well into the "socket" (hip bone). This can happen because the socket is too shallow or the ligaments are too loose.

**Risk Factors:**

1. **Family History:** If someone in your family had DDH, your child might be more likely to have it.
2. **Breech Birth:** Babies born bottom-first (breech) are at higher risk.
3. **Firstborn Girls:** Girls and firstborn babies are more likely to have DDH.
4. **Swaddling Incorrectly:** Tight swaddling with legs straight can increase the risk.

**What lifestyle habits or environmental factors increase the risk of developing DDH? How can I prevent it?**

**Risk Factors:**

1. **Swaddling:** Swaddling a baby too tightly, especially with their legs straight, can push the hips out of place.
2. **Cradle Positioning:** Using baby carriers or seats that keep the legs together for long periods can increase the risk.

**Prevention:**

1. **Proper Swaddling:** If you swaddle, leave room for the baby’s legs to bend and move.
2. **Hip-Friendly Carriers:** Use carriers that support the baby’s thighs and keep their hips spread apart.
3. **Regular Checkups:** Get your baby’s hips checked during routine doctor visits, especially if they have risk factors.

**If I have a family history, is my risk of developing DDH higher? What preventive measures should I take?**

**Family History and Risk:**
Yes, if someone in your family had DDH, your child’s risk is higher. It doesn’t mean they’ll definitely have it, but they’re more likely to.

**Preventive Measures:**

1. **Tell Your Doctor:** Let your pediatrician know about your family history. They can monitor your baby’s hips more closely.
2. **Hip Exams:** Make sure your baby gets regular hip exams during checkups.
3. **Avoid Tight Swaddling:** Always swaddle with the legs loose and able to move.
4. **Early Treatment:** If DDH is found early, it’s easier to treat with braces or other methods.

**Real-Life Example:**

Imagine a baby named Mia. Her mom had DDH as a child. Mia’s mom tells the doctor about this during her first checkup. The doctor checks Mia’s hips and notices they’re a little loose. Mia gets a special brace to wear for a few months. This helps her hips grow properly, and she avoids bigger problems later.

By being aware of risk factors and taking simple steps, parents can help their kids avoid or manage DDH.

**What are the main symptoms of Developmental Dysplasia of the Hip (DDH) in children?**

Developmental Dysplasia of the Hip (DDH) is a condition where a baby’s hip joint doesn’t form properly. Here are some common signs to look for:

1. **Uneven leg lengths**: One leg may look shorter than the other.
2. **Uneven skin folds**: The folds on the thighs or buttocks may look different on each side.
3. **Limited movement**: The hip may not move as freely as it should. For example, one leg might not spread out as far as the other.
4. **Clicking or popping sound**: You might hear a clicking or popping noise when moving the hip.
5. **Limping or walking oddly**: When the child starts walking, they might limp or walk on their toes.

**What signs indicate the disease is worsening?**

If DDH isn’t treated, it can get worse. Here are some signs that the condition might be worsening:

1. **Increased pain**: The child might start to feel pain in the hip, groin, or knee.
2. **Difficulty walking**: Walking might become harder, and the limp might get more noticeable.
3. **Less mobility**: The hip might become stiffer, making it harder to move the leg.
4. **Uneven wear on shoes**: One shoe might wear out faster than the other because of the way the child walks.

**Should I seek immediate medical attention?**

Yes, if you notice any of these signs, it’s important to see a doctor as soon as possible. Early treatment can help prevent more serious problems later on. Here are some situations when you should seek immediate medical attention:

1. **Noticeable limp**: If your child starts limping or walking differently.
2. **Pain**: If your child complains of pain in the hip, groin, or knee.
3. **Limited movement**: If your child’s leg doesn’t move as freely as it should.
4. **Uneven legs or skin folds**: If you notice one leg is shorter or the skin folds don’t match.

**Real-life example**

Imagine you have a baby who is starting to walk. You notice that one leg seems shorter, and the skin folds on their thighs don’t look the same. When they walk, they limp a little. These could be signs of DDH. If you see these signs, it’s a good idea to take your child to the doctor. The doctor might do some tests, like an ultrasound or X-ray, to check the hip. If DDH is found early, treatments like a special brace can help the hip develop normally. If it’s not treated, the child might have more pain and trouble walking as they grow older.

In short, if you notice any unusual signs in your child’s hips or walking, it’s best to get it checked out right away. Early treatment can make a big difference.

**What methods do doctors usually use to confirm the diagnosis of DDH? What is the purpose of each test?**

1. **Physical Exam**:
   Doctors check the baby’s hips during regular check-ups. They move the legs to see if the hip joint is loose or dislocated. This is the first step to spot any problems.
2. **Ultrasound**:
   This test uses sound waves to create pictures of the baby’s hip joint. It’s safe and doesn’t hurt. Doctors use it to see if the hip is properly formed or if it’s dislocated. It’s often used for babies under 6 months old.
3. **X-ray**:
   For older babies (over 6 months), doctors may use X-rays. X-rays show clear images of the bones. They help doctors see if the hip socket is shallow or if the hip is out of place.

**Purpose of these tests**:

- To find out if the hip joint is normal or not.
- To see how severe the problem is.
- To decide the best treatment.

**What indicators are most important in test results? How do these results reflect the severity of the disease?**

1. **Hip Stability**:
   If the hip is loose or dislocated, it’s a sign of DDH. The more unstable the hip, the worse the condition.
2. **Shape of the Hip Socket**:
   A shallow hip socket (called dysplasia) means the hip isn’t forming right. A deeper socket is better.
3. **Position of the Femur (Thigh Bone)**:
   If the thigh bone is not sitting properly in the hip socket, it shows the hip is dislocated.

**How these results reflect severity**:

- Mild DDH: The hip is loose but not dislocated.
- Moderate DDH: The hip is partially dislocated.
- Severe DDH: The hip is fully dislocated, and the socket is very shallow.

**After confirming the diagnosis of DDH, do I need to schedule regular follow-ups? How often should I have them and what should be checked?**

Yes, regular follow-ups are very important. DDH needs to be monitored closely to make sure the treatment is working and the hip is developing properly.

1. **Frequency of Follow-ups**:
   - For babies in a brace or harness: Check every 2–4 weeks.
   - After treatment (like surgery or casting): Check every 3–6 months for a few years.
   - As the child grows: Yearly check-ups until the hip is fully developed (around age 5–6).
2. **What’s Checked During Follow-ups**:
   - Hip movement and stability.
   - X-rays or ultrasounds to see how the hip is growing.
   - Signs of pain or walking problems (for older kids).

**Why follow-ups matter**:

- To make sure the hip is healing and growing correctly.
- To catch any new problems early.
- To adjust treatment if needed.

**Real-Life Example:**

Imagine a baby named Mia. At her 2-month check-up, the doctor notices her left hip is loose. An ultrasound confirms DDH. Mia wears a special harness for 3 months. Her parents take her for check-ups every 2 weeks. The doctor checks her hip and does ultrasounds to see if it’s improving. After 3 months, Mia’s hip is stable, and she only needs check-ups every 6 months. By age 2, her hip is normal, and she can run and play like other kids.

Regular check-ups and early treatment helped Mia grow up healthy!

**What symptoms indicate that I need to seek immediate medical attention?**

If you notice any of the following signs in your child, it’s important to see a doctor right away:

1. **Uneven leg lengths**: One leg looks shorter than the other when your child is lying down.
2. **Uneven skin folds**: The folds on the thighs or buttocks don’t match on both sides.
3. **Limited movement**: Your child has trouble moving one leg or hip as much as the other.
4. **Clicking or popping sounds**: You hear or feel a clicking or popping when moving your child’s hip.
5. **Limping or walking oddly**: If your child is old enough to walk, they might limp or walk on their toes.

For example, if you’re changing your baby’s diaper and notice one leg doesn’t move as easily as the other, or if your toddler starts limping suddenly, these could be signs of DDH.

**How should I handle and self-monitor during an emergency situation?**

If you suspect DDH, here’s what you can do:

1. **Stay calm**: Keep your child comfortable and avoid moving their hips too much.
2. **Monitor symptoms**: Keep an eye on the symptoms mentioned above. Write down what you notice so you can tell the doctor.
3. **Avoid pressure on the hips**: Don’t force your child’s legs into any position that seems uncomfortable.
4. **Seek medical help**: Call your doctor or go to the emergency room if the symptoms seem severe or if your child is in pain.

For example, if your baby seems to be in pain when you move their legs, stop moving them and call your doctor. If your toddler is limping and complaining of hip pain, take them to the doctor as soon as possible.

Remember, early treatment can make a big difference, so don’t wait if you notice any of these signs.

**What treatments will I receive during hospitalization? What is the purpose of each treatment?**

1. **Pavlik Harness**:
   - This is a soft brace that helps keep the baby’s hips in the right position.
   - **Purpose**: It gently moves the hip joint into place so it can grow normally.
   - *Example*: Think of it like a seatbelt that guides the hip into the correct spot over time.
2. **Casting (Spica Cast)**:
   - If the harness doesn’t work, a hard cast might be used to hold the hips in place.
   - **Purpose**: It keeps the hips stable so they can heal properly.
   - *Example*: Like a plaster cast for a broken arm, but for the hips.
3. **Surgery**:
   - In severe cases, a doctor might need to fix the hip joint with surgery.
   - **Purpose**: To reposition the hip and make sure it stays in place.
   - *Example*: Like fixing a puzzle piece that’s in the wrong spot.
4. **Physical Therapy**:
   - Gentle exercises to help the hip move and strengthen.
   - **Purpose**: To improve flexibility and muscle strength around the hip.
   - *Example*: Like stretching and moving your legs to keep them strong.

**How can I prevent infections or other complications during hospitalization? What can family members do to assist with recovery?**

1. **Prevent Infections**:
   - Wash hands often with soap and water.
   - Keep the hospital room clean.
   - Follow the doctor’s instructions for caring for braces, casts, or wounds.
   - *Example*: Treat the hospital room like your kitchen—keep it tidy and clean to avoid germs.
2. **Family Assistance**:
   - Help with daily care, like feeding, bathing, and changing diapers.
   - Learn how to use the Pavlik harness or care for the cast properly.
   - Keep the baby comfortable and calm.
   - *Example*: Family members are like a support team, making sure the baby is happy and healthy.

**How can I reinforce the effects of treatment after discharge? What rehabilitation programs are recommended?**

1. **Follow-Up Appointments**:
   - Regular check-ups with the doctor to monitor progress.
   - **Purpose**: To make sure the hip is growing correctly.
   - *Example*: Like taking your car for a tune-up to keep it running smoothly.
2. **Home Exercises**:
   - Simple stretches or movements recommended by the doctor or physical therapist.
   - **Purpose**: To keep the hip flexible and strong.
   - *Example*: Like doing a few stretches every day to stay healthy.
3. **Wearing Braces or Casts**:
   - Continue using any devices as instructed by the doctor.
   - **Purpose**: To keep the hip in the right position while it heals.
   - *Example*: Like wearing a retainer after braces to keep teeth straight.
4. **Physical Therapy Sessions**:
   - Attend therapy sessions to improve movement and strength.
   - **Purpose**: To help the hip work normally again.
   - *Example*: Like going to a coach to practice and get better at a sport.

By following these steps, you can help your child’s hip heal properly and prevent future problems.

**Developmental Dysplasia of the Hip (DDH) in Children**

**1. What are the common medications used to treat DDH? How should I take them correctly?**

- **Medications:** DDH is not typically treated with medications. Instead, it is managed with physical methods like braces, harnesses, or sometimes surgery. Medications might only be used if the child needs pain relief or has an infection, but these are rare cases.
- **How to take them:** If a doctor prescribes pain relief (like acetaminophen or ibuprofen), follow the instructions carefully. Give the right dose at the right time. Always use the measuring tool that comes with the medicine, not a kitchen spoon.

**2. What should I do if I forget to give my child their medication or give it at the wrong time? Will it affect the treatment?**

- **If you forget:** If the medication is for pain and you miss a dose, give it as soon as you remember. If it’s almost time for the next dose, skip the missed one. Don’t double up. Missing one dose of pain medication usually won’t affect the overall treatment of DDH.
- **Impact on treatment:** Since DDH treatment mainly relies on braces or surgery, missing a dose of pain medication won’t change the outcome of the hip correction. Just try to stay on schedule as much as possible.

**3. What foods should I avoid while giving my child medication for DDH? Are there common side effects?**

- **Foods to avoid:** If your child is taking pain medication like ibuprofen, avoid giving them sugary or acidic drinks (like soda or orange juice) right before or after the medicine. These can upset their stomach. Always give the medicine with food or milk to protect their stomach.
- **Common side effects:** Pain medications can sometimes cause stomach upset, nausea, or drowsiness. If your child seems unusually sleepy or complains of a stomachache, let the doctor know. For DDH treatment with braces or harnesses, side effects are usually related to skin irritation or discomfort from the device, not from medications.

**Real-Life Example:**

Imagine your child is wearing a special harness to correct their hip. They might feel a little uncomfortable, so the doctor suggests giving them ibuprofen to help with the pain. You give the medicine with a small snack, like crackers, to avoid stomach issues. If you forget one dose, it’s okay—just give the next one on time. The harness is doing the main work to fix the hip, so the medicine is just there to keep your child comfortable.

**1. What types of pain may be caused by DDH or its treatment? Where is the pain usually felt?**

Developmental Dysplasia of the Hip (DDH) is a condition where a baby’s hip joint doesn’t form properly. The hip joint is where the thigh bone (femur) connects to the pelvis. In DDH, the hip can be loose, partially dislocated, or fully dislocated. This can cause pain in the hip area, groin, or even the thigh. The pain might feel like a dull ache or sharp discomfort, especially when moving the leg.

If a child is being treated for DDH, they might wear a special brace or harness (like the Pavlik harness) to keep the hip in the right position. This can sometimes cause discomfort because it limits movement or puts pressure on the skin. After surgery (if needed), there might be pain around the hip or where the incision was made.

**2. What methods can help relieve pain? What are the recommended pharmacological and non-pharmacological treatments?**

**Non-Pharmacological Methods (Without Medicine):**

- **Bracing or Harness:** If the child is wearing a brace, adjusting it properly can help reduce discomfort. Soft padding can also be used to prevent skin irritation.
- **Gentle Movement:** Physical therapy or gentle exercises (if recommended by the doctor) can help keep the hip flexible and reduce stiffness.
- **Heat or Cold Packs:** A warm towel or cold pack (wrapped in a cloth) can soothe sore muscles around the hip.
- **Comfortable Positioning:** Keeping the child in a comfortable position, like lying on their back with legs slightly apart, can help.

**Pharmacological Methods (With Medicine):**

- **Pain Relievers:** Doctors might recommend over-the-counter pain relievers like acetaminophen (Tylenol) or ibuprofen (Advil) for older children. Always follow the doctor’s instructions for dosage.
- **Prescription Medications:** For severe pain after surgery, the doctor might prescribe stronger painkillers for a short time.

**3. What steps should I take if the pain is more than expected or worsens? Do I need to contact my doctor to adjust my treatment plan?**

If the pain is worse than expected or doesn’t improve, here’s what to do:

1. **Check for Signs of Problems:** Look for redness, swelling, or warmth around the hip. These could be signs of infection or another issue.
2. **Adjust the Brace or Harness:** If the child is wearing a brace, make sure it’s not too tight or causing pressure sores. If it’s uncomfortable, contact the doctor for advice.
3. **Stop Activities That Make It Worse:** If certain movements or positions increase the pain, avoid them until you talk to the doctor.
4. **Contact the Doctor:** If the pain is severe, doesn’t go away, or you’re worried, call the doctor. They might need to adjust the treatment plan, check for complications, or change medications.

For example, if your child had surgery and the pain gets worse instead of better after a few days, this could mean something isn’t healing right. The doctor might need to see them to make sure everything is okay.

**Real-Life Example:**

Imagine your baby is wearing a Pavlik harness to treat DDH. At first, they seem fine, but after a few days, they start crying more and seem uncomfortable. You notice redness where the harness touches their skin. This could mean the harness is too tight or rubbing. You’d want to call the doctor to adjust the harness and check for any skin irritation.

In short, always keep an eye on your child’s pain and comfort. If something doesn’t seem right, don’t hesitate to reach out to the doctor. They’re there to help!

**What post-surgery care precautions should I take?**

After surgery for developmental dysplasia of the hip (DDH), it’s important to keep the area clean and dry to prevent infection. Follow your doctor’s instructions on how to care for the wound. Avoid putting weight on the hip unless your doctor says it’s okay. Use crutches or a walker if needed. Keep your child from rough play or activities that could hurt the hip.

**How long after surgery can I return to normal daily activities?**

It depends on the surgery and how well your child heals. Usually, it takes a few weeks to a few months. Your doctor will tell you when it’s safe to go back to normal activities like walking, running, or playing.

**How soon after surgery can I begin resuming activities?**

Light activities, like moving the leg gently, might start right after surgery. But more active things, like walking or playing, will take longer. Your doctor will give you a plan based on how your child is healing.

**What activities are recommended during the recovery period?**

During recovery, gentle movements and physical therapy exercises are best. These help keep the hip flexible and strong. Swimming or water therapy can also be good because it’s easy on the joints. Avoid high-impact activities like jumping or running until your doctor says it’s okay.

**How do I schedule my exercise during the postoperative recovery period?**

Start with simple exercises, like moving the hip gently, as soon as your doctor allows. Gradually increase the intensity and time as your child gets stronger. Follow the physical therapist’s plan and don’t push too hard. Rest is just as important as exercise.

**What are the stages during postoperative recovery? What precautions should I take in each stage?**

1. **Early Stage (0-2 weeks):** Focus on rest and wound care. Avoid putting weight on the hip. Use pain medicine as needed.
2. **Middle Stage (2-6 weeks):** Start gentle exercises and physical therapy. Still avoid heavy activities.
3. **Late Stage (6 weeks and beyond):** Gradually return to normal activities. Keep doing exercises to strengthen the hip.

In each stage, follow your doctor’s advice and don’t rush the process.

**Do I need a specialized rehabilitation plan? How does it help with recovery?**

Yes, a rehabilitation plan is important. It helps your child regain strength and movement in the hip. A physical therapist will create a plan tailored to your child’s needs. This plan helps prevent stiffness and improves healing.

**How can I prevent recurrence after surgery?**

To prevent DDH from coming back, follow your doctor’s advice closely. Keep up with physical therapy and exercises. Make sure your child avoids activities that could strain the hip. Regular check-ups with the doctor are also important to catch any problems early.

**What complications can occur after surgery? What steps should I take?**

Complications can include infection, blood clots, or the hip dislocating again. Watch for signs like redness, swelling, or fever. Call your doctor if you notice these. Follow all post-surgery instructions to reduce the risk of complications.

By following these steps and working closely with your doctor, your child can have a smooth recovery and get back to normal activities safely.

**Developmental Dysplasia of the Hip (DDH) in Children**

**1. What foods are helpful in recovery from DDH or during recovery from surgery? What foods may aggravate the condition?**

- **Helpful Foods**:
  - **Protein-rich foods**: Like eggs, chicken, fish, and beans. These help muscles and tissues heal after surgery.
  - **Calcium-rich foods**: Like milk, cheese, yogurt, and leafy greens. These strengthen bones.
  - **Vitamin D foods**: Like fortified cereals, fatty fish (salmon), and egg yolks. Vitamin D helps the body absorb calcium.
  - **Fruits and vegetables**: Like oranges, strawberries, and broccoli. These provide vitamins and antioxidants that help the body heal.
- **Foods to Avoid**:
  - **Sugary snacks and drinks**: Like candy and soda. Too much sugar can cause inflammation and slow healing.
  - **Processed foods**: Like chips and fast food. These are low in nutrients and can make recovery harder.

**Real-life example**: After surgery, a child could have a breakfast of scrambled eggs (protein) with a glass of milk (calcium) and some orange slices (vitamin C). Avoid giving them sugary cereal or soda.

**2. How should I adjust my diet to prevent constipation or other common problems?**

- **Fiber-rich foods**: Like whole grains (oatmeal, whole wheat bread), fruits (apples, pears), and vegetables (carrots, peas). Fiber helps prevent constipation.
- **Hydration**: Make sure the child drinks plenty of water throughout the day. Water helps fiber work better and keeps stools soft.
- **Small, frequent meals**: Instead of heavy meals, offer smaller portions more often. This helps digestion and prevents discomfort.

**Real-life example**: If a child is recovering from DDH surgery, offer them a snack like whole-grain crackers with peanut butter and a glass of water instead of heavy, greasy foods.

**3. What exercises can I do during the recovery phase? How often should I do them?**

- **Gentle movements**: After surgery, the doctor may recommend gentle exercises to keep the hip joint moving. These are usually done with the help of a physical therapist.
- **Range-of-motion exercises**: These are slow, controlled movements to improve flexibility. For example, moving the leg in small circles.
- **Strengthening exercises**: Once healing progresses, light exercises to strengthen the muscles around the hip may be added.

**How often**: The doctor or therapist will guide this, but usually, exercises are done a few times a day for short periods (5-10 minutes).

**Real-life example**: A child might lie on their back and slowly move their leg in a small circle, guided by a parent or therapist, 2-3 times a day.

**4. What precautions should I take when exercising, especially during postoperative recovery?**

- **Follow the doctor’s instructions**: Never push the child to do more than what’s recommended.
- **Avoid high-impact activities**: Like running or jumping. These can strain the hip.
- **Use support if needed**: A brace or crutches may be needed to protect the hip during recovery.
- **Watch for pain**: If the child feels pain during exercise, stop immediately and tell the doctor.

**Real-life example**: If a child is using crutches after surgery, make sure they don’t try to walk without them too soon. Always check with the doctor before starting any new activity.

**5. Does DDH affect the quality of my sleep? What are some ways to improve sleep and help the body recover?**

- **Sleep impact**: Yes, DDH or surgery can make it hard for a child to sleep comfortably, especially if they’re in a cast or brace.
- **Improving sleep**:
  - **Comfortable position**: Use pillows to support the child’s body and keep the hip in a neutral position.
  - **Pain management**: Give prescribed pain medicine before bedtime if the doctor recommends it.
  - **Relaxing routine**: A warm bath or quiet story before bed can help the child relax.
  - **Dark, quiet room**: Make sure the sleeping environment is calm and free from distractions.

**Real-life example**: If a child is in a hip brace, place a pillow under their knees to keep their legs slightly bent. This can make sleeping more comfortable.

By focusing on good nutrition, gentle exercises, and proper sleep, a child recovering from DDH can heal better and faster. Always follow the doctor’s advice for the best results!

**Psychological Problems Associated with DDH and Postoperative Recovery**

**1. Anxiety and Depression:**

- **Anxiety:** Children with DDH might feel anxious about wearing a brace or cast. They might worry about looking different or not being able to play like other kids. Parents might also feel anxious about their child’s health and future.
- **Depression:** Long-term treatment or surgery can make a child feel sad or frustrated. They might miss out on activities and feel isolated. Parents might also feel down if they see their child struggling.

**Real-life Example:** Imagine a 5-year-old girl who loves to dance. She has to wear a special brace for DDH and can’t join her dance class. She might feel left out and sad. Her parents might feel stressed about her missing out and worry about her recovery.

**2. How to Deal with These Feelings Positively:**

- **Talk About It:** Encourage the child to express their feelings. Parents should also share their concerns with family or friends.
- **Stay Active:** Find activities the child can do while wearing a brace or cast. This keeps them engaged and happy.
- **Positive Reinforcement:** Praise the child for being brave and strong. This boosts their confidence.
- **Professional Help:** Sometimes, talking to a counselor can help both the child and parents cope better.

**Signs You Need Psychological Support**

**1. For the Child:**

- **Behavior Changes:** If the child becomes very quiet, angry, or starts acting out, it might be a sign they need help.
- **Sleep Problems:** Trouble sleeping or frequent nightmares can indicate stress.
- **Loss of Interest:** If the child stops enjoying things they used to love, it could be a sign of depression.

**2. For Parents:**

- **Constant Worry:** If you find yourself always worried and unable to relax, you might need support.
- **Feeling Overwhelmed:** If daily tasks feel too hard, or you feel very sad or angry, it’s a sign to seek help.
- **Isolation:** If you feel alone and don’t want to talk to anyone, it’s important to reach out.

**How to Find Professional Help or Support Resources**

**1. Talk to Your Doctor:**

- Your child’s doctor can recommend a psychologist or counselor who specializes in children’s health.

**2. School Resources:**

- Schools often have counselors who can help children cope with their feelings.

**3. Support Groups:**

- Look for local or online support groups for parents of children with DDH. Sharing experiences can be comforting.

**4. Mental Health Hotlines:**

- Many countries have hotlines where you can talk to someone about your feelings. They can guide you to the right resources.

**Real-life Example:** A mom notices her son is very quiet and doesn’t want to play with his friends after his DDH surgery. She talks to his doctor, who recommends a child psychologist. The psychologist helps the boy express his feelings and suggests fun activities he can do while recovering. The mom also joins a support group where she meets other parents going through the same thing.

By recognizing the signs and seeking help, both children and parents can better cope with the challenges of DDH and its treatment.

**1. Are there any recommended health apps or devices that can help manage DDH or postoperative recovery?**

For children with **developmental dysplasia of the hip (DDH)**, there aren’t many specific apps or devices just for this condition. But some general health tools can help parents manage their child’s care and recovery after surgery. Here are a few examples:

- **Baby Tracker Apps**: Apps like *Baby Tracker* or *Huckleberry* can help parents log their child’s daily activities, like feeding, sleeping, and mood. After DDH surgery, tracking these can help spot any changes in the child’s comfort or behavior.
- **Physical Therapy Apps**: Apps like *PhysiApp* or *PT Timer* can guide parents through simple exercises or stretches recommended by a physical therapist. These exercises are often part of recovery after DDH treatment.
- **Wearable Devices**: For older kids, fitness trackers like *Fitbit* can monitor activity levels. This is helpful after surgery to make sure the child is moving enough but not overdoing it.
- **Pain Management Apps**: Apps like *PainScale* let parents track their child’s pain levels and share this info with the doctor. This is especially useful after surgery to ensure the child is comfortable.

**2. How can I use health tracking tools to monitor my condition or recovery progress?**

If your child has DDH or is recovering from surgery, tracking tools can help you stay on top of their progress. Here’s how:

- **Track Symptoms**: Use a notebook or app to write down any signs of discomfort, like limping, crying during diaper changes, or trouble moving their legs. This helps the doctor understand how your child is doing.
- **Monitor Movement**: After surgery, your child might need to wear a brace or cast. Use a fitness tracker (if age-appropriate) to make sure they’re moving enough but not too much. For babies, you can log how often they kick or move their legs.
- **Record Physical Therapy Progress**: If your child is doing physical therapy, use an app to track their exercises and improvements. For example, note if they’re gaining more flexibility or strength in their hips.
- **Share with the Doctor**: Bring your notes or app data to doctor visits. This helps the doctor see how your child is recovering and adjust the treatment plan if needed.

**Real-Life Example:**

Imagine your baby has DDH and just had surgery. You use a baby tracker app to log their sleep, feeding, and mood. You notice they’re fussier than usual and not moving their legs much. You share this with the doctor, who adjusts their pain medication and suggests gentle leg exercises. Over time, you see improvement in their movement and mood, and the app helps you stay organized and informed.

By using these tools, you can play an active role in your child’s recovery and make sure they’re on the right track!

**1. How to Find Reliable Information About Developmental Dysplasia of the Hip (DDH) or Post-Operative Care**

When looking for information about DDH or how to care for your child after surgery, it’s important to use trusted sources. Here are some ways to find reliable information:

- **Ask Your Doctor or Healthcare Team**: They can give you the most accurate and personalized advice. For example, if your child needs a brace or cast after surgery, they can explain how to care for it.
- **Reputable Medical Websites**: Websites like the **American Academy of Pediatrics (AAP)**, **Mayo Clinic**, or **NHS (UK)** have easy-to-understand information about DDH. These sites are written by experts and reviewed for accuracy.
- **Children’s Hospitals**: Many children’s hospitals, like **Boston Children’s Hospital** or **Children’s Hospital of Philadelphia**, have online resources about DDH. These are great because they specialize in kids’ health.
- **Books or Pamphlets**: Your doctor might give you brochures or recommend books about DDH. These are often written in simple language for parents.
- **Avoid Dr. Google**: Random websites or forums can have wrong or scary information. Stick to trusted sources to avoid confusion.

**2. Are There Support Groups or Community Resources to Help My Family Cope?**

Yes! Dealing with DDH can feel overwhelming, but you’re not alone. Here are some ways to find support:

- **Local Support Groups**: Some hospitals or clinics have groups for parents of kids with DDH. These groups let you share experiences and tips. For example, you might learn how other parents handle wearing a Pavlik harness (a special brace for DDH).
- **Online Communities**: Websites like **Facebook** or **Reddit** have groups for parents of kids with DDH. These can be helpful for asking questions or getting advice from people who’ve been through the same thing.
- **Nonprofit Organizations**: Groups like **Steps Charity** (in the UK) or **International Hip Dysplasia Institute** (global) offer resources, support, and even events for families dealing with DDH.
- **Physical Therapists or Social Workers**: If your child needs therapy after surgery, the therapist can help you learn exercises or connect you with resources. Social workers at the hospital can also help with emotional or financial support.
- **Talk to Other Parents**: Sometimes, just talking to someone who understands can make a big difference. For example, if your child is in a cast, another parent might have tips for keeping them comfortable.

**Real-Life Example**

Imagine your baby is diagnosed with DDH and needs to wear a Pavlik harness. You might feel worried or unsure. By talking to your doctor, you learn how to put on the harness correctly. You also join an online group where other parents share tips, like how to dress your baby comfortably over the harness. This support helps you feel more confident and less alone.

**What is Developmental Dysplasia of the Hip (DDH)?**

Developmental Dysplasia of the Hip (DDH) is a condition where a baby’s hip joint doesn’t form properly. The hip joint is like a ball (the top of the thigh bone) that fits into a socket (part of the pelvis). In DDH, the ball may be loose in the socket or even completely out of place. If not treated early, it can cause pain, walking problems, or arthritis later in life.

**What is the usual cost of treatment for DDH?**

The cost of treating DDH depends on how severe it is and the type of treatment needed. Here’s a rough idea:

1. **Mild Cases (Pavlik Harness):**
   - A Pavlik harness is a soft brace that holds the baby’s hips in the right position.
   - Cost: Around 100to100*to*300.
   - This is often the first treatment and works well for many babies.
2. **Moderate Cases (Casting or Surgery):**
   - If the harness doesn’t work, the baby might need a cast or minor surgery to fix the hip.
   - Cost: 2,000to2,000*to*5,000, depending on the hospital and location.
3. **Severe Cases (Major Surgery):**
   - In rare cases, older children may need more complex surgery.
   - Cost: 10,000to10,000*to*20,000 or more, including hospital stays and follow-up care.

**Are there insurance or subsidy policies to reduce the financial burden?**

Yes, there are ways to reduce costs:

1. **Health Insurance:**
   - Most health insurance plans cover DDH treatment, including braces, casts, and surgeries.
   - Check with your insurance provider to see what’s covered and if there are any out-of-pocket costs.
2. **Government Programs (e.g., Medicaid in the U.S.):**
   - If your family has a low income, you might qualify for Medicaid or similar programs.
   - These programs often cover the full cost of treatment.
3. **Hospital Financial Aid:**
   - Many hospitals offer financial assistance or payment plans for families who can’t afford treatment.
   - Ask the hospital’s billing department about options.

**What social assistance can I apply for if the disease or surgery causes financial hardship?**

If DDH treatment causes financial stress, here are some options:

1. **Non-Profit Organizations:**
   - Some charities help families with medical costs. For example, the *Shriners Hospitals for Children* provides free or low-cost care for children with orthopedic conditions like DDH.
2. **Government Assistance Programs:**
   - Programs like **WIC (Women, Infants, and Children)** or **SNAP (food stamps)** can help with basic needs, freeing up money for medical expenses.
   - **Temporary Assistance for Needy Families (TANF)** provides cash aid to families in need.
3. **Local Community Support:**
   - Churches, community centers, or local charities may offer help with bills, transportation, or other needs.
4. **Crowdfunding:**
   - Websites like GoFundMe allow families to raise money for medical expenses. Many people are willing to help if they know your story.

**Real-Life Example:**

Imagine a family with a 6-month-old baby diagnosed with DDH. The doctor recommends a Pavlik harness, which costs 200.The family has insurance, so they only pay 200.*The family has insurance*, *so they only pay*20. But if the baby needs surgery later, the cost could be $5,000. The family applies for Medicaid and gets approved, so they don’t have to pay anything. They also reach out to a local charity for help with groceries, so they can focus on their baby’s recovery.

**Key Takeaways:**

- DDH treatment costs vary, but insurance or government programs can help.
- If you’re struggling financially, ask about hospital aid, non-profits, or community support.
- Early treatment is important and can prevent bigger problems (and costs) later.

Let me know if you need more help!

**How can I balance mental health, family responsibilities, and work in disease management? What are some practical suggestions?**

Balancing mental health, family responsibilities, and work while managing a child’s developmental dysplasia of the hip (DDH) can feel overwhelming. Here are some simple tips to help:

1. **Ask for help**: You don’t have to do everything alone. Ask family, friends, or neighbors to help with tasks like cooking, cleaning, or watching your child. For example, if your child needs regular doctor visits, ask someone to help with school pickups or errands.
2. **Take care of yourself**: Your mental health matters. Take short breaks to relax, even if it’s just 10 minutes to drink tea or take a walk. If you feel stressed, talk to someone you trust or join a support group for parents of children with DDH.
3. **Plan ahead**: Use a calendar or app to organize doctor appointments, work deadlines, and family activities. This helps you stay on top of things without feeling rushed.
4. **Talk to your employer**: If work feels too much, let your employer know about your situation. They might offer flexible hours or let you work from home sometimes.
5. **Focus on small wins**: Celebrate small successes, like your child wearing their brace without fuss or completing a week of physical therapy. This keeps you motivated.
6. **Involve your family**: Teach siblings or other family members about DDH so they can help and feel included. For example, they can play games with the child that don’t involve too much movement.

**How might DDH affect future quality of life? How can I plan ahead to improve health?**

DDH, if not treated early, can cause long-term problems like pain, difficulty walking, or arthritis in the hip. But with proper care, most children grow up to live normal, active lives. Here’s how you can plan ahead:

1. **Follow the treatment plan**: If your child needs a brace, harness, or surgery, make sure they use it as the doctor recommends. This helps their hip develop correctly.
2. **Encourage safe activities**: Once treated, your child can play and exercise, but avoid high-impact sports like football or gymnastics unless the doctor says it’s okay. Swimming or biking are great options because they’re gentle on the joints.
3. **Regular check-ups**: Keep up with doctor visits to make sure the hip is growing well. Early detection of any issues can prevent bigger problems later.
4. **Stay positive**: Help your child stay confident and active. If they feel different because of DDH, remind them that they’re strong and capable. For example, if they need a brace, explain it’s like wearing glasses—it helps them get better.
5. **Plan for the future**: If DDH causes long-term issues, like arthritis, talk to the doctor about ways to manage it. Staying active, eating healthy, and maintaining a good weight can help protect the hip joint.

By staying organized, asking for help, and focusing on your child’s treatment, you can manage DDH while keeping your family and work life balanced.

**Reply from DeepSeek-V3 (Third time)**

**What is Developmental Dysplasia of the Hip (DDH)?**

DDH is a condition where a baby’s hip joint doesn’t form properly. The hip joint is like a ball (the top of the thigh bone) sitting in a socket (part of the pelvis). In DDH, the ball may be loose in the socket, or it may slip out completely. This can happen in one or both hips.

If not treated, DDH can lead to problems like pain, limping, or arthritis as the child grows older. Think of it like a door hinge that doesn’t fit right—it might work for a while, but over time, it can wear out and cause trouble.

**Who is most likely to develop DDH? How common is it?**

DDH is more common in:

- **Girls**: They are about 4 times more likely to have it than boys.
- **Firstborn babies**: They have less room in the womb, which can affect hip development.
- **Babies born breech (feet first)**: This position can put extra pressure on the hips.
- **Family history**: If a parent or sibling had DDH, the risk is higher.

DDH affects about 1 to 3 out of every 1,000 babies. It’s not super common, but it’s important to catch it early.

**Will DDH have a long-term impact on my quality of life? What aspects should I pay special attention to?**

If DDH is found and treated early, most children grow up with normal hips and no long-term problems. Treatment might include a special harness, brace, or in rare cases, surgery.

However, if DDH is not treated, it can lead to:

- **Pain**: The hip may hurt during activities like walking or running.
- **Limping**: The child might walk unevenly.
- **Arthritis**: The hip joint can wear out faster, causing stiffness and pain later in life.

**What to pay attention to:**

- **Early signs**: Look for uneven leg folds, one leg that seems shorter, or difficulty moving one hip.
- **Regular check-ups**: Doctors check babies’ hips during routine visits. Don’t skip these!
- **Follow treatment plans**: If your child needs a harness or brace, use it as directed. It might seem uncomfortable at first, but it helps the hip grow correctly.

Think of DDH like a crooked picture frame. If you fix it early, it hangs straight and looks great. But if you wait too long, it might fall and break. Early action makes all the difference!

### What parts of the body does developmental dysplasia of the hip (DDH) mainly affect? What do these parts do in a healthy state?

**Parts Affected:**

DDH mainly affects the hip joint. The hip joint is where the thigh bone (femur) connects to the pelvis. In a healthy hip, the top of the thigh bone (the "ball") fits snugly into a socket in the pelvis. This allows the leg to move smoothly in many directions, like when you walk, run, or sit.

**What These Parts Do in a Healthy State:**

- The **ball and socket** work together to let your leg move freely.

- The **ligaments** (strong bands of tissue) hold the joint in place.

- The **cartilage** (smooth tissue) cushions the joint so it doesn’t hurt when you move.

Think of the hip joint like a well-fitting puzzle piece. When it’s healthy, it moves easily and doesn’t cause pain.

---

### What symptoms or consequences may arise if these parts are damaged? Can you provide specific examples?

**Symptoms of DDH:**

1. **Uneven Legs:** One leg might look shorter than the other because the hip joint isn’t in the right place.

- *Example:* A baby might have one foot that doesn’t line up with the other when lying down.

2. **Limited Movement:** The hip might not move as freely as it should.

- *Example:* A parent might notice their baby’s legs don’t spread apart easily during diaper changes.

3. **Limping or Waddling:** In older kids, walking might look uneven or awkward.

- *Example:* A toddler might walk with a limp or sway side to side like a duck.

4. **Pain:** If DDH isn’t treated, it can cause pain in the hip, especially as the child grows.

- *Example:* A teenager might feel pain in their hip after running or playing sports.

**Consequences if Untreated:**

- The hip joint can wear out faster, leading to arthritis (joint pain and stiffness) at a young age.

- The child might need surgery later in life to fix the problem.

---

### Real-Life Example:

Imagine a baby named Mia. Her parents notice that one of her legs seems shorter, and her hips make a clicking sound when they move her legs. The doctor says Mia has DDH. If treated early with a special brace, Mia’s hip can grow normally, and she’ll be able to run and play like other kids. But if it’s not treated, Mia might have trouble walking or need surgery when she’s older.

In short, DDH affects the hip joint, and early treatment is key to avoiding long-term problems!

**What are the main causes of DDH? What risk factors may increase the chances of developing it?**

**Main Causes:**
Developmental Dysplasia of the Hip (DDH) happens when the hip joint doesn’t form properly. The hip joint is like a ball (the top of the thigh bone) sitting in a socket (part of the pelvis). In DDH, the ball doesn’t fit well in the socket, or it can slip out. This can happen before birth, during infancy, or even as a child grows.

**Risk Factors:**

1. **Family History:** If someone in the family had DDH, the baby might be more likely to have it.
2. **Breech Position:** If the baby is in a breech position (feet first) before birth, the hips can be pushed out of place.
3. **Firstborn Child:** The first baby often has less room in the womb, which can affect hip development.
4. **Girls:** Girls are more likely to have DDH than boys.
5. **Swaddling Too Tight:** If a baby’s legs are wrapped tightly straight down, it can increase the risk.

**What lifestyle habits or environmental factors increase the risk of developing DDH? How can I prevent it?**

**Lifestyle/Environmental Factors:**

1. **Swaddling Incorrectly:** Swaddling a baby with their legs straight and tight can push the hips out of place. Always swaddle with the legs loose and able to move.
2. **Carrying the Baby Incorrectly:** Using baby carriers that don’t support the hips properly can increase the risk. Make sure the baby’s legs are spread apart and supported.

**Prevention Tips:**

1. **Proper Swaddling:** When swaddling, leave room for the baby’s legs to bend and move.
2. **Hip-Friendly Carriers:** Use carriers that support the baby’s hips in a natural, spread position.
3. **Regular Checkups:** Take your baby to the doctor for regular checkups. The doctor will check the hips to make sure they’re developing correctly.

**If I have a family history, is my risk of developing DDH higher? What preventive measures should I take?**

**Family History and Risk:**
Yes, if someone in your family had DDH, your baby might have a higher chance of having it too. This doesn’t mean it will definitely happen, but it’s something to watch for.

**Preventive Measures:**

1. **Tell Your Doctor:** Let your doctor know about your family history. They might check your baby’s hips more closely.
2. **Hip Exams:** Make sure your baby gets regular hip exams during checkups. The doctor will move the baby’s legs to see if the hips are stable.
3. **Ultrasound or X-ray:** If the doctor thinks there might be a problem, they might do an ultrasound or X-ray to look at the hips more closely.
4. **Early Treatment:** If DDH is found early, it’s easier to treat. Treatment might include a special brace or harness to help the hips develop correctly.

**Real-Life Example:**

Imagine a baby named Emma. Her mom had DDH as a baby, so the doctor checks Emma’s hips carefully at every visit. When Emma is 2 months old, the doctor notices her hips are a little loose. They do an ultrasound and find mild DDH. Emma wears a special harness for a few months, and her hips grow perfectly. Because her mom told the doctor about the family history, Emma got help early and avoided bigger problems later.

**Developmental Dysplasia of the Hip (DDH)** is a condition where a baby’s hip joint doesn’t form properly. The hip joint is like a ball (the top of the thigh bone) that fits into a socket (part of the pelvis). In DDH, the ball is loose or slips out of the socket. Here’s what you need to know:

**What are the main symptoms of DDH?**

1. **Uneven leg lengths**: One leg may look shorter than the other.
2. **Uneven skin folds**: The folds on the thighs or buttocks may look different on each side.
3. **Limited movement**: The hip may not move as freely as it should. For example, one leg might not spread out as far as the other.
4. **Clicking or popping sound**: You might hear a clicking noise when moving the hip.
5. **Limping or waddling walk**: When the child starts walking, they might limp or walk awkwardly.

*Real-life example*: Imagine a baby whose legs don’t seem to move the same way during diaper changes. Or a toddler who walks with a funny wobble. These could be signs of DDH.

**What signs indicate the disease is worsening?**

1. **Pain**: If the child starts crying or seems uncomfortable when their hip is moved.
2. **Difficulty walking**: If the limp or waddle gets worse as the child grows.
3. **Stiffness**: If the hip becomes harder to move or feels stuck.
4. **Visible dislocation**: In severe cases, the hip might look out of place.

*Real-life example*: If a 2-year-old who used to walk normally suddenly starts limping or refuses to walk, this could mean DDH is getting worse.

**Should I seek immediate medical attention?**

- **Yes, if**:
  1. You notice any of the symptoms above, especially in a newborn or young child.
  2. The child seems to be in pain or can’t move their hip properly.
  3. The walking pattern changes suddenly or gets worse.

DDH is easier to treat when caught early. If left untreated, it can lead to long-term problems like arthritis or a permanent limp.

*Real-life example*: If you notice something unusual during diaper changes or when your child starts walking, don’t wait. Talk to a doctor right away. It’s better to check and be sure than to miss something important.

In short, DDH is a treatable condition, but early detection is key. If you see any signs, don’t hesitate to see a doctor. They can help fix the problem before it causes bigger issues.

**What methods do doctors usually use to confirm the diagnosis of Developmental Dysplasia of the Hip (DDH)? What is the purpose of each test?**

1. **Physical Examination**:
   - Doctors check the baby’s hips during routine check-ups. They move the legs to see if the hips are stable or if there’s a “clunk” sound, which could mean the hip is dislocated.
   - **Purpose**: To find signs of looseness or instability in the hip joint early on.
2. **Ultrasound**:
   - This is a painless test that uses sound waves to create pictures of the baby’s hips. It’s often used for babies under 6 months old because their bones are still soft.
   - **Purpose**: To see if the hip joint is properly formed or if the ball of the hip is sitting correctly in the socket.
3. **X-ray**:
   - For older babies (usually over 6 months), doctors use X-rays to look at the bones of the hip joint.
   - **Purpose**: To check how the bones are growing and if the hip is in the right position.

**What indicators are most important in the test results? How do these results reflect the severity of the disease?**

1. **Hip Stability**:
   - If the hip moves too much or is dislocated, it’s a sign of DDH.
   - **Severity**: A loose hip is milder, but a fully dislocated hip is more serious.
2. **Shape of the Hip Joint**:
   - On an ultrasound or X-ray, doctors look at how the ball of the hip fits into the socket. If the ball is not centered or the socket is too shallow, it’s a problem.
   - **Severity**: A shallow socket or a ball that’s out of place means the hip isn’t developing properly.
3. **Age of the Child**:
   - Younger babies (under 6 months) have softer bones, so treatment is easier. Older children may need more complex care.
   - **Severity**: The older the child, the harder it is to fix the hip without surgery.

**After confirming the diagnosis of DDH, do I need to schedule regular follow-ups? How often should I have them and what should be checked?**

1. **Yes, regular follow-ups are very important**.
   - DDH treatment takes time, and doctors need to make sure the hip is growing correctly.
2. **How often**:
   - For babies in a **Pavlik harness** (a soft brace that holds the hips in place), check-ups are usually every 1-2 weeks at first.
   - After the hip is stable, visits may be every few months.
   - For older children who had surgery, follow-ups depend on their recovery but are usually every 3-6 months.
3. **What’s checked**:
   - **Hip movement**: Doctors make sure the hip is stable and moving well.
   - **Imaging tests**: Ultrasounds or X-rays are used to see if the hip joint is growing normally.
   - **Growth and development**: Doctors check if the child is meeting milestones like crawling or walking.

**Real-life example:**

Imagine a baby named Mia. At her 2-month check-up, the doctor notices her left hip feels loose. An ultrasound shows the hip socket is shallow. Mia wears a Pavlik harness for a few months. Her parents take her for regular check-ups. Over time, the hip socket deepens, and Mia’s hip grows normally. By her first birthday, she’s walking just like any other toddler! Regular follow-ups helped catch and fix the problem early.

**What symptoms indicate that I need to seek immediate medical attention?**

If you notice any of the following signs in your child, it’s important to see a doctor right away:

1. **Uneven leg lengths**: One leg looks shorter than the other when your child is lying down.
2. **Uneven skin folds**: The folds on the thighs or buttocks don’t look the same on both sides.
3. **Limited movement**: Your child has trouble moving one leg as much as the other. For example, one leg might not spread out as far during a diaper change.
4. **Hip clicking or popping**: You hear or feel a clicking or popping sound when moving your child’s hip.
5. **Limping or unusual walking**: Once your child starts walking, they might limp or walk on their toes.

**Real-life example**: Imagine you’re changing your baby’s diaper and notice one leg doesn’t move as easily as the other. Or, when your toddler starts walking, they seem to wobble more on one side. These could be signs of DDH.

**How should I handle and self-monitor during an emergency situation?**

DDH isn’t usually an emergency that requires rushing to the hospital, but if you notice any of the symptoms above, here’s what to do:

1. **Stay calm**: DDH is treatable, especially when caught early.
2. **Avoid forcing movement**: Don’t try to push or pull your child’s leg if it seems stiff or painful.
3. **Monitor symptoms**: Keep an eye on the signs you noticed, like uneven leg lengths or limited movement. Write them down so you can tell the doctor.
4. **Schedule a doctor’s visit**: Call your pediatrician and explain what you’ve noticed. They’ll likely want to see your child soon.
5. **Follow up**: If the doctor confirms DDH, they might recommend treatments like a special brace or physical therapy. Stick to the plan to help your child’s hip develop properly.

**Real-life example**: If you notice your baby’s legs look uneven, don’t panic. Just make a note of it and call your doctor. They might ask you to come in for a check-up. If your child needs a brace, think of it like a cast for a broken bone—it helps their hip grow the right way.

By staying observant and acting quickly, you can help your child get the care they need for a healthy hip!

**What treatments will I receive during hospitalization? What is the purpose of each treatment?**

1. **Pavlik Harness**:
   - **What it is**: A soft brace that keeps your baby’s hips in the right position.
   - **Purpose**: It helps the hip joint grow correctly by holding the ball of the hip in the socket.
   - **Example**: Think of it like a seatbelt for the hips, gently guiding them into place.
2. **Casting (Spica Cast)**:
   - **What it is**: A hard cast that covers the baby’s hips and legs to keep them stable.
   - **Purpose**: It holds the hip in place after the doctor adjusts it (if needed).
   - **Example**: Like a plaster mold that keeps the hip from moving while it heals.
3. **Surgery (if needed)**:
   - **What it is**: A doctor may need to fix the hip joint if it’s very out of place.
   - **Purpose**: To move the hip back into the correct position so it can grow normally.
   - **Example**: Like fixing a puzzle piece that’s in the wrong spot.
4. **Medications**:
   - **What it is**: Pain relief or antibiotics (if there’s an infection).
   - **Purpose**: To keep your child comfortable and prevent infections.
   - **Example**: Like giving medicine for a headache or a cut.

**How can I prevent infections or other complications during hospitalization? What can family members do to assist with recovery?**

1. **Prevent Infections**:
   - Wash hands often with soap and water.
   - Keep the hospital room clean.
   - Follow the doctor’s instructions for caring for braces, casts, or wounds.
   - **Example**: Treat the hospital room like your kitchen—keep it tidy and clean to avoid germs.
2. **Family Help**:
   - Learn how to care for the brace or cast (e.g., keeping it dry, checking for redness).
   - Comfort your child during treatments or check-ups.
   - Help with exercises or positioning as advised by the doctor.
   - **Example**: Be like a coach, cheering your child on and helping them stay on track.

**How can I reinforce the effects of treatment after discharge? What rehabilitation programs are recommended?**

1. **Follow-Up Appointments**:
   - Go to all check-ups to make sure the hip is healing well.
   - **Example**: Like taking your car for regular tune-ups to keep it running smoothly.
2. **Physical Therapy**:
   - Gentle exercises to strengthen the hip and improve movement.
   - **Example**: Like stretching and playing to help the hip get stronger.
3. **Home Care**:
   - Keep using braces or casts as directed.
   - Avoid activities that could hurt the hip (e.g., rough play).
   - **Example**: Treat the hip like a delicate flower—handle it with care.
4. **Healthy Habits**:
   - Feed your child a balanced diet to help bones grow strong.
   - Encourage light play as recommended by the doctor.
   - **Example**: Think of food and play as fuel for healing and growth.

By following these steps, you can help your child’s hip heal properly and grow strong!

**Developmental Dysplasia of the Hip (DDH) in Children**

**What are the common medications used to treat DDH? How should I take them correctly?**

DDH is not typically treated with medications. Instead, it is usually managed with physical methods like braces, harnesses, or sometimes surgery. The goal is to help the hip joint develop correctly. For example, a Pavlik harness is a soft brace that holds the baby’s hips in the right position. It’s like a special seatbelt for the hips! If your child needs a harness, the doctor will show you how to put it on and take it off correctly. Always follow the doctor’s instructions.

**What should I do if I forget to use the harness or use it at the wrong time? Will it affect the treatment?**

If you forget to put the harness on your baby or take it off at the wrong time, don’t panic. Just put it back on as soon as you remember. However, try to stick to the schedule the doctor gave you. Using the harness correctly is important because it helps the hip grow properly. If you miss using it too often, it might slow down the treatment. If you’re unsure, call your doctor for advice.

**What foods should I avoid while using the harness or other treatments for DDH? Are there common side effects?**

Since DDH treatment doesn’t involve medications, there are no specific foods to avoid. However, if your baby is wearing a harness, make sure they are comfortable and can move freely. Sometimes, the harness might cause mild redness or irritation on the skin. If this happens, you can put a soft cloth under the harness to protect the skin. If the redness gets worse or your baby seems very uncomfortable, contact your doctor.

In summary, DDH is treated with physical methods like harnesses, not medications. Follow the doctor’s instructions carefully, and if you have any concerns, don’t hesitate to ask for help.

**What types of pain may be caused by DDH or its treatment? Where is the pain usually felt?**

**Developmental Dysplasia of the Hip (DDH)** is a condition where a baby’s hip joint doesn’t form properly. The hip joint is a ball-and-socket joint, but in DDH, the ball (top of the thigh bone) may not fit well into the socket (part of the pelvis). This can cause pain or discomfort in the hip area, groin, or even the thigh. If left untreated, it can lead to limping, difficulty walking, or arthritis later in life.

**Pain from DDH itself**:

- Babies may not feel pain, but as they grow, they might feel discomfort in the hip, groin, or thigh, especially when walking or running.
- Older children or teens with untreated DDH might feel pain in the hip or lower back due to uneven wear on the joint.

**Pain from treatment**:

- Treatments like braces, casts, or surgery can cause temporary discomfort. For example, a baby wearing a brace might feel some pressure or soreness around the hips.
- After surgery, there might be pain around the incision site or stiffness in the hip.

**What methods can help relieve pain? What are the recommended treatments?**

**Non-pharmacological (non-medicine) methods**:

1. **Braces or harnesses**: These help hold the hip in the right position. They might feel strange at first, but they usually don’t cause much pain.
2. **Physical therapy**: Gentle exercises can help strengthen the muscles around the hip and improve movement.
3. **Heat or cold packs**: For older kids, a warm pack can relax muscles, and a cold pack can reduce swelling or pain.
4. **Rest**: Avoiding activities that strain the hip can help.

**Pharmacological (medicine) methods**:

1. **Pain relievers**: Over-the-counter medicines like acetaminophen (Tylenol) or ibuprofen (Advil) can help with pain. Always check with a doctor for the right dose for a child.
2. **Prescription painkillers**: After surgery, stronger medicines might be needed for a short time.

**What steps should I take if the pain is more than expected or worsens? Do I need to contact my doctor?**

1. **Monitor the pain**: If your child seems more uncomfortable than usual, or if the pain doesn’t go away with rest or medicine, take note.
2. **Check for other signs**: Look for redness, swelling, or fever, which could mean an infection, especially after surgery.
3. **Contact your doctor**: If the pain is severe, lasts a long time, or gets worse, call your doctor. They might need to adjust the treatment plan. For example, they might change the brace, suggest different exercises, or check for complications.
4. **Follow up**: Regular check-ups are important to make sure the hip is healing properly.

**Real-life example:**

Imagine a baby named Mia who has DDH. She wears a special harness to keep her hips in place. At first, she fusses a little because it’s new, but she gets used to it. Her parents give her gentle massages and use warm towels to soothe her. If Mia cries a lot or seems in pain, her parents call the doctor to make sure everything is okay.

For an older child, like 8-year-old Jake, who had surgery for DDH, he might feel some pain after the operation. His parents give him medicine as prescribed and help him do simple exercises to keep his hip moving. If Jake’s pain gets worse or he can’t walk, his parents take him back to the doctor to check for any problems.

By paying attention to pain and working with the doctor, most kids with DDH can recover well and live active lives!

**What post-surgery care precautions should I take?**

After surgery for developmental dysplasia of the hip (DDH), it’s important to keep the hip safe and help it heal. Here are some key precautions:

- **Keep the wound clean and dry**: Follow your doctor’s instructions for cleaning the surgical area to avoid infection.
- **Use assistive devices**: Your child may need a brace, cast, or crutches to protect the hip. Make sure they use them as directed.
- **Avoid putting weight on the hip**: Depending on the surgery, your child may need to avoid standing or walking for a while.
- **Watch for signs of infection**: Look for redness, swelling, or fever, and tell the doctor if you notice these.

**How long after surgery can I return to normal daily activities?**

This depends on the type of surgery and how well your child heals. For example:

- Light activities like sitting or moving gently may start within a few weeks.
- Walking and running may take several months.
- Your doctor will give a timeline based on your child’s progress.

**How soon after surgery can I begin resuming activities?**

Your child can start gentle movements as soon as the doctor says it’s safe. For example:

- Simple stretches or exercises may begin a few weeks after surgery.
- More active play, like running or jumping, will take longer—sometimes 3 to 6 months or more.

**What activities are recommended during the recovery period?**

During recovery, focus on activities that help the hip heal without straining it:

- **Gentle exercises**: These may include stretching or moving the hip as guided by a physical therapist.
- **Non-weight-bearing play**: Activities like drawing, reading, or playing with toys while sitting are good.
- **Swimming (later in recovery)**: Once the doctor approves, swimming can be a great way to strengthen the hip without putting too much pressure on it.

**How do I schedule my exercise during the postoperative recovery period?**

Your doctor or physical therapist will create a plan. Here’s an example:

- **Weeks 1-2**: Rest and gentle movements.
- **Weeks 3-6**: Start light exercises to improve flexibility.
- **Months 2-3**: Add strengthening exercises.
- **Months 4-6**: Gradually return to normal activities.

Always follow the plan and check with the doctor before making changes.

**What are the stages during postoperative recovery? What precautions should I take in each stage?**

1. **Early Stage (First few weeks)**:
   - Focus on healing and pain management.
   - Avoid putting weight on the hip.
   - Keep the surgical area clean.
2. **Middle Stage (Weeks 3-6)**:
   - Start gentle exercises to improve movement.
   - Continue using assistive devices like braces or crutches.
3. **Late Stage (Months 2-6)**:
   - Gradually increase activity levels.
   - Strengthen the hip with guided exercises.
   - Avoid high-impact activities like jumping or running until the doctor says it’s okay.

**Do I need a specialized rehabilitation plan? How does it help with recovery?**

Yes, a rehabilitation plan is very helpful. It’s like a step-by-step guide to help your child’s hip heal and get stronger. A physical therapist will create a plan that includes:

- Exercises to improve movement and strength.
- Tips to avoid re-injury.
- A timeline for returning to normal activities.

This plan ensures your child recovers safely and fully.

**How can I prevent recurrence after surgery?**

To prevent DDH from coming back:

- Follow the doctor’s instructions for braces or exercises.
- Avoid activities that strain the hip too soon.
- Attend all follow-up appointments to monitor progress.
- Encourage healthy habits, like maintaining a healthy weight, to reduce stress on the hip.

**What complications can occur after surgery? What steps should I take?**

Some possible complications include:

- **Infection**: Keep the wound clean and watch for redness, swelling, or fever. Tell the doctor right away if you see these signs.
- **Stiffness**: Gentle exercises can help, but don’t push too hard.
- **Re-dislocation**: Follow the doctor’s advice on activity limits and braces.

If you notice anything unusual, like severe pain or trouble moving the hip, contact the doctor immediately. Regular check-ups will help catch and address problems early.

**Developmental Dysplasia of the Hip (DDH) in Children: Diet, Exercise, and Sleep Tips**

**1. What foods are helpful in recovery from DDH or during recovery from surgery? What foods may aggravate the condition?**

- **Helpful Foods**:
  - **Protein-rich foods**: Like eggs, chicken, fish, beans, and yogurt. These help repair muscles and tissues after surgery.
  - **Calcium and Vitamin D**: Milk, cheese, leafy greens (like spinach), and fortified cereals. These strengthen bones.
  - **Fruits and vegetables**: Oranges, strawberries, broccoli, and carrots. They have vitamins and antioxidants that help healing.
  - **Whole grains**: Brown rice, oatmeal, and whole-grain bread. They give energy and help digestion.
- **Foods to Avoid**:
  - **Sugary snacks and drinks**: Candy, soda, and cookies can cause inflammation and slow healing.
  - **Processed foods**: Chips, fast food, and frozen meals. They lack nutrients and can make recovery harder.

**Real-life example**: Think of your body like a car. After surgery, it needs the right fuel (healthy food) to run smoothly and fix itself. Junk food is like bad fuel—it won’t help the car run well.

**2. How should I adjust my diet to prevent constipation or other common problems?**

- **Drink plenty of water**: Staying hydrated helps digestion.
- **Eat fiber-rich foods**: Fruits (like apples and pears), vegetables (like carrots and peas), and whole grains (like oatmeal). Fiber keeps things moving in your gut.
- **Avoid too much cheese or processed foods**: These can make constipation worse.

**Real-life example**: Imagine a garden hose. Water (hydration) and fiber are like turning on the tap—they keep everything flowing. Without them, the hose gets clogged.

**3. What exercises can I do during the recovery phase? How often should I do them?**

- **Gentle movements**: If your child is in a cast or brace, focus on small movements like wiggling toes or gentle leg stretches (if allowed by the doctor).
- **Physical therapy exercises**: After surgery, a therapist may guide your child through safe exercises to strengthen the hip.
- **Frequency**: Start slow, like 5–10 minutes a day, and increase as the doctor advises.

**Real-life example**: Think of recovery like learning to ride a bike. You start with training wheels (gentle exercises) and slowly build up to riding on your own.

**4. What precautions should I take when exercising, especially during postoperative recovery?**

- **Follow the doctor’s advice**: Don’t push too hard or do exercises that aren’t approved.
- **Avoid high-impact activities**: Running, jumping, or rough play can hurt the healing hip.
- **Watch for pain**: If an exercise hurts, stop and tell the doctor.
- **Use braces or supports if needed**: These protect the hip during recovery.

**Real-life example**: Imagine healing a broken vase. You handle it carefully and don’t move it too much until the glue (surgery) dries completely.

**5. Does DDH affect the quality of my sleep? What are some ways to improve sleep and help the body recover?**

- **Sleep challenges**: Pain, discomfort, or wearing a brace/cast can make it harder to sleep.
- **Tips for better sleep**:
  - **Comfortable position**: Use pillows to support the body and keep the hip in a good position.
  - **Relax before bed**: Read a book or listen to calming music.
  - **Keep a routine**: Go to bed and wake up at the same time every day.
  - **Ask the doctor about pain relief**: If pain keeps your child awake, the doctor may suggest safe options.

**Real-life example**: Think of sleep like charging a phone. If the phone doesn’t charge fully (poor sleep), it won’t work well the next day. Good sleep helps the body “recharge” and heal.

By focusing on healthy foods, gentle exercises, and good sleep, your child can recover better from DDH or surgery. Always check with the doctor for specific advice!

**Psychological Problems Associated with DDH and Postoperative Recovery**

**1. Anxiety:**

- **Why it happens:** Children with DDH might feel anxious because of hospital visits, strange medical equipment, or fear of pain after surgery. Parents might also feel anxious about their child’s health and recovery.
- **Example:** Imagine a child who has to wear a special brace or cast. They might feel scared because they can’t move like before. Parents might worry if the treatment is working.

**2. Depression:**

- **Why it happens:** Long-term treatment or limited movement can make a child feel sad or frustrated. They might miss playing with friends or feel different from others.
- **Example:** A child who loves soccer but can’t play for months might feel left out and sad.

**3. Frustration or Anger:**

- **Why it happens:** Children might get upset because they can’t do things they used to enjoy. Parents might feel stressed and overwhelmed, which can lead to frustration.
- **Example:** A child might throw a tantrum because they can’t run around, and a parent might feel helpless trying to comfort them.

**How to Deal with These Feelings Positively**

**1. For Anxiety:**

- **Stay calm and explain things:** Use simple words to explain what’s happening. For example, “This brace will help your hip get stronger.”
- **Distraction:** Use toys, games, or stories to take their mind off worries.
- **Routine:** Keep a daily routine to make things feel normal.

**2. For Depression:**

- **Encourage small wins:** Celebrate little achievements, like sitting up or playing a quiet game.
- **Stay connected:** Help your child talk to friends or family, even if it’s through video calls.
- **Fun activities:** Find new hobbies they can do while resting, like drawing or reading.

**3. For Frustration or Anger:**

- **Listen and validate:** Let your child express their feelings. Say, “I know it’s hard, but you’re doing great.”
- **Teach coping skills:** Show them how to take deep breaths or count to ten when upset.
- **Take breaks:** Parents should also take time for themselves to relax and recharge.

**Signs You Need Psychological Support**

**For Children:**

- Crying a lot or being very clingy.
- Not wanting to eat or sleep well.
- Losing interest in things they used to enjoy.
- Saying things like “I’m no good” or “I hate this.”

**For Parents:**

- Feeling overwhelmed or crying often.
- Having trouble sleeping or eating.
- Feeling angry or hopeless.
- Struggling to take care of your child or yourself.

**How to Find Professional Help or Support Resources**

**1. Talk to Your Doctor:**

- Your child’s doctor can recommend a psychologist or counselor who works with kids and families.

**2. Look for Support Groups:**

- Join groups for parents of children with DDH. They can share tips and offer emotional support.

**3. School Resources:**

- If your child is in school, ask if they have a counselor who can help.

**4. Online Resources:**

- Websites like the American Psychological Association (APA) or local mental health organizations can guide you to help.

**5. Hotlines:**

- If you’re feeling really stressed, call a mental health hotline. They can listen and point you to resources.

**Real-Life Example:**

Imagine a family with a 5-year-old named Mia who has DDH. Mia feels sad because she can’t dance like her friends. Her mom feels anxious about the surgery. They talk to their doctor, who suggests a child psychologist. The psychologist helps Mia express her feelings through drawing and teaches her mom ways to stay calm. They also join a support group and meet other families going through the same thing. Over time, Mia feels happier, and her mom feels more confident.

Remember, it’s okay to ask for help. Taking care of your mental health is just as important as treating the physical problem.

**1. Are there any recommended health apps or devices that can help manage DDH or postoperative recovery?**

For children with **developmental dysplasia of the hip (DDH)**, there aren’t many specific apps or devices just for this condition. But some general health tools can help parents manage their child’s care and recovery after surgery. Here are a few examples:

- **Baby Tracker Apps**: Apps like *Baby Tracker* or *Huckleberry* can help parents keep track of their child’s daily activities, like feeding, sleeping, and diaper changes. After DDH surgery, these apps can help monitor if the child is comfortable and recovering well.
- **Physical Therapy Apps**: Apps like *PhysiApp* or *PT Timer* can guide parents through simple exercises or stretches recommended by a physical therapist. These exercises are often needed after DDH treatment to help the hip heal and strengthen.
- **Wearable Devices**: Devices like *Fitbit Ace* (for kids) can track movement and activity levels. After surgery, parents can use this to make sure their child is moving enough but not overdoing it.
- **Pain Management Apps**: Apps like *PainScale* can help parents track their child’s pain levels and share this information with the doctor. This is especially helpful after surgery to ensure the child isn’t in too much discomfort.

**2. How can I use health tracking tools to monitor my condition or recovery progress?**

For DDH, tracking tools can help parents keep an eye on their child’s recovery and make sure everything is going as planned. Here’s how you can use them:

- **Track Movement and Activity**: After DDH surgery, doctors often recommend gentle movement to help the hip heal. A wearable device or app can help you see if your child is moving enough but not too much. For example, if the doctor says your child should walk a little each day, you can use a step tracker to monitor this.
- **Monitor Pain Levels**: Use a pain tracking app or even a simple notebook to write down how much pain your child is in each day. This helps you notice if the pain is getting better or worse and share this with the doctor.
- **Record Physical Therapy Progress**: If your child needs physical therapy, use an app to track their exercises and progress. For example, if the therapist says your child should do 10 leg lifts a day, you can log this in the app and see improvements over time.
- **Keep a Recovery Journal**: Write down or use an app to record things like how your child is sleeping, eating, and feeling. This helps you spot any problems early, like if your child is having trouble sleeping because of discomfort.

**Real-Life Example:**

Imagine your 1-year-old had surgery for DDH. You use a baby tracker app to log their sleep and feeding patterns. You notice they’re sleeping more than usual, which might mean they’re still in pain. You also use a step tracker to make sure they’re moving a little each day, as the doctor recommended. At the next check-up, you show the doctor your notes and the tracker data. This helps the doctor adjust the treatment plan if needed.

By using these tools, you can feel more in control of your child’s recovery and make sure they’re on the right track!

**1. What are some ways to access reliable information about Developmental Dysplasia of the Hip (DDH) or post-operative care?**

- **Talk to your doctor or pediatrician**: They are the best source of accurate information. Ask them to explain DDH and the treatment plan in simple terms. For example, they might say, “Your child’s hip joint is not forming properly, and we need to help it grow correctly.”
- **Trusted medical websites**: Look for websites like the **American Academy of Pediatrics (AAP)**, **Mayo Clinic**, or **CDC**. These sites are reliable and explain things clearly. For example, they might say, “DDH means the hip socket is too shallow, so the ball of the joint doesn’t fit well.”
- **Children’s hospitals**: Many children’s hospitals have online resources. For example, **Boston Children’s Hospital** or **Children’s Hospital of Philadelphia** have easy-to-read guides about DDH and what to expect after surgery.
- **Books or pamphlets**: Your doctor might give you brochures or recommend books written for parents. These often use pictures and simple language to explain things.
- **Avoid unreliable sources**: Don’t rely on random blogs or social media posts. Stick to trusted medical professionals or organizations.

**2. Are there support groups or community resources that can help me or my family cope better?**

- **Local support groups**: Ask your doctor if there are local groups for parents of children with DDH. For example, you might meet other parents whose children are also wearing a hip brace or recovering from surgery. Sharing experiences can make you feel less alone.
- **Online communities**: Websites like **Facebook** or **Reddit** have groups for parents of children with DDH. For example, you might find a group called “DDH Parents Support Group” where people share tips and encouragement.
- **Nonprofit organizations**: Groups like **Steps Charity Worldwide** or **International Hip Dysplasia Institute (IHDI)** offer resources, stories from other families, and even financial help for treatment.
- **Physical therapists or nurses**: They can teach you how to care for your child after surgery. For example, they might show you how to change diapers while your child is in a cast or how to do gentle exercises to help their hip heal.
- **Family and friends**: Don’t be afraid to ask for help. For example, if your child is in a cast, you might need help carrying them or doing household chores.

**Real-Life Example:**

Imagine your child, Mia, is diagnosed with DDH. The doctor says she needs a special brace to help her hip grow correctly. You feel worried and confused. Here’s how you can cope:

1. **Ask the doctor questions**: “How long will Mia wear the brace? Will it hurt her?”
2. **Visit a trusted website**: Look up DDH on the Mayo Clinic website to learn more.
3. **Join a Facebook group**: Connect with other parents who have been through the same thing.
4. **Reach out to a nonprofit**: Contact the International Hip Dysplasia Institute for tips and support.

By using these resources, you’ll feel more confident and less alone in helping Mia through her treatment.

**What is Developmental Dysplasia of the Hip (DDH)?**

Developmental Dysplasia of the Hip (DDH) is a condition where a baby’s hip joint doesn’t form properly. The hip joint is like a ball and socket. In DDH, the ball (top of the thigh bone) doesn’t fit well into the socket (part of the pelvis). This can cause the hip to be loose, dislocated, or not work properly. If not treated early, it can lead to pain, walking problems, or arthritis later in life.

**What is the usual cost of treatment for DDH?**

The cost of treating DDH depends on how severe it is and the type of treatment needed. Here’s a rough idea:

1. **Mild Cases**: If the hip is just loose, a special harness (like the Pavlik harness) might be used. This costs around **100−100−300**. Regular check-ups with the doctor are needed, which can add to the cost.
2. **Moderate to Severe Cases**: If the hip is dislocated, surgery might be needed. Surgery costs can range from **5,000to5,000*to*20,000** or more, depending on the hospital and the complexity of the surgery. After surgery, your child might need a cast or physical therapy, which adds to the cost.
3. **Long-Term Care**: Some children need follow-up care, like X-rays or physical therapy, for years. This can add hundreds or thousands of dollars over time.

**Are there insurance or subsidy policies to reduce the financial burden?**

Yes, there are ways to reduce the cost:

1. **Health Insurance**: Most health insurance plans cover DDH treatment, including harnesses, surgery, and follow-up care. Check with your insurance provider to see what’s covered and if there are any out-of-pocket costs.
2. **Government Programs**: In many countries, government programs help cover medical costs for children. For example:
   - In the **U.S.**, Medicaid or CHIP (Children’s Health Insurance Program) can help low-income families.
   - In the **U.K.**, the NHS (National Health Service) covers most treatments for free.
   - In **Canada**, public healthcare covers surgery and hospital stays, but some costs (like braces) might need private insurance.
3. **Hospital Financial Aid**: Some hospitals offer financial assistance or payment plans for families who can’t afford treatment. Ask the hospital’s billing department about options.

**What social assistance can I apply for if the disease or surgery causes financial hardship?**

If DDH treatment causes financial problems, here are some ways to get help:

1. **Charities and Nonprofits**: Some organizations help families with medical costs. For example:
   - **Shriners Hospitals for Children** (U.S.) provides free or low-cost care for children with orthopedic conditions like DDH.
   - **MiracleFeet** (global) helps children in low-income countries get treatment for clubfoot and similar conditions.
2. **Disability Benefits**: If DDH causes long-term disability, your child might qualify for disability benefits. In the U.S., this is through **SSI (Supplemental Security Income)**. In other countries, similar programs exist.
3. **Local Community Support**: Some communities have funds or programs to help families in need. Check with local churches, community centers, or social services.
4. **Crowdfunding**: Websites like **GoFundMe** can help raise money for medical expenses. Many families use these platforms to share their story and ask for donations.

**Real-Life Example**

Imagine a family with a 6-month-old baby diagnosed with DDH. The doctor says the baby needs a Pavlik harness for 3 months. The harness costs 200,and they need 4 doctor visits,each costing 200,*and they need* 4 *doctor visits*,*eachcosting* 50. Their insurance covers 80% of the costs, so they pay $120 out of pocket.

If the baby needed surgery, the cost might be 15,000. Within surance covering 8015,000. *With insurance covering* 803,000. If they can’t afford this, they could apply for Medicaid or ask the hospital for a payment plan.

**Summary**

- DDH treatment costs vary: 100−100−300 for a harness, 5,000−5,000−20,000+ for surgery.
- Insurance, government programs, and hospital aid can reduce costs.
- Social assistance includes charities, disability benefits, and crowdfunding.
- Always ask your doctor or hospital about financial help options.

**How can I balance mental health, family responsibilities, and work in disease management? What are some practical suggestions?**

Managing a child’s developmental dysplasia of the hip (DDH) can feel overwhelming. But with some planning, you can balance your mental health, family life, and work. Here are some simple tips:

1. **Ask for help**: You don’t have to do everything alone. Ask family, friends, or neighbors to help with chores or babysitting. For example, if your child needs regular doctor visits, someone can help with school pickups.
2. **Take care of yourself**: Your mental health matters. Take short breaks, even if it’s just 10 minutes to relax. Do something you enjoy, like reading or walking. A calm mind helps you care for your child better.
3. **Plan ahead**: Use a calendar to keep track of doctor appointments, work deadlines, and family activities. This helps you stay organized and reduces stress.
4. **Talk to your employer**: Let your boss know about your situation. They might offer flexible hours or let you work from home on busy days.
5. **Join a support group**: Talking to other parents dealing with DDH can help. They can share tips and make you feel less alone.
6. **Celebrate small wins**: Managing DDH is a long process. Celebrate small successes, like your child wearing a brace without fuss or improving in physical therapy.

**How might DDH affect future quality of life? How can I plan ahead to improve health?**

DDH can affect a child’s future if not treated early. For example, untreated DDH can lead to pain, walking problems, or arthritis in the hip later in life. But with proper care, most children grow up to live active, healthy lives. Here’s how you can plan ahead:

1. **Follow the treatment plan**: If your child needs a brace, cast, or surgery, stick to the doctor’s advice. For example, wearing a Pavlik harness (a special brace) can help the hip grow correctly.
2. **Encourage physical activity**: Once the hip is better, let your child play and move. Activities like swimming or biking are great for strengthening muscles without hurting the hip.
3. **Regular check-ups**: Even after treatment, take your child for regular doctor visits. This helps catch any problems early.
4. **Healthy habits**: Teach your child to eat well and stay active. A healthy body supports strong bones and muscles.
5. **Stay positive**: DDH can be tough, but most kids do well with treatment. Focus on the progress your child is making. For example, if they start walking without pain, that’s a big win!
6. **Plan for the future**: If your child needs long-term care, talk to your doctor about what to expect. This helps you prepare emotionally and financially.

By staying proactive and positive, you can help your child live a happy, healthy life despite DDH.

**Reply from Gemini 2.0 Flash (First time)**

Absolutely! Here's an explanation of developmental dysplasia of the hip (DDH) in simple terms:

**What is Developmental Dysplasia of the Hip (DDH)?**

Imagine your hip as a ball-and-socket joint. The top of your thigh bone (the ball) fits into a curved space in your hip bone (the socket). In DDH, the socket is too shallow, so the ball doesn't fit snugly. It might be loose or even pop out of place.

**Who is Most Likely to Develop DDH?**

DDH is more common in:

- **Girls:** Girls are about eight times more likely to have DDH than boys.
- **Firstborn babies:** Doctors aren't sure why, but firstborns have a higher risk.
- **Babies born in the breech position:** If a baby is positioned bottom-down in the womb, their hips might not develop properly.
- **Babies with a family history of DDH:** If someone in your family has had DDH, you're more likely to have it too.

DDH affects about 1 in every 1,000 babies.

**Will DDH Have a Long-Term Impact?**

Most babies with DDH who get treatment early have no long-term problems. Treatment might involve wearing a special brace for a few months to help the hip joint develop correctly. In some cases, surgery might be needed.

If DDH isn't treated, it can lead to:

- **Limping**
- **Hip pain**
- **Arthritis**

**What to Pay Attention To**

- **Check your baby's hips:** If you notice that one leg seems to turn out more than the other, or if their legs don't move the same way, talk to your doctor.
- **Follow your doctor's advice:** If your baby is diagnosed with DDH, make sure to follow the treatment plan carefully.

**Important Note:** This information is just for general knowledge and shouldn't replace advice from a doctor. If you have any concerns about DDH, please talk to a healthcare professional.

This is for informational purposes only. For medical advice or diagnosis, consult a professional.

Absolutely! Let's talk about Developmental Dysplasia of the Hip (DDH).

**What parts of the body does DDH mainly affect?**

DDH mainly affects the hip joint. This is where your thigh bone (femur) meets your hip bone (pelvis). Think of it like a ball (the top of your thigh bone) fitting into a socket (part of your hip bone). In DDH, the socket is too shallow, so the ball doesn't fit snugly.

**What do these parts do in a healthy state?**

In a healthy hip, the ball sits nicely in the socket, allowing for a wide range of movement – walking, running, jumping, you name it! The joint is stable and strong.

**What symptoms or consequences may arise if these parts are damaged?**

With DDH, the hip joint can be loose or even dislocated. This can lead to:

- **Limping:** Imagine walking with a wobbly leg.
- **Pain:** The hip might ache, especially after activity.
- **Unequal leg length:** One leg might look shorter than the other.
- **Waddling gait:** Walking with a side-to-side motion like a duck.

**Specific examples:**

- A child with DDH might have trouble keeping up with friends on the playground.
- They might avoid sports or activities that put stress on the hip.
- As they grow, they might develop arthritis in the hip joint.
- In severe cases, surgery might be needed to fix the hip.

It's important to remember that DDH is treatable, especially when caught early. If you're worried about your child's hips, talk to your doctor.

**What are the main causes of DDH? What risk factors may increase the chances of developing it?**

DDH means a baby's hip joint doesn't form correctly. Imagine the hip joint like a ball (the top of the thigh bone) fitting into a socket (part of the hip bone). In DDH, the socket might be too shallow, so the ball doesn't sit in it properly.

We don't know exactly what causes DDH, but some things make it more likely:

- **Being a girl:** Girls are more likely to have DDH than boys.
- **Breech birth:** If a baby is born feet first, there's a higher chance of DDH.
- **Family history:** If someone in your family has DDH, you're more likely to have it too.
- **Firstborn child:** Firstborn children are more likely to have DDH.
- **Tight swaddling:** Wrapping a baby's legs tightly together can increase the risk.

**What lifestyle habits or environmental factors increase the risk of developing DDH? How can I prevent it?**

There aren't really any lifestyle habits that cause DDH. However, some things can increase the risk:

- **Tight swaddling:** Tight swaddling can keep a baby's legs from moving freely, which can affect hip development.

To help prevent DDH:

- **Allow your baby's legs to move freely:** Avoid tight swaddling that keeps their legs straight. Instead, allow them to bend at the hips and knees, like a frog's legs.
- **Use baby carriers or slings that support the baby's hips:** These can help keep the baby's hips in a good position.

**If I have a family history, is my risk of developing DDH higher? What preventive measures should I take?**

Yes, if someone in your family has DDH, your risk is higher. While you can't completely prevent DDH, here's what you can do:

- **Talk to your doctor:** Let them know about your family history. They might recommend an ultrasound of your baby's hips after birth to check for DDH.
- **Follow safe swaddling practices:** Avoid tight swaddling that keeps your baby's legs straight.
- **Encourage hip-healthy positioning:** Use baby carriers or slings that support your baby's hips in a bent position.
- **Regular checkups:** Make sure your baby has regular checkups with their doctor to monitor their hip development.

What are the main symptoms of developmental dysplasia of the hip (DDH)?

DDH is a condition in which the hip joint doesn't form properly. It can cause a variety of symptoms, including:

Limping

Waddling gait

Pain in the hip or groin

Stiffness in the hip

Unequal leg lengths

Limited range of motion in the hip

What signs indicate the disease is worsening?

If DDH is not treated, it can worsen over time. Signs that the disease is worsening include:

Increasing pain

Decreasing range of motion

Deformity of the hip

Difficulty walking

Should I seek immediate medical attention?

If you think your child may have DDH, it is important to see a doctor as soon as possible. Early diagnosis and treatment can help prevent long-term problems.

Here are some real-life examples or situations to help you better understand DDH:

A parent may notice that their child's legs are different lengths or that their child has a hard time moving their hip.

A doctor may notice that a child has a limp or waddling gait.

An older child or adult may experience pain or stiffness in their hip.

If you have any concerns about DDH, it is important to see a doctor.

This is for informational purposes only. For medical advice or diagnosis, consult a professional.

Absolutely! Let's talk about Developmental Dysplasia of the Hip (DDH) in a way that's easy to understand.

**What is DDH?**

Imagine your hip as a ball-and-socket joint. In DDH, the socket is too shallow, so the ball (the top of your thigh bone) doesn't fit snugly. This can make the hip loose or even cause it to pop out of place.

**How do doctors check for DDH?**

- **Physical exam:** Doctors gently move your baby's legs and hips to feel for any looseness or clicking.
- **Ultrasound:** This uses sound waves to create pictures of the hip joint. It's like a special camera that lets doctors see how the bones fit together.
- **X-ray:** This is like a regular camera that takes pictures of the bones. It's usually used for older babies and children whose bones are more developed.

**What do the test results mean?**

The test results help doctors see how severe the DDH is. They might describe it as:

- **Mild:** The hip is a little loose.
- **Moderate:** The hip is more loose and might pop out of place sometimes.
- **Severe:** The hip is completely out of place.

**What happens after diagnosis?**

If your child is diagnosed with DDH, they'll need regular checkups to make sure their hip is healing properly. The doctor will:

- **Check the hip:** They'll move the leg and hip to feel for any changes.
- **Do more tests:** They might do more ultrasounds or X-rays to see how the hip is progressing.
- **Adjust the treatment:** If needed, they might change the type of brace or harness your child wears.

**How often should I have follow-ups?**

The frequency of follow-ups depends on how severe the DDH is and how old your child is. In general, you can expect to see the doctor every few weeks or months until the hip is healed.

**What else should I know?**

- **Early treatment is important:** The sooner DDH is treated, the better the chances of a full recovery.
- **DDH is common:** It affects about 1 in 1,000 babies.
- **Most babies with DDH do well:** With treatment, most babies with DDH go on to have normal hips.

I hope this helps! If you have any other questions, please don't hesitate to ask.

Okay, let's talk about DDH and how to spot potential problems. Remember, I'm here to explain, not give medical advice. Always talk to a doctor for real medical concerns.

**What is DDH?**

Imagine your hip as a ball (the top of your thigh bone) fitting snugly into a socket (part of your hip bone). DDH is when that socket is too shallow, so the ball doesn't fit in right. It can be a little loose or even pop out.

**What are the signs of DDH in babies?**

DDH doesn't always cause pain, especially in babies, so it can be tricky to spot. Here are some things to look for:

- **Uneven leg creases:** When a baby lies on their back with their knees bent, do the skin folds on their thighs and buttocks look different on each side?
- **One leg seems shorter:** Does one leg seem shorter than the other?
- **Limited movement:** Does one hip seem less flexible or not move as far as the other?
- **Clicking or clunking:** Do you hear a click or clunk when you move the baby's hips?
- **Waddling or toe walking:** When they start walking, do they waddle or walk on their toes?

**What to do if you spot these signs?**

If you notice any of these signs, don't panic, but don't wait either. Make an appointment with your doctor. They can check your baby's hips and see if there's anything to worry about. Early treatment is important for DDH.

**How is DDH treated?**

Treatment depends on how old the child is and how severe the DDH is. Babies might wear a special harness to keep their hips in the right position. Older children might need surgery.

**Can I prevent DDH?**

There's no sure way to prevent DDH, but some things might help:

- **Swaddling:** If you swaddle your baby, make sure their legs can bend and move freely. Don't wrap them too tightly.
- **Car seats and carriers:** Choose car seats and carriers that support your baby's hips in a frog-like position, with their knees bent and their hips spread.

**Remember:**

- I'm not a doctor, so this isn't medical advice.
- Always talk to your doctor if you have any concerns about your child's health.

I hope this helps you understand DDH a little better. If you have any other questions, feel free to ask.

**What treatments will I receive during hospitalization? What is the purpose of each treatment?**

Treatment for DDH depends on the age of the child and the severity of the condition. In general, the goal of treatment is to put the hip back into the socket so it can develop normally.

Here are some common treatments for DDH in children:

- **Pavlik harness:** This is a soft brace that holds the baby's legs in a frog-like position. It is used for babies under 6 months old. The Pavlik harness helps to keep the hip in the socket so it can heal.
- **Closed reduction:** This is a procedure where the doctor moves the hip back into the socket without surgery. It is done under anesthesia.
- **Open reduction:** This is a surgery where the doctor makes an incision and moves the hip back into the socket. It is done when the closed reduction is not successful or when the hip is very unstable.
- **Spica cast:** This is a cast that covers the child's legs and torso. It is used after a closed or open reduction to hold the hip in place while it heals.

**How can I prevent infections or other complications during hospitalization? What can family members do to assist with recovery?**

There are several things you can do to prevent infections or other complications during hospitalization:

- **Keep the incision clean and dry.**
- **Wash your hands often, especially before and after touching your child.**
- **Make sure your child is up to date on all of their vaccinations.**
- **Follow the doctor's instructions for caring for the cast or brace.**

Family members can help with recovery by:

- **Providing emotional support.**
- **Helping with activities of daily living, such as bathing and dressing.**
- **Keeping the child's spirits up.**
- **Encouraging the child to move around as much as possible.**
- **Following the doctor's instructions for home care.**

**How can I reinforce the effects of treatment after discharge? What rehabilitation programs are recommended?**

After discharge, it is important to continue to follow the doctor's instructions. This may include:

- **Wearing a brace or cast.**
- **Going to physical therapy.**
- **Seeing the doctor for regular checkups.**

Rehabilitation programs can help your child regain strength and flexibility. They can also help your child learn how to move safely and avoid further injury.

Additional tips for reinforcing the effects of treatment after discharge:

- **Make sure your child gets plenty of rest.**
- **Encourage your child to eat a healthy diet.**
- **Help your child stay active.**
- **Be patient and supportive.**

It is important to remember that every child is different and will recover at their own pace. With patience and support, your child will be able to return to their normal activities.

t sounds like you're curious about how medications are used to treat developmental dysplasia of the hip (DDH) in children. That's a great question! However, it's important to know that **medications are not typically used to treat DDH**.

DDH is a condition where the hip joint doesn't form properly. It's like a puzzle piece that doesn't quite fit together. Instead of using medicine, doctors usually treat DDH with things like:

- **A Pavlik harness:** This is a special brace that gently holds the baby's legs in a position that helps the hip joint develop correctly. It's like training wheels for the hips!
- **Casting:** In some cases, a cast might be used to hold the hip in place.
- **Surgery:** If other treatments don't work, surgery might be needed to correct the hip problem.

**Since medications aren't used for DDH, the questions you asked about medications don't really apply in this case.**

It's always a good idea to talk to a doctor if you have concerns about DDH or any other health issue. They can give you the most accurate and personalized advice.

Okay, let's talk about developmental dysplasia of the hip (DDH) and pain. Imagine DDH as a puzzle where the hip joint pieces don't fit together perfectly from birth.

**What types of pain may be caused by DDH or its treatment? Where is the pain usually felt?**

DDH itself might not always cause pain, especially in babies. But as a child grows, if the hip isn't stable, they might feel pain in their hip, groin, or thigh. Think of it like a door that's not hung correctly. It might swing okay at first, but later, it could start to rub and become painful.

Treatments for DDH, like wearing a special brace (Pavlik harness), can sometimes cause discomfort too. It's like wearing new shoes that need to be broken in. There might be some rubbing or pressure. After surgery, there will be pain as the body heals. This is similar to how your arm might hurt after you get a shot.

**What methods can help relieve pain? What are the recommended pharmacological and non-pharmacological treatments?**

For pain from DDH or its treatment, doctors might suggest a few things:

- **Non-pharmacological treatments:** These are things that don't involve medicines. For babies in a Pavlik harness, sometimes adjusting the harness can help. Think of it as adjusting the straps on a backpack so it doesn't dig into your shoulders. Gentle massage or warm baths can also soothe discomfort. For older children, physical therapy can help strengthen the muscles around the hip and improve movement. It’s like exercise to help your muscles get stronger.
- **Pharmacological treatments:** These involve medicines. Doctors might recommend pain relievers like acetaminophen (like Tylenol) or ibuprofen (like Advil or Motrin). These medicines can help reduce pain and inflammation, like when you have a headache. It’s important to always use these medicines exactly as your doctor tells you.

**What steps should I take if the pain is more than expected or worsens? Do I need to contact my doctor to adjust my treatment plan?**

It's important to keep track of the pain. If the pain is getting worse, or if it's not getting better with the treatments, it's time to talk to the doctor. Think of it like this: if you have a toy that's broken, you might try to fix it yourself at first. But if it's still broken, you ask a grown-up for help. It's the same with pain. If you're concerned, it's always best to ask your doctor. They might need to adjust the treatment plan, like changing the type of brace or medicine. They’re the experts and can help make sure your hip heals properly.

It sounds like you or someone you know is going through surgery for developmental dysplasia of the hip (DDH). That can be a lot to take in, so let's break down the post-surgery care, recovery, and precautions in simple terms.

**What is DDH?**

Imagine your hip as a ball-and-socket joint. In DDH, the socket in the hip doesn't fully cover the ball of the thighbone. This can cause pain and problems moving the hip. Surgery helps to correct this.

**Post-Surgery Care**

After surgery for DDH, there are some important things to keep in mind:

- **Keep the hip stable:** This might mean using a special brace or cast to keep the hip in the correct position while it heals.
- **Pain management:** You'll likely have some pain after surgery. Your doctor will give you medicine to help with this.
- **Wound care:** Keep the incision clean and dry to prevent infection.
- **Follow-up appointments:** It's important to go to all your follow-up appointments so your doctor can check how your hip is healing.

**Returning to Normal Activities**

It's important to take it slow and not rush back into activities too quickly. Your doctor will tell you when it's safe to start doing more.

- **Early recovery:** You'll start with gentle exercises to help your hip heal. This might include moving your foot and ankle.
- **Later recovery:** As your hip gets stronger, you can start doing more activities, like walking and swimming.
- **Full recovery:** It can take several months or even a year to fully recover from DDH surgery.

**Recommended Activities During Recovery**

Your doctor or physical therapist will recommend specific exercises to help you recover. These might include:

- **Gentle range-of-motion exercises:** These help to keep your hip flexible.
- **Strengthening exercises:** These help to make the muscles around your hip stronger.
- **Weight-bearing exercises:** These help you to gradually put more weight on your hip.

**Scheduling Exercise**

Your physical therapist will help you create a plan for gradually increasing your activity level. It's important to follow their instructions carefully.

**Stages of Recovery and Precautions**

Recovery from DDH surgery happens in stages. Each stage has its own precautions:

- **Stage 1:** Focus on healing and pain management. You'll need to be careful not to put too much weight on your hip.
- **Stage 2:** Start gentle exercises to improve flexibility and strength. You'll still need to be careful not to overdo it.
- **Stage 3:** Gradually increase your activity level. You can start doing more activities, but you'll still need to be careful not to injure your hip.

**Specialized Rehabilitation Plan**

A specialized rehabilitation plan is important for a good recovery. A physical therapist can create a plan that is tailored to your specific needs. This plan will help you:

- **Heal properly:** The right exercises will help your hip heal correctly.
- **Regain strength and flexibility:** Your physical therapist will teach you exercises to make your hip strong and flexible again.
- **Prevent future problems:** A good rehabilitation plan can help to prevent problems with your hip in the future.

**Preventing Recurrence**

There are a few things you can do to help prevent DDH from coming back:

- **Follow your doctor's instructions:** This includes going to all your follow-up appointments and doing the exercises your physical therapist recommends.
- **Maintain a healthy weight:** This can help to take stress off your hip.
- **Use proper lifting techniques:** This can help to prevent injury to your hip.

**Complications**

As with any surgery, there is a risk of complications after DDH surgery. These can include:

- **Infection:** This can be treated with antibiotics.
- **Bleeding:** Some bleeding is normal after surgery.
- **Nerve damage:** This is rare, but it can happen.
- **Hip dislocation:** This is also rare, but it can happen if the hip is not properly supported.

If you have any concerns about complications, talk to your doctor.

This is for informational purposes only. For medical advice or diagnosis, consult a professional.

Okay, let's talk about Developmental Dysplasia of the Hip (DDH) and how to help your body heal. Imagine your hip is like a ball (the top of your thigh bone) fitting into a socket (part of your hip bone). With DDH, the socket might be too shallow, so the ball doesn't fit snugly and can even slip out.

**What to eat to help your hip heal:**

Think of your body as a construction site. To rebuild, you need the right materials!

- **Protein:** Builds and repairs muscles and tissues. Think chicken, fish, beans, or even Greek yogurt.
- **Calcium:** Makes bones strong. Milk, cheese, leafy greens, and fortified foods are good sources.
- **Vitamin D:** Helps your body absorb calcium. Sunshine helps, but you can also find it in fatty fish, eggs, and fortified milk.
- **Vitamin C:** Helps heal wounds and build collagen, which is like the "glue" holding tissues together. Citrus fruits, berries, and peppers are packed with it.
- **Iron:** Carries oxygen to your tissues, giving them energy to heal. Red meat, beans, and spinach are good sources.

**Foods that might not be the best for your hip right now:**

- **Processed foods:** These can be high in salt, unhealthy fats, and sugar, which can slow down healing. Think chips, sugary drinks, and fast food.
- **Sugary drinks:** They can cause inflammation, which isn't good for healing. Stick to water, milk, or unsweetened tea.
- **Too much alcohol:** It can interfere with healing and make pain worse.

**How to avoid constipation:**

- **Fiber:** It's like a natural "broom" for your gut. Whole grains, fruits, and veggies are full of it.
- **Water:** Keeps things moving smoothly. Aim for plenty of fluids throughout the day.
- **Exercise:** Even gentle movement helps your gut work. Talk to your doctor about what's safe for you.

**Exercises to help your hip heal:**

Your doctor or physical therapist will give you specific exercises, but here are some general ideas:

- **Gentle stretches:** Help improve flexibility and range of motion.
- **Strengthening exercises:** Make the muscles around your hip stronger to support it.
- **Low-impact activities:** Walking or swimming can be good for getting your body moving without putting too much stress on your hip.

**Precautions when exercising:**

- **Listen to your body:** If you feel pain, stop! It's okay to take breaks and rest.
- **Start slow:** Don't try to do too much too soon. Gradually increase the intensity and duration of your exercises.
- **Warm up:** Get your muscles ready for exercise with some light cardio or stretching.
- **Cool down:** Help your muscles recover with gentle stretching after exercise.

**Sleep and healing:**

- **DDH might make it harder to find a comfortable sleeping position.** Try using pillows to support your hip and keep it in a good alignment.
- **A regular sleep schedule helps your body heal.** Aim for 8-10 hours of sleep each night.
- **A relaxing bedtime routine can help you fall asleep.** Try a warm bath, reading a book, or listening to calming music.

**Remember, everyone heals at their own pace.** Be patient with yourself and celebrate small victories along the way. Your doctor and physical therapist are there to guide you and answer any questions you have.

**What psychological problems may be associated with developmental dysplasia of the hip (DDH) or postoperative recovery, such as anxiety or depression? How can I deal with them positively?**

DDH is a condition where the hip joint doesn't form properly. This can lead to pain, limping, and other problems. Treatment for DDH often involves surgery or wearing a cast or brace.

Some children with DDH or who have had surgery may experience anxiety or depression. Anxiety is a feeling of worry or fear. Depression is a feeling of sadness or hopelessness.

For example, a child with DDH may be anxious about falling or being unable to participate in activities with friends. A child who has had surgery may be depressed because they are in pain or cannot do the things they used to do.

It is important to remember that these feelings are normal. Many children experience anxiety or depression at some point in their lives. There are many things you can do to help your child deal with these feelings.

Here are some tips:

- Talk to your child about their feelings. Let them know that it is okay to be sad or scared.
- Help your child find healthy ways to cope with their feelings. This could include exercise, spending time with friends and family, or creative activities.
- Encourage your child to focus on the positive aspects of their life.
- If your child's anxiety or depression is severe, talk to their doctor. They may be able to recommend a therapist or other mental health professional.

**What are the signs that I need psychological support? How can I find professional help or support resources?**

If you are concerned about your child's mental health, there are some signs you can look for. These include:

- Changes in mood, such as being more irritable or withdrawn
- Changes in sleep or appetite
- Loss of interest in activities they used to enjoy
- Difficulty concentrating
- Feelings of hopelessness or worthlessness
- Thoughts of death or suicide

If you notice any of these signs, it is important to seek professional help. There are many resources available to help children with mental health problems.

Here are some ways to find help:

- Talk to your child's doctor. They can refer you to a therapist or other mental health professional.
- Contact your local mental health agency.
- Search online for mental health resources in your area.
- Talk to a trusted adult, such as a family member, friend, or teacher.

Remember, you are not alone. Many people care about you and want to help.

**Are there any recommended health apps or devices that can help manage DDH or postoperative recovery?**

Unfortunately, there aren't any specific apps or devices designed to directly manage DDH itself. DDH usually requires treatment by a doctor, like wearing a special brace or sometimes surgery.

However, some general health apps might be helpful for parents to keep track of things like:

- **Appointments:** You can use a calendar or reminder app to remember doctor's visits, therapy sessions, or follow-ups.
- **Notes:** A simple notes app can be used to jot down any questions you have for the doctor, or to record observations about your child's progress.

**How can I use health tracking tools to monitor my condition or recovery progress?**

Again, for DDH, the main tracking will be done by your doctor during checkups. They'll examine your child's hip and might take X-rays to see how things are progressing.

But, as a parent, you can also keep track of some things at home:

- **How often and for how long your child wears their brace:** If your child needs to wear a brace, you can use a simple chart or even a notes app to record how many hours a day they wear it. This can help you make sure they're following the doctor's instructions.
- **Your child's movements and development:** Note any changes in how your child moves, especially their legs and hips. Are they starting to crawl or walk? Are they favoring one side? This information can be useful to share with your doctor.

**Important Note:** Always talk to your doctor about the best ways to manage and track your child's DDH. They'll give you specific advice and instructions based on your child's needs.

**What are some ways to access reliable information about developmental dysplasia of the hip (DDH) or post-operative care?**

Imagine you're building a puzzle. You wouldn't want to use pieces from a different puzzle, right? It's the same with health information. You need reliable sources, not just any information.

Here are some good places to look for information about DDH:

- **Your doctor or healthcare provider:** They are the experts! They know your child's specific situation and can give you the most accurate advice.
- **Hospitals or clinics:** Many hospitals have websites or pamphlets with information about DDH. These are usually written by doctors and other healthcare professionals.
- **Reputable medical websites:** Some websites, like the ones from children's hospitals or medical associations, have reliable information about health conditions. Look for websites that end in ".org" or ".edu."
- **Books or pamphlets:** Your doctor or library might have books or pamphlets about DDH.

**Are there support groups or community resources that can help me or my family cope better?**

Dealing with DDH can be tough, both for the child and the family. It's like climbing a mountain – it's easier if you have others to help you along the way.

Here are some resources that can help:

- **Support groups:** These are groups of people who have been through similar experiences. They can share their stories, offer advice, and understand what you're going through.
- **Online forums:** There are online communities where you can connect with other families dealing with DDH.
- **Therapists or counselors:** These professionals can help you and your family manage the emotional challenges of DDH.
- **Local charities or organizations:** Some organizations might offer financial assistance or other resources for families dealing with DDH.

Remember, you're not alone! Many families have walked this path before, and there are people who can help you and your child.

The cost of treating developmental dysplasia of the hip (DDH) in children can vary.

**What is the usual cost of treatment for DDH?**

The cost of DDH treatment can vary. It depends on the type of treatment needed. For example, if your child only needs a Pavlik harness, the cost will be lower than if they need surgery.

**Are there insurance or subsidy policies to reduce the financial burden?**

Most health insurance plans cover the cost of DDH treatment. However, it's important to check with your insurance company to see what is covered. There may also be government programs that can help with the cost of treatment.

**What social assistance can I apply for if the disease or surgery causes financial hardship?**

If your child's DDH treatment causes financial hardship, there are programs that can help. For example, Medicaid is a government program that provides health insurance to low-income families. There may also be state or local programs that can help.

Here are some real-life examples or situations to help you understand the cost of DDH treatment:

- **Example 1:** A child with DDH only needs a Pavlik harness. The cost of treatment is $5,000. The family's health insurance covers 80% of the cost, so the family only has to pay $1,000.
- **Example 2:** A child with DDH needs surgery. The cost of treatment is $20,000. The family's health insurance covers 80% of the cost, so the family has to pay $4,000. The family is also able to get help from a government program that pays for 50% of the remaining cost. In the end, the family only has to pay $2,000.

I hope this helps you understand the cost of DDH treatment.

**How can I balance mental health, family responsibilities, and work in disease management? What are some practical suggestions?**

It can be tough to juggle everything when you're dealing with a health issue, whether it's for yourself or your child. It's like trying to keep all the balls in the air at once! But don't worry, there are ways to make it easier.

Let's take developmental dysplasia of the hip (DDH) in children as an example. Imagine you're a parent of a baby with DDH. You have to take them to doctor's appointments, make sure they wear their harness or cast, and maybe even do special exercises with them. On top of that, you might have other kids to take care of, a job to go to, and a house to run. It can feel overwhelming!

Here are some tips to help you balance everything:

- **Talk to your doctor or a therapist:** They can help you understand your child's condition and how to manage it. They can also help you deal with any stress or anxiety you might be feeling.
- **Join a support group:** Talking to other parents who are going through the same thing can be really helpful. You can share tips and support each other.
- **Ask for help:** Don't be afraid to ask for help from your family and friends. They can help you with things like childcare, errands, or even just listening when you need to talk.
- **Take care of yourself:** Make sure you're getting enough sleep, eating healthy foods, and exercising. It's important to take care of yourself so you can take care of your child.
- **Plan ahead:** Try to plan your days and weeks in advance. This will help you stay organized and make sure you're not forgetting anything.
- **Be flexible:** Things don't always go according to plan. Be prepared to adjust your plans as needed.

Remember, you're not alone. Many people are going through similar challenges. By taking care of yourself and asking for help, you can manage your child's DDH and still have a fulfilling life.

**How might DDH affect future quality of life? How can I plan ahead to improve my child's health?**

DDH can sometimes cause problems later in life, like hip pain or arthritis. But there are things you can do to help prevent these problems and improve your child's quality of life.

Here are some things to keep in mind:

- **Follow your doctor's instructions:** Make sure your child wears their harness or cast as directed, and that you do any special exercises with them.
- **Go to all of your child's appointments:** This will help your doctor monitor your child's progress and make sure they're getting the best possible care.
- **Talk to your doctor about long-term care:** Ask your doctor what you can do to help prevent problems later in life.
- **Encourage your child to be active:** Exercise is important for keeping your child's hips strong and healthy.
- **Be patient:** It can take time for your child's hips to heal. But with proper care, most children with DDH go on to live normal, healthy lives.

Remember, you're your child's biggest advocate. By working with your doctor and taking care of your child, you can help them have a bright and healthy future.

**Reply from Gemini 2.0 Flash (Second time)**

This is for informational purposes only. For medical advice or diagnosis, consult a professional.

Absolutely! Let's talk about Developmental Dysplasia of the Hip (DDH) in a way that's easy to understand.

**What is DDH?**

Imagine your hip joint like a ball (the top of your thighbone) fitting snugly into a socket (part of your hip bone). With DDH, this fit isn't quite right. The socket might be too shallow, or the ball might not be perfectly round, causing the hip to be loose or even slip out of place.

**Who is most likely to develop DDH?**

DDH is more common in:

- Girls
- Firstborn babies
- Babies born in a breech position (buttocks first)
- Babies with a family history of DDH

It affects about 1 in every 100 babies, so it's not super common, but it's important to be aware of.

**Will DDH have a long-term impact?**

The good news is that most babies with DDH can be successfully treated, especially if it's caught early. Treatment usually involves wearing a special harness that helps the hip joint develop properly.

**Here's what to pay special attention to:**

- **Early diagnosis is key.** The sooner DDH is detected, the easier it is to treat.
- **Follow your doctor's instructions carefully.** This might include wearing a harness for a certain amount of time or doing special exercises.
- **Keep an eye out for any signs of hip problems as your child grows.** This might include limping, one leg appearing shorter than the other, or difficulty moving the hip.

With proper treatment and care, most children with DDH go on to live normal, active lives.

**What parts of the body does DDH mainly affect? What do these parts do in a healthy state?**

DDH mainly affects the **hip joint**. The hip joint is where your thigh bone (femur) meets your pelvis. It's like a ball (the top of your thigh bone) fitting into a socket (part of your pelvis).

In a healthy hip, the ball fits snugly into the socket, allowing for a wide range of движения like walking, running, and jumping. The hip joint is also supported by strong ligaments that keep the ball in place.

**What symptoms or consequences may arise if these parts are damaged? Can you provide specific examples?**

If the hip joint is not formed correctly in DDH, the ball may not fit snugly into the socket. This can lead to:

- **Hip instability:** The hip may feel loose or wobbly, and it may even pop out of place. This can make it difficult to walk or move the leg.
- **Pain:** The hip may be painful, especially during activity.
- **Limp:** The child may develop a limp, or they may walk with a waddle.
- **Unequal leg length:** One leg may appear shorter than the other.
- **Delayed motor skills:** The child may be slow to reach developmental milestones like rolling over, crawling, or walking.

**Here are some specific examples:**

- A baby with DDH may have trouble spreading their legs apart during diaper changes.
- A toddler with DDH may limp or walk with a waddle.
- An older child with DDH may experience hip pain during sports or other activities.

It's important to remember that DDH is treatable, especially if it's diagnosed early. If you're concerned that your child may have DDH, it's important to see a doctor for diagnosis and treatment.

**What are the main causes of DDH? What risk factors may increase the chances of developing it?**

DDH is a condition where the hip joint doesn't form properly. The hip is a ball-and-socket joint. In DDH, the socket may be too shallow, and the ball may not fit snugly.

Doctors don't know exactly what causes DDH. But some things make it more likely:

- **Being a girl:** Girls are more likely to have DDH than boys.
- **Breech birth:** Babies who are born feet first are more likely to have DDH.
- **Family history:** DDH can run in families.

**What lifestyle habits or environmental factors increase the risk of developing DDH? How can I prevent it?**

There aren't any lifestyle habits or environmental factors that are known to cause DDH. But there are some things that can help prevent it:

- **Swaddling:** When you swaddle a baby, make sure their legs can bend and move freely. Don't wrap them too tightly.
- **Car seats:** Use car seats that are appropriate for your baby's age and size. Make sure the seat fits snugly and that your baby's legs can move.

**If I have a family history, is my risk of developing DDH higher? What preventive measures should I take?**

If you have a family history of DDH, your risk of developing it is higher. But there are some things you can do to help prevent it:

- **Talk to your doctor:** If you're concerned about DDH, talk to your doctor. They can examine your baby's hips and make sure they're developing properly.
- **Get regular checkups:** It's important to take your baby to all their well-child visits. During these visits, the doctor will check your baby's hips.
- **Follow your doctor's instructions:** If your doctor recommends any treatment for DDH, be sure to follow their instructions carefully.

**What are the main symptoms of developmental dysplasia of the hip (DDH)?**

DDH is a condition where the hip joint doesn't form properly. It can cause a number of symptoms, including:

- Limping or waddling
- One leg appearing shorter than the other
- Limited range of motion in the hip
- Clicking or popping sound in the hip
- Pain in the hip or groin

**What signs indicate the disease is worsening?**

If DDH is not treated, it can worsen over time. Signs that the disease is worsening include:

- Increasing pain
- Decreasing range of motion
- Deformity of the hip
- Difficulty walking

**Should I seek immediate medical attention?**

If you think your child may have DDH, it is important to see a doctor as soon as possible. Early diagnosis and treatment can help prevent long-term problems.

Here are some real-life examples or situations to help you better understand DDH:

- A parent may notice that their child's legs are different lengths when they are changing their diaper.
- A child may start to limp or waddle when they are learning to walk.
- A child may complain of pain in their hip or groin after playing sports.

If you notice any of these signs or symptoms, it is important to see a doctor for an evaluation.

This is for informational purposes only. For medical advice or diagnosis, consult a professional.

Absolutely! Let's talk about Developmental Dysplasia of the Hip (DDH) in a way that's easy to understand.

**What is DDH?**

Imagine your hip joint like a ball (the top of your thighbone) fitting into a socket (part of your hip bone). In DDH, the socket is too shallow, so the ball doesn't fit snugly. This can cause the hip to feel loose or even pop out of place.

**How do doctors check for DDH?**

Doctors use a few simple tests to check for DDH:

- **Physical exam:** They'll gently move your baby's legs and hips to feel for any looseness or clicking.
- **Ultrasound:** This uses sound waves to create pictures of the hip joint. It's like a special camera that can see through the skin.
- **X-ray:** This uses a small amount of radiation to take pictures of the bones in the hip. It's usually used for older babies and children.

**What do the test results mean?**

The test results will show how well the ball fits into the socket. If the ball is loose or not fitting well, it means your baby has DDH. The more loose the hip is, the more severe the DDH.

**What happens after diagnosis?**

Most babies with DDH need to wear a special brace called a Pavlik harness. This harness gently holds the hip in the correct position so it can heal. You'll need to take your baby to the doctor regularly to make sure the harness is fitting properly and the hip is healing.

**How often will I need follow-up appointments?**

The doctor will tell you how often your baby needs to be seen. It's usually every few weeks at first, then less often as the hip heals. At these appointments, the doctor will check the hip, adjust the harness, and make sure everything is going well.

**How can I learn more?**

If you have any questions about DDH, don't hesitate to ask your doctor. They're the best person to talk to about your baby's specific situation. You can also find more information online at websites like the American Academy of Orthopaedic Surgeons (AAOS) or the Children's Hospital of Philadelphia (CHOP).

It sounds like you're interested in learning more about developmental dysplasia of the hip (DDH). That's great! It's important to be informed about your health.

DDH is a condition where the hip joint doesn't form properly. It can happen in babies and children. Early diagnosis and treatment are important for the best outcome.

**What are the signs that I should see a doctor right away?**

With DDH, it's not always an emergency, but you should see a doctor soon if you notice any of these signs in a baby or child:

- **Uneven leg creases:** When a baby lies on their back with their knees bent, the skin folds on their thighs and buttocks might not match up.
- **One leg seems shorter:** One leg might look shorter than the other.
- **Limited movement:** The baby's hip might not move as much as it should, especially when trying to spread the legs out.
- **Clicking or clunking sound:** You might hear a clicking or clunking sound when you move the baby's hip.
- **Limping or waddling:** An older child might limp or waddle when walking.
- **Delayed motor skills:** The child might be slow to reach milestones like rolling over, crawling, or walking.

**What should I do in an emergency?**

DDH is usually not an emergency. However, if your child is in severe pain or cannot move their leg at all, you should seek medical attention right away. This could indicate a more serious problem, like a hip dislocation.

Here are some things you can do while waiting to see a doctor:

- **Keep the child comfortable:** Gently hold the child and avoid moving their leg too much.
- **Do not try to fix the problem yourself:** Do not try to pop the hip back into place or force any movement.
- **Call your doctor or go to the emergency room:** Explain the situation and follow their instructions.

Remember, it's always better to be safe and seek medical advice if you're concerned about your child's health.

**What treatments will I receive during hospitalization? What is the purpose of each treatment?**

If your child has DDH, they may need to wear a special brace or cast to help their hip joint grow properly. This is like wearing a seatbelt for your hips! The brace or cast will hold the hip in the correct position so that the ball and socket can fit together snugly as they grow. This may take several weeks or months, and your child may need to stay in the hospital for a short time to get used to the brace or cast.

In some cases, surgery may be necessary to correct DDH. This is like fixing a broken toy so it works properly again. The surgeon will make a small cut in your child's hip and realign the bones so they fit together correctly. After surgery, your child will need to wear a cast or brace to protect their hip while it heals.

**How can I prevent infections or other complications during hospitalization? What can family members do to assist with recovery?**

To prevent infections, it's important to keep the area around your child's hip clean and dry. This means washing your hands often, especially before and after changing your child's diaper or touching their brace or cast. You should also make sure that your child's clothes and bedding are clean.

Family members can help with recovery by providing love and support. This means spending time with your child, playing games, reading stories, and helping them stay positive. It's also important to follow the doctor's instructions carefully and make sure your child goes to all their follow-up appointments.

**How can I reinforce the effects of treatment after discharge? What rehabilitation programs are recommended?**

After your child leaves the hospital, it's important to continue following the doctor's instructions. This may include wearing a brace or cast for a certain amount of time each day, doing special exercises, and going to physical therapy. Physical therapy is like going to a gym for your hip! The therapist will teach your child exercises to strengthen their hip muscles and improve their range of motion.

It's also important to make sure your child eats a healthy diet and gets plenty of rest. This will help their body heal and grow strong. With your love and support, your child will be able to overcome DDH and live a happy, healthy life.

This is for informational purposes only. For medical advice or diagnosis, consult a professional.

This is for informational purposes only. For medical advice or diagnosis, consult a professional.

Developmental dysplasia of the hip (DDH) is a condition where the hip joint doesn't form properly in babies and young children. It's like a puzzle piece that doesn't quite fit together.

**Common Treatments:**

- **Pavlik Harness:** This is a special brace that gently holds the baby's hips in the correct position, allowing them to grow normally. It's like training wheels for the hips!

**Important Notes:**

- **Always follow your doctor's instructions carefully.** They know what's best for you.
- **Never adjust the Pavlik harness yourself.** Let your doctor do it to ensure it's positioned correctly.

**What if I miss a dose or take it at the wrong time?**

- **Call your doctor or healthcare provider right away.** They will tell you what to do.
- **Don't take a double dose to make up for a missed one.** This could be harmful.

**What foods should I avoid?**

- **There are no specific foods to avoid while using the Pavlik harness.** However, it's always a good idea to eat a healthy diet to support your overall growth and development.

**Common side effects:**

- **Skin irritation:** The harness may rub against the skin, causing redness or irritation.
- **Call your doctor if you notice any skin problems.** They can help you manage them.

**Remember:**

- **DDH is usually treatable, especially when caught early.**
- **With proper treatment, most children with DDH go on to live normal, healthy lives.**

**Additional resources:**

- **KidsHealth:** <https://kidshealth.org/en/parents/ddh.html>
- **Nemours KidsHealth:** <https://kidshealth.org/en/parents/ddh.html>
- **Seattle Children's Hospital:** <https://www.seattlechildrens.org/clinics/orthopedics/child-and-young-adult-hip-preservation-program/>

I hope this information is helpful! If you have any other questions, please don't hesitate to ask.

**What types of pain may be caused by developmental dysplasia of the hip (DDH) or its treatment? Where is the pain usually felt?**

Imagine your hip joint like a ball (the top of your thigh bone) fitting snugly into a socket (part of your hip bone). With DDH, the socket might be too shallow, so the ball doesn't fit well and can slip out.

**Pain from DDH itself:**

Babies with DDH usually don't feel pain. But if DDH isn't treated, older kids or teens might feel:

- **Hip pain:** This might feel like a dull ache or a sharp pain, especially when they move their leg or walk.
- **Groin pain:** The groin is the area where your thigh meets your tummy.
- **Knee pain:** Sometimes, the pain is felt in the knee instead of the hip.

**Pain from DDH treatment:**

- **Pavlik harness:** This is a soft brace that babies wear to help their hips grow correctly. It might rub and cause some skin irritation or discomfort.
- **Surgery:** If surgery is needed, there will be pain afterward. Doctors will give medicine to help with this.

**What methods can help relieve pain? What are the recommended pharmacological and non-pharmacological treatments?**

**For pain from DDH itself (in older kids/teens):**

- **Rest:** Taking a break from activities that make the pain worse can help.
- **Physical therapy:** Exercises can help strengthen the muscles around the hip and make it more stable.
- **Pain medicine:** Doctors might suggest over-the-counter pain relievers like acetaminophen or ibuprofen. For more severe pain, they might prescribe stronger medicine.

**For pain from Pavlik harness:**

- **Check the straps:** Make sure the straps aren't too tight or rubbing the skin.
- **Skin care:** Keep the skin clean and dry. Talk to your doctor about creams or powders that can help with irritation.

**For pain after surgery:**

- **Pain medicine:** Doctors will prescribe pain medicine to take at home.
- **Rest:** It's important to rest and let the hip heal.
- **Physical therapy:** Exercises will help you regain strength and movement.

**What steps should I take if the pain is more than expected or worsens? Do I need to contact my doctor to adjust my treatment plan?**

Yes, always tell your doctor if the pain:

- **Gets worse:** If the pain is getting more intense or doesn't go away with medicine.
- **Changes:** If the pain changes location or feels different.
- **Affects daily life:** If the pain is keeping you from doing things you enjoy.

Your doctor might want to:

- **Examine you:** To make sure there are no problems.
- **Change your treatment:** They might adjust your medicine, recommend different exercises, or suggest other treatments.

It's always better to be safe and talk to your doctor if you're worried about pain. They're there to help you!

It sounds like you or someone you know is dealing with DDH surgery. That can be a lot to take in, so let's break down the post-surgery care, recovery, and precautions in simple terms.

**What precautions should I take after surgery?**

Imagine your hip is like a puzzle, and surgery was like putting a tricky piece in the right spot. You want to keep that piece secure while it heals. That means:

- **No roughhousing:** Avoid activities like jumping, running, or anything that could jolt your hip.
- **Gentle movements:** Focus on slow, controlled movements as instructed by your doctor or physical therapist.
- **Keep it clean:** Follow instructions on caring for the incision site to prevent infection.
- **Follow weight-bearing instructions:** Your doctor will tell you how much weight you can put on your leg. It might be none at first, then gradually increase.
- **Use assistive devices:** Crutches or a walker might be needed to help you move around safely.
- **Attend all follow-up appointments:** This is crucial for your doctor to check your progress and make sure everything is healing well.

**How long until I can return to normal activities?**

Think of recovery like climbing a ladder. You start at the bottom and gradually work your way up. It takes time for your hip to heal, so you can't rush it. The timeline varies depending on the person and the specific surgery. It could take several weeks or even months before you can return to all your normal activities.

**How soon until I can resume some activities?**

Even before you can return to all normal activities, you can start doing some gentle exercises. This helps your hip get stronger and more flexible. Your doctor or physical therapist will guide you on what's safe and when.

**What activities are recommended during recovery?**

- **Gentle exercises:** These might include things like ankle pumps, leg lifts, or gentle stretches.
- **Walking:** Short walks are great for getting your body moving, but don't overdo it.
- **Swimming:** Once your incision has healed, swimming can be a great low-impact exercise.
- **Play therapy:** For children, play therapy can help them regain strength and mobility in a fun way.

**How do I schedule my exercise?**

Your doctor or physical therapist will create a personalized exercise plan for you. They'll tell you what exercises to do, how often, and how many repetitions.

**What are the recovery stages and precautions for each stage?**

Recovery usually involves different stages:

- **Early stage:** Focus is on pain management and preventing infection. You'll likely be resting a lot and using crutches or a walker.
- **Middle stage:** You'll start doing more exercises to improve strength and flexibility. You might start putting more weight on your leg.
- **Late stage:** You'll gradually return to your normal activities. You'll continue doing exercises to maintain your strength and flexibility.

Your doctor or physical therapist will tell you what to expect at each stage and what precautions to take.

**Do I need a specialized rehabilitation plan?**

Yes, a specialized rehabilitation plan is very important. It's like a roadmap that guides you through your recovery. It helps you regain your strength, flexibility, and mobility safely and effectively.

**How can I prevent recurrence after surgery?**

- **Follow your doctor's instructions:** This is the most important thing you can do.
- **Maintain a healthy weight:** This puts less stress on your hip.
- **Continue doing your exercises:** Even after you've recovered, it's important to keep your hip strong and flexible.

**What complications can occur and what should I do?**

Complications are rare, but they can happen. They might include infection, blood clots, or nerve damage. If you experience any unusual pain, swelling, redness, or fever, contact your doctor right away.

Remember, everyone's recovery is different. Be patient with yourself and don't get discouraged if you don't see results immediately. Just keep following your doctor's instructions and you'll be on your way to a full recovery.

**What foods are helpful in recovery from developmental dysplasia of the hip (DDH) or during recovery from surgery? What foods may aggravate the condition?**

A healthy diet is important for everyone, including children with DDH. Eating plenty of fruits, vegetables, and whole grains can help your body heal and stay strong. These foods provide the vitamins and minerals your body needs.

For example, imagine your body is like a construction site. Fruits and vegetables are like the workers bringing in the materials, and whole grains are like the cement that holds everything together.

Some foods, like sugary drinks and processed snacks, don't have many nutrients and may not be helpful for recovery. It's best to talk to a doctor or a registered dietitian for personalized advice.

**How should I adjust my diet to prevent constipation or other common problems?**

Constipation can sometimes be a problem after surgery or while wearing a cast or brace. Eating foods high in fiber, like fruits, vegetables, and whole grains, can help prevent constipation. Drinking plenty of water is also important.

Think of fiber as a broom that sweeps through your intestines and helps things move along. Water is like the oil that keeps everything running smoothly.

**What exercises can I do during the recovery phase? How often should I do them?**

The exercises you can do will depend on your specific situation and what your doctor recommends. They might include gentle stretches or movements to help strengthen the muscles around your hip.

It's important to follow your doctor's instructions carefully and not do any exercises that cause pain. Think of it like learning a new dance routine. You start with the basic steps and gradually add more complex moves as you get better.

**What precautions should I take when exercising, especially during postoperative recovery?**

After surgery, it's important to be careful and not put too much stress on your hip. Your doctor will give you specific instructions on what you can and cannot do.

In general, it's a good idea to avoid high-impact activities, like running or jumping, until your doctor says it's okay. It's like protecting a delicate flower. You wouldn't want to throw a ball at it, would you?

**Does DDH affect the quality of my sleep? What are some ways to improve sleep and help the body recover?**

Sometimes, DDH or the treatments for it can make it difficult to sleep comfortably. Creating a relaxing bedtime routine, like taking a warm bath or reading a book, can help. Making sure your bedroom is dark, quiet, and cool can also improve sleep.

Think of sleep as your body's repair time. It's when your body fixes any damage and gets ready for the next day. Getting enough sleep is like giving your body a tune-up.

**What psychological problems may be associated with developmental dysplasia of the hip (DDH) or postoperative recovery, such as anxiety or depression? How can I deal with them positively?**

DDH is a condition where the hip joint doesn't form properly. This can lead to pain, difficulty walking, and other problems. Treatment for DDH often involves surgery, casting, or bracing.

It's normal for kids with DDH to feel worried, scared, or even sad. They might worry about:

- The pain
- Missing school or playing with friends
- What other kids think
- Being different

Here are some ways to help kids deal with these feelings:

- Talk about it: Encourage kids to talk about how they're feeling. Listen patiently and offer support.
- Be positive: Help kids focus on the positive aspects of their treatment, like getting better and being able to do more things.
- Find distractions: Help kids find fun things to do that can take their mind off their worries, like reading, playing games, or watching movies.
- Connect with others: Let kids know they're not alone. Connect them with other kids who have DDH or have gone through similar treatments.

**What are the signs that I need psychological support? How can I find professional help or support resources?**

If you notice any of these signs, it might be helpful to seek psychological support:

- Increased anxiety or worry
- Sadness or crying more often
- Irritability or anger
- Difficulty sleeping or eating
- Loss of interest in things they used to enjoy
- Withdrawing from friends and family

If you're concerned, talk to your doctor or another healthcare provider. They can help you find a mental health professional who can provide support and treatment.

Here are some other resources that can help:

- The American Academy of Pediatrics
- The American Psychological Association
- The National Institute of Mental Health

Remember, it's okay to ask for help. Getting support can make a big difference in how kids cope with DDH and its treatment.

This is for informational purposes only. For medical advice or diagnosis, consult a professional.

Absolutely! Let's talk about DDH and how some tools can help.

**What is DDH?**

Imagine your hip joint like a ball (femoral head) fitting snugly into a socket (acetabulum). With DDH, the socket is too shallow, so the ball doesn't fit well and can even slip out. It's like a door that doesn't close properly.

**How can apps and devices help?**

While there aren't specific apps or devices to "fix" DDH, some can help with monitoring and recovery:

- **HipScreen App:** This app helps healthcare providers and families understand hip surveillance programs for children with cerebral palsy, which can sometimes involve DDH. It has tools for measuring X-rays and understanding guidelines.

**How can tracking tools help?**

- **Keeping track of appointments:** You can use a calendar app or a simple notebook to record doctor's appointments, therapy sessions, and any important notes about your child's progress.

**Important Note:** Always talk to your doctor about the best ways to manage DDH and use any tools. They can give you personalized advice and make sure everything is going well.

It's great you're looking for information and support! Here's a breakdown of how to find reliable resources and support for Developmental Dysplasia of the Hip (DDH):

**Finding Reliable Information**

- **Your Doctor is Your Best Resource:** They know your child's specific situation and can give you the most accurate advice. Don't hesitate to ask them questions!
- **Reputable Websites:** Look for websites from trusted organizations like the American Academy of Orthopaedic Surgeons (AAOS) or the Pediatric Orthopaedic Society of North America (POSNA). These sites have information written by experts.
- **Libraries:** Your local library or school library can be a treasure trove of information. Librarians can help you find books or articles that are accurate and easy to understand.

**Important Note:** Be careful about where you get your information. Some websites might have outdated or incorrect information. Always double-check with your doctor if you're unsure about something you read online.

**Support Groups and Community Resources**

- **Parent Support Groups:** Connecting with other parents who have gone through similar experiences can be incredibly helpful. You can share stories, ask questions, and get advice from people who understand what you're going through.
- **Online Communities:** There are many online forums and social media groups where you can connect with other families affected by DDH. These can be a great source of support and information.
- **Local Resources:** Your doctor or hospital might be able to connect you with local resources, such as therapists, counselors, or organizations that offer support to families with children who have DDH.

**Remember:** You're not alone in this! There are many resources available to help you and your family cope with DDH. Don't be afraid to reach out and ask for help when you need it.

It's tough to put an exact price tag on treating developmental dysplasia of the hip (DDH) in kids. It's like asking how much it costs to fix a car – it depends on what's broken!

For DDH, the cost depends on a few things:

- **How old the child is:** Younger babies might just need a special brace, while older kids might need surgery.
- **How severe the DDH is:** A little looseness in the hip is easier to fix than a hip that's completely out of joint.
- **Where you live:** Medical costs can be different in different places.
- **What kind of treatment is needed:** Braces, casts, physical therapy, and surgery all have different costs.

**Think of it like this:** Imagine your friend needs a new bike. If they just need a tire pump, that's cheap. But if they need a whole new wheel, that's more expensive. DDH treatment is similar – simple fixes cost less than big ones.

**Now, about help with costs:**

- **Insurance:** Most health insurance plans will help pay for DDH treatment. It's like car insurance – it helps cover the costs when something goes wrong.
- **Subsidies:** Some countries or states have programs to help families with medical costs. It's like getting a discount at the grocery store – it makes things a little more affordable.

**If money is tight:**

Sometimes, even with insurance, it's hard to pay for everything. If your family is having a tough time, there might be programs that can help:

- **Social assistance:** This is like getting help from your community. It could be money for food, housing, or medical bills.
- **Charities:** Some groups help families with medical needs. It's like when people donate toys to kids at Christmas – they're helping those who need it.

**To find out what help is available:**

- **Talk to your doctor or the hospital:** They know a lot about DDH and might know about programs that can help.
- **Check with your local government:** They often have websites or offices that can tell you about social assistance programs.
- **Search online:** You can find lots of information about charities and other groups that help families with medical costs.

Remember, it's okay to ask for help! Lots of families go through tough times, and there are people who want to help make things easier.

**How can I balance mental health, family responsibilities, and work in disease management? What are some practical suggestions?**

It can be tough to juggle everything when you're dealing with a health issue, whether it's your own or a family member's. It's like trying to keep all the balls in the air at once! But don't worry, here are some tips to help you find a balance:

- **Take care of your mental health:** It's important to remember that you're not alone in this. Talking to a therapist, counselor, or support group can help you manage stress and anxiety. Think of it like having a coach to help you through the tough times.
- **Prioritize:** You don't have to do everything. Focus on the most important things, like your health, your family, and your work. Let go of the things that are less important. It's like deciding which toys to bring on a trip – you can't bring them all!
- **Ask for help:** Don't be afraid to ask for help from your family, friends, or community. They can help with things like childcare, errands, or even just listening when you need to talk. Remember, everyone needs a helping hand sometimes.
- **Plan ahead:** Planning can help you feel more in control. Make a schedule for your week, including appointments, work, and family time. This can help you stay organized and make sure you're taking care of everything. It's like packing your bag for a trip – it's always better to be prepared!
- **Take breaks:** It's important to take breaks for yourself, even if it's just for a few minutes each day. Do something you enjoy, like reading, listening to music, or spending time in nature. This will help you recharge and come back to your responsibilities feeling refreshed. Think of it like taking a pit stop on a long road trip – it helps you keep going!

**How might developmental dysplasia of the hip (DDH) affect future quality of life? How can I plan ahead to improve my health?**

DDH is a condition where the hip joint doesn't form properly. It can cause pain, stiffness, and difficulty walking. But don't worry, there are things you can do to improve your health and quality of life.

- **Early diagnosis and treatment:** The earlier DDH is diagnosed and treated, the better the outcome. So if you're concerned about your child's hip development, talk to your doctor right away. It's like getting a small crack in a window fixed before it spreads.
- **Physical therapy:** Physical therapy can help strengthen the muscles around the hip and improve flexibility. This can help reduce pain and improve function. Think of it like exercise for your hips!
- **Assistive devices:** In some cases, assistive devices like crutches or a walker may be needed. These can help you get around safely and comfortably. It's like having a helpful tool to make things easier.
- **Surgery:** In some cases, surgery may be needed to correct the hip problem. This can be a big decision, but it can also significantly improve your quality of life. It's like getting a tune-up for your car to make it run better.
- **Long-term follow-up:** It's important to see your doctor for regular checkups to make sure your hip is healing properly and to address any concerns. It's like going to the dentist for regular checkups to keep your teeth healthy.

Living with DDH can be challenging, but it doesn't have to hold you back. By taking care of your health and working with your doctor, you can live a full and active life. Remember, you're not alone in this!

**Reply from Gemini 2.0 Flash (Third time)**

**What is developmental dysplasia of the hip (DDH)?**

Imagine your hip as a ball-and-socket joint. The top of your thigh bone (the ball) fits into a curved space in your hip bone (the socket). In DDH, the socket is too shallow, so the ball doesn't fit in snugly. It might be loose or even pop out.

**Who is most likely to develop DDH?**

DDH is more common in:

- Girls
- Firstborn babies
- Babies who were in the breech position (buttocks first) in the womb
- Babies with a family history of DDH

About 1 in 1,000 babies are born with DDH.

**Will DDH have a long-term impact on my quality of life?**

If DDH is found and treated early, most children grow up with no problems. Treatment might involve a special harness or cast to help the hip joint develop properly.

If DDH isn't treated, it can cause problems later in life, such as:

- Hip pain
- Limping
- Arthritis

It's important to get regular checkups so your doctor can make sure your hips are healthy.

This is for informational purposes only. For medical advice or diagnosis, consult a professional.

Absolutely! Let's talk about Developmental Dysplasia of the Hip (DDH) in a way that's easy to understand.

**What parts of the body does DDH mainly affect?**

DDH mainly affects the **hip joint**. This is where your thigh bone (femur) meets your pelvis. Think of it like a ball (the top of your thigh bone) fitting into a socket (part of your pelvis). In a healthy hip, the ball fits snugly into the socket, allowing for smooth movement.

**What happens in DDH?**

In DDH, the socket is too shallow, so the ball doesn't fit in properly. This can cause the hip to be loose or even dislocate (come out of the socket).

**What symptoms or consequences may arise if these parts are damaged?**

DDH often doesn't cause pain in babies, so it can be hard to spot. Some signs include:

- One leg may appear shorter than the other.
- The folds of skin on the thighs or buttocks may look uneven.
- The hip may make a clicking or popping sound when moved.

If DDH isn't treated, it can lead to problems later in life, such as:

- Limping
- Hip pain
- Arthritis

**Can you provide specific examples?**

Imagine a baby whose hip is dislocated. They might have trouble moving that leg, and it might look shorter than the other leg. As they grow older, they might develop a limp or have pain in their hip.

**Important Note:** If you're concerned about your child's hip health, it's always best to talk to a doctor. They can properly diagnose DDH and recommend the best course of treatment.

**What are the main causes of developmental dysplasia of the hip (DDH)? What risk factors may increase the chances of developing it?**

DDH means the hip joint doesn't form correctly. The hip is a ball-and-socket joint. The top of the thigh bone (femur) is the ball. It fits into a socket in the hip bone. In DDH, the socket may be too shallow. Or the ball may not fit well in the socket.

We don't know exactly what causes DDH. But some things make it more likely:

- **Being a girl:** Girls are more likely to have DDH than boys.
- **Breech birth:** Babies who are born feet first are more likely to have DDH.
- **Family history:** DDH can run in families.

**What lifestyle habits or environmental factors increase the risk of developing DDH? How can I prevent it?**

There aren't any lifestyle habits that cause DDH. But some things can help prevent it:

- **Swaddling:** When you wrap a baby, make sure their legs can bend and move freely. Don't wrap them too tightly.
- **Car seats:** Use car seats that fit your child's age and size. Make sure their legs can move.

**If I have a family history, is my risk of developing DDH higher? What preventive measures should I take?**

Yes, if someone in your family has DDH, you're more likely to have it too. If you're worried, talk to your doctor. They can check your hips. They may also suggest other things to do, like getting an ultrasound.

**What are the main symptoms of developmental dysplasia of the hip (DDH)?**

DDH means a baby's hip joint doesn't fit together properly. It's like a ball (the top of the thighbone) and socket (part of the hip bone) that don't quite match up.

Some babies with DDH might not have any symptoms. But here are some things you might notice:

- **Uneven leg creases:** The skin folds on the back of the legs might not look the same on both sides.
- **One leg turns out:** One leg might turn outward more than the other.
- **Limited movement:** The baby might not move one leg as well as the other.
- **Clicking or clunking:** You might hear a clicking or clunking sound when you move the baby's hips.

**What signs indicate the disease is worsening?**

If DDH isn't treated, it can get worse. Here are some signs:

- **Delayed development:** The baby might be late in learning to roll over, sit, or walk.
- **Limping:** When the child starts walking, they might limp.
- **Pain:** The child might start to feel pain in their hip.

**Should I seek immediate medical attention?**

If you think your baby might have DDH, it's important to see a doctor as soon as possible. Early treatment can help prevent problems later in life.

This is for informational purposes only. For medical advice or diagnosis, consult a professional.

Absolutely! Let's talk about Developmental Dysplasia of the Hip (DDH) in a way that's easy to understand.

**What is DDH?**

Imagine your hip joint like a ball (the top of your thighbone) fitting snugly into a socket (part of your hip bone). With DDH, this socket is too shallow, so the ball doesn't fit in properly. It's like a loose tooth in a wobbly socket. This can cause the hip to feel unstable and might even pop out of place.

**How do doctors check for DDH?**

Doctors have some clever ways to see if a baby has DDH:

- **Physical exam:** They gently move the baby's legs and hips to feel for any looseness or clicking sounds. It's like checking if a door opens and closes smoothly.
- **Ultrasound:** This uses sound waves to create a picture of the hip joint. It's like using a special camera to see inside the body without X-rays.
- **X-ray:** This uses radiation to take pictures of the bones. It's like taking a regular photo, but it shows the bones instead of the outside of the body.

**What do the tests show?**

The tests help doctors see how well the ball fits into the socket. If the ball is loose or not sitting in the socket properly, it means the baby has DDH. The more loose or out of place the ball is, the more severe the DDH.

**What happens after diagnosis?**

If a baby is diagnosed with DDH, they might need to wear a special brace called a Pavlik harness. This harness helps keep the hip in the correct position so it can grow properly. It's like wearing a cast for a broken arm, but it's for the hip.

Babies with DDH will need regular checkups with their doctor to make sure the hip is healing well. The doctor will probably do more physical exams and maybe some more ultrasounds or X-rays. It's like going to the dentist for regular checkups to make sure your teeth are healthy.

**Remember:**

- DDH is usually diagnosed in babies, but it can sometimes be missed and diagnosed later in childhood.
- Early diagnosis and treatment are important for DDH. The sooner it's treated, the better the chances of a full recovery.
- Most babies with DDH who are treated early will have normal hip function.

I hope this helps you understand DDH better! If you have any more questions, feel free to ask.

Developmental dysplasia of the hip (DDH) is a condition in which the hip joint doesn't form properly. It can cause pain, clicking or popping in the hip, and limping. In severe cases, it can even lead to disability.

**What symptoms indicate that I need to seek immediate medical attention?**

If your child has any of the following symptoms, you should seek immediate medical attention:

- Severe pain in the hip
- Inability to move the hip
- Deformity of the hip
- Signs of infection, such as fever, redness, or swelling

**How should I handle and self-monitor during an emergency situation?**

If your child is experiencing any of the above symptoms, you should:

1. Call your doctor or 911 immediately.
2. Keep your child calm and still.
3. Do not try to move the hip or force it into place.
4. Apply ice to the hip to reduce pain and swelling.
5. If your child is bleeding, apply pressure to the wound.

Here are some real-life examples or situations to help you better understand:

- Imagine your child is playing in the park and suddenly falls. They are unable to move their leg and are in a lot of pain. This is an emergency situation and you should seek immediate medical attention.
- Imagine your child has been diagnosed with DDH and is wearing a brace. One day, you notice that the brace is causing redness and swelling on their skin. This is also an emergency situation and you should seek immediate medical attention.

It is important to remember that DDH is a serious condition that can lead to long-term problems if left untreated. If you are concerned about your child's hip health, please see a doctor for evaluation.

**What treatments will I receive during hospitalization? What is the purpose of each treatment?**

Treatment for DDH depends on the age of the child and the severity of the condition. In general, the goal of treatment is to put the hip back into its normal position.

- **Pavlik harness:** This is a soft brace that is used for babies up to 6 months old. It holds the baby's legs in a frog-like position, which helps to keep the hip in the socket.
- **Closed reduction:** This is a procedure in which the doctor moves the hip back into the socket without surgery. It is usually done under anesthesia.
- **Open reduction:** This is a surgery in which the doctor makes an incision and moves the hip back into the socket. It is usually done for older children or when closed reduction is not successful.
- **Hip spica cast:** This is a cast that holds the hip in place while it heals. It is usually used after closed or open reduction.

**How can I prevent infections or other complications during hospitalization? What can family members do to assist with recovery?**

- **Keep the incision clean and dry:** If your child has surgery, it is important to keep the incision clean and dry to prevent infection. Your doctor will give you instructions on how to care for the incision.
- **Watch for signs of infection:** Signs of infection include fever, redness, swelling, and drainage from the incision. If you notice any of these signs, call your doctor right away.
- **Follow your doctor's instructions:** It is important to follow your doctor's instructions carefully to ensure that your child recovers properly. This includes keeping the hip in the correct position, attending all follow-up appointments, and following any restrictions on activity.
- **Provide support and encouragement:** Family members can help with recovery by providing support and encouragement. This includes helping with daily activities, providing emotional support, and celebrating milestones.

**How can I reinforce the effects of treatment after discharge? What rehabilitation programs are recommended?**

- **Continue to follow your doctor's instructions:** It is important to continue to follow your doctor's instructions after discharge. This includes keeping the hip in the correct position, attending all follow-up appointments, and following any restrictions on activity.
- **Physical therapy:** Your doctor may recommend physical therapy to help your child regain strength and flexibility in the hip.
- **Occupational therapy:** Your doctor may recommend occupational therapy to help your child learn how to do daily activities, such as dressing and bathing, while protecting the hip.
- **Regular checkups:** It is important to take your child for regular checkups to make sure that the hip is healing properly.

With proper treatment, most children with DDH will go on to live normal, healthy lives.

It sounds like you're curious about how medications are used to treat developmental dysplasia of the hip (DDH) in children. That's a great question! However, it's important to know that **medications are not typically used to treat DDH**.

DDH is a condition where the hip joint doesn't form properly. Think of it like a ball and socket joint, where the ball (the top of the thighbone) should fit snugly into the socket (part of the hip bone). In DDH, the socket may be too shallow, or the ball may not be the right shape, so they don't fit together well.

**How is DDH usually treated then?**

The main treatments for DDH focus on helping the hip joint develop correctly. Here are the most common ones:

- **Pavlik Harness:** This is like a soft brace that babies wear to keep their legs in a position that helps the hip joint develop properly. Imagine it like training wheels for the hips!
- **Other Braces or Casts:** Older babies or children might need different types of braces or casts to hold their hip in the correct position.
- **Surgery:** In some cases, surgery might be needed to correct the hip joint.

**Why no medication?**

Medications usually work by targeting specific problems in the body, like reducing inflammation or fighting infection. DDH is a structural issue, meaning it's about the shape and fit of the bones. So, medication wouldn't be able to fix that. It's like trying to fix a wobbly table by taking medicine instead of tightening the screws!

**Important Note:**

If your child has been diagnosed with DDH, it's really important to talk to your doctor. They can explain the best treatment plan for your child's specific situation. They can also answer any questions you have about the treatments.

I hope this explanation helps!

Okay, let's talk about DDH and pain.

**What types of pain may be caused by DDH or its treatment? Where is the pain usually felt?**

Imagine your hip is like a ball fitting into a socket. With DDH, the socket might be too shallow, so the ball doesn't fit snugly. This can cause pain, especially when moving the leg. Think of it like a wobbly toy – it might hurt if you try to play with it too much. The pain is usually felt in the hip, groin, or thigh. Sometimes, kids with DDH might also have knee pain. Treatments like braces or casts can also sometimes cause discomfort or skin irritation.

**What methods can help relieve pain? What are the recommended pharmacological and non-pharmacological treatments?**

If a child with DDH is in pain, there are things that can help:

- **Non-pharmacological treatments:** These are things that don't involve medicines. A warm bath or gentle massage might feel good. Sometimes, a physical therapist can teach exercises to help. If a brace or cast is causing pain, the doctor might be able to adjust it.
- **Pharmacological treatments:** These involve medicines. The doctor might recommend pain relievers like acetaminophen (Tylenol) or ibuprofen (Advil). It's important to always use these medicines exactly as the doctor says.

**What steps should I take if the pain is more than expected or worsens? Do I need to contact my doctor to adjust my treatment plan?**

If your child's pain is getting worse, or if the pain is not getting better with the treatments, it's important to call the doctor. It could mean that something needs to be adjusted. Maybe the brace isn't fitting right, or maybe the treatment plan needs to be changed. It's always better to check with the doctor if you're worried.

This is for informational purposes only. For medical advice or diagnosis, consult a professional.

**Post-Surgery Care for DDH in Children**

**What post-surgery care precautions should I take?**

After surgery for DDH, it's important to be careful and follow your doctor's instructions. This might include:

- **Keeping the hip in the correct position:** Your doctor might give you a brace or cast to wear to keep the hip in place while it heals.
- **Avoiding certain activities:** You might need to avoid activities that put stress on the hip, such as running, jumping, or playing sports.
- **Keeping the incision clean and dry:** This will help prevent infection.
- **Managing pain:** Your doctor might prescribe pain medication to help you manage any pain you're feeling.

**How long after surgery can I return to normal daily activities?**

The amount of time it takes to return to normal daily activities will vary depending on the type of surgery you had and how quickly you heal. Your doctor will give you specific instructions, but it might take several weeks or even months.

**How soon after surgery can I begin resuming activities?**

Your doctor will tell you when it's safe to start resuming activities. It's important to start slowly and gradually increase your activity level as you heal.

**What activities are recommended during the recovery period?**

Your doctor might recommend some gentle exercises to help you regain strength and flexibility in your hip. These might include:

- **Range of motion exercises:** These exercises help to keep your hip joint moving.
- **Strengthening exercises:** These exercises help to strengthen the muscles around your hip.
- **Walking:** Walking is a good way to get some exercise without putting too much stress on your hip.

**How do I schedule my exercise during the postoperative recovery period?**

Your doctor or physical therapist will help you create an exercise plan that's right for you. It's important to follow this plan carefully and not do too much too soon.

**What are the stages during postoperative recovery? What precautions should I take in each stage?**

There are typically three stages of postoperative recovery:

- **Stage 1:** This stage lasts for the first few weeks after surgery. The focus is on protecting the hip and managing pain. You'll need to avoid activities that put stress on the hip and keep the incision clean and dry.
- **Stage 2:** This stage begins after the first few weeks. The focus is on regaining strength and flexibility in the hip. You'll start doing some gentle exercises and gradually increase your activity level.
- **Stage 3:** This stage begins after a few months. The focus is on returning to normal activities. You'll continue to do exercises to maintain your strength and flexibility.

**Do I need a specialized rehabilitation plan? How does it help with recovery?**

A specialized rehabilitation plan can help you recover more quickly and safely. Your physical therapist will create a plan that's tailored to your specific needs and goals.

**How can I prevent recurrence after surgery?**

There is no way to guarantee that DDH will not recur after surgery. However, there are some things you can do to reduce your risk, such as:

- **Following your doctor's instructions carefully.**
- **Maintaining a healthy weight.**
- **Avoiding activities that put stress on the hip.**

**What complications can occur after surgery? What steps should I take?**

Some possible complications of surgery for DDH include:

- **Infection**
- **Bleeding**
- **Nerve damage**
- **Blood clots**
- **Hip dislocation**

If you experience any of these complications, it's important to see your doctor right away.

**Remember:** It's important to follow your doctor's instructions carefully and to listen to your body. If you have any questions or concerns, don't hesitate to ask your doctor or physical therapist.

It sounds like you're curious about how to best support a child with developmental dysplasia of the hip (DDH). That's great! It's important to remember that I'm an AI and can't give medical advice. Always talk to your doctor or a registered dietitian for personalized advice. They know your child's specific needs best.

Let's talk generally about some things related to recovery, using DDH as an example.

**Food and Recovery**

Think of your body like a construction site. After surgery or during recovery, it needs the right building materials to get stronger. Healthy foods are like those materials!

- **Helpful Foods:** Generally, a balanced diet with lots of fruits, vegetables, lean protein (like chicken or fish), and whole grains is a good idea. These foods provide the vitamins and minerals your body needs to heal. Imagine fruits and veggies as the "workers" bringing in the materials, and protein as the "cement" that helps build things back up.
- **Foods to Limit:** Sometimes, foods that are very processed or high in sugar can make it harder for your body to focus on healing. It's like having too much clutter on the construction site! Again, your doctor or dietitian can give you specific advice.

**Constipation**

Sometimes, kids can get constipated after surgery or while wearing a brace. Here are some things that can help:

- **Fiber-rich foods:** Think of fiber like a little "broom" that helps things move along. Fruits, vegetables, and whole grains are great sources of fiber.
- **Water:** Drinking plenty of water is super important! It helps keep everything soft and moving. Imagine trying to mix cement without enough water – it wouldn't work very well!

**Exercise and Recovery**

After surgery for DDH, your doctor or physical therapist will give you specific exercises to help your child get stronger. These exercises are like physical therapy for the hip. They'll show you how to do them safely and how often.

- **Importance of following instructions:** It's really, really important to do the exercises exactly as your doctor or physical therapist shows you. This helps make sure the hip heals correctly.
- **Precautions:** Be careful and don't try to do too much too soon. Think of it like learning to ride a bike. You start slowly and gradually get better. Your doctor or physical therapist will tell you what's safe and what to avoid.

**Sleep and Recovery**

Sleep is like a "recharging station" for your body. When you sleep, your body can focus on repairing itself.

- **DDH and sleep:** Sometimes, being in a cast or brace can make it hard to get comfortable and sleep well.
- **Tips for better sleep:** Talk to your doctor about ways to make your child more comfortable. They might have some helpful suggestions. A regular bedtime routine can also help.

Remember, every child is different, and healing takes time. Be patient, follow your doctor's instructions, and ask them any questions you have. They are there to help!

**What psychological problems may be associated with developmental dysplasia of the hip (DDH) or postoperative recovery, such as anxiety or depression? How can I deal with them positively?**

Imagine a little kid named Lily. She was born with DDH, which means her hip joint wasn't in the correct position. She had to wear a special brace for a few months. At first, she was a little sad because she couldn't move around as freely as before. But her parents helped her understand that the brace would help her hip heal properly. They made wearing the brace fun by decorating it with stickers and telling her stories about superheroes who wore special gear.

Sometimes, kids with DDH or those recovering from surgery may feel a little down or worried. They might miss playing with their friends or feel different because of their condition. It's also possible for the parents to feel anxious or overwhelmed.

But just like Lily, there are many ways to deal with these feelings positively. Here are a few things you can do:

- **Talk about your feelings:** It's okay to feel sad, frustrated, or even a little angry. Talking to your parents, friends, or a trusted adult can help you feel better.
- **Focus on the positive:** Remember that the treatment or surgery is helping you get better. Think about all the fun things you'll be able to do once you're fully recovered.
- **Find healthy distractions:** Engage in activities that make you happy, such as reading, drawing, playing games, or spending time with loved ones.
- **Practice relaxation techniques:** Deep breathing exercises, meditation, or listening to calming music can help you relax and reduce anxiety.

**What are the signs that I need psychological support? How can I find professional help or support resources?**

If you notice any of the following signs, it might be a good idea to seek psychological support:

- **Feeling sad or hopeless for a long time:** If you've been feeling down for more than a couple of weeks, it's essential to talk to someone about it.
- **Having trouble sleeping or eating:** Changes in your sleep or appetite can be a sign that you're struggling emotionally.
- **Losing interest in things you used to enjoy:** If you no longer find joy in activities you once loved, it could be a sign of depression.
- **Feeling anxious or worried most of the time:** If you're constantly feeling on edge or worrying excessively, it's essential to seek help.
- **Having difficulty concentrating:** If you find it hard to focus on schoolwork or other tasks, it could be a sign of emotional distress.

If you're experiencing any of these signs, don't hesitate to reach out for help. Here are some resources that can provide support:

- **Your parents or a trusted adult:** They can listen to your concerns and help you find professional help if needed.
- **Your doctor:** They can assess your emotional well-being and refer you to a mental health professional.
- **A school counselor:** They can provide support and guidance to students who are struggling emotionally.
- **Mental health hotlines:** These hotlines offer confidential support and can connect you with resources in your area.

Remember, seeking psychological support is a sign of strength, not weakness. Taking care of your mental health is just as important as taking care of your physical health.

**Are there any recommended health apps or devices that can help manage developmental dysplasia of the hip (DDH) or postoperative recovery?**

For DDH in children, there aren't specific apps or devices to manage the condition itself. DDH usually requires treatment like a Pavlik harness or surgery, guided by a doctor. However, some general health apps might be helpful for parents to keep track of appointments, medication schedules, or physical therapy exercises. It's always best to talk to your child's doctor about what tools might be useful in your specific situation.

**How can I use health tracking tools to monitor my condition or recovery progress?**

If your child is recovering from DDH treatment, you might use a notebook or a simple calendar to track things like:

- How long they wear their harness each day
- Any discomfort or skin issues
- Progress with physical therapy exercises
- Upcoming doctor's appointments

This can help you communicate better with your child's doctor and make sure their recovery is going smoothly. Again, it's always a good idea to discuss any tracking methods with your child's doctor to ensure they align with the treatment plan.

**What are some ways to access reliable information about developmental dysplasia of the hip (DDH) or post-operative care?**

Imagine you're building a puzzle. You wouldn't want to use pieces from a different puzzle, right? It's the same with health information. You want reliable sources, like:

- **Your doctor or healthcare provider:** They're like the instruction manual for your specific puzzle. They know your child's case best.
- **Reputable websites:** Think of these as puzzle guides. Websites from children's hospitals or medical organizations are usually good bets.
- **Books and pamphlets:** These can be like smaller picture clues, offering information in print.

**Are there support groups or community resources that can help me or my family cope better?**

Dealing with DDH can be like learning a new game with tricky rules. It can be helpful to talk to others who've played the game before:

- **Support groups:** These are like teams where families facing similar challenges can share tips and support each other.
- **Online forums:** Think of these as online game rooms where you can connect with others and ask questions.
- **Local community centers:** They may offer resources or connect you with support groups in your area.

Remember, it's okay to ask for help!

This is for informational purposes only. For medical advice or diagnosis, consult a professional.

It's tough when a kid has hip problems, and it can be expensive. Let's talk about DDH costs and how to get help.

**How much does DDH treatment cost?**

It's hard to give an exact number, but here's the deal:

- **Early treatment is cheaper:** If DDH is caught early, like with a special brace called a Pavlik harness, it usually costs less. Think of it like fixing a small crack in a phone screen – easier and cheaper than replacing the whole screen.
- **Surgery is pricier:** If DDH is more serious or caught late, surgery might be needed. This is like needing to replace the whole phone screen – more complicated and more expensive.

**Does insurance help?**

Good news! Most health insurance plans cover DDH treatment. It's like having a safety net for your wallet. But here's the catch:

- **Check your plan:** Every insurance plan is different. Some might cover more than others. It's like having different phone plans – some let you talk more, some cost less.
- **Ask your insurance company:** They can tell you exactly what's covered and what you might have to pay yourself.

**What if I can't afford treatment?**

Don't worry, there's help!

- **Government programs:** Some countries have programs that help families with medical costs. It's like getting a discount on your phone bill.
- **Charities:** There are groups that help families with sick kids. Think of them like friends who want to help you out.
- **Talk to the hospital:** Many hospitals have programs to help families who can't afford treatment.

**Remember:**

- **Early treatment is key:** The sooner DDH is caught, the better. It's like fixing a broken bone before it gets worse.
- **Don't be afraid to ask for help:** There are people and programs that can help you pay for treatment.

I hope this helps!

**How can I balance mental health, family responsibilities, and work in disease management? What are some practical suggestions?**

Imagine you're a parent with a little one who has developmental dysplasia of the hip (DDH). This means their hip joint isn't formed perfectly, and they might need to wear a special brace for a while.

This can be a lot to handle! You're probably worried about your child, making sure they're comfortable in the brace, taking them to doctor's appointments, and maybe even doing special exercises with them. On top of that, you might have other kids to take care of, a job to go to, and your own need for some "me time."

It's like juggling many balls at once! Here are some tips to help you manage:

- **Don't be afraid to ask for help.** Talk to your partner, family, or friends. Maybe they can help with childcare, cooking, or even just give you a break to relax.
- **Make a schedule.** This can help you stay organized and make sure you're not forgetting anything.
- **Take care of yourself.** It's important to eat healthy foods, get enough sleep, and find time to do things you enjoy. This will help you stay strong and have the energy to care for your child.
- **Connect with other parents.** Talking to other parents who are going through the same thing can be really helpful. You can share tips and support each other.

**How might [disease name] affect future quality of life? How can I plan ahead to improve my health?**

Let's go back to the example of DDH. If DDH isn't treated, it can sometimes lead to problems later in life, like hip pain or arthritis. But, with early diagnosis and treatment, most kids with DDH grow up to have healthy hips and can do all the things they love, like playing sports or dancing.

So, how can you plan ahead?

- **Follow your doctor's instructions.** This might include making sure your child wears their brace as prescribed, taking them to all their appointments, and doing any exercises the doctor recommends.
- **Be patient.** Treatment for DDH can take time, but it's important to stick with it.
- **Stay positive.** It can be tough to see your child going through treatment, but try to stay positive and focus on the future.

Remember, you're not alone in this. There are many resources available to help you and your child.
