# Supplementary material for: Evaluating and Validating Large Language Models for Health Education on Developmental Dysplasia of the Hip: 2-Phase Study With Expert Ratings and a Pilot Randomized Controlled Trial
Source: J Med Internet Res. 2026 Jan 19;28:e73326. doi: 10.2196/73326 (PMC12865344; doi:10.2196/73326)
Supplement: Multimedia Appendix 2 [file jmir_v28i1e73326_app2.docx]

1. Flesch–Kincaid Reading Ease:

Flesch–Kincaid Reading Ease is calculated based on the statistical method developed by Dr. Rudolf Flesch of the United States. The calculation is based on factors such as the number of words in a sentence and the number of syllables within it. The score ranges from 0 to 100, with higher numbers indicating easier-to-read text. The calculation formula is as follows:


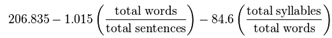


The difficulty level corresponds as follows:

0 – 30 Very difficult

30 – 50 Difficult

50 – 60 Fairly difficult

60 – 70 Standard

70 – 80 Fairly easy

80 – 90 Easy

90–100 Very easy

The readability rating provided on this page is based on this metric.

2. Flesch-Kincaid Grade Level

Flesch-Kincaid Grade Level assesses text based on U.S. elementary and secondary school grade levels. A higher score indicates greater English writing proficiency from the author, though it also demands higher reading proficiency from the reader. The calculation formula is as follows:


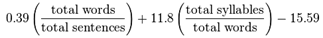


The Flesch-Kincaid Grade Level is divided into 12 grades, roughly corresponding to the reading levels of U.S. elementary and secondary school grades. For example, a score of 8.0 indicates that an eighth-grade reader can understand the document's content.

3. SMOG

The SMOG score roughly indicates the number of years of formal education required to comprehend the text. This algorithm was developed by G. Harry McLaughlin. The formula is as follows:


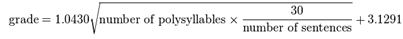


Where polysyllables refers to words containing three or more syllables.
