# Supplementary material for: Evaluating and Validating Large Language Models for Health Education on Developmental Dysplasia of the Hip: 2-Phase Study With Expert Ratings and a Pilot Randomized Controlled Trial
Source: J Med Internet Res. 2026 Jan 19;28:e73326. doi: 10.2196/73326 (PMC12865344; doi:10.2196/73326)
Supplement: Multimedia Appendix 3 [file jmir_v28i1e73326_app3.docx]

1. Which group is most commonly affected by developmental dysplasia of the hip in children?

A. Boys

B. Girls

C. Premature infants

D. Twins

E. Unknown

2. Which of the following conditions may increase the risk of developmental dysplasia of the hip in children?

A. Vaginal delivery

B. First-born child

C. Normal fetal position during pregnancy

D. Family history of the condition

E. Don't know

3. If left untreated, what complications may arise from childhood hip dysplasia?

A. Stunted growth

B. Vision problems

C. Delayed language development

D. Abnormal gait

E. Don't know

4. What is the most common treatment for infantile hip dysplasia?

A. Surgical intervention

B. Wearing corrective braces

C. Medication

D. Rehabilitation massage

E. Don't know

5. Which of the following infant holding positions helps prevent hip dysplasia?

A. Allowing the infant's legs to spread naturally in a frog-like position

B. Straightening and swaddling the infant's legs

C. Holding the infant upright for extended periods

D. Frequently placing the infant on one side for lying down

E. Don't know

6. How can parents conduct a preliminary home check for infant hip issues?

A. Observing whether the infant can roll over

B. Testing the infant's grasping ability

C. Comparing symmetry of skin creases on both thighs

D. Checking the infant's hearing

E. Don't know

7. Which of the following factors is unrelated to childhood hip dysplasia?

A. Gender

B. Birth weight

C. Fetal position

D. Parental height

E. Don't know

8. How long is a corrective brace typically worn to treat childhood hip dysplasia?

A. 1-2 weeks

B. 1-2 months

C. 3-6 months

D. Over 1 year

E. Don't know

9. At what age is hip dysplasia screening typically performed?

A. Immediately after birth

B. At one month old

C. During the 3-month checkup

D. During the 1-year checkup

E. Don't know

10. Which statement about childhood hip dysplasia is correct?

A. Only girls are affected

B. Early treatment yields better outcomes

C. Surgery is always required once diagnosed

D. It resolves spontaneously with age

E. Don't know
